# A Social-Ecological View of Barriers and Facilitators for HIV Treatment Adherence: Interviews with Puerto Rican HIV Patients

**DOI:** 10.1371/journal.pone.0125582

**Published:** 2015-09-30

**Authors:** Eida M. Castro, Lydia E. Santiago, Julio C. Jiménez, Daira Dávila-Vargas, Milagros C. Rosal

**Affiliations:** 1 Clinical Psychology Programs, Ponce School of Medicine and Health Sciences, Ponce, Puerto Rico, United States of America; 2 Psychiatry Department, Ponce School of Medicine and Health Sciences, Ponce, Puerto Rico, United States of America; 3 University of Puerto Rico Medical Science Campus School of Nursing, San Juan, Puerto Rico, United States of America; 4 University of Massachusetts Medical School, Worcester, Division of Preventive and Behavioral Medicine, Massachusetts, United States of America; University of Missouri-Kansas City, UNITED STATES

## Abstract

**Purpose:**

To identify perceived barriers and facilitators for HAART adherence among people living with HIV/AIDS in Southern Puerto Rico using a Social Ecological framework.

**Patients and Methods:**

Individual in-depths interviews were conducted with 12 HIV patients with a history of HAART non-adherence. Interviews were audio-taped and transcribed. Content analysis was performed for each transcribed interview by three independent coders using a codebook. Using Atlas TI, super-codes and families were generated to facilitate the categorization tree as well as grounded analyses and density estimates

**Results:**

Most participants reported a monthly income of $500 or less (n = 7), a high school education level (n = 7), being unemployed (n = 9) and being recipients of government health insurance (n = 11). Three out of six women reported living alone with their children and most men informed living with their parents or other relatives (n = 4). For the grounded analyses, the top four sub-categories linked to high number of quotations were mental health barriers (G = 32) followed by treatment regimen (G = 28), health system (G = 24) and interpersonal relations (G = 16). The top four sub-categories linked to high number of codes are treatment regimen (D = 4), health status perception (D = 3), interpersonal relations (D = 3) and health system (D = 3).

**Conclusion:**

The results of this study suggest the interconnection of HIV treatment adherence barriers at various system levels. Future studies on HIV treatment barriers should explore these interactions and investigate the possible synergistic effect on non-adherent behavior

## Introduction

HIV/AIDS is a chronic condition that requires a complex lifelong treatment. The goal of the Highly Active Anti-retroviral Treatment (HAART) is to achieve and maintain viral suppression, preserve immune function and stop HIV progression. A major complication of the HAART is side effects, which go from diarrhea and nausea to skin rash, lipodisthrophy and hyperglicemia among others [1]. Drug resistance is also a major concern. When a patient misses doses, the virus can become resistant to the regimen and the condition is likely to progress to AIDS. Since the HAART regimen was first used in 1996, the morbidity and mortality rates of people living with HIV/AIDS have decreased substantially, making HIV a chronic condition [2]. Great efforts have been made to make HAART available to patients; however, optimal HAART adherence is critical, but problematic [3, 4].

As of January 2014, in Puerto Rico (PR) there have been reported 45,788 HIV/AIDS cases and 25,896 reported deaths of people living with HIV/AIDS (approximately PR population, 3.5 million habitants) [4]. A total of 19,896 people are living with HIV/AIDS in Puerto Rico and, out of these, 58.5% have progressed to AIDS [5]. According to a report published by the Puerto Rico Department of Health titled “*Puerto Rico Unmet Needs 2012*”, approximately 70.94% of people living with HIV/AIDS in Puerto Rico (N = 13,748) received primary medical care during the year 2010 [6]. On the other hand, the Ponce health region of Puerto Rico, a mostly rural zone, is the third region with most HIV/AIDS cases (15% of cases, N = 6,923) when compared to the San Juan (24% of cases) and Bayamon (18%) regions located in the metropolitan area of northern Puerto Rico. In the Ponce region, however, the number of AIDS cases is significantly higher (84%, N = 5,858) when compared to the rest of the island [7]. While a number of factors contribute to progression from HIV to AIDS in Puerto Rico and Ponce region (eg. late HIV testing), poor medication adherence may account for many cases. For example, informal discussions with health care providers and HIV case managers in Southern Puerto Rico suggest that some patients, especially women, tend to miss their monthly routine and fail to pick up their prescription refills. Also, patients from distant regions travel to the southern region of the island for treatment to avoid being recognized as HIV positive patients and sometimes the distance prevents them from having timely access to their medication.

A meta-analysis conducted by Mills et al (2006) reported significantly lower adherence rates in North America (55%; 95% CI, 49% -62%) when compared with Sub-Sahara Africa (77%; 95% CI, 68% -85%) [6]. Various bio-psychosocial factors have been associated with poor medication adherence: complex drug regimen, side effects, perceived stigma, depression, self-efficacy, social support and a negative social context [8–18] (i.e. poverty-associated conditions [9]). For example, Dilorio et al (2009) tested a psychological model to explain HAART adherence and found that medication-taking behaviors are affected by the interaction of self-efficacy, depression, stigma, patient satisfaction and social support, all potentially modifiable variables [15]. However, Penn, Watermeyer and Evan (2011) concluded that even though some barriers in HAART adherence seem to be universal, others may be culturally specific such as the socio-cultural (i.e. myths and rumors), economic (i.e. poor financial security) and systemic factors (i.e. poor communication between hospital and clinics) [11]. Former models of medication adherence have focused on patient level barriers, however, the field is moving forward with more systemic models in order to gain a more comprehensive understanding of this problem [19].

The purpose of this study was to identify perceived barriers and facilitators for HAART adherence among people living with HIV/AIDS in Southern Puerto Rico. The data presented in this article is part of a three-phase, mixed method study aimed at determining predictors of HAART non-adherence in Puerto Rican people living with HIV.

## Material and Methods

### Study design

We used qualitative methods (key informant interviews) and a social ecological framework to gain in-depth understanding of perceived system barriers (i.e. individual, micro-system, meso-system, exo-system, macro-system and crono-system.) associated with HAART adherence. The purpose was to focus on the phenomenological aspect of voluntarily missing doses in the context of a systemic socio-ecological perspective. Thus, instead of the quantification of medication adherence we inquired about the subject’s perceived experience regardless of time and frequency. Findings will report on the development of a HAART model of adherence and future interventions to reduce HIV/AIDS disparities in Puerto Rico.

### Ethic Statement

Ethical approval was granted by the Ponce School of Medicine and Health Science (protocol number 120522-EC) and the University of Puerto Rico, Medical Science Campus (protocol number A8160112). Prior to the interview, the study was explained to the participant and an opportunity to ask questions was provided. Written informed consent was obtained from each participant. Confidentiality was kept by using a study identification number, rather than subjects' names. During the interviews, participants were asked to change their name in order to minimize risk of identification, however, all of them decided not to mention their name during the interviews. Since there were no names recorded during the in-depth interviews subjects were identified as “participant #1”, “participant # 2”, etc. Data was kept in a locked filing cabinet and was available only to the researcher.

### Study sample

A mixed purposeful sampling method was employed (stratified and criterion) in order to facilitate data triangulation [20]. Participants were stratified by gender (6 women and 6 men) and risk group (injection drug user (n = 4), men who have sex with men (n = 4), heterosexual (n = 4)). Considering the phenomenological nature of the study, the recruited sample size provided enough data to reach a descriptive saturation. The inclusion criteria were: 1) 21 years of age and older, 2) receiving HAART treatment at the time of the study, 3) a history of HAART non-adherence for at least one month as reported by the health care provider or case manager based on clinical record notes and, 4) willing to provide voluntary consent. Participants were recruited from clinics and community and government based organizations (C/GBO) that provide health services to patients living with HIV/AIDS in the southern region of Puerto Rico. Before beginning the recruitment process, the principal investigator met with the clinic administrators, health care providers and case managers to discuss the study objectives, inclusion/exclusion criteria and to design a recruitment process. Case managers and health care providers who invited patients to participate in the study identified potential participants. The principal investigator discussed the informed consent and conducted all the interviews.

### Interview guide

**Fig 1:**
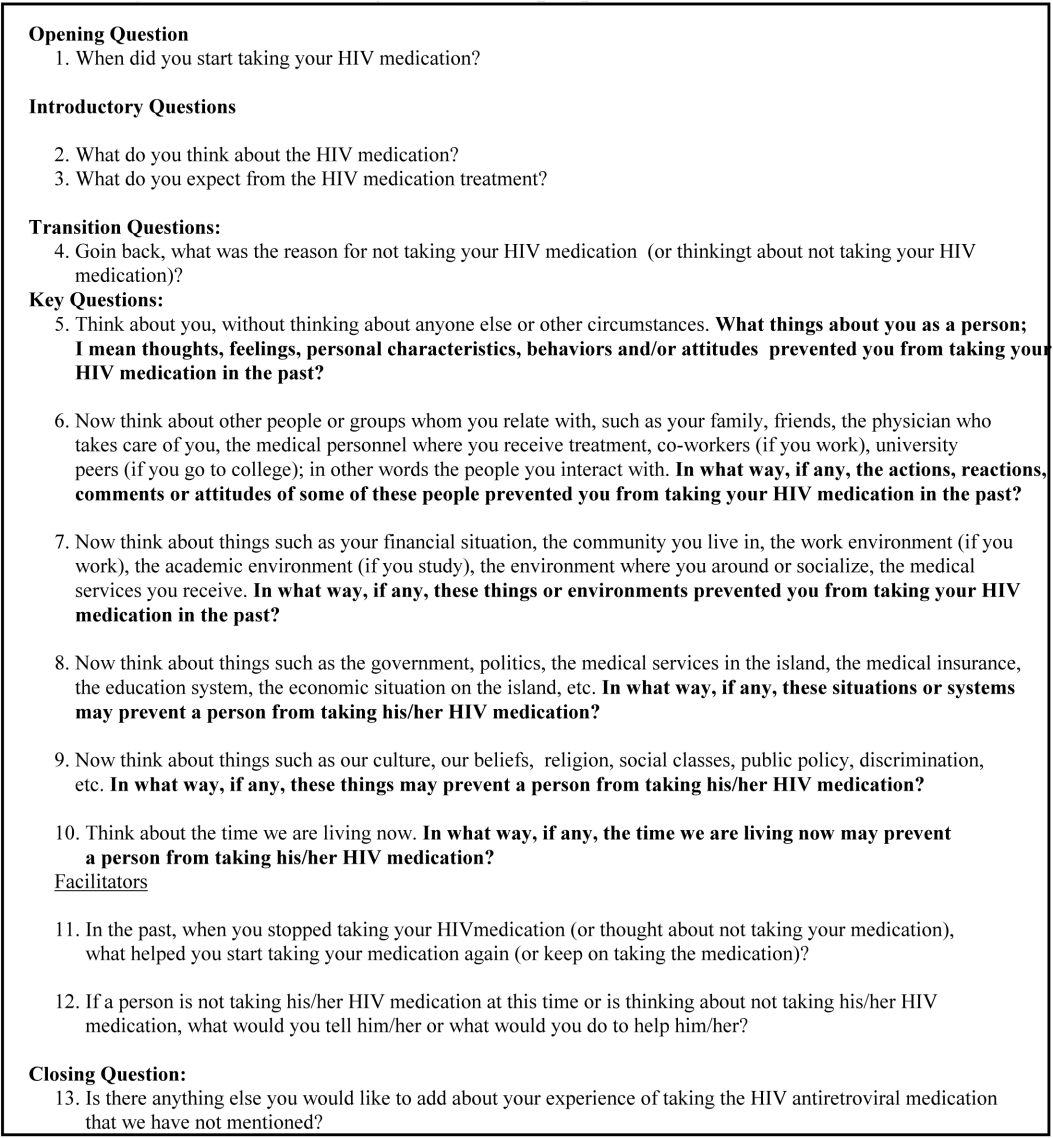
Interview guide (Prompt questions not included).

The principal investigator and three members of the research team, with expertise in qualitative research design, developed the interview guide (Fig 1). Questions were designed following the research question and the framework of the Social Ecological Model and techniques proposed by Kvale (1996) [21] (e.g. introducing questions, probes, etc.). The interview guide was pilot-tested with two adult HIV patients for clarity and relevance of the questions, which resulted in slight modifications of some of the questions and the addition of prompts. Questions were open ended in order to facilitate participants’ insightful response and prompts were used to assist in focused elaboration and depth in participants’ responses. Questions addressed patient-perceived barriers and facilitators of HAART adherence at various systems’ levels (e.i. individual, micro-system, meso-system, etc.).

### Data Collection

Face-to-face in-depth interviews were conducted using an open-ended question guide to ask about the barriers for HAART non-adherence at different system levels (individual, micro-system, meso-system, etc.). It also inquired about the facilitators to HIV treatment adherence in order to identify opportunities for interventions. This process took approximately 40 minutes. A $15 stipend was provided to participants to compensate for their time and effort. The interviews were audio-recorded, transcribed by a research assistant for analysis and translated into English for publication purposes.

### Data Analysis

Transcribed interviews were analyzed using a combination of hand coding and Atlas TI (Hapberg, Germany), a software program designed to facilitate the analysis of qualitative data. Content analysis was performed for each transcribed interview by three independent coders (the principal investigator and two trained research assistants) as a form of investigator triangulation. An initial list of themes was identified and coded by the principal investigator, a process guided by the theoretical framework described above. A codebook was developed to further define and operationalize each of the themes. New themes and concepts were added to the codebook as they emerged from the transcripts. Two additional members of the research team with expertise in qualitative data analysis coded each transcript independently, coders subsequently met to discuss codes and categories and to further establish the inter-coder agreement. In other words, after all members finished coding the transcript, they met to compare their analysis and discuss discrepancies. Disagreements were discussed until consensus was reached. Using Atlas TI, super-codes and families were generated to facilitate the categorization tree. Categories and sub-categories (i.e. Micro-system level barriers and peer influence, etc.) were derived from initial codes. Grounded analyses and density estimates were also performed. Grounded analysis (G) is the number of quotations assigned to each code. Density (D) is the number of codes assigned to each category.

## Results

**Fig 2:**
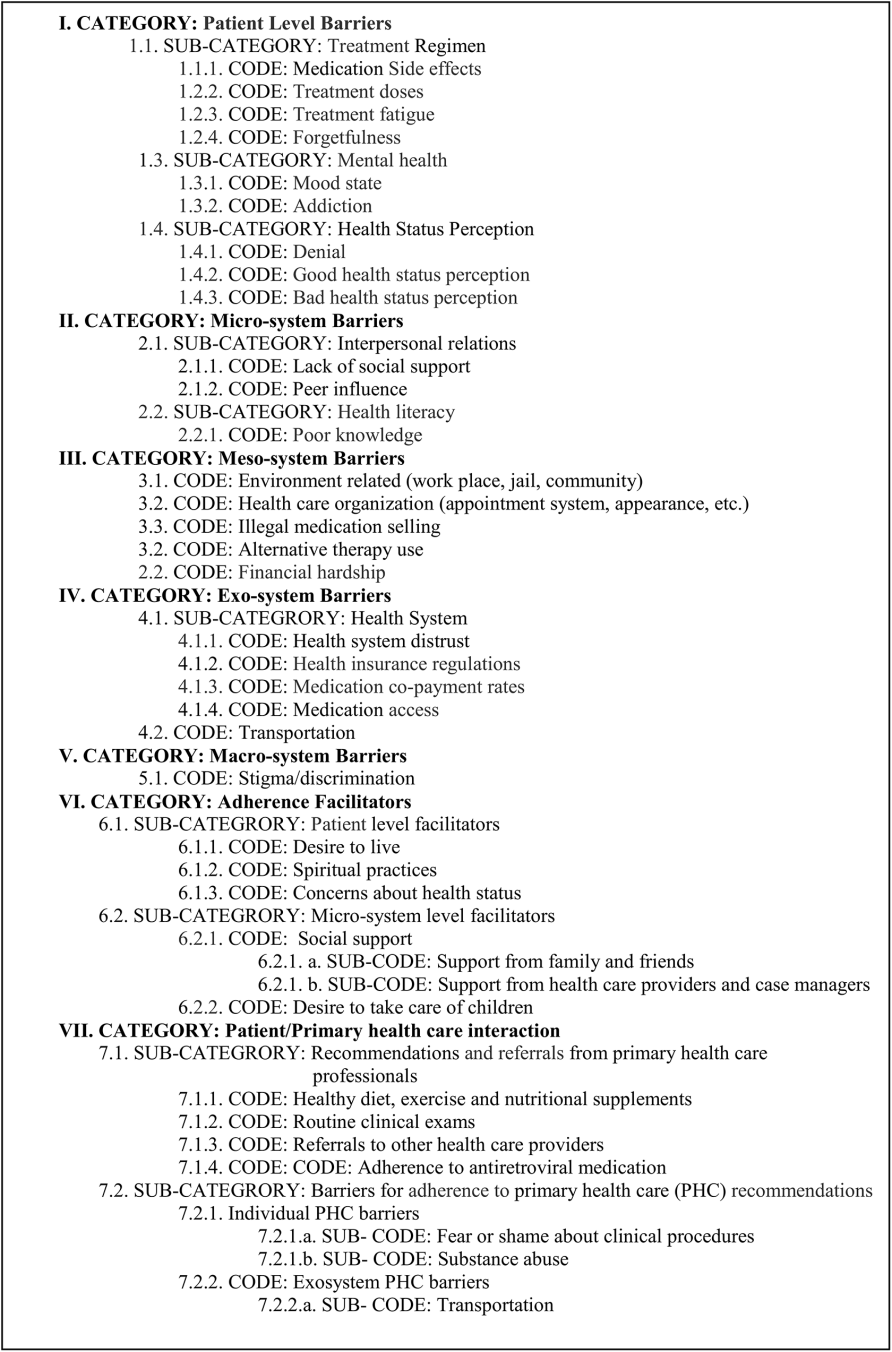
Emergent themes by category.

Twelve in-depth interviews were conducted with HIV/AIDS patients who voluntarily missed medication doses at any time during the course of their treatment (6 women and 6 men)**.** Participants’ characteristics are summarized in Table 1. Mean age was 40.5 (SD = 11.41) for women and 39.5 (SD = 5.54) for men. Most participants reported a monthly income of $500 or less (n = 8), a high school education level (n = 6), being unemployed (n = 9) and being recipients of government health insurance (n = 11). Three out of six women reported living alone with their children and most men informed living with their parents or other relatives (n = 4). On the other hand, the qualitative analysis of transcripts summarized in Fig 2 revealed seven category themes corresponding to various system level barriers to HAART adherence as well as facilitators.

**Table 1:** Participants’ characteristics.

Variable		Frequency (%)
**Sex**	Male	6 (50%)
	Female	6 (50%)
**Living with**	Spouse/partner	3 (25%)
	Parents (or one of them)	3 (25%)
	Siblings	3 (25%)
	Other family members	2 (16.7%)
	Alone	1 (8.3%)
**Vocational status**	Employed (either part or full time)	2 (16.7%)
	unemployed	9 (75%)
	Student	1 (8.3%)
**Academic history**	Middle school	4 (33.3%)
	High School	6 (50%)
	Technical/Associate degree	2 (16.7%)
**Civil status**	Never married	5 (41.7%)
	Living together (not married)	3 (25.0%)
	Married but living separate	1 (8.3%)
	Divorced	2 (16.7%)
	Widow	1 (8.3%)
**Sexual preference**	Same sex	3 (25%)
	Opposite sex	8 (66.7%)
	Both sexes	1 (8.3%)
**Monthly Income**	$500 or less	8 (66.7%)
	$501 to $999	3 (25%)
	$1,000 to 1,500	1 (8.3%)

Grounded (G) and density analyses (D) for HAART adherence barriers and facilitators are summarized in Table 2. For the grounded analyses, the top four sub-categories linked to high number of quotations were mental health barriers (G = 32) followed by treatment regimen (G = 28), health system (G = 24) and interpersonal relations (G = 15). The top four sub-categories linked to high number of codes are treatment regimen (D = 4), health status perception (D = 3), interpersonal relations (D = 3) and health system (D = 3).

**Table 2:** Grounded and density analyses of HAART adherence barriers and facilitators.

**HAART Adherence Barriers**		**Grounded (G)**	**Density (D)**
Patient level (G = 69; D = 9)	Treatment regimen	28	4
	Mental health	32	2
	Health Status perception	9	3
Micro-system level (G = 21; D = 4)	Interpersonal relations	15	3
	Health literacy	5	1
Meso-system level (G = 19; D = 5)	Environment related	8	1
	Health care organization	2	1
	Illegal medication selling	5	1
	Alternative therapy use	2	1
	Financial hardship	2	1
Exo-system level (G = 33; D = 4)	Health System	24	3
	Transportation	9	1
Macro-system barriers (G = 12; D = 1)	Stigma & discrimination	12	1
**HAART Adherence Facilitators**		**Grounded (G)**	**Density (D)**
Patient level (G = 18; D = 3)	Desire to live	4	1
	Spiritual practice/beliefs	4	1
	Concern about health status	10	1
Micro-system level (G = 25; D = 3)	Social support (family/friends and clinical personnel)	18	2
	Desire to take care of children	7	1

### Patients’ perception of level barriers for HAART adherence

Patient level HAART adherence barriers were those related to the participant’s personal characteristics, emotions, behaviors and perceptions. When participants were asked about how their thoughts, feeling or personal characteristics might have prevented them in the past from taking the HAART medication, they described a list of factors summarized in these categories: treatment regimen, mental health issues and health status perception. A total of 69 quotations (grounded analysis) and 9 codes (density analysis) were linked to the patient level category. As expected, medication side effects, a treatment regimen sub-category, emerged as an adherence barrier. The following verbatim depict the anticipatory belief that medication side effects will make the participant sick, associating emesis as a medication side effect to a sick status.

Participant 8: “*So that's why I stopped taking them [the HAART medication]*. *Because*, *I'll be honest*, *yesterday they changed my meds and today I had breakfast and had that in my mind*, *and I said I'm going to take them*, *I'm going to take them but then I have also in mind that I will become ill*, *throwing up and those other things”*

This participant, on the other hand, stopped taking the HAART medication after a considerate weight gain:

Participant 11: “*I ate a lot and became very fat*. *Wow*, *I used to weight 140 pounds*. *I have never weighed that much*. *I said*, *Oh*, *no*, *this can’t be and I stopped taking the [HAART] medication*.*”*

Another expected theme found was the interaction between mental health issues and HAART medication adherence. Various patients, such as the one below, talked about depression:

Participant 12:*“Ah*, *sometimes when I’m depressed I don’t take them [HAART medication]*.*”*

Other patients talked about the interference of addictive behavior with HAART non-adherence:

Participant 4: *“When you are part of the “drug” environment*, *you become irresponsible for your health*, *irresponsible with others*, *and even irresponsible with the doctor’s appointments and all the costs*.*”*

Participant 8: *“When I drink [alcohol] then*, *I don’t mix the [HAART] pills because that is not good*, *mixing the two things”*

Surprisingly, some participants attributed good and bad health status perception to medication non-adherence. The following verbatim was from a patient who had not taken her medication for 20 years and this is what encouraged her non-adherence:

Participant 10:*“Nothing*, *I didn’t get sick*, *not even a cold*. *I knew I was HIV positive but*, *the first thing that came to my mind was that I didn’t have anything*.*”*

Another participant narrated how every time she took the HAART medication regimen she felt her health was deteriorating.

Participant 8:*“Sometimes negative thoughts come to my mind and I say- why I am going to keep taking the medication if I am falling behind [not getting well]”*

### Patients’ perception of micro-system barriers for HAART adherence

Micro-system level barriers are those factors related to interpersonal face-to-face relationships manifested in different contexts. Grounded value for this category is 69 and density value was 4. The subcategories that emerged under this theme are barriers related to interpersonal relations and health literacy.

A female patient explains how feeling alone due to lack of social support was a catalyst for not taking her medication.

*Participant 11*: *“Well*, *it always affected me because I was always alone*. *My mother was never with me and my father neither*. *I don’t know*, *I always felt alone like I didn’t matter to anyone…”*

An injection drug user shares his experience about how peers influenced his decision not to take the HAART medication.

Participant 3: [peers to participant] “*Are you going to waste time looking for that [meds]*?. *Is like*, *any way we were using drugs that [medication] is not going to have any effect…I listened to them and let myself [influence] by what they said”*

### Patients’ perception of meso-system barriers for HAART adherence

Meso-systems are the interaction of two micro-systems containing the subject. In order to identify these interactions, participants were asked how their social environments might have influenced their non-adherent behavior. They identified the following barriers: environment related, health care organization, illegal medication selling, alternative therapy and economic hardship. Grounded value for this category is 19 and density value was 5.

This female patient shares her experience of the first time she was sentenced to serve time in jail for a crime she committed. She talks about how being in jail and away from her children was the precipitating factors for becoming depressed and not taking the HAART medication.

Participant 6: *“Look*, *I was an inmate; I got out of jail six or seven months ago*. *Inside there I was depressed; first time in my life [in jail] and I fell behind and behind*…*I was given the medications and the doctor and the nurse would tell me to take them and I faked it; afterwards I took them out of my mouth…I waited until I was outside or another place and I took them out and hid them*. *I didn’t drink them*. *Sometimes I had a lot of little [pill] envelopes and I was like “oh my God*, *wait a minute*, *if they come to do an inspection or something and they inspect the cell they will find it*. *I would take them and throw them out*.*”*

Surprisingly, two participants mentioned HAART medication being sold in the illegal street market.

*Participant 12*: *They sell them*.

*Interviewer*: *They sell the medications*? *Do they have clients to sell them to*?

*Participant 12*: *They sell them for…for drugs…*

*Interviewer*: *And who would buy the medication*?

*Participant 12*: *“Los de los puntos” (Drug Lords)*

### Patients’ perception of exo-system barriers for HAART adherence

Exo-system barriers are those taking place between two or more contexts, at least one of which does not contains the patient. For example, exo-system level health system barriers emerging from patients’ narratives included the health system, particularly medication access, distrust and co-payment rates. Transportation was another common barrier identified. Grounded value for this category is 33 and density value is 4.

Sometimes the bureaucratic health system procedures and regulations make it difficult to provide medication on time. The following quote came from a patient, beneficiary of the government health insurance plan, who had to wait for the medication to be approved.

Patient 3: *“But what happens now is that they are taking two or three months to approve some medications*, *already the treatment is being interrupted abruptly*. *That is what is happening*.*”*

Some participants stated that there are times when the HAART medication is no available in the pharmacies.

Participant 1: “*I needed another medication*. *I need to look for it because last month it wasn’t available*.”

Participant 8: “*Well*, *we were without medication*, *I mean*, *usually they solve it quickly but*, *sometimes we are two or three days without taking the medicine*.”

### Patients’ perception of macro-system barriers for HAART adherence

Macro-system barriers are those patterns embedded in the culture or subculture and manifested in a continuum of micro-meso-exo systems. As expected, real and perceived stigma and discrimination was the only macro-system barrier identified in the narratives. Some participants talked about their experience of being branded and rejected and others described the experience of other fellow patients. Grounded value for this category is 12 and density value is 1.

A woman reported her experience of being rejected by family members and neighbors, after they found out she was HIV positive. She felt ashamed and traumatized with the experience.

Participant 10: *“…everybody found out*, *and everybody looked at me like*

*“look*, *she has AIDS*, *don’t go near her*, *so on”*, *and that*

traumatized me even more and that’s why I was in denial with the

pills…”

Sometimes, fear of being rejected might complicate the medication-taking routine and doses may be missed. This woman described how difficult it was to keep up with the medication schedule when she was at work.

Participant 8: *“I couldn’t take them [the pills] because my boss and the co-workers were around*. *Other times I would have to hide but*, *I would take the pills hours later [after the indicated hour]*.

Another participant shared the story of a friend who was HIV positive and was rejected by member of the community. Apparently, after the neighbors found out he was HIV positive they yelled at him and mocked him. He then refused to take the HAART medication.

Participant 4: *“My friend*, *he is constantly yelled at by the neighbors*. *[the participant tells him] go to the clinic [to continue with the HIV treatment] and he says…ah*, *not anymore*.*”*

### HAART adherence facilitators

Participants were asked about the factors or situations that helped them resume their HAART regimen. The identified facilitators were grouped around a multi-level ecological perspective. Their responses were categorized under patient level facilitators and micro-system level facilitators. The majority of the quotes fell under the micro-level facilitators (G = 25). Surprisingly, concerns about health status were the most quoted code (G = 10) under participant level facilitators, when compared to other factors at the patient level (desire to live, G = 4; spiritual beliefs/practices, G = 4). Grounded value for this category is 25 and density value is 3.

Desire to live, spiritual practices and beliefs along with participants’ concerns about poor health status were the patient levels facilitators identified. To our surprise, some participants waited until their viral load was high, and opportunistic conditions developed, to take their HAART medication.

#### Patient level facilitators

Patient level facilitators include the individual’s desire to live (G = 4), spiritual practices (G = 4) and concerns about health status (G = 10). The most quoted facilitator by participants was concern with health status. In other words, once patients experienced physical symptoms as a consequence of viral replication due to non-adherence, they became worried about their health or even scared and started taking their medication. The following participant described his health condition when he returned to the clinic after a long period without taking his medication. He was so deteriorated that the doctor told him he might not survive.

Participant 13: *“When I returned here [the HIV clinic] I looked liked Jesus Christ in the cross*, *so skinny that my ribs would show off*. *Here [the HIV clinic] they raised me up again”*

Some participants take long breaks from taking the medication and start the regimen again once they feel their health is starting to deteriorate.

Participant 12: *“I felt weak…sometimes when I stop taking them I feel like*, *Oh my God*, *so tired and then I realize I have to start taking them again*.*”*

#### Micro-system level facilitators

As expected, social support emerged as the most cited form of HAART adherence facilitator. According to participants, family, friends, neighbors, clinicians and case managers among others provided support. Another facilitator, mostly quoted by females, was the desire to become healthy again so they can take care of their children. The following quote depicts this facilitator:

Participant 11: *“My children; I’m constantly thinking about them*. *I have two children; they are very healthy and they are the reason why I exist*.*”*

### Primary health care experience

The purpose of exploring this area was to inquire about participants’ barriers for not following up on primary health care (PHC) recommendations. We first asked about the clinical recommendations provided by their physicians and then queried them about challenges related to follow up recommendations. Recommendations ranged from HAART medication adherence to those related to healthy diet and exercise. Other recommendations were routine clinical laboratory tests and referrals to other health care professionals. For a complete list of recommendation codes refer to Fig 2. In general, most participants said they followed the PHC recommendations. However, some reported barriers for not following these recommendations. The identified barriers fell under the patient level and the exo-system level.

#### Patient level barriers for PHC recommendations

Patient level barriers for PHC recommendation adherence were fear or shame about clinical procedures and substance abuse. For example, one male participant was afraid of dental procedures and another female participant felt ashamed about gynecological procedures.

Participant 1: *“The PAP test is difficult*, *I feel ashamed*. *It’s been a lot of years [without performing a PAP smear test]*

Participant 4: *“It’s difficult [to follow the recommendation referral to] the dentist because I’m afraid…”*

Exo-system level barrier for PHC recommendations

The only exo-system barriers to PHC recommendation adherence were related to transportation limitations. The following quote emphasized the problem.

Participant 10: *What prevents me from following the [laboratory] tests is the [problem with] transportation*. *Where I live is not near…you have to walk a lot…”*

## Discussion

The purpose of this study was to identify perceived HAART adherence barriers and facilitators in a sample of HIV patients with a history non-adherent behavior. The study was framed under a social ecological framework; an approach that facilitates the understanding of medication adherence behavior by studying the interaction of socio-ecological systems: individual, micro-system, meso-system, exo-system, macro-system and crono-system [19]. A total of twelve HIV patients (6 women and 6 men) participated in the study providing an interesting range of responses. The social ecological perspective framework was used for the guided interviews and the emergent categories falling under these system level categories: patient level barriers, micro-system level, meso-system level, exo-system level and macro-system level. Table 3 summarizes key findings and implications.

**Table 3:** Key Findings and implications for HAART adherence barriers and facilitators.

Key Findings	Implications
1. Patient level medication adherence barriers were the most commonly cited (G = 69) challenge.	a) Studies looking at the interactions of these barriers with the adherence behavior of Puerto Rican patients are warranted.
2. Within the patient level barriers, mental health factors are still very prevalent.	b) Mental health, particularly depression and addictions, is a common barrier identified in the literature.
	c) Screening efforts should be reinforced.
3. Exo-system level barriers are the second most cited category (G = 33)	d) Unfolding the role of health system barriers in HAART adherence will shed light to challenges beyond patients’ control.
	e) Future studies should include perspectives of actors representing different areas of the health system (e.g. health care providers, administrators, case managers, etc.).
4. Within the exo-system level barriers, those related to the health system were more common.	f) Health system barriers are usually beyond the patients’ control.
5. Medication adherence facilitators fell into two categories: patient level and micro-system level.	g) Comprehensive studies on adherence facilitators should inquire about other system level factors (e.g. meso-system, exo-system, etc.).
6. Social support remains a common medication adherence facilitator	h) Social support is a facilitator widely studied in the literature. Intervention efforts should consider this facilitator.
7. Concerns about health status, particularly health deterioration, was the second most common medication adherence facilitator	i) This facilitator may be a dangerous one, particularly if patients wait until their health is deteriorated to start taking their medication.
8. HAART adherence is a complex challenge engaging multi-level systems.	j) Studying multiple systems’ levels of barriers to HIV treatment adherence can better guide the development of more comprehensive interventions.

Some of the emergent barriers have been widely cited in the literature; among these are medication side effects [22], treatment regimen [23], depression [24], transportation, stigma [25], and drug addiction [26]. Other barriers have been less studied, for example, peer influence, a good or bad health status perception, and illegal medication selling [9]. For example, medication side effects and treatment regimen (e.g. inconvenient scheduling), among other factors, may lead to treatment fatigue and subsequent non-adherence; yet, this phenomenon is not widely addressed [27]. Studies on medication side effects and regimen as proxies for HAART treatment fatigue and adherence, and opportunities for interventions are warranted. Depression is a very common barrier identified in the literature. As such, Gonzalez et. al. conducted a study looking at the psychosocial and cultural factor associated with depression in a sample Hispanic men diagnosed with HIV [28]. The investigators identified several correlates of depression such as stress, self-esteem, substance abuse and physical violence, and recommended further research to determine how interactions of these variables impact medication adherence. Regarding perception of health status, Cardarelli and collaborators found that HIV patients (including Hispanics) when rating their general health as fair/poor were 4 times more likely to be non-adherent to HAART (odds ratio [OR], 4.34; 95% confidence interval [CI], 1.19–15.79) [29].

HIV treatment non-adherence has been studied widely [8] and efforts are currently concentrated in the creation and implementation of interventions to promote adherence [30], including the incorporation of technology-assisted interventions [31]. The field has moved forward significantly but, in order to maximize these efforts and become more efficient, it is imperative to incorporate a systemic approach for a more comprehensive understanding of the phenomenon and, thus, develop more effective intervention [19].

Our findings suggest that HAART medication non-adherence is a complex problem engaging multi-level system factors into the equation. For example, patient level barriers manifest and interact with other systems as in the case of patients with depression (patient level) encountering stigmatizing discrimination (exo-system) and lack of social support (micro-system), sinking deeper into depression, thus, engaging in HAART treatment non-adherent behavior. Another hypothetical example is when a drug addict patient (patient level) takes peer advice of not taking the medication (micro-system), and gets access to an illegal antiretroviral medication market (meso-system), thus, becoming a burden to the health system (exo-system). Even though these are hypothetical cases they depict the complex, interacting multi-level factors interweaving with the problem of HAART non-adherence.

One way we analyzed the data was by looking at the number of quotations assigned to a code or category (G = grounded analysis). This level of analysis gave us the opportunity to identify the most commonly categories cited by the participants, giving us an approximation of category saturation. The two most cited barriers for adherence were mental health factors (e.g. depression, substance abuse, G = 35) and treatment regimen (G = 28) which are also common barriers to non-adherence. Depression has been identified as one of the most difficult barriers for medication adherence not only for antiretroviral treatment [13], but for the treatment of other medical conditions as well [32–33]. A systematic review conducted by Lowther et. Al (2014) revealed a high point prevalence of depression (33.6%) among people living with HIV under treatment, thus, increasing the risk of HAART non-adherence [34]. On the other hand, addiction is another common challenging barrier for optimal antiretroviral adherence [35]. Surprisingly, the third most commonly cited barrier was related to the health system (e.g. medication access, medication co-payment, etc.). This finding warrants further exploration considering that health system level barriers are not under the patient or even health care provider’s control. For example, one of the situations cited by participants was that they had to wait for the medication to be available in the pharmacy, thus, not being able to take their medication. Another situation was related to health insurance coverage, particularly being unable to cover medication co-payment. Verification of these stories was out of the scope of the study; however, future projects should include the perspective of health system administrators or stakeholders for a more comprehensive exploration of this apparent barrier. Other commonly cited barriers were related to interpersonal relations (e.g interpersonal conflict, peer pressure, G = 16) and stigma (e.g. social and internalized, G = 12). Interpersonal relations is a factor that needs further exploration as it suggests interpersonal conflict or peer pressure as a potential proxy for non-adherent behavior. This factor should not be explored by itself but as part of a cluster of other system level factors (personal, macro-system, etc.) that may be potentiating a synergistic effect for treatment non-adherence. Stigma, on the other hand, has been widely proven to be a risk factor for HAART non-adherence [33].

One of the goals of this study was to identify HAART adherence facilitators. Different from adherence barrier, adherence facilitator fell into two system level categories: patient level and micro-system level facilitators. One reason this might have happened is that the interview guided participants in barrier categories to talk about their experiences by responding to questions already contextualized into each system level, while using a single question to ask for facilitator experiences (refer to Fig 1). A recommendation for future studies is to explore the existence of other system level facilitators for HAART treatment adherence by incorporating other types of questions or interviewing stakeholders or actors representing multiple systems (e.g. clinicians, administrators, etc.).

Adherence facilitator categories were also subject of grounded analysis. As expected, social support was the most common cited facilitator for adherence (G = 18) by participants. Other studies have identified social support as a potential HAART adherence facilitator. For example, a cohort study conducted by Achieng et. al. (2012) looking for facilitators of retention in care and antiretroviral treatment adherence in Kenya found that among other factors, participation in support groups predicted better adherence, and time to treatment failure reduction [36]. One interesting finding about adherence facilitators was the emergence of concerns with health status (G = 10). These concerns were either imaginary (fear of health deterioration) or real (actual health deterioration). The fact that some participants waited until their health deteriorated to take their medication is like playing at the “Russian roulette”. It represents a very dangerous facilitator because the patient’s life is placed at risk, and it further complicates their care. Patients relying on this facilitator are probably using the psychological defense mechanism of denial, making contact with the reality of their diagnosis only when the physical signs of an opportunistic condition appear. The development of effective strategies to identify and tackle this dangerous facilitator is recommended.

Questions about primary health care (PHC) interactions were added in this study in an effort to expand our understanding of PHC recommendation adherence among participants. Some participants identified patient level barriers, such as fear or shame about certain clinical procedures and substance abuse, as well as exo-system level barriers, such as transportation problems. However, we did not find enough evidence of saturation of barriers to PHC recommendations, as most participants stated they followed their primary health care recommendations. The identified barriers are common in the general populations as well [37–38]. One potential explanation is that some PHC services provided to the patients were incorporated in the clinic where recruitment took place, thus, facilitating access and further adherence of such recommendations.

### Strengths and Limitations

One of the strengths of this study is the use of a social ecological model to provide a comprehensive view of the HAART non-adherence phenomenon. This approach has been recommended elsewhere [18] and we have received a broader response from participants by using a comprehensive interview guide. A limitation of the study was having only one recruitment site, thus limiting the emergence of other system barriers or facilitators attached to other site realities. Another limitation was the sample size. Although twelve is an acceptable number of participants for a descriptive study, it may not be enough to reach saturation of certain phenomena; such was the case of PHC recommendation barriers.

## Conclusion

The results of this study suggest the interconnection of HIV treatment adherence barriers at various system levels. Future studies on HIV treatment barriers should explore these interactions and investigate the possible synergistic effect on non-adherent behavior. Studying multiple system levels of barriers to HIV treatment adherence can better guide the development of more comprehensive interventions.

## Supporting Information

S1 Dataset Raw Qualitative Data. (DOC)
