# Supplementary material for: A Social-Ecological View of Barriers and Facilitators for HIV Treatment Adherence: Interviews with Puerto Rican HIV Patients
Source: PLoS One. 2015 Sep 30;10(9):e0125582. doi: 10.1371/journal.pone.0125582 (PMC4589346; doi:10.1371/journal.pone.0125582)
Supplement: S1 Dataset — (DOC) [file pone.0125582.s001.doc]

Raw Qualitative Data: In-depth Interviews transcriptions

Project: Psychosocial barriers to HIV treatment adherence among people with HIV/AIDS (PWHA) living in the Ponce health region

PI: Eida M. Castro, PsyD., MSc.

Entrevista #1

**ENTREVISTADOR:** Entrevista número uno, fase uno, proyecto Adherencia a Tratamiento. Okay, ya entonces discutimos el consentimiento y pues lo firmaste como que estás de acuerdo en participar del estudio, ¿verdad?

**Participante femenina 1:** Sí.

**ENTREVISTADOR:** Vamos a comenzar entonces la entrevista. ¿Desde cuándo tú estás tomando los medicamentos para el VIH? ¿Cuánto tiempo hace que estas tomándotelos?

**Participante Femenina 1:** Hace quince años.

**ENTREVISTADOR:** QUINCE AÑOS, OKAY. Y, ¿EMPEZASTE A TOMARTE LOS MEDICAMENTOS CUANDO TE DIERON EL DIAGNOSTICO O PASÓ ALGÚN TIEMPO?

**Participante Femenina 1:** Cuando me diagnosticaron que era VIH. Rápido.

**ENTREVISTADOR:** INMEDIATAMENTE. OKAY, ¿CUÁNTO TIEMPO MÁS O MENOS…? ¿INMEDIATAMENTE TE LO RECETARON?

**Participante Femenina 1:** Como dos meses.

**ENTREVISTADOR:** OKAY.

**Participante Femenina 1:** Cuando caí en el hospital porque tenía una condición. Me mandaron para acá y rapidito…

**ENTREVISTADOR:** AHÍ EMPEZASTE A TOMAR… Y EN EL LUGAR DONDE TE ESTABAN TRATANDO, EN AQUEL MOMENTO, ¿TENÍAN LOS MEDICAMENTOS PARA TRATAR EL VIH? ¿LOS TENÍAN AHÍ DISPONIBLES?

**Participante Femenina 1:** Aquí fue que yo vine nada más…

**ENTREVISTADOR:** SI, OKAY. ¿Y LOS TENÍAN DISPONIBLE AHÍ O…?

**Participante Femenina 1:** Sí.

**ENTREVISTADOR:** ¿…O TUVISTE QUE ESPERAR QUE LOS VINIERAN A TRAER?

**Participante Femenina 1:** No, rapidito.

**ENTREVISTADOR:** ¿Y CUÁNDO TE DIERON ESOS MEDICAMENTOS, VERDAD, LEES LA INFORMACIÓN QUE TE DICE LOS EFECTOS SECUNDARIOS Y TODO ESO DE LOS MEDICAMENTOS?

**Participante Femenina 1:** Sí.

**ENTREVISTADOR:** ESA INFORMACIÓN, ¿LA LEÍSTE O TE LA…?

**Participante Femenina 1:** Si las leí.

**ENTREVISTADOR:** LAS LEÍSTE.

**Participante Femenina 1:** Y me explicaron.

**ENTREVISTADOR:** AH Y TE LA EXPLICARON. ¿TE ACUERDAS DE LOS EFECTOS SECUNDARIOS QUE TENÍAN?

**Participante Femenina 1:** Sí.

**ENTREVISTADOR:** ¿CUÁLES ERAN, POR EJEMPLO?

**Participante Femenina 1:** Dolor de estómago, nauseas, vómitos, mareos.

**ENTREVISTADOR:** ¿TE DIERON ESOS EFECTOS?

**Participante Femenina 1:** Sí. Dolor de cabeza.

**ENTREVISTADOR:** AJA.

**Participante Femenina 1:** A veces no podía caminar casi. Me caían mal.

**ENTREVISTADOR:** ¿Y QUE TÚ PIENSAS DE LOS MEDICAMENTOS PARA TRATAR EL VIH? LO PRIMERO QUE TE VIENE A LA MENTE CUANDO TE HABLAN ASÍ DE ESOS TRATAMIENTOS.

**Participante Femenina 1:** Por ejemplo para mí, los medicamentos que me dieron al principio eran bien fuertes, pero pues ayudan mucho a uno porque te bajan la carga viral. Yo tenía las defensas bien bajas.

**ENTREVISTADOR:** Y, ¿QUÉ TÚ ESPERAS DEL TRATAMIENTO? ¿CUÁL ES TU EXPECTATIVA, LO QUE TÚ ESPERAS DE ESE TRATAMIENTO PARA EL VIH?

**Participante Femenina 1:** Una mejor calidad de vida…vivir más tiempo.

**ENTREVISTADOR:** CUANDO ESCUCHAS COMENTARIOS DE OTRAS PERSONAS QUE HAN DEJADO DE TOMARSE LOS MEDICAMENTOS PARA EL TRATAMIENTO DEL VIH, QUE PIENSAS

**Participante Femenina 1**: Que no quieren vivir. No quieren vivir o no le importa nada.

**ENTREVISTADOR:** ALGO MÁS QUE TE VIENE A LA MENTE CUANDO ESCUCHAS QUE EN VERDAD LAS PERSONAS NO SE TOMAN LOS MEDICAMENTOS

**Participante Femenina 1**: Que…mucha ayuda. Los medicamentos son caros y que quizás otros no pueden venir a tener tratamiento y no tienen los medicamentos y otros no lo valorizan.

**ENTREVISTADOR:** ¿Y QUÉ RAZONES DAN LAS PERSONAS, VERDAD, CUANDO TÚ LO ESCUCHAS, QUE RAZONES DAN LAS PERSONAS PARA DEJAR DE TOMARSE LOS MEDICAMENTOS?

**Participante Femenina 1**: Que a veces les cae mal o que no tienen ganas de bebérselas.

**ENTREVISTADOR:** ¿ESCUCHAS ALGO MÁS?

**Participante Femenina 1**: No

**ENTREVISTADOR:** ¿Y EN ALGÚN MOMENTO HAS PENSADO O HAZ DEJADO DE TOMARTE EL TRATAMIENTO PARA EL VIH?

**Participante Femenina 1**: Estuve un tiempito que no bebí medicamento pero era que no tenía transportación pero gracias a Dios aquí me están ayudando

**ENTREVISTADOR:** ¿CUÁNTO TIEMPO ESTUVISTE?

**Participante Femenina 1**: Como seis meses.

**ENTREVISTADOR:** ¿SEIS MESES? OKAY. NOTA AL CALCE, VERDAD, LO QUE NOSOTRAS HABLEMOS AQUÍ TAMPOCO…ESTA INFORMACIÓN NO LA VAN A TENER TAMPOCO NI LOS MANEJADORES DE CASO NI NADA O SEA QUE ES CONFIDENCIAL ACÁ…ENTRE TÚ Y YO. CUANDO PENSASTE DE DEJAR DE TOMAR MEDICAMENTOS ME DIJISTE QUE FUE POR TRANSPORTACIÓN, ALGUNA OTRA RAZÓN, ALGO MÁS QUE PASO, QUE DEJASTE TE TOMAR TUS MEDICAMENTOS.

**ENTREVISTADOR:** NO, PORQUE NO PODÍA VENIR A LAS CITAS. CUANTAS VECES TE HA PASADO ESO, QUE HAS DEJADO…DENTRO DEL PERIODO DE 15 AÑOS QUE HAS ESTADO CON LA CONDICIÓN.

**Participante Femenina 1:** Dos veces.

**ENTREVISTADOR:** ¿Y LA MÁS RECIENTE FUE CUANDO?

**Participante Femenina 1:** Hace como un año atrás

**ENTREVISTADOR:** COMO, VERDAD, COMO SABEMOS A MUCHAS PERSONAS CON VIH SE LES HACE DIFÍCIL TOMARSE LAS PASTILLAS TODOS LOS DÍAS, POR MUCHAS RAZONES QUE ESTÁN DENTRO Y FUERA DEL CONTROL DE LA PERSONA. AHORA YO TE VOY A HACER UNAS PREGUNTAS SOBRE LAS POSIBLES RAZONES QUE EN ALGÚN MOMENTO TÚ HAYAS DEJADO DE TOMAR EL MEDICAMENTO PARA EL VIH. AHORA QUE YO QUIERO QUE PIENSES EN TI, SOLAMENTE EN TI. SIN INCLUIR OTRAS PERSONAS O CIRCUNSTANCIAS.

**Participante Femenina 1:** Okay.

**ENTREVISTADOR:** SOLAMENTE TÚ. CONSIDERAS QUE ALGÚN…QUE TENGAS ALGÚN SENTIMIENTO ALGÚN PENSAMIENTO O CREENCIA PERSONAL QUE INFLUYA O QUE INFLUYÓ EN ESAS DOS OCASIONES EN QUE DEJASTE DE TOMARTE LOS MEDICAMENTOS

**Participante Femenina 1:** No.

**ENTREVISTADOR:** CUANDO DEJASTE DE TOMARTE EL MEDICAMENTO EN AQUEL MOMENTO, ¿QUÉ SENTÍAS? ¿CÓMO TÚ TE…QUÉ SENTÍAS?

**Participante Femenina 1:** Que iba a decaer.

**ENTREVISTADOR:** QUE IBAS A DECAER. DECAER CÓMO, POR EJEMPLO.

**Participante Femenina 1:** Que me podía morir, iba para atrás, para atrás, para atrás. Y cada rato me enfermaba.

**ENTREVISTADOR:** ¿TE ENFERMABAS A CADA RATO? ¿Y CÓMO TE HACE SENTIR ESO?

**Participante Femenina 1:** Sí. Yo estuve el año pasado a veces con bronquitis.

**ENTREVISTADOR:** AH, WOW.

**Participante Femenina 1:** Las defensas bien bajas. Bajé de peso como 30 libras.

**ENTREVISTADOR:** WOW. ESO FUE UN PERIODO DE TIEMPO BIEN CORTO.

**Participante Femenina 1:** Sí.

**ENTREVISTADOR:** Y, ¿CÓMO TÚ TE SENTÍAS? VIÉNDOTE QUE TÚ ESTABAS DECAYENDO.

**Participante Femenina 1:** Bien mal. Lo último que hice fue llamé a *¿?* le expliqué mi situación y el rapidito me ayudó.

**ENTREVISTADOR:** OKAY. Y EN ESE MOMENTO QUE PENSABAS POR EJEMPLO CUANDO TE PASÓ TODO ESO QUE NO TE LOS ESTABAS TOMANDO, QUE COSAS TU PENSABAS ADEMÁS DE ESO QUE TU ME ESTABAS DICIENDO.

**Participante Femenina 1:** Pues, si no cojo tratamiento y busco ayuda me voy a ir por ahí mismo.

ENTREVISTADOR: OKAY. Y QUE COSAS ESTABAN PASANDO EN TU VIDA EN ESE MOMENTO.

Participante Femenina 1: Problemas personales con mis hijos, con mi abuela que está en cama.

ENTREVISTADOR: ESTABAS CUIDÁNDOLO

Participante Femenina 1: Todavía.

ENTREVISTADOR: TODAVÍA?

Participante Femenina 1: Si.

ENTREVISTADOR: ALGUNA OTRAS COSAS QUE PASABAN EN TU VIDA PERSONAL. O SEA QUE TENÍAS PROBLEMAS CON TUS HIJOS Y TU ABUELITA? O SEA, CON TUS HIJOS Y TU ABUELITA QUE ESTABA EN CAMA.

Participante Femenina 1: Sí. Sí.

**ENTREVISTADOR:** AHORA VAMOS A HABLAR DE LAS PERSONAS CON LAS QUE TÚ TE RELACIONAS. O SEA, LAS PERSONAS…TU FAMILIA, POR EJEMPLO AMISTADES, TODAS ESAS PERSONAS CON LAS QUE TÚ TE RELACIONAS. ¿DE QUÉ MANERA, SI ALGUNA, LAS PERSONAS CON QUIEN TÚ TE RELACIONAS INFLUYERON EN QUE DEJARAS DE TOMARTE LOS MEDICAMENTOS? SI HUBO ALGO…LA MÁS MÍNIMA COSA QUE TÚ PUEDAS PENSAR…ALGO QUE INFLUYÓ PARA QUE DEJARAS DE TOMARTE LO MEDICAMENTOS.

Participante Femenina 1: Hasta ahora ninguna familia…el único problema es que no tenía transportación para venir.

**ENTREVISTADOR:** OKAY. ¿TÚ FAMILIA ENTONCES NO INFLUYÓ EN ESO?

**Participante Femenina 1:** No.

**ENTREVISTADOR:** Y AMISTADES, POR EJEMPLO. NADA DE ESO.

**Participante Femenina 1:** No.

**ENTREVISTADOR:** ¿LOS MÉDICOS QUE TE ATENDÍAN DE ALGUNA FORMA INFLUYERON? ALGO QUE TÚ VISTE, ALGUNA ACTITUD O ALGO.

**Participante Femenina 1**: No. Me ayudaron bastante.

**ENTREVISTADOR:** ¿EL PERSONAL DE ENFERMERÍA?

**Participante Femenina 1**: También.

**ENTREVISTADOR:** ¿EL PERSONAL QUE TE ATIENDE AQUÍ MISMO, EN EL LUGAR DONDE RECIBES TRATAMIENTO?

**Participante Femenina 1**: También.

**ENTREVISTADOR:** ¿DE ALGUNA MANERA INFLUYÓ EN QUE…EN LA MANERA EN QUE TE TRATABAN, O ALGO ASÍ?

**Participante Femenina 1**: No.

**ENTREVISTADOR:** NADA DE ESO. ¿O ALGUNA OTRA PERSONA QUE A LO MEJOR POR ALGUNA RAZÓN INFLUYERAN AUNQUE FUERA INDIRECTAMENTE?

**Participante Femenina 1**: No.

**ENTREVISTADOR:** OKAY. AHORA PIENSA EN EL AMBIENTE DONDE TÚ TE DESENVUELVES, ¿DE QUÉ MANERA, SI ALGUNA, ESE AMBIENTE INFLUYÓ EN LA DECISIÓN DE DEJAR DE TOMARTE LOS MEDICAMENTOS? DONDE TÚ ESTÁS, EL AMBIENTE DONDE TU ESTAS, DONDE TE DESENVUELVES…EN TU CASA, EN TU TRABAJO…

**Participante Femenina 1**: No.

**ENTREVISTADOR:** OKAY. LA COMUNIDAD EN DONDE VIVES, DONDE TÚ VIVES, SI DE ALGUNA MANERA INFLUYÓ EN ESO.

**Participante Femenina 1**: No, tampoco.

**ENTREVISTADOR:** OKAY. ME DIJISTE QUE TRABAJAS, ¿VERDAD?

**Participante Femenina 1**: Sí, barbera.

**ENTREVISTADOR:** ¿LOS MEDICAMENTOS TE LOS TOMAS EN EL TRABAJO? SI TE TOCAN DURANTE…

**Participante Femenina 1**: En casa, porque como yo soy barbera…

**ENTREVISTADOR:** AH, OKAY.

**Participante Femenina 1**: Trabajo en casa, a domicilio.

**ENTREVISTADOR:** AH, BUENO. TRABAJAS EN TU CASA.

**Participante Femenina 1**: Sí.

**ENTREVISTADOR:** O SEA, CUANDO TE LOS VAS A TOMAR, VAS Y TE LOS TOMAS.

**Participante Femenina 1**: Sí.

**ENTREVISTADOR:** Y EL AMBIENTE DONDE TU SOCIALIZAS, O COMO DICEN POR AHÍ UNO JANGEA, SOCIALIZA, ¿DE ALGUNA MANERA INFLUYÓ EN ESO, EN QUE NO TE LOS TOMARAS EN ALGÚN MOMENTO?

**Participante Femenina 1:** No.

**ENTREVISTADOR:** ME DIJISTE QUE EN LUGAR DONDE RECIBES SERVICIOS MÉDICOS NO. ALGÚN OTRO LUGAR QUE A LO MEJOR TÚ, DIGAMOS, TE TOCA TOMÁRTELA, A LO MEJOR NO QUE TE LO HAYAS DEJADO DE TOMAR POR MUCHO TIEMPO, TE TOCÓ TOMARTE UNA DOSIS Y ESTAS EN UN SITIO…

**Participante Femenina 1**: Que no puedo.

**ENTREVISTADOR:** …Y NO TE LO TOMAS.

**Participante Femenina 1**: Me ha pasado sí, en casa.

**ENTREVISTADOR:** ¿EN TU CASA?

**Participante Femenina 1**: Cuando tengo dos o tres clientes.

**ENTREVISTADOR:** Oh, okay.

**Participante Femenina 1**: Tengo las medicinas en la nevera. Tengo que esperar que se vayan y se me va la hora.

**ENTREVISTADOR:** AH, OKAY. Y A VECES…Y ESA DOSIS LA SALTAS O…

**Participante Femenina 1**: La hago más tarde.

**ENTREVISTADOR:** Te la bebes más tarde.

**Participante Femenina 1**: Mas tarde.

**ENTREVISTADOR:** AHORA PIENSA, AQUÍ, EN PUERTO RICO. LAS COSAS QUE ESTÁN PASANDO ACÁ EN PUERTO RICO. ¿DE QUÉ MANERA, SI ALGUNA, LAS COSAS QUE PASAN EN PUERTO RICO, INFLUYEN EN LA DECISIÓN EN DEJAR DE TOMARTE LOS MEDICAMENTOS?

**Participante Femenina 1**: No.

**ENTREVISTADOR:** EL GOBIERNO, LAS COSAS QUE PASAN EN EL GOBIERNO…

**Participante Femenina 1**: No.

**ENTREVISTADOR:** LA POLÍTICA…

**Participante Femenina 1**: No.

**ENTREVISTADOR:** LOS SERVICIOS DE SALUD DEL PAÍS…

**Participante Femenina 1**: ¿Los servicios de salud?

**ENTREVISTADOR:** Aja.

**Participante Femenina 1**: Bueno…

**ENTREVISTADOR:** Y LOS PLANES MÉDICOS, AHORA, POR EJEMPLO. Y LOS… ¿HAS TENIDO ALGÚN PROBLEMA CON LOS PLANES MÉDICOS QUE NO TE CUBRAN LOS MEDICAMENTOS?

**Participante Femenina 1**: Algunos medicamentos que no me cubre.

**ENTREVISTADOR:** Ajá. Y que ha pasado ahí cuando no te cubre el plan médico.

**Participante Femenina 1:** No voy al médico primario, vengo aquí y aquí me los proveen.

**ENTREVISTADOR:** TE LOS PROVEEN… ¿Y ESOS MEDICAMENTOS TIENEN QUE VER RELACIONADOS DIRECTAMENTE CON LA CONDICIÓN?

**Participante Femenina 1**: Sí.

**ENTREVISTADOR:** Y LOS MEDICAMENTOS CUANDO TE LOS PROVEEN ACÁ, SIEMPRE ESTÁN DISPONIBLES? O EN ALGÚN MOMENTO TIENES QUE ESPERAR PORQUE NO LOS TIENEN Y TIENES QUE ESPERAR HASTA QUE LLEGUEN.

**Participante Femenina 1**: Hasta ahora sí. Lo único era que me habían recetado hasta hace poco una pastilla para subir las defensas porque los medicamentos que me dan no me suben las defensas. Me bajan la carga viral pero las defensas no me suben. Y me hicieron un estudio y salí que necesitaba un medicamento más. Que tengo que buscarlo porque el mes pasado no lo había.

**ENTREVISTADOR:** AH, OKAY. O SEA, QUE ESTUVISTE EL MES PASADO SIN PODER TOMARTE ESE MEDICAMENTO.

**Participante Femenina 1:** Una pastilla, diaria.

**ENTREVISTADOR:** Y LAS DEFENSAS, ¿CÓMO ESTÁN?

**Participante Femenina 1:** Bajitas.

**ENTREVISTADOR:** BAJITAS TODAVÍA.

**Participante Femenina 1:** Sí.

**ENTREVISTADOR:** Y AHORA LO TIENES, EL MEDICAMENTO…LO TIENES.

**Participante Femenina 1:** Voy a buscarlo ya mismo.

**ENTREVISTADOR:** LO TIENES…O SEA QUE TE DIJERON QUE LO TIENEN DISPONIBLE.

**Participante Femenina 1:** Sí. Tenían el medicamento pero tenía que aprobarlo el plan.

**ENTREVISTADOR:** AH, OKAY, OKAY.

**Participante Femenina 1:** Tenía que aprobarlo el plan.

**ENTREVISTADOR:** ¿QUÉ PLAN TÚ TIENES? DEL GOBIERNO…

**Participante Femenina 1:** Tu Mano.

**ENTREVISTADOR:** Ah okay. ¿Plan privado?

**Participante Femenina 1:** Sí, de aquí.

**ENTREVISTADOR:** ¿SE TARDÓ UN MES EN APROBÁRTELO EL PLAN MÉDICO?

**Participante Femenina 1:** Viene ahora. Porque había llamado y todavía no estaba aprobado.

**ENTREVISTADOR:** Y LA SITUACIÓN ECONÓMICA DEL PAÍS, TÚ CREES QUE DE ALGUNA MANERA INFLUYE EN QUE POR EJEMPLO, Y CUANDO YO DIGO VERDAD QUE DEJE DE TOMARTE LOS MEDICAMENTOS, PUEDEN SER EJEMPLOS COMO ESE QUE NO LOS TIENEN, NO POR QUE NECESARIAMENTE…QUE SON RAZONES QUE PUEDEN ESTAR FUERA…DE TU CONTROL O FUERA DE TU CONTROL. QUE NO TIENES EL MEDICAMENTO, ESTÁ FUERA DE TU CONTROL, PERO NO TE LOS HAS PODIDO TOMAR.

**Participante Femenina 1:** No me lo he podido tomar.

**ENTREVISTADOR:** DE ALGUNA…POR EJEMPLO LA SITUACIÓN ECONÓMICA DEL PAÍS O ALGUNA OTRA SITUACIÓN QUE ESTE FUERA DE TU CONTROL QUE NO TE LO HAYAS PODIDO TOMAR EN ALGÚN MOMENTO. SI TE ACUERDAS DE ALGUNA OTRA…ALGÚN OTRO EJEMPLO.

**Participante Femenina 1:** No.

**ENTREVISTADOR:** LA TRANSPORTACIÓN, ME DIJISTE, ¿VERDAD?

**Participante Femenina 1:** Sí.

**ENTREVISTADOR:** CUÉNTAME UN POQUITO DE ESO, ¿QUÉ PASO CON LA TRANSPORTACIÓN? CÓMO FUE QUE SUCEDIÓ TODO ESTO.

**Participante Femenina 1:** Porque mi hermano me traía de allá para acá. Y después se fue para la Florida. Después me traía mi prima, ella empezó a trabajar y no tenía quien me trajera.

**ENTREVISTADOR:** AH OK.

**Participante Femenina 1:** Pero yo no tenía conocimiento de que había transportación aquí, ayudaban.

**ENTREVISTADOR:** AH, OK.

**Participante Femenina 1:** Nunca pregunté. Cuando llamé a ¿? me dijo que había transportación y porqué yo no había explicado. No tenía conocimiento.

**ENTREVISTADOR:** ALGUNA RAZÓN POR LA QUE NO PREGUNTASTE, O SEA, NO TE ATREVISTE…

**Participante Femenina 1:** No me atreví.

**ENTREVISTADOR:** AH, JA JA.

**Participante Femenina 1:** No me atreví.

**ENTREVISTADOR:** ¿QUÉ PENSABAS, QUE A LO MEJOR TE IBAN A…TE IBAN A…?

**Participante Femenina 1:** Sí. No me atreví. Yo soy de las que vengo a cita y pues me voy. No me atrevo preguntar si hay fondo, si hay…no. Nada de eso.

**ENTREVISTADOR:** Y SI PREGUNTAS, ¿QUÉ TÚ CREES QUE VAN A PENSAR DE TI?

**Participante Femenina 1:** Ay, no. No me atrevería (se ríe).

**ENTREVISTADOR:** (SE RÍE) NO, PORQUE A VECES UNO…UNO NO SE ATREVE, UNO DICE ‘AY VAN A PENSAR QUE YO SOY ESTO QUE YO HAGO AQUELLO, LO OTRO’. (SE RÍE)

**Participante Femenina 1:** (se ríe) Eso no.

**ENTREVISTADOR:** ESO PASO. ALGUNA SITUACIÓN QUE ESTÉ PASANDO EN EL PAÍS, A LO MEJOR ESO TÚ CREES QUE INDIRECTAMENTE INFLUYE, POR EJEMPLO, ALGO QUE ESTÉ PASANDO EN EL PAÍS QUE ESTE FUERA DE TU CONTROL Y TÚ CREES QUE POR ESA RAZÓN TÚ NO TIENES LOS MEDICAMENTOS…

**Participante Femenina 1:** No, para mí el plan de la Reforma, nada más. Que a veces tienen series cosas que perjudican a los pacientes, en aprobar los medicamentos y eso.

**ENTREVISTADOR:** LA APROBACIÓN DE LOS PLANES MÉDICOS. AHORA PIENSA EN LAS COSAS COMO LAS COSTUMBRES, CREENCIAS DE NUESTRA SOCIEDAD…YA VAS MÁS COMO LA SOCIEDAD, LA CULTURA Y TODA ESAS COSAS. ¿DE QUÉ MANERA, SI ALGUNA, LAS CREENCIAS EN LA SOCIEDAD, LA COSTUMBRES, INFLUYEN EN LA DECISIÓN DE DEJAR DE TOMAR LOS MEDICAMENTOS PARA EL VIH?

**Participante Femenina 1:** De qué manera…

**ENTREVISTADOR:** UJUM. POR EJEMPLO, TE HAS SENTIDO DISCRIMINADA ALGUNA VEZ POR TENER LA CONDICIÓN.

**Participante Femenina 1:** No.

**ENTREVISTADOR:** NUNCA TE HAS SENTIDO ASÍ. ALGUNA CREENCIA RELIGIOSA, A LO MEJOR ESO HAYA INFLUIDO.

**Participante Femenina 1:** Soy católica.

**ENTREVISTADOR:** ¿Sí, no? Dime…

**PARTICIPANTE FEMENINA 1:** (SE RÍE). NO.

**ENTREVISTADOR:** NO TE…OKAY. LA SITUACIÓN SOCIAL, POR EJEMPLO, ECONÓMICA, SOCIAL, CREES QUE HA INFLUIDO EN ALGO.

**Participante Femenina 1:** No.

**ENTREVISTADOR:** ALGUNA OTRA COSA DE LA SOCIEDAD, CÓMO LA GENTE PIENSA, Y…POR EJEMPLO…PERSONAS…OTRAS PERSONAS… ¿CONOCES A OTRAS PERSONAS QUE VIVEN CON LA CONDICIÓN TAMBIÉN?

**Participante Femenina 1:** A los que vienen aquí.

**ENTREVISTADOR:** Y DE LAS PERSONAS QUE VIENEN ACÁ, ¿QUÉ HAS ESCUCHADO? ¿HAS ESCUCHADO DE PERSONAS QUE HAN DEJADO DE TOMARSE LOS MEDICAMENTOS?

**Participante Femenina 1:** Sí.

**ENTREVISTADOR:** ¿Y QUÉ TE DICEN ESAS PERSONAS?

**Participante Femenina 1:** Que caen en depresión, que no quieren beberse las pastillas. Que a veces le caen mal…así.

**ENTREVISTADOR:** TODAS ESAS COSAS. Y CON RELACIÓN A LO DE, POR EJEMPLO, LOS PLANES MÉDICOS, LA SITUACIÓN ECONÓMICA, ¿HAS ESCUCHADO ALGO?

**Participante Femenina 1:** Sí, eso lo de los medicamentos.

**ENTREVISTADOR:** SÍ, QUE LE HAN PASADO LO MISMO.

**Participante Femenina 1:** Sí. Lo mismo.

**ENTREVISTADOR:** AHORA YO QUIERO QUE PIENSES EN LOS TIEMPOS QUE ESTAMOS VIVIENDO. ESTOS TIEMPOS DE AHORA. ¿DE QUÉ MANERA, SI ALGUNA, ESTOS TIEMPOS QUE VIVIMOS INFLUYEN EN LA DECISIÓN DE DEJAR DE TOMARSE LOS MEDICAMENTOS? LOS TIEMPOS…LA ERA…LA ÉPOCA QUE ESTAMOS VIVIENDO AHORA. HAY ALGUNA…HA INFLUENCIADO EN…

**Participante Femenina 1:** No.

**ENTREVISTADOR:** Y PERSONAS QUE TÚ HAS ESCUCHADO QUE VIENES AQUÍ, Y ESTÁN EN LA SALA DE ESPERA Y TÚ LAS ESCUCHAS…ALGO QUE TU HAYAS ESCUCHADO DE LOS TIEMPOS DE AHORA. COMO QUE…HAYAN INFLUENCIADO EN ESO.

**Participante Femenina 1:** Hasta ahora no. Lo que dicen es que los medicamentos le han cambiao. Que antes eran más medicamentos que uno tenía que beberse, que eran tres pastillas al día, ahora es una.

**ENTREVISTADOR:** ¿Y eso es bueno o es malo?

**Participante Femenina 1:** En parte es bueno.

**ENTREVISTADOR:** OKAY. QUE ESO HA MEJORADO ENTONCES. ENTONCES CUANDO EN EL PASADO DEJASTE DE TOMARTE LOS MEDICAMENTOS DEL VIH…CUANDO EN EL PASADO DEJASTE DE TOMARTE LOS MEDICAMENTOS DEL VIH, ¿QUÉ TE AYUDÓ…? VOY A PONER LA GRABADORA DE ACÁ… CUANDO EN EL PASADO DEJASTE DE TOMARTE LOS MEDICAMENTOS DEL VIH, ¿QUÉ TE AYUDÓ A VOLVER A TOMÁRTELOS?

**Participante Femenina 1:** ¿Qué me ayudó?

**ENTREVISTADOR:** AJÁ.

**Participante Femenina 1:** Los médicos aquí.

**ENTREVISTADOR:** ¿LOS MÉDICOS?

**Participante Femenina 1:** Ujum.

**ENTREVISTADOR:** LOS MÉDICOS. ¿QUÉ HICIERON…CÓMO…QUÉ HICIERON…?

**Participante Femenina 1:** Me motivaron…me motivaron…me dieron los medicamentos…me orientaron.

**ENTREVISTADOR:** UJUM. OKAY. ¿CÓMO TE ORIENTARON? ¿QUÉ TE DIJERON? ¿QUÉ COSAS TE DIJERON?

**Participante Femenina 1:** Que al yo dejar el tratamiento como yo estaba de salud que había deteriorado había ido para atrás. Me tuvieron que dar muchos medicamentos, antibióticos…para la bronquitis.

**ENTREVISTADOR:** ¿ESTUVISTE HOSPITALIZADA O ALGO ASÍ?

**Participante Femenina 1:** No. Aquí cogía terapias…me dieron pastillas. Y venia cada dos semanas a darme terapia para los pulmones.

**ENTREVISTADOR:** ADEMÁS DE LOS MÉDICOS, ¿ALGO MÁS QUE TE HAYA AYUDADO A TOMAR LA DECISIÓN DE VOLVER AL TRATAMIENTO?

**Participante Femenina 1:** El trabajador social.

**ENTREVISTADOR:** Alguien más en tu familia…. ¿Tienes apoyo de tu familia? ¿Sí? ¿Estás bien?

**PARTICIPANTE FEMENINA 1:** SÍ.

**ENTREVISTADOR:** ¿QUÉ TÚ LE DIRÍAS A UNA PERSONA QUE ESTÁ PENSANDO EN DEJAR DE TOMARSE LOS MEDICAMENTOS EN ESTOS MOMENTOS?

**Participante Femenina 1:** Que no los dejes.

**ENTREVISTADOR:** OKAY. ¿QUÉ LE RECOMENDARÍAS? POR EJEMPLO, LAS PERSONAS QUE HAS TENIDO LA OPORTUNIDAD DE HABLAR DE ESO, QUE TÚ HAS ESCUCHADO, ¿QUÉ TÚ LE RECOMIENDAS A ESAS PERSONAS?

**Participante Femenina 1:** ¿Que le recomendaría?

**ENTREVISTADOR:** SÍ.

**Participante Femenina 1:** Que sigan el tratamiento…esta condición no es fácil…

**ENTREVISTADOR:** ¿ALGO QUE TE PUEDA AYUDAR?

**Participante Femenina 1:** Nada.

**ENTREVISTADOR:** ¿ALGO MÁS QUE LE RECOMENDARÍAS A LAS PERSONAS?

**Participante Femenina 1:** Que si no tienen transportación, que llamen a la clínica y pregunten.

**ENTREVISTADOR:** AHORA, CUANDO ESCUCHAS LA FRASE “ADHERENCIA O TRATAMIENTO A LAS RECOMENDACIONES MÉDICAS”, ¿CON QUÉ TÚ LAS RELACIONAS ESO? A LAS RECOMENDACIONES MÉDICAS.

**Participante Femenina 1:** ¿A las recomendaciones?

**ENTREVISTADOR:** AJÁ. QUE TÚ CREES QUE ESO? QUE TU ENTIENDES POR RECOMENDACIONES MÉDICAS?

**Participante Femenina 1:** Para mejorar una calidad de vida…mejor tratamiento y buscar a que uno se recupere.

**ENTREVISTADOR:** ADEMÁS DE TOMARTE EL MEDICAMENTO PARA LA CONDICIÓN, QUE OTRAS COSAS TE RECOMIENDA EL MÉDICO. EL TE DICE QUE TE TOMES EL MEDICAMENTO, PERO QUE OTRAS COSAS ÉL TE DICE QUE HAGAS

**Participante Femenina 1:** Que la beba al día, las horas que me tocan, los suplementos, los antibióticos.

**ENTREVISTADOR:** TE MANDA A HACER PRUEBAS, POR EJEMPLO…

**Participante Femenina 1:** Sí.

**ENTREVISTADOR:** …OTRAS PRUEBAS, O QUE VAYAS A CHEQUEARTE A OTROS MÉDICOS. EL HACE…ÉL TE DICE ESO.

**Participante Femenina 1:** Me hacen pruebas pero aquí hay ginecólogos, hay psicólogos…

**ENTREVISTADOR:** ¿Y TE HAN REFERIDO A ALGUNO DE ESOS OTROS ESPECIALISTAS?

**Participante Femenina 1:** Sí. Aquí siempre he ido. Y a veces viene una guagua aquí, vienen dentistas. Todo eso hay aquí.

**ENTREVISTADOR:** OKAY. Y ALGUNAS DE ESAS…A VECES CUANDO TE HACEN ESOS REFERIDOS, SE TE…REFERIDOS QUE VAYAS A ALGÚN ESPECIALISTA A HACERTE ALGUNOS ANÁLISIS, O TE VAYAS A HACER OTRAS PRUEBAS COMO EL PAP O ANÁLISIS VERDAD. ¿HAY ALGUNO DE ESO QUE SE TE HACE DIFÍCIL SEGUIR, O SEA, HACÉRTELO, POR ALGUNA RAZÓN.=?

**Participante Femenina 1:** Sí.

**ENTREVISTADOR:** SE TE HACE DIFÍCIL, ¿SÍ? ¿CUÁL DE ELLOS?

**Participante Femenina 1:** La prueba del PAP.

**ENTREVISTADOR:** LA DEL PAP? Y ENTONCES, PORQUE SE TE HACE DIFÍCIL? QUE DE ESO…

**Participante Femenina 1:** Porque me da vergüenza (se ríe).

**ENTREVISTADOR:** (SE RÍE) ESO PASA. SÍ. Y TE LA HAS HECHO, LA PRUEBA?

**Participante Femenina 1:** No. Llevo años…

**ENTREVISTADOR:** SÍ, ¿MÁS DE 5 AÑOS?

**Participante Femenina 1:** Y tenía cita…

**ENTREVISTADOR:** SÍ, MÁS DE 5 AÑOS. ME DICES... ¿SÍ, NO?

**Participante Femenina 1:** Sí (se ríe).

**ENTREVISTADOR:** ALGUNA OTRA PRUEBA, QUE SE YO, DEL AZÚCAR, COLESTEROL…

**Participante Femenina 1:** Todas esas me las hacen.

**ENTREVISTADOR:** ¿TE LO HACEN?

**Participante Femenina 1:** Sí. Cada tres meses.

**ENTREVISTADOR:** ¿TE LAS HACEN AQUÍ MISMO…AQUÍ MISMO TE LAS HACEN?

**Participante Femenina 1:** Aquí hacen los exámenes de carga viral…y eso. Pero yo me los hago allá en Salinas.

**ENTREVISTADOR:** ¿HAY ALGUNA OTRA CONDICIÓN QUE TU HAYAS DESARROLLADO, ADEMÁS DEL VIH?

**Participante Femenina 1:** Asma es lo que tengo. Padezco mucho de asma.

**ENTREVISTADOR:** ¿ASMA?

**Participante Femenina 1:** Sí.

**ENTREVISTADOR:** Y EL ASMA, ¿TE LO ESTAS TRATANDO?

**Participante Femenina 1:** Me dan aquí terapia.

**ENTREVISTADOR:** LAS CITAS…CUANDO A VECES… ¿HAS FALTADO A CITAS MÉDICAS? SE TE HA HECHO DIFÍCIL VENIR A LAS CITAS…

**Participante Femenina 1:** Sí, me tocaba el día 11. Recuerda que yo tengo mi abuela en cama. Llamé ayer a ¿? y me…

**ENTREVISTADOR:** ADEMÁS DE ESA DIFICULTAD, VERDAD, DE QUE TÚ CUIDAS A TU ABUELITA, ¿ALGUNA OTRA DIFICULTAD PARA VENIR A LAS CITAS MÉDICAS…OTRAS COSAS QUE PASAN QUE TÚ DICES ‘SIEMPRE ME PASA ESTO QUE NO PUEDO…QUE TENGO QUE FALTAR A LA CITA’?

**Participante Femenina 1:** No.

**ENTREVISTADOR:** OKAY. HAY ALGO QUE TÚ QUIERAS AÑADIR SOBRE LO QUE ESTAMOS…SOBRE EL TEMA QUE HEMOS ESTADO HABLANDO QUE A LO MEJOR YO NO…QUE TÚ QUIERAS…QUE TÚ QUIERAS DECIR Y YO NO TE HE PREGUNTADO ALGO

**Participante Femenina 1:** No.

**ENTREVISTADOR:** OKAY. PUES ENTONCES TERMINAS RÁPIDO. TERMINAMOS YA LA ENTREVISTA. TUVE QUE GRABAR EN ESTA PORQUE SE ME LLENO LA MEMORIA DE LA GRABADORA. YO ESPERO QUE ESTE HAYA GRABADO…

**------------ FINAL DE LA ENTREVISTA ------------**

Entrevista #2

Participante masculino (IDU en rehabilitación)

**ENTREVISTADOR: Entrevista individual número dos. Ya vamos a comenzar el proceso de la entrevista. El participante ya firmó consentimiento. Esta de acuerdo en participar del estudio y comenzamos entonces la entrevista. Este… ¿desde cuándo te estas tomando los medicamentos para el VIH?**

Participante masculino 2: Desde el 1995.

**ENTREVISTADOR: 95. Ya hace…bastante tiempo. Estamos hablando de más de 20 años. ¿Y empezaste a tomar los medicamentos cuando te dieron el diagnostico o paso un tiempo?**

Participante masculino 2: No, pasó un tiempo. El diagnóstico me lo dieron en el 1991.

**ENTREVISTADOR: Ah ok.**

Participante masculino 2: Me dieron unos medicamentos que cuando fue que salió la AZT. Pues no me estaban cayendo muy bien y entonces eso…yo estaba en Estados Unidos. Entonces regresé aquí a Puerto Rico entonces fue cuando pue’ mi hermano me llevó a una clínica fue de la hermana de ella y allí me dieron otros medicamentos entontes fue cuando empecé a coger esos medicamentos.

**ENTREVISTADOR: Okay. Así que estuviste un tiempo, desde el 91 al 95 sin medicamentos porque te hacían…**

Participante masculino 2: No me caían bien.

**ENTREVISTADOR: …no te caían bien. Y tú los dejaste por tu propia voluntad…**

Participante masculino 2: Eso es así.

**ENTREVISTADOR: Te voy a poner la grabadora acá un poquito más…por si acaso. Un poquito más al medio… Entonces en el lugar donde te trataban, ¿tenían los medicamentos…ellos tenían los medicamentos disponibles o pasaba que a veces no los tenían en el momento, tenían que…?**

Participante masculino 2: Al principio cuando yo…bueno, en ese momento me dieron la AZT. De eso desconozco como yo el deje rápido no tengo conocimiento de eso.

**ENTREVISTADOR: Y después de eso, cuando empezaste a tomar los medicamentos, ¿siempre lo tenían?**

Participante masculino 2: Sí, siempre los tenían aquí.

**ENTREVISTADOR: Y la información…tu lees la información que dice de los efectos secundarios de los medicamentos, cuando…**

Participante masculino 2: Eso es así.

**ENTREVISTADOR: ¿Los lees o te los explican?**

Participante masculino 2: Bueno, me lo explican y…sí, me lo explican.

**ENTREVISTADOR: Okay. Y conoces cuales son los efectos secundarios…**

Participante masculino 2: Sí, uno de ellos que yo utilizo fue la Sustiva que es le da como sueño a uno. Yo se lo he dicho a ellos…una vez quería cambiarlos, tu sabe, pero pues…como me adaptado a ellos ya pues no…pue’ he seguio’ con ella. Sigo bebiéndomela.

**ENTREVISTADOR: Okay.**

Participante masculino 2: Pero esta ultima vez que deje de beberme los medicamentos, querían hacerme otro examen más… ¿un genotipo es?

**ENTREVISTADOR: Sí.**

Participante masculino 2: Pero era porque tenía la hemoglobina baja…las plaquetas…entonces…que eso fue antes de ingresar ahora. Pero como ya me subieron un poco no…no hubo necesidad de hacerlo.

**ENTREVISTADOR: ¿Te han tenido que cambiar mucho los medicamentos?**

Participante masculino 2: No, no. Este…

**ENTREVISTADOR: ¿Cuántas veces te han cambiado?**

Participante masculino 2: Ninguna.

**ENTREVISTADOR: ¿Ninguna? Volviste otra vez al tratamiento…siempre ha sido el mismo.**

Participante masculino 2: Sí, sí.

**ENTREVISTADOR: ¿Qué piensas de los medicamentos para tratar el VIH? ¿Qué piensas de ellos?**

Participante masculino 2: Yo, para mi funcionan…funcionan. Siempre y cuando uno lo utilice como tienen que ser. Pero como hay a veces que uno pue’, por ignorancia los deja o deja de bebérselo un dia o dos y cuando viene a ver deja de bebérselos por completo y pue’ , como la situación mía yo tuve la adicción pue’ lo deje de usar un tiempo.

**ENTREVISTADOR: ¿Y que tu esperas…cuál es tu expectativa…que tu esperas del tratamiento para el VIH?**

Participante masculino 2: ¿Que yo espero? Bueno, esperar como tal, no espero nada. Solamente es mantenerme con ellos porque ya como quien dice para mí ya no va haber una cura. Pero por lo menos puedo vivir con ellos. Me mantienen y pue’ tengo una mejor salud pa’ como estaba antes. Pue’ si apareciera una cura para otras persona, porque ya conmigo ya yo entiendo que pa mi es tarde pa eso, pero por lo menos puedo vivir con ellos y estoy…me siento bien.

**ENTREVISTADOR: Y cuando escuchas comentarios de otras personas que han dejado de tomarse los medicamentos, ¿qué tú piensas de ellos?**

Participante masculino 2: Bueno yo una vez, este…una persona que yo pue’ si vamo’ a ver nos criamos juntos en el mismo pueblo donde yo vivo. Dejó de beberse los medicamentos y en tiempo pue’ tu sabe ya era tarde…cuando empezó a bebérselos otra vez ya era tarde.

**ENTREVISTADOR: Ah ok.**

Participante masculino 2: Porque estuvo mucho tiempo sin bebérselos y parece que ya no era el mismo sistema…se le fue pa tras pa tras pa tras. Se descuidó.

**ENTREVISTADOR: ¿Y qué piensas de eso que hizo…o sea que piensas cuando las personas hacen eso? ¿Qué te viene a la mente?**

Participante masculino 2: Yo no sé si fue que lo hizo por suicidarse el mismo o ya estaba cansao’ de beberse los medicamentos. Pue’, son muchas cosas lo que le viene a la mente a uno. Por lo menos a mí, en estos momentos lo dejaba por la adicción a veces y no por mucho tiempo, porque a mi me preocupa bebérmelos.

**ENTREVISTADOR: Okay.**

Participante masculino 2: Pero pue’, por circunstancias de la vida uno hace ignorancias, pue’ cosas ignorantes.

**ENTREVISTADOR: ¿Y qué razones, verdad, esas otras personas que tú has escuchado? Que dejan los medicamentos, ¿qué razones dan ellos por dejar de tomarse los medicamentos?**

Participante masculino 2: Bueno, algunas me han dicho que se sienten bien, que no necesitan bebérselos. Pero yo entiendo que no es así. Porque ya a la vez que uno tiene un medicamento y está estable con ese medicamento ya tiene…es como decir es un hábito que tiene que tener a diario. Al dejar de bebérselo no funciona igual.

**ENTREVISTADOR: Sí.**

Participante masculino 2: Pero, para muchas personas se creen que porque se bebieron el medicamento, están bien y pue’ y lo dejan de beber van a estar…se les descontrola el sistema.

**ENTREVISTADOR: ¿Y qué otras razones escuchas que dicen?**

Participante masculino 2: Que están cansao’ de bébeselos…como yo que han dejado de bebérselo por las sustancias.

**ENTREVISTADOR: ¿Algo más? ¿Alguna otras razones que escuchas así de la gente que te lo dicen, o escuchas verdad que los hablan?**

Participante masculino 2: Que se han cansao’…de que están cansaos de beberse los medicamentos. Eso es lo único que yo he oído.

**ENTREVISTADOR: Y cuando uno se cansa…cansarse es… ¿cómo uno se cansa?**

Participante masculino 2: Pues...este…de estar bebiendo…mayormente…bueno hay personas que se cansan por estar bebiéndoselo todos los días. Hay unas que ven tanta medicación, como que tanta pastilla para bebérsela, tu sabe, que…antes cuando empezó esto de la condición del VIH eran 7, 8, 9 pastillas que uno tenía que bebérsela. Por lo menos ahora es 3. Hay medicamentos que te bebes una pastilla na’ más al día y con eso está. Pero hay gente que se cansa por eso.

**ENTREVISTADOR: Aun cuando sea una pastilla, ¿se cansan?**

Participante masculino 2: Eso es así.

**ENTREVISTADOR: Este… ¿Cuándo dejaste de tomarte el medicamento…la razón que me dijiste que había sido la…?**

Participante masculino 2: Sí, porque estaba…estaba en la adicción y pue’ me olvidaba de bebérmelo y hubo un tiempo que ya ni me acordaba de ella.

**ENTREVISTADOR: ¿Había alguna razón por la que dejabas de tomártela?**

Participante masculino 2: No.

**ENTREVISTADOR: ¿Cuántas veces has dejado de tomártela?**

Participante masculino 2: Eh…como dos veces.

**ENTREVISTADOR: Dos veces…**

Participante masculino 2: Y ha sido por lo mismo

**ENTREVISTADOR: ¿Por lo mismo?**

Participante masculino 2: Yeah.

**ENTREVISTADOR: Y este…y además de… a veces a uno se le olvida dosis también.**

Participante masculino 2: Sí, eso se me ha olvidado.

**ENTREVISTADOR: ¿Te ha pasado?**

Participante masculino 2: Sí, dos y tres días corrido.

**ENTREVISTADOR: Ah okay…**

Participante masculino 2: Pero me acuerdo otra vez y empiezo de nuevo.

**ENTREVISTADOR: Y cuando se te olvidan las dosis que… ¿te pasa con frecuencia eso?**

Participante masculino 2: Muchas veces pue’ cuando uno está en las sustancias y yo siempre me he bebido los medicamentos, pero hay a veces que cuando uno se alimenta el medicamento le hace daño a uno en el estómago…molesta en el estómago. Entonces muchas veces por eso es que no me los bebía.

**ENTREVISTADOR: Ah, ¿por qué no comías?**

Participante masculino 2: Eso es así.

**ENTREVISTADOR: ¿Cuántos días estabas sin comer? ¿Más de un día?**

Participante masculino 2: No, un día a…más tardar un dia.

**ENTREVISTADOR: Este…te voy a hacer ahora otras preguntas. Entonces preguntas que te voy a hacer me las vas a contestar pensando en esas veces que dejaste de tomarte los medicamentos mucho tiempo pero también pensando también en las veces que has dejado de tomar las dosis. Ya sea una o dos dosis. Pero, que tengas en mente esas dos situaciones. A veces…verdad, a muchas personas con VIH se les hace difícil tomarse las pastillas todos los días, por diversas razones que están dentro y fuera del control de uno. Puede ser que a veces tú tengas el control y a veces uno no tiene el control. Te voy a hacer unas preguntas sobre las posibles razones para que en algún momento tú hayas dejado de tomarte los medicamentos o las dosis. Verdad, que se te hayan olvidado. Ahora piensa en ti, solamente en ti, sin incluir otras personas sin incluir otras circunstancias. ¿Consideras que tengas algún sentimiento, pensamiento o creencia personal que haya influido en dejar de tomarte los medicamentos?**

Participante masculino 2: No.

**ENTREVISTADOR: Las veces que dejaste de tomarte los medicamentos o las dosis, ¿qué sentías? ¿Qué estabas…que sentimientos tenías en ese momento?**

Participante masculino 2: Sentimiento, pue’ que yo creo que ninguno, solamente que pue’ muchas veces dejaba de bebérmelas como le dije por…por la adicción a drogas otra que me olvidaba de bebérmela a diario. Otras veces era que cuando me daban las recetas para ir a sacarla no tenía el dinero para pagar el deducible, para sacarlas a tiempo. Tu sabe, porque se supone que hay unos fondos que le dan a uno para uno…pero que en esos momentos no había ninguno, no tenía donde sacarla, tenía que esperar par de días a lo que conseguía el dinero. Porque primero me iba para un lao antes de hacer lo que tenía que hacer para mí y mi salud. Pue’, esa son las cosas que me pasaban.

**ENTREVISTADOR: Y ahora, ¿te cubre todo el tratamiento o tienes que pagarlo?**

Participante masculino 2: Yo estuve pagando el deducible, pero que ahora mismo, pue’ me estuvo, no raro, porque yo sé que es así, pero este cuando estoy yendo a la clínica ellos me están pagando…según ellos me dicen que están y que los fondos ahí. Y me están pagando los deducibles y si hay otros medicamentos que no me los cubre la tarjeta pue’ ellos también me los pagan. Pero antes de eso, tuve…yo tenía que pagar los deducibles.

**ENTREVISTADOR: ¿Cuánto tiempo atrás hace de eso?**

Participante masculino 2: Eso…le estoy hablando por lo menos este…seis o siete meses atrás.

**ENTREVISTADOR: Ah, o sea que hace poquito esta… ¿Y te quedabas mucho tiempo sin tomártelos por que no la tenías?**

Participante masculino 2: Bueno, la última vez que estuve…pero esta vez que estuve fue por eso…una de esas razones y otra por que estaba también en la adiciones. Estuve casi como tres meses sin bebérmelas.

**ENTREVISTADOR: ¿La de los tres meses fue porque no tenías el dinero?**

Participante masculino 2: Aja, y después que los saque también pues me olvidaba de bebérmelo y cuando eso era…porque…uno necesitaba tener la sustancia para uno tener...estar tranquilo…

**ENTREVISTADOR: ¿Había alguna interacción entre la sustancia y el medicamento? Si te tomabas el medicamento sentías que te hacia alguna…**

Participante masculino 2: El estómago…me molestaba demaciao’.

**ENTREVISTADOR: Cuando usabas la sustancia, ¿también había algo ahí que interactuaba, como que…si te tomabas el medicamento y la sustancia a la vez, que podía pasar?**

Participante masculino 2: No sé, yo me…cogía la sustancia, después comía algo, entonces después era que me bebía los medicamentos.

**ENTREVISTADOR: Y en esos momentos, verdad, como te dije, ya sea cuando dejaste de tomártelos completo o cuando se te olvidaba dosis, que te venía a la mente, que te pasaba por la mente, que pensabas?**

Participante masculino 2: Pues…aquí se me va a descontrolar otra vez el sistema porque ya…como porque ya yo tenía…ya yo estaba con la…como se dice eso…estaba no detectable. Entonces pensando otra vez, que iba otra vez, porque eso, no es que no lo tenga, pero siempre me puede volver a aparecer, entonces las plaquetas me bajan, las defensas, ‘muglobina. Todo eso.

**ENTREVISTADOR: ¿Y qué sentías de eso que pensabas?**

Participante masculino 2: Pues yo pensaba que se me iba a caer el mundo…

**ENTREVISTADOR: Y cómo te sentías cuando…**

Participante masculino 2: Sentido de culpa por mí mismo, porque yo era…no lo quería hacer porque yo quisiera hacerlo, sino que, pues la razones que le explique. Pero yo sé que el medicamento es una forma para mantener a uno bien. Pue’ hasta la fecha yo siempre me lo he bebido diario. Esas son las únicas veces que he estao’ sin bebérmela.

**ENTREVISTADOR: Créeme, que esta información que me estás dando ayuda a entender también a otras personas que probablemente están pasando por lo que tu estas pasando. Ahora mismo probablemente hay mucha gente pasando por lo que tú pasaste en algún momento. Y esto que tú estás diciendo, nos ayuda a nosotros a entender para poder ayudar…como podemos ayudar. Este…y entonces, cosas que estaban pasando en tu vida en esos momentos. Me dijiste de la adicción. ¿Algunas otras situaciones que estaba pasando en tu vida en esos momentos que dejaste de tomarlo? Problemas, situaciones…**

Participante masculino 2: Problemas siempre ha habido…discusiones con mis hermanos…pero eso es cosa de familia…cosas que siempre han pasado, pero no era cosa que me diera el deso de dejarme de beberme los medicamentos ni nada de eso. Bueno, de hecho, mi mama no sabe de la condición mía. Solamente dos hermanos míos que lo saben.

**ENTREVISTADOR: Algo más de acerca de ti, verdad, de…a lo mejor algo más…cosas tuyas personales que hayan interferido.**

Participante masculino 2: Na’, no creo.

**ENTREVISTADOR: Ahora vamos a hablar de las personas con las que tú te relacionas. Este… ¿de qué manera, si alguna, las personas con las que tú te relacionas influyeron en dejarte de tomar los medicamentos? Alguna influencia que hayan tenido ellos.**

Participante masculino 2: Pues, yo estuve, como le dije, yo estuve una vez en la clínica donde me atiendo, y hay una señora que da unas charlas que es de…del área de San Juan. Ella no…no sé qué fue lo que ella me dijo que era parte de una agencia ahí. Tenían una muchacha que siempre estaba en unas reuniones que ella iba. Entonces parece que le dijo a ella que se había dejado de beber los medicamentos porque ya estaba cansada de bebérselos. Entonces, no paso mucho tiempo que la muchacha pue’ falleció. Entonces ella, siempre estaba dando ese consejo allí, que no dejáramos de beber los medicamentos porque pue’, en un momento dado eso es lo que nos iba a pasar.

**ENTREVISTADOR: Y eso que ella decía, digamos de alguna manera, como que te toco para dejar de tomártelo o para tomártelos.**

Participante masculino 2: No, para seguir bebiéndomelos. Sí.

**ENTREVISTADOR: Y algo que te haya tocado de alguien, a veces, que se yo, a veces con sus comentarios, cosas que te toquen y tú digas ‘eso me toco’ y la cogiste con el tratamiento. Que diga ‘eso que está haciendo esta persona lo que me está diciendo es como que me llegó, me tocó y te puso de una manera que tú dices “no más, voy a dejar”…**

Participante masculino 2: No.

**ENTREVISTADOR: ¿Nada de eso? Familia, familiares…**

Participante masculino 2: Tampoco.

**ENTREVISTADOR: Amistades…**

Participante masculino 2: Como casi nadie sabe de mi condición…

**ENTREVISTADOR: Okay. Los médicos que te atienden, si alguna situación de algún médico que tú digas…”como que esto que me dijo me hace pensar que yo voy a dejar de tomar los medicamentos”**

Participante masculino 2: No.

**ENTREVISTADOR: O el personal de enfermería, personal que te atiende, alguna otra persona…**

Participante masculino 2: Esa gente, siempre lo que he oído es pue’…de los medicamentos, que siempre lo tengamos al dia, que no los bebamos, que eso es lo que nos va a mantener a nosotros…tu sae’, estables porque to’ lo que yo he oído de cura y de vacunas…yo sé, yo entiendo…yo sé que es asi, que cura para nosotros ya no va a ver, pero sé que este empezando ahora, que vaya a cometer un error pue’ y con los avances que ya hay ahora, se evite que se contagie a otra persona…pue’ seria grandioso para esa persona, tu sae’. Allá nosotros cometimos un error, pue’, nosotros no sabíamos na’ de eso, pero nos tocó.

**ENTREVISTADOR: Ahora quiero que pienses en el ambiente en donde tu estas, donde tú te desenvuelves donde tú vives…en el ambiente… ¿de qué manera, si alguna, ese ambiente influyo en la decisión de dejar de tomar los medicamentes? El ambiente en donde estabas.**

Participante masculino 2: Donde estaba…

**ENTREVISTADOR: Donde estabas en ese momento que dejaste de tomarte los medicamentos.**

Participante masculino 2: Pue’, como le dije, estaba en la adicción. Al encontrarme de un dia a otro sin dinero, de ir a conseguir sustancia para poder…este...pue’ utilizando la sustancia como tal. Pue’ me olvidaba…no quería ni tocar los medicamentos. Porque nada…todo lo que me decían o todo lo que yo hacía…todo me molestaba.

**ENTREVISTADOR: Y en ese ambiente había como que…la gente, las personas con las que te relacionabas…sabían de la condición?**

Participante masculino 2: No. Pero pue’, yo hacía mis cosas en ese sitio pero yo siempre protegía a los demás. Nunca, este…le dije a otra persona “usa de lo que yo tenía” o que me venga a decir “préstame eso porque no tengo…yo mejor decidía…allá a veces se lo decía a la gente. No lo decía lo que tenía la condición pero le decía…”tú no sabes si yo tengo este…estoy infectao’ con el VIH o algo y tu estas pidiéndome esto para usarlo”…“No, no importa” Pero yo siempre decía no dárselo porque como yo estoy seguro de lo que yo tengo yo no quería pasárselo a otra persona. Porque siempre me voy a quedar con ese sentido de culpa.

**ENTREVISTADOR: ¿En esos momentos tenías trabajo? Anteriormente a la adicción… ¿trabajabas?**

Participante masculino 2: Muchos años atrás, hace como cuatro o cinco años atrás…mi tío tiene una mueblería y estuve trabajando con él.

**ENTREVISTADOR: ¿Y ya tenías la condición cuando trabajabas con él?**

Participante masculino 2: Sí, sí porque yo estuve en estados unidos. Eso fue en el ’91. Entonces yo regrese pa’ acá en el ’95 creo que fue.

**ENTREVISTADOR: Y en el ambiente de trabajo, verdad, cuando te tomabas los medicamentos…si te tocaban los medicamentos tomártelos ahí, te los tomabas o era momento que no te los tomabas…**

Participante masculino 2: Sí, me los bebía. Pero había veces que me los bebía fuera de hora.

**ENTREVISTADOR: Ok. ¿Para qué no te vieran?**

Participante masculino 2: No, no pa que no me vieran, sino que…vamo’ a ponel que yo me levantaba por la mañana, me bebía el de por la mañana porque desayunaba. Entonces me iba a trabajar…salía a almorzal me los bebía, pero había veces que salía tarde del trabajo, entonces, o sea que me cogía horas extra y pue’ yo…a la hora que me los bebía siempre no me los bebía a esa hora porque estaba haciendo otra cosa…entonces…más tarde era que me los bebía. Y había veces que me olvidaba de ellos porque como era tan tarde no quería bebérmelos.

**ENTREVISTADOR: Ah, okay. ¿Por alguna razón en específico?**

Participante masculino 2: A lo mejor era porque…me pongo a pensar era porque me iban a lo mejor…como la Sustiva, esa Sustiva uno se la bebe y le pone a uno como…bueno una vez yo la use dos veces por equivocación en el dia y yo sentía que yo estaba tumbao’…yo pue’…me sentía raro. Y cuando me puse a chequial bien era que me había bebio’ la Sustiva esa. Me la bebí por la otra.

**ENTREVISTADOR: Ah okay. ¿Cuántos medicamentos tomas al dia?**

Participante masculino 2: Este, bebo dos pastillas por la mañana y dos por la tarde

**ENTREVISTADOR: Son cuatro al dia?**

Participante masculino 2: Ajá.

**ENTREVISTADOR: Y el ambiente, verdad, volviendo a lo del ambiente donde te desenvuelves…el ambiente donde jangeas, donde socializas, ¿Cómo influyó eso en que dejaras de tomarte los medicamentos?**

Participante masculino 2: Pue’ viendo a to’ el mundo…to’ el mundo se pasaba to’ el día ahí…yo como no tenía na’ que hacer me quedaba ahí to’ el día y me olvidaba de irme a beber los medicamentos…

**ENTREVISTADOR: ¿No te los llevabas contigo?**

Participante masculino 2: No. Aunque había veces en que yo me lo echaba en el bolsillo. Y teniéndolo en el bolsillo también se me olvidaba.

**ENTREVISTADOR: Y en ese momento, verdad, cuando se te olvidaban, era porque estabas bajo el efecto de la sustancia o era que pues, como que no te importaba…?**

Participante masculino 2: Pue’, pue’, buscando a veces…cuando estaba buscando para la sustancia, después que me sustanciaba pues lo tenia encima pero me olvidaba de ellos, entonces cuando me vengo a acoldal es tarde…

**ENTREVISTADOR: ¿Estabas bajo los efectos…?**

Participante masculino 2: Eso es así.

**ENTREVISTADOR: Este…y el lugar donde recibes los servicios médicos, ¿de alguna manera influyo para que tu dijeras como “yo estoy molesto con estos, con ese lugar”, o algo así?**

Participante masculino 2: No.

**ENTREVISTADOR: ¿Algún otro lugar que tú crees que haya influenciado en eso?**

Participante masculino 2: Tampoco.

**ENTREVISTADOR: Ok. Ahora quiero que pienses en Puerto Rico. Las cosas que están pasando en Puerto Rico, verdad. ¿De qué manera, si alguna, las cosas que pasan aquí en Puerto Rico influyen en la decisión de dejar de tomar los medicamentos?**

Participante masculino 2: No creo. Yo sé que van a haber problemas, van a habel este…como se dice…asesinatos, van a habel choques, va a habel violencia domestica…va a habel de todo, pero pue’ no creo que eso sea para uno tener que dejar de beber los medicamentos. No creo.

**ENTREVISTADOR: Y por ejemplo, cosas así como, como el gobierno, las políticas del gobierno y todo eso, esas políticas que tu crean que…como que no ayuden al paciente…**

Participante masculino 2: Yo, pue’ no…bueno de bebérmelo como tal no. Porque de bebérmelo yo me lo bebo, pero hay a veces que el gobierno, si se supone que hay unos fondos para los pacientes y están ahí, porque uno a veces va a los sitios que uno se atiende y siempre le dicen a uno que no tenemos fondos o no tenemos esto…

**ENTREVISTADOR: ¿Y qué está pasando con eso…que fue lo que paso con eso?**

Participante masculino 2: No sé, en velda no sé. Puede ser que uno pida ayuda y le dicen a uno que no hay fondo, que por el momento no se puede hacer na’…

**ENTREVISTADOR: Y en algún momento te han…por ejemplo, te han…no te han dado el medicamento, se han tardado en darte el medicamento, por falta de fondo?**

Participante masculino 2: No. Los medicamentos siempre han estado ahí. Pero como otras cositas que uno pida a veces para que lo ayuden y cosas así…

**ENTREVISTADOR: ¿Qué otras ayudas le dan…?**

Participante masculino 2: Este…como decir los suplementos también…

**ENTREVISTADOR: Ah ok.**

Participante masculino 2: O si es que estas viviendo aparte le pagan la luz, el agua, esto, aquello…Tu sae’ cosas que…de emergencia que le puedan ayudar a uno.

**ENTREVISTADOR: ¿Y los suplementos que le dan de suplementos?**

Participante masculino 2: Bueno, ahora mismo, allá en Juana Díaz, te los daban. Lo están dando, pero que allí mismo atendían a uno. Ahora están mandando a uno aquí a la playa pa ver al nutricionista que vea a uno, entonces evalué a uno para que entonces ellos no los den allá. Entonces acá le ponen a uno…vamo’ a ponel dos cajas al mes…pues uno va allá y lo que le da es una caja al mes…

**ENTREVISTADOR: Ah, okay.**

Participante masculino 2: Entonces ellos dicen que pue’, como no hay dinero y hay que volver a comprar y pue’. Como tal no me han dado dos cajas a mí, siempre me han dado una.

**ENTREVISTADOR: ¿Más que una? Ah okay. Y me habías dicho de los deducibles, verdad, que no tenías para pagar los deducibles…**

Participante masculino 2: Eso es así.

**ENTREVISTADOR: Que tarjeta de plan médico tú tienes, privado o del gobierno?**

Participante masculino 2: La del gobierno.

**ENTREVISTADOR: ¿Y aun así tienes que pagar deducible?**

Participante masculino 2: Dice cero, y estaban cobrando tres dólares por…por cada…allá me cobraban como seis pesos por tres potes de medicamento que me daban.

**ENTREVISTADOR: ¿Y porque ellos cobraron, si dice cero?**

Participante masculino 2: No sé.

**ENTREVISTADOR: Y si no pagabas los tres dólares, no te daban…**

Participante masculino 2: No me daban los medicamentos. Entonces ahora yo lo estaba diciendo allá en la clínica entonces me dijeron “ah pue’ dígale a ellos que me llamen” pue’ yo le dije que lo llamaran. Y entonces ellos cubrieron los gastos del deducible, pero yo estuve mucho tiempo…porque yo antes no pagaba…entonces empezamos a pagal y a pagal y yo digo porque nosotros estamos pagando si yo tengo una cubierta especial que se supone que yo no estuviera pagando deducible y estoy pagando deducible. Y no soy yo na’ ma’! Son todo el mundo allí, todos los pacientes allí.

**ENTREVISTADOR: Y muchos de los pacientes que tú conoces, que le cobran deducible, ¿tampoco la han…muchas veces tampoco tienen los medicamentos? ¿No tienen para pagarlo?**

Participante masculino 2: Bueno, buscan la forma, como…no sé. Buscarían por otro lao’ cuando no hay fondos ahí o algo pero pues, antes…tenían el mismo problema que yo.

**ENTREVISTADOR: Okay. Alguna vez, verdad, por la transportación...has tenido problemas de transportación y por eso se te ha hecho difícil buscar los medicamentos…**

Participante masculino 2: No, porque como yo vivo cerca de allí, pues nunca…casi nunca pido…nunca le he pedido transportación. Solamente para acá pa’ Ponce, que dos veces. Dos veces desde el ’96 y hasta ahora, dos veces na’ más le he pedido transportación para acá.

**ENTREVISTADOR: Alguna otra barrera o situación, verdad, que ocurran asa como lo que estamos hablando de que si los servicios del país, la parte económica, cosas así, verdad, que otra barrera tú crees que haya, otra situación que influye…Ahora piensa en las cosas como las costumbres del país, las creencias, verdad de nuestra sociedad, de qué manera si alguna tú crees que las costumbres de nuestra sociedad influyen en la decisión de dejar de tomar los medicamentos.**

Participante masculino 2: Pues yo no creo.

**ENTREVISTADOR: ¿Te has sentido discriminado alguna vez por tener la condición?**

Participante masculino 2: Bueno, hasta le fecha no.

**ENTREVISTADOR: …rechazado…**

Participante masculino 2: Hasta la fecha no.

**ENTREVISTADOR: Verdad, si no te ha pasado a ti, como tú crees que sentirse rechazado por tener la condición tú crees que eso influye en la decisión de dejar de tomar lo medicamentos.**

Participante masculino 2: Bueno hay personas que son débiles de mente y al pensar esas cosas dejan de beberse los medicamentos o que pueden hacer otras cosas peores que esa como pensar en el suicidio…tu sae’ eso está aquí en la mente de uno. Si uno es flojo de mente to’as esas cosas van a venir a uno. Pero gracias a Dios yo siempre me he mantenido firme. Hasta que Dios quiera.

**ENTREVISTADOR: Y has escuchado de personas que, como tú dices, estar flojos de mente quizás los dejen de tomar, porque digan “me están rechazando, me siento a un lado”…**

Participante masculino 2: No.

**ENTREVISTADOR: Por ejemplo, ¿si tú crees que las creencias religiosas pueden influir en que uno deje de tomarse los medicamentos? ¿Alguna creencia religiosa?**

Participante masculino 2: No creo.

**ENTREVISTADOR: La situación social que uno vive, eso crees que influye en el…**

Participante masculino 2: Bueno, no sé. Yo pensaría que de ser así porque no tuviera la forma de como tener los medicamentos o…pero de que los tuvieran yo no creo que tenga ningún problema en bebérselo. O que se yo…

**ENTREVISTADOR: Ahora quiero que pienses en esta época que estamos viviendo…en los tiempos que estamos viviendo. ¿De qué manera estos tiempos en que vivimos influyen la decisión de dejar de tomarse los medicamentos para el VIH?**

Participante masculino 2: pues, habrán personas…habrán personas con…no le digo de mí. Porque yo…puede ser que haya personas que se sientan solas, que no tengan familia, que nunca han tenio’ por lo menos una fiestecita en la Navidad, que se hayan sentido apoyadas. Porque lo ha habido…son personas que a veces se sienten rechazadas, ya no le importa más na’, dejan de bebérselo y por ahí siguen hasta que Dios los mande a buscar si…

**ENTREVISTADOR: ¿Algo más? Ahora cuando en el pasado dejaste de tomarte los medicamentos, ¿qué te ayudo a volver a tomártelos?**

Participante masculino 2: Bueno, cuando, pue’ como ahora. Empecé a buscar ayuda otra vez para venir…porque mientras yo esté bien yo siempre voy a estar con los medicamentos al dia…voy a estar bebiéndomelo…pero cuando estoy mal, yo siempre…como ahora estoy acá, busque ayuda.

**ENTREVISTADOR: ¿Acá fue que buscaste la ayuda?**

Participante masculino 2: Eso es así.

**ENTREVISTADOR: ¿Cuánto hace que estas acá?**

Participante masculino 2: Bueno, en esta vez yo llevo dos meses y cuatro días ya yo llevo aquí. Tuve que hacerlo porque ya me estaba sintiendo otra vez que se estaba descontrolando mi salud. Dejando de beberme los medicamentos y pues me puse a pensar primero yo antes de nada. Me vine para acá.

**ENTREVISTADOR: Tenias la carga viral…como estaba?**

Participante masculino 2: Estaba todo….la carga viral la tengo bajita. No me acuerdo en cuanto fue. Pero la hemoglobina la tenía en 10 y pico.

**ENTREVISTADOR: Las defensas…**

Participante masculino 2: Y los glóbulos blancos la tenía en…no me acuerdo el numero…

**ENTREVISTADOR: Pero estaban bajitos?**

Participante masculino 2: Estaban to’ bajito.

**ENTREVISTADOR: ¿Todas las defensas estaban bajitas?**

Participante masculino 2: Estaban bajitas. Las defensas, sí.

**ENTREVISTADOR: ¿Que le dirías a una persona que está pensando en dejar de tomarse los medicamentos?**

Participante masculino 2: ¿Qué le diría?

**ENTREVISTADOR: Si.**

Participante masculino 2: Que eso sería tremendo error dejarlos, porque pue’ ya que ha empeazao’ a beberse los medicamentos de dejarlos, eso lo que esta es destruyéndose el mismo. Esta echando su vida al hoyo como digo yo, porque eso lo que va a mantener a uno bien.

**ENTREVISTADOR: Y has tenido oportunidad de hablar con personas que han dejado de tomar el medicamento.**

Participante masculino 2: No, todavía, pero pue’ llegara el tiempo, porque de aquí mismo a veces llevan a uno a charlas y cosas así, y ahí uno tiene tiempo de venir y si hay esa clase de pregunta, uno da su testimonio también.

**ENTREVISTADOR: Si, exacto. Uno aprende tanto, verdad, de la experiencia del otro.**

Participante masculino 2: Eso es así.

**ENTREVISTADOR: Y si tuvieras la oportunidad, verdad, de hablar con personas que están dejando los medicamentos o que lo dejaron, ¿qué le recomendarías a ellos? Según tu experiencia.**

Participante masculino 2: Bueno, ¿que lo hayan dejao’?

**ENTREVISTADOR: Si, que lo hayan dejado….**

Participante masculino 2: Bueno si tiene la oportunidad otra vez de bebérselo y que este…que todavía le funcione que sigan bebiéndoselo, porque eso es lo que va a mantener a uno. Es como digo yo, ya no va haber cura y si ya tú te los bebes y estas bien…no quiere decir porque te los bebiste vas a estar bien por siempre, tienes que seguir bebiéndotelos para esta bien. Es cuando le da un dolor de cabeza a uno, uno se bebe una Tylenol y se le quita el dolor de cabeza a uno. Pero, vuelve otra vez el dolor y tiene que volverse a beber lo medicamentos otra vez pa poder quitarse el dolor de cabeza. Esto es continúo.

**ENTREVISTADOR: Sí. Algo más que le recomendarías a la persona…así como lo que tú me decías cuando son flojos de mentes, así…**

Participante masculino 2: que se mantengan firmes porque la mente de uno es la que lleva a uno a hacer cosas que uno quiera. Eso es lo ma’ poderoso que tiene uno, la mente. Y si uno deja de beberse los medicamentos porque es débil de mente, digo yo, si se pone a pensar en la enfermedad que tiene de que no va a poder vivir ma’ de lo que quería vivir o que si tú quieres tener una familia la puede tener. Todo lo que quiera tener lo puede tener siempre y cuando se mantenga este…manteniendo la salud al dia. Se puede vivir. Yo al principio lo pensaba…yo creía que todo esto de medicamentos y eso…pero pue’, pero nunca me paso por la mente dejar de bebérmelos. Y de hacerme daño yo mismo. Porque yo creo que pa eso es que esta la ciencia, pa estar bregando con las cosas que están pasando, como las enfermedades y todo eso y pue’…yo por lo menos yo creo en eso y los medicamentos me han mantenio’ bien…siempre me lo he bebido, esas son las únicas veces que yo dejao’ de bebérmelos, cuando estoy sustanciao’ o cuando estoy en la adición que me voy a lo loco por ahí como digo yo, y se me ha olvidao’. Pero todo eso son cosas que llevan a uno a no bebérselas. Mantienen a uno. Eso es lo que mantiene vivo a uno.

**ENTREVISTADOR: Eso es así. ¿Este…sabes lo que es…las recomendaciones médicas, sabes lo que es las recomendaciones médicas?**

Participante masculino 2: ¿Recomendaciones?

**ENTREVISTADOR: Aja.**

Participante masculino 2: Pues, como beberse los medicamentos al dia, a la hora o pue’…sí.

**ENTREVISTADOR: Y cuando escuchas la frase adherencia o cumplimiento con las recomendaciones médicas, ¿con qué lo relacionas?**

Participante masculino 2: No te entiendo.

**ENTREVISTADOR: Okay. Cuando escuchas…esa palabra, recomendaciones médicas, verdad, cumplir con las recomendaciones médicas, que es lo primero que te viene a la mente.**

Participante masculino 2: Bueno, cumplir con lo que me recomienda el doctor. Si tengo que beberme un medicamento porque me va a hacer bien, pue’ cumplir con eso, bebérmelo. Porque se va a hacer el bien pa’ mí. Si el me lo recomienda es porque está claro de lo que está haciendo.

**ENTREVISTADOR: Y además de tomarte los medicamentos y de recetarte los medicamentos, verdad, el doctor, ¿qué otras cosas te recomienda tu médico que hagas? Además de tomarte los medicamentos. Ya eso, pues, él siempre lo recomienda. ¿Qué otras cosas él recomienda que hagas? O sea, ¿qué otras cosas te recomienda tu médico?**

Participante masculino 2: Bueno, por lo menos él sabe, pue’ las andanzas que yo he tenio’, me recomienda “mira, bébete los medicamentos y deja las loqueras esas que tú tienes por ahí y ponte en un buen vivir y vive una vida sana y cuídate”. El me lo dice…tenemos mucha confianza.

**ENTREVISTADOR: ¿Y él te ha hecho referidos para hacerte otros estudios…?**

Participante masculino 2: Eso es así.

**ENTREVISTADOR: ¿Qué otros estudios te ha hecho?**

Participante masculino 2: Bueno, yo tengo las várices en el esófago que se me han reventao’. Estao’ como cuatro veces en el hospital. Entonces me ha hecho referidos para el baso, el hígado…tu sae’ para hacer el sonograma. Y otro referido para que fue, pero todo eso el…para mí él me ha atendio’ bien.

**ENTREVISTADOR: O sea, que él te recomienda esas…te recomienda esos referidos. Eso también son cosas que te ha recomendado, referidos para…Y alguna de esas recomendaciones o, verdad… ¿se te ha hecho difícil seguirlas…algunas de las recomendaciones que te da el médico?**

Participante masculino 2: Él me ha consiguió…yo la he hecho. Entonces la última vez me volvió a hacer un referido para hacer un sonograma y eso y como le dije, una vez fui, la maquina estaba rota entonces como yo estaba pue’, en la adicción, pue’ deje de ir. No volví a darle seguimiento para que volvieran a hacerme el examen ese y me olvido.

**ENTREVISTADOR: ¿Te lo hiciste después?**

Participante masculino 2: No me lo hice.

**ENTREVISTADOR: ¿Algún otro…alguna otra recomendaciones, de esa, referidos que te ha hecho el medico que se te ha hecho difícil o que no la ha hecho todavía…no la hayas podido hacer?**

Participante masculino 2: Él lo que me recomendó ahora, en esta vez de dejar los medicamentos…perdón…que le mando una carta a ellos, a lo de la clínica para que pudieran hacerme el genotipo. Pero allá me dicen que no porque y que la hemoglobina mía y que subió. Pero el, por parte de él me dijo que me la hiciera…pero ellos no…pues…ellos allá me dijeron que no, que no hacía falta hacérmelo.

**ENTREVISTADOR: Que ahí fue…no fue porque tú…**

Participante masculino 2: No fue por él, sino fue por allá.

**ENTREVISTADOR: ¿Por la clínica?**

Participante masculino 2: Ajá.

**ENTREVISTADOR: ¿Qué otro exámenes médicos, además del genotipo, del baso y eso…qué otros exámenes médicos te recomendó?**

Participante masculino 2: Este…anteriormente…las personas esas que hacen el cateterismo a uno…

**ENTREVISTADOR: ¿Cardiólogo?**

Participante masculino 2: Pues…si…pa’ ir a que me diera seguimiento sobre eso.

**ENTREVISTADOR: Okay. ¿Fuiste?**

Participante masculino 2: Si, una vez. Si eso fue una sola vez que me hizo un referido para allá.

**ENTREVISTADOR: ¿Y salió todo…?**

Participante masculino 2: Había salio’ bien, pero después de eso, como estaba…volví otra vez a…volví a los vicios y eso y al tratar de salirme de los vicios, eso como que me daba otra irritación y volvía a vomitar sangre, a ensuciar sangre…mire como me dejo esto…mire.

**ENTREVISTADOR: Sí. ¿Y eso es qué?**

Participante masculino 2: Esto es de las venas del esófago…las várices. Entonces ellos me metían una manga por ahí con una lucecita y que se yo pa’ pegármelas y que se ello, eso. Y esta vez me quedaron así.

**ENTREVISTADOR: Y eso fue por el uso de sustancias o por la misma condición?**

Participante masculino 2: No sé qué fue eso. Yo sé que antes a mí me daban unos dolores bien terribles en estomago…por muchas horas. Eso fue en Nueva York cuando yo estuve por allá. Ahí yo me vine para acá y esos dolores me venían con frecuentemente. Pero una vez, trate de dejar el vicio y estando aquí…la primera vez estando aquí me dio eso. Empecé a vomitar sangre…a ensuciar sangre…me tuvieron que dar transfusión de sangre.

**ENTREVISTADOR: Y ahora mismo… ¿le dio no hace mucho?**

Participante masculino 2: Hace como...yo le digo como cinco años atrás.

**ENTREVISTADOR: Entonces, ¿se ha dado seguimiento con eso?**

Participante masculino 2: Bueno, hasta la fecha no. Bueno…me han chequiao’ sí, pero que no…

**ENTREVISTADOR: ¿Está todo bien?**

Participante masculino 2: Si.

**ENTREVISTADOR: Alguna otra…algún otro examen que se haga…que le haya recomendado el medico…referidos para otra cosa…o algo mas o alguna otra condición…Me dijo que el cardiólogo fue uno, verdad, que lo chequeo y todo estaba bien. ¿Le mando otro referido para el cardiólogo, para seguir viéndolo?**

Participante masculino 2: Eh…no me acueldo…no me acueldo…

**ENTREVISTADOR: Me imagino que fue…lo de las várices, ¿quién se lo está atendiendo?**

Participante masculino 2: Eh…se me olvidó el nombre de él…

**ENTREVISTADOR: Pero la especialidad… ¿gastroenterólogo o algo así?**

Participante masculino 2: Eso el que le dicen…este… ¿cateterismo es que le dicen?

**ENTREVISTADOR: Cateterismo es para el corazón…**

Participante masculino 2: No…este…yo no sé cómo…

**ENTREVISTADOR: ¿Endoscopia?**

Participante masculino 2: Endoscopia…endoscopia…perdón.

**ENTREVISTADOR: ¿La endoscopia se la hizo?**

Participante masculino 2: Sí. Eso fue lo que me hicieron. Una endoscopia entonces…

**ENTREVISTADOR: Y colonoscopia, ¿también le hicieron?**

Participante masculino 2: Eh…no.

**ENTREVISTADOR: ¿No se la ha recomendado el doctor?**

Participante masculino 2: No…todavía.

**ENTREVISTADOR: Y las citas médicas… ¿ha tenido que faltar a una cita médica? ¿O a veces se le hace difícil llegar a los sitios…?**

Participante masculino 2: Yo…las citas médicas siempre…el doctor que yo tengo esta al lao de casa. Entontes la clínica queda un poco más abajo. A menos que no sea un referido que ellos me den para acá pa’ Ponce o algo, pero nunca…siempre he ido a las citas…siempre.

**ENTREVISTADOR: Y cuando estaba en la adicción, ¿iba a las citas médicas?**

Participante masculino 2: A veces no iba, entonces ‘ah yo voy y la cambio, yo voy otro día” que se yo.

**ENTREVISTADOR: ¿Y era por la adicción?**

Participante masculino 2: Por la adicción.

**ENTREVISTADOR: Y que…trataba siempre de mantenerse…estaba bajo los efectos de la adición o era que…**

Participante masculino 2: No, porque y que no tenía para buscar sustanciame. Eso como no me dejaba tranquilo pues tenía que estar….ese era el problema

**ENTREVISTADOR: Hay algo más que quisiera añadir a este tema, que no hemos hablado, algo que usted entienda que es importante**

Participante masculino 2: Así que me venga a la mente ahora mismo, no. No tengo na’ que decir

**ENTREVISTADOR: Así que hemos terminado la entrevista.**

Entrevista #3

Participante

**ENTREVISTADOR: Entrevista número tres. Okay, acá el participante ya se le explico el consentimiento informado. Firmó como autorizando al estudio. Así que vamos a comenzar con la entrevista. ¿Desde cuándo estas tomando medicamentos para la condición, para el VIH?**

Participante masculino 3: Hacen 26 días.

**ENTREVISTADOR: 26 días. Comenzaste… ¿hace 26 días que te detectaron la condición?**

Participante masculino 3: No. La condición me la detectaron en el 1990.

**ENTREVISTADOR: Ah okay. ¿Así que en 26 días que comenzaste con el tratamiento?**

Participante masculino 3: Eh, estaba tomándolo antes y los deje y volví a comenzarlo a los 26 de noviembre.

**ENTREVISTADOR: Te felicito por la decisión. Cuando te dieron…te detectaron la condición, te dieron el diagnostico, ¿empezaste a tomar los medicamentos rápido allí o paso algún tiempo después que te detectaron la condición? ¿Pasó algún tiempo para tomarte los medicamentos?**

Participante masculino 3: Pasaron casi 14 años.

**ENTREVISTADOR: Ah, okay…**

Participante masculino 3: Después…después de que me diagnosticaron la condición.

**ENTREVISTADOR: ¿Y fue por qué no tenías que tomártelos o fue que no querías…sabes…?**

Participante masculino 3: No quería tomármelos.

**ENTREVISTADOR: No querías tomártelos, ah okay.** **Pero si el médico…cuando te la detectaron el médico te dijo que tenías que tomártelas. Me dijiste que fue en el 90, ¿verdad?**

Participante masculino 3: Si.

**ENTREVISTADOR: Hace ya mucho tiempo.**

Participante masculino 3: 22…

**ENTREVISTADOR: 22 años. Y el lugar donde te trataban, ¿tenía los medicamentos para tratar el VIH?**

Participante masculino 3: Sí.

**ENTREVISTADOR: ¿Lees la información que explica los efectos secundario de los medicamentos?**

Participante masculino 3: Sí.

**ENTREVISTADOR: ¿Si? ¿Y qué sabes de los efectos secundarios? ¿Qué te…qué efectos secundarios tienen los medicamentos que…?**

Participante masculino 3: Por el momento no me han dao’ ninguno, pero el doctor que me medicó los medicamentos me dijo que los efectos secundarios podían ser rash en el cuerpo, que…malestar en el estómago, pues como también tengo…este…deteriorado también pues…el hígado, el estómago, un poco de dolor de cabeza y vómitos y nauseas.

**ENTREVISTADOR: ¿Te ha dado uno de esos?**

Participante masculino 3: No, gracias a Dios.

**ENTREVISTADOR: Gracias a Dios. Ahora desde los 26 días que llevas. ¿Y antes te había dado?**

Participante masculino 3: No.

**ENTREVISTADOR: ¿No? ¿Qué piensas sobre los medicamentos para tratar el VIH?**

Participante masculino 3: Pues, pensaba antes que era incomodo, pues…de solo pensar que estaba tomando medicamentos para poder adaptar la vida de uno. Por…el diagnostico. Pero era un poco ignorante porque no tenía la orientación perfecta de lo que eran los medicamentos y la enfermedad. Pero luego que he recopilado un poco de información pues pienso que los medicamentos, pues, me están siendo efectivos. Que antes me levantaba un poquito desanimao’ y pues, ahora tengo un poquito de más de ánimo.

**ENTREVISTADOR: Y esa información, ¿cómo tu…cómo la has recopilado? ¿De dónde la sacaste?**

Participante masculino 3: Pues, en diferentes charlas que he ido. Como la psicóloga de aquí que viene parte de parte de Ryan White. Y con mucha literatura.

**ENTREVISTADOR: Este… ¿qué esperas del tratamiento para el VIH? ¿Qué tu esperas?**

Participante masculino 3: ¿En cuál aspecto?

**ENTREVISTADOR: ¿Cómo la expectativa que tú tienes del tratamiento? ¿Que tú crees que va a hacer?**

Participante masculino 3: Pues, primero que nada, poniendo a Dios por delante, yo espero y confió que los medicamentos me van a ser efectivos. Porque ya estoy sintiendo como el cambio en el sistema. Por los medicamentos.

**ENTREVISTADOR: ¿Qué cambios has sentido?**

Participante masculino 3: pues, que antes me levantaba un poquito sin fuerzas, desanimao’. Y ya estaba sintiendo las calenturas que le dan de noche a uno. Y ya eso se me ha quitado.

**ENTREVISTADOR: Cuando escuchas comentarios de otras personas que han dejado de tomarse los medicamentos para el VIH, ¿qué piensas de eso?**

Participante masculino 3: Pues, unas veces veo la moneda de las dos caras. Porque algunos comentan que es que hicieron daño, o que no estaban siendo efectivos en ellos. Y pue’, he pensado de que a lo mejor no han indagado en otros medicamentos, porque hay un número de medicamentos pa’ la condición. Pues y tal vez algunas veces pienso que todavía están un poquito no orientados referente a los medicamentos.

**ENTREVISTADOR: Que fue lo que te paso a ti, ¿verdad?**

Participante masculino 3: Sí.

**ENTREVISTADOR: ¿No estabas bien orientado? ¿Y qué razón dan esas otras personas para dejar de tomarse los medicamentos? Que hayas escuchado.**

Participante masculino 3: Pues, mucho de los que yo he conocio’ que han tomado medicamento, mucho lo han dejado porque han decaído en las sustancias controladas otra vez. Este…se le interrumpe el tratamiento. Este…y pue’, y muchos que el efecto secundario no han podido tolerar.

**ENTREVISTADOR: Y además de dejarte…tú te has dejado de tomar los medicamentos, obviamente, por eso es que estas acá, verdad. Te has dejado de tomar los medicamentos en alguna ocasión.**

Participante masculino 3: Estuve…me lo diagnosticaron en el 90, después de 14 años comencé a usarlo, y estuve un periodo de cinco meses, y luego estuve…después de eso, 6 años más sin tomarlos. Hasta el 26 de noviembre de este año.

**ENTREVISTADOR: Y has pensado…a veces uno deja de tomarse los medicamentos, ¿verdad? Y lo hace…ya está hecho. Pero a veces hay personas que piensan en dejar de tomarse los medicamentos pero no necesariamente lo dejan. ¿A ti te pasa eso? Como qué piensas en dejarlos de tomar…**

Participante masculino 3: Fíjate, siendo sincero…este, y no es que he pensado, es que pue’…pues le pregunto a Dios, si es necesario yo tomarlo por siempre o hasta cierto tiempo de que yo me haya recuperado los C4, la carga viral, o etc. Pero, por dejarlo, todavía no me ha llegado ese pensamiento. Pero si, como ser humano en mi conducta de ser humano le pregunto a Dios si voy a estar con ellos toa’ mi vida o hasta que me estabilice las defensa y la carga viral.

**ENTREVISTADOR: Y has recibido alguna respuesta, así como, de Dios, como que sientes…**

Participante masculino 3: Este…por el momento no he recibio’. Por el momento todavía siento que necesito seguir tomando los medicamentos.

**ENTREVISTADOR: Dosis de los medicamentos… has dejado de tomar…por ejemplo uno deja de tomarse los medicamentos por mucho tiempo pero hay veces que como se le olvida una dosis. ¿Te ha pasado eso?**

Participante masculino 3: Por el momento he sido bien responsable en cuanto a eso.

**ENTREVISTADOR: Que cuando te los empiezas a tomar, las dosis no…no las…**

Participante masculino 3: No.

**ENTREVISTADOR: A muchas personas, verdad, como te ha pasado a ti, se les hace difícil tomarse las pastillas todos los días, por diversas razones que están dentro y fuera del control de uno. Ahora te voy a hacer unas preguntas sobre las posibles razones para que en algún momento hayas dejado el medicamento para el VIH. Quiero que pienses ahora en ti, solamente en ti, si incluir otras personas o sin incluir otras circunstancias. ¿Consideras que tengas, algún sentimiento, pensamiento o creencia personal, verdad, tuya, que influyó en dejar de tomarte el medicamento?**

Participante masculino 3: Pues, el uso de las sustancias controladas.

**ENTREVISTADOR: ¿Cuánto tiempo estuviste usando sustancias?**

Participante masculino 3: Estuve, casi 6 años. De forma consecutiva.

**ENTREVISTADOR: Y durante esos seis años, ¿no los estuviste tomando?**

Participante masculino 3: No, porque recuérdese cuando uno entra al ambiente de ahí de las drogas, uno se torna a ser irresponsable por tu salud, irresponsable con los demás, y hasta irresponsable con las citas médicas y todos los costos.

**ENTREVISTADOR: Por ejemplo, cuando a veces hay personas que cuando dejan de tomarse los medicamentos, se sienten…además del uso de sustancias, verdad, o a veces, que se yo, tienen algunos sentimientos de coraje, miedo, tristeza…sentimientos…algunos de esos sentimientos tu tenías…que sentías, verdad, o tenías algunos de esos sentimientos que quizás influían para que dejaras de tomarte los medicamentos…**

Participante masculino 3: Pues, en el momento que me diagnosticaron la enfermedad, que el doctor me hizo la receta para ir a tomar los medicamentos, comenzar…pues ese momento los sentimientos que me dieron fue de que “wow, esta….a tomar unos medicamentos, porque estoy enfermo con una enfermedad que es muy alertadora y muy abarcadora en el mundo que es la del SIDA.” Y el sentimiento que me dio fue temor de que me…de que no me funcionaran y pudiera perder mi vida.

**ENTREVISTADOR: Y ese sentimiento de temor, te hacia decir “pues mira, no me los voy a tomar”.**

Participante masculino 3: No, en todo momento, me…pensar simplemente en tomármelos, tu sabes, y ver tanto medicamento, pue’ me desmotivo a seguir tomándomelos.

**ENTREVISTADOR: ¿Te desmotivaron?**

Participante masculino 3: Me desmotivaron.

**ENTREVISTADOR: ¿Ver tanto medicamento?**

Participante masculino 3: Sí.

**ENTREVISTADOR: Y sentir…el ver tanto medicamento y lo otro era que...**

Participante masculino 3: Me sentí desmotivado a tomar tanto medicamento.

**ENTREVISTADOR: Y por ejemplo, este…si en algún momento te sentías tan y tan triste, por ejemplo…a veces hay personas que le puede pasar, que se sienten triste así como…como que le apestan las cosas, la vida, como dice uno por ahí…no tomar los medicamentos. ¿Te ha pasado eso?**

Participante masculino 3: Yo le doy gracias a Dios que todavía no he pasado por esa situación de que me apeste la vida, ni…ni nada de eso. Simplemente que como ser humano, pue’…le de este sentimiento a uno de uno cargar con una enfermedad así. Pero de que me apeste la vida, con todo y eso he tenido la enfermedad…he sido bien consciente de que ha sido por, pue’, por malas decisiones que he tomado. O sea que respecto a eso, pues no he llegado que me apeste la vida.

**ENTREVISTADOR: Coraje, por ejemplo, que a veces a uno le da coraje y coraje, pues, como uno dice toma decisiones con coraje…te da coraje y tomas la decisión de no tomarte los medicamentos, o algo así.**

Participante masculino 3: Nunca…nunca he pasao’ por eso.

**ENTREVISTADOR: Pensamientos…¿qué pensabas en esos momentos cuando dejabas de tomar los medicamentos, qué venía a tu mente? ¿Qué te pasaba por la mente?**

Participante masculino 3: Cuando me los deje de tomar?

**ENTREVISTADOR: Aja. ¿Cuándo te los dejabas de tomar?**

Participante masculino 3: Pue’, este…”wow, Dios mío, y si por no tomarme los medicamentos, pue’, este…me…me…fallezco más temprano. Por ignorancia, deje de no tomarlos. Aunque…aunque mi pasara lo que me pasara. Entiendes? Porque estaba todavía ignorante de que podía hacerme los medicamentos, hasta donde yo podía llegar con los medicamentos, temor también a los efectos secundarios. Porque oía muchos mitos de ellos, y esos fueron los primeros pensamientos que me habían dado.

**ENTREVISTADOR: Y ese temor, de que te fuera algo…que te fueras a morir, ¿era lo suficiente fuerte como para que tú te tomaras los medicamentos, o aun así como que no…verdad…?**

Participante masculino 3: No, le puse las cosas…le puse las cosas a Dios. Si no me tomo los medicamentos y fallezco, pue’… Pero desistí completamente de no beber más…

**ENTREVISTADOR: A pesar de que tenías esos pensamientos…**

Participante masculino 3: Esos pensamientos…

**ENTREVISTADOR: …era más fuerte lo otro…**

Participante masculino 3: …a veces duda, esas cuestiones, pue’, deje de tomármelas. Y también influyo mucho también…por andar en el vicio de las drogas. Influyo mucho también.

**ENTREVISTADOR: ¿Qué cosas pasaban…que estaban pasando en tu vida en ese momento…cuando dejaste de tomarte…además del vicio? ¿Estaban pasando algunas otras cosas con tu vida…cuando dejaste de tomar los medicamentos?**

Participante masculino 3: Pue’ más o menos para ese tiempo, a mí me diagnosticaron en el 1990…en el 1993 perdí a mi mamá, me encontré solo…me encontré deambulando, y…y también estuve…también…fueron influyente en que yo no cogiera el tratamiento para la condición ni na’…por simplemente me…a perder a mi mamá…este…por simplemente pensar de que no tenía un sitio donde poder cambiarme de ropa o donde asearme para ir bien a las cita, etcétera. Todas esas cosas influyeron.

**ENTREVISTADOR: O sea, que no querías ir a citas…como…estar así.**

Participante masculino 3: No, no.

**ENTREVISTADOR: Sin peinarte, sin…**

Participante masculino 3: Sin nada…sin asearme. Nada de eso.

**ENTREVISTADOR: Querías limpiar…por lo menos ir presentable…**

Participante masculino 3: Sí.

**ENTREVISTADOR: No podías estar así pues no…no te daba el deseo de ir a las citas. ¿Y ahí era que te daban los medicamentos…la receta?**

Participante masculino 3: Sí. El seguimiento que da el internista.

**ENTREVISTADOR: Este…cómo… ¿qué cosas del vicio, verdad, obviamente tienes experiencia con esto…qué cosas del vicios son las que tú dices que como que te hacen decir “contra, estas cosas del vicio es que me hace no querer tomarme los medicamentos”? Sabes que…que del vicio…yo que no conozco verdad, si me fueras como a explicar a mí eso.**

Participante masculino 3: Pues, missi, lo que sucede es que cuando está el vicio de las drogas, este…la heroína y la cocaína que era lo que yo utilizaba, son una sustancia que la heroína es un down y la cocaína es otro. Pue’ cuando uno está en ese ambiente pues uno se siente en todo momento bien. Sae’ si tiene algún dolor de cabeza, algún dolor de un musculo, de espalda…o etcétera etcétera…esa sustancia pue’ te elimina todo eso. En ese momento, que yo utilizaba la droga, no sentía ningún, este…como le digo…efecto o dolor o cualquier cosa de la enfermedad o de cualquier otra enfermedad. Y por eso es que no le podía tanta atención y a los medicamentos ni a los tratamientos, por eso. Porque lamentablemente eso te tapa cualquier dolor y cualquier cosa de tu mente y aunque…y unos son ignorantes porque lo que hacía era perjudicándose más.

**ENTREVISTADOR: Ah, okay. Entiendo ahora. O sea que como tú te sentías bien, pues a lo mejor no…**

Participante masculino 3: Sí, en el momento.

**ENTREVISTADOR: En ese momento…**

Participante masculino 3: Que la utilizaba.

**ENTREVISTADOR: Entonces no veías la necesidad de tomarte el medicamento porque tú te estabas sintiendo bien.**

Participante masculino 3: Pero ahora al estar con la menta limpia y clara, que llevo hace como 9 meses. Pue’ ya uno empieza a cogerle más afecto y cariño a la vida. Tu sae’…y ya uno entiende a instruirse de la enfermedad, a instruirse con los medicamentos y eso la motivación a to’ eso te lleva a tomarte los medicamentos y a seguir el tratamiento…

**ENTREVISTADOR: Y otras personas que tienen la condición, que también tienen la adicción, ¿tú crees que le pasa lo mismo que a ti?**

Participante masculino 3: Sí. Yo diría que unos 85%. El otro 15% pue’ ya hemos perdido la esperanza de seguir viviendo, de seguir luchando y se tiran a morir, como decimos.

**ENTREVISTADOR: ¿Alguna vez estuviste en esa situación?**

Participante masculino 3: No, gracias a Dios, nunca.

**ENTREVISTADOR: Okay. Ahora vamos a hablar de las personas con las que tú te relacionas, ¿de qué manera, si alguna, las personas con las que te relacionas influyeron en que dejaras de tomarte los medicamentos?**

Participante masculino 3: Aparte de que no tengo familiares cercanos, pero personas profesionales como ustedes pues siempre tuve, trabajadora social, consejero, personas de la religión, que siempre me han llevado de la mano, orientándome referente a la enfermad, referente a los medicamentos que debía de preocuparme má’ por tomar los medicamentos para poder alargar un poco la vida. Porque…más cercano, no tengo…no soy…no tengo familia. Pero personas profesionales así como ustedes siempre me han aconsejado.

**ENTREVISTADOR: Y personas que como que no te ayudan, como dicen por ahí, malas compañías o malas personas…no malas personas, sino mala compañías, que no te ayuden a tomarte…sabes, como que no te ayudan como para tomarte los medicamentos.**

Participante masculino 3: Por el momento no existen.

**ENTREVISTADOR: ¿De alguna manera la familia influyo a que no te los tomaras…?**

Participante masculino 3: Nunca, porque…nunca…nunca mi familia supió de la enfermedad y por el momento no tengo familia cercana.

**ENTREVISTADOR: Okay. ¿Tu familia no sabe?**

Participante masculino 3: No

**ENTREVISTADOR: Amistades que hayas tenido que a lo mejor, verdad, de alguna manera…**

Participante masculino 3: No.

**ENTREVISTADOR: A veces hay personas que tienen alguna…se sienten a veces como rechazados por otras personas, personal médico, verdad, y a veces eso mismo los hace pensar o tomar la decisión de no tomarse los medicamentos. ¿Te ha pasado a ti?**

Participante masculino 3: Por el momento no he sentido el rechazo de ninguna persona. Porque como son pocas las personas que conocen de la enfermedad.

**ENTREVISTADOR: Personal que te atiende, o el lugar donde recibes tratamiento de alguna manera eso como que, te ha puesto a pensar en no hacerlo, pero pensar voy a dejar de tomar los medicamentos, como que tengo deseo de dejar de tomar…cosas que pasen, igual donde vas a atenderte.**

Participante masculino 3: Pues de momento, ahí se han portao’ bien cordialmente, llevo poco ya allí. Por el momento no se han puesto eso en mente. Estar motivado para que yo siga tomándome los medicamentos y hasta me regañan si yo no voy a alguna cita o fallo a buscar los medicamentos o etc.

**ENTREVISTADOR: Y las otras personas, cuando los dejaste de tomar, de alguna manera las otras personas que estaban contigo acompañándote en el vicio, esas personas de alguna manera crees que influenciaron para que dejaras de tomar los medicamentos?**

Participante masculino 3: Sí.

**ENTREVISTADOR: ¿Cómo lo…como…**

Participante masculino 3: A perder tiempo a buscar eso. Si como quiera que sea estábamos usando droga, eso no te va a hacer efecto, esas cosas así.

**ENTREVISTADOR: ¿Y qué tu hacías entonces?**

Participante masculino 3: Los escuchaba y me dejaba llevar por lo que ellos decían.

**ENTREVISTADOR: ¿Eran personas que también tenían la condición? Ahora piensa en el ambiente donde tú te desenvuelves, verdad, el ambiente, ¿de qué manera, si alguna, o el ambiente en que te estabas desenvolviendo en ese momento que dejaste de tomarte los medicamentos, de qué manera si alguna el ambiente influyó en la decisión de tomarte los medicamentos? El ambiente en donde estabas.**

Participante masculino 3: Donde estaba? El ambiente que yo estaba era de las drogas. No era recluso ni nada para lo que yo…me tomaba los medicamentos y me los dejaba de tomar.

**ENTREVISTADOR: ¿Y cómo es ese ambiente?**

Participante masculino 3: Viste, missy, de que uno hace esto en un momento en que uno se levanta y es a pensar na’ ma’ en la droga. Sae’ uno no piensa el medicamento ni las enfermedades. Le pasa por la mente a uno que uno está enfermo cuando empieza las drogas.

**ENTREVISTADOR: Y, verdad, además de ese ambiente de las drogas, la comunidad donde tú vives o donde vivías en ese momento, ¿de alguna manera tú crees que el ambiente que había en la…en el sitio donde vivías, verdad, en ese momento, influenciaba para dejar de tomar…para tomar la decisión de dejar de tomarte los medicamentos?**

Participante masculino 3: To’ el mundo desconoce de mi enfermedad.

**ENTREVISTADOR: ¿Trabajabas…o trabajas…trabajaste?**

Participante masculino 3: Trabajaba.

**ENTREVISTADOR: ¿Y en el lugar donde trabajabas, te tomabas los medicamentos? ¿Cuándo estabas en el trabajo, tú los tomabas allí? ¿O estudiabas o estudias?**

Participante masculino 3: No. Trabajaba.

**ENTREVISTADOR: ¿Y porque no te los tomabas allí, en el lugar…?**

Participante masculino 3: Fue cuando estuve casi en los 14 años sin tomar el medicamento.

**ENTREVISTADOR: Ah, okay, que ya estabas…wow fue un montón de tiempo.**

Participante masculino 3: Si.

**ENTREVISTADOR: ¿Me imagino que te subió mucho la carga viral?**

Participante masculino 3: Desconozco porque todo ese año…todos esos años yo no fui más a sacarme los laboratorios ni nada.

**ENTREVISTADOR: ¿Y cuándo sacaste el primer laboratorio? ¿Lo tenías bien alto?**

Participante masculino 3: En 13 mil, na’ ma. Y eso que fue con el vicio. Que yo pensaba que ya yo estaba ya…a punto de…

**ENTREVISTADOR: Además, verdad, de…ah y el lugar donde recibías servicios médicos, ¿ese ambiente de alguna manera haya influenciado en no dejar de tomar los medicamentos?**

Participante masculino 3: ¿En no dejarlos?

**ENTREVISTADOR: el dejarlos, perdón, en dejarlos.**

Participante masculino 3: ¿En dejarlos? No.

**ENTREVISTADOR: Algún otro lugar, además del ambiente de la droga, ¿algún otro lugar que tu creas que haya influenciado…?**

Participante masculino 3: Ningún otro lado.

**ENTREVISTADOR: Y, por ejemplo, personas que tú has visto, ejemplos que tú has visto de otra gente, has visto quizás otro ambiente, alguno otros lugares que influyen a esas otras personas. Que tú conozcas…**

Participante masculino 3: Si puede ser que haya otro…

**ENTREVISTADOR: ¿Y qué…que otros ambientes…como por ejemplo cuál?**

Participante masculino 3: Pues, en la iglesia, otro centro de rehabilitación…

**ENTREVISTADOR: Okay, ¿cosas que pasan en esos…? ¿Cómo que cosas?**

Participante masculino 3: Algunas veces no están los fondos, algunas veces no están los medicamentos, algunas veces por algún contratiempo la personas se desmotiva y dejan de ir a buscar los medicamentos. No los toman más.

**ENTREVISTADOR: ¿Contratiempos en el sitio? ¿Sí? ¿Cuáles son…y has tenido esos contratiempos tu…?**

Participante masculino 3: Por el momento no.

**ENTREVISTADOR: ¿Y que le ha pasado a otras personas? ¿Cuándo van a buscar los medicamentos y los servicios, que contratiempos han tenido, que tú has escuchado?**

Participante masculino 3: Han tenio’ contratiempo de que llegan que ya le toca el dia de tomar los medicamentos o no han llegado o dicen que no tienen los fondos para comprarlos, mucho le dan…le vuelven a pasar el proceso otra vez con el internista o hacer diligencia de otros papeleos más, etcétera.

**ENTREVISTADOR: ¿Entonces se van y no se los toman?**

Participante masculino 3: No es que los dejan. Se desmotivan y no pueden seguir más con este dolor de cabeza, siempre que voy pa’ allá no están o tengo que pasar por otro proceso. Y yo no tengo casi fuerza pa eso…así.

**ENTREVISTADOR: ¿Y eso no te ha pasado a ti?**

Participante masculino 3: Al momento no. Le doy gracias a Dios.

**ENTREVISTADOR: Wow, pero le pasa a otras personas.**

Participante masculino 3: Si, y también porque en un momento le puede pasar a uno también. Pero por el momento gracias a Dios…

**ENTREVISTADOR: Y esa, pensando verdad, ahora piensa en Puerto Rico. ¿De qué manera, si alguna, las cosas que pasan ahora en Puerto Rico, influyen en la decisión de dejar de tomar los medicamentos, ya sea en la tuya o en la de otra persona? En las cosas que puedan estar pasando en Puerto Rico.**

Participante masculino 3: No entiendo porque las cosas que estén pasando en Puerto Rico tienen que influir en mi vida yo dejar de tomarlo. Al revés lo que me debe de motivarme un poco más a seguir tomándomelos para ver, pue’, si yo puedo ser parte de la solución de las cosas que están pasando en Puerto Rico, tu sae’. Aunque pue, yo no estoy educado o haiga sido quien haiga sido pero puedo ser algún instrumento de útil para servir a las cosas que están pasando en Puerto Rico.

**ENTREVISTADOR: Por ejemplo, ¿tú crees que decisiones que toma el gobierno influyen, cosas del gobierno influyen en esa…?**

Participante masculino 3: Mire, missy, este…le voy a ser sincero y bien trasparente referente a eso. Yo me he encontrao’ en lugares como en las cárceles, un sinnúmero de cosas que hay mucho pacientes de la condición de HIV que guardan mucho rencor con el personal…tu sae’, que existe en el gobierno de alta jerarquía, de cuellos blancos, que están directamente relacionados con los fondos de los tratamientos de los VIH. Y conozco personas de que han empezado sus tratamientos y se han encontrado que con sus fondos, con sus…en los sitios que ellos han cualificado pa’ un sinnúmero de cosas por la condición de HIV han perdido todo eso por cuestión del gobierno que está en el momento…tu sae’ o otro gobierno o persona que asiste en el lugar donde ellos cuentan para esos fondos. Y se han desmotivado completamente. Porque se han sentidos rechazados de parte de la comunidad. Porque pueden ver más allá…equivocadamente o no equivocadamente, pero pueden ver más allá y crean ese rencor en sus corazones y en su mente de que es que no…nos desprecian, nos marginan y to’as esas cosas. Y yo entiendo que el gobierno en ese aspecto ha destruido muchas vidas y ha lastimado muchas, muchas vidas. El gobierno…

**ENTREVISTADOR: ¿Muchas vidas de pacientes de VIH?**

Participante masculino 3: …de pacientes de HIV.

**ENTREVISTADOR: ¿Y sabes de ejemplo de personas que con ese rencor la han cogido con decir “mira no vale la pena tomarse…”?**

Participante masculino 3: Han desistido de seguir tomando los tratamiento, missy. Han desistido. Por el rencor que cogen…

**ENTREVISTADOR: ¿Y has visto personas que también han tenido ese rencor, por ejemplo, con los servicios de salud, que tenemos en el país, como los servicios de salud, piensan más o menos lo mismo y dejan de tomarse los medicamentos?**

Participante masculino 3: No la he visto, pero la he escuchado.

**ENTREVISTADOR: ¿Qué has escuchado de eso?**

Participante masculino 3: de que han dejado de tomar medicamentos, como le dije orita, por que han ido a sus respectivas agencia donde lo ayudaban y CDT y consultorios médicos, etcétera. A bregar su situación de los medicamentos y seguimiento y han tenido muchas dificultades, sae’. Simplemente ven de que porque lo echan pa’ lao’ por la condición del VIH.

**ENTREVISTADOR: Los echan para un lado, ¿los mismos servicios de salud? ¿Y cómo que los echan para un lado?**

Participante masculino 3: No le dan el tratamiento como tienen que dárselo, y ellos ven cómo es que…de que ya ellos no tienen más remedio.

**ENTREVISTADOR: ¿Algo así te ha pasado a ti?**

Participante masculino 3: Gracias a Dios no.

**ENTREVISTADOR: Y por ejemplo, los planes médicos… ¿por alguna razón verdad, por cuestión de los planes médicos las personas han…has escuchado de las personas que no han podido tomarse los medicamentos…este…por ejemplo, no hayan…que el plan médico no se lo cubra o que a lo mejor pues tengan que pagar deducible, cosas así, y no puedan tomarse por eso?**

Participante masculino 3: Puede ser que haiga visto dos o tres casos. De que yo haiga visto, no…no lo he visto. Pero hace poco tuve una situación de que cuando fue a buscar los medicamentos, pue’, el proceso del lugar donde yo voy a darme el tratamiento, pue’ aparentemente tienen que mandar una receta a un sitio que se llama Adapt, que Adapt aparentemente es que paga los fondos para esos tratamientos, y algunas veces se tardan hasta una semana. Pero que pasa, que ahora se están tardando dos o tres meses para poder aprobar esos medicamentos. Lamentablemente a uno tardarse dos o tres meses de dejar de tomar unos medicamentos, ya se está interrumpiendo en forma abruptamente el tratamiento. Eso está pasando. Ayer yo fui también para saludar y me están explicando que hay personas que se están topando con esa situación, de que se están tardando dos y tres, hasta cuatro meses para poder aprobar esos medicamentos. El lugar donde yo estoy yendo, pues lo que está haciendo es…momentariamente lo que está haciendo es que ellos lo están supliendo. Pero mi temor es, como le dije yo ayer al señor, de que un dia que yo vaya y no estén ni en el lugar…que es en el CDT…y todavía Adapt haya aprobado la receta.

**ENTREVISTADOR: Y ahí…no está en el control de la persona.**

Participante masculino 3: Si, y recuérdese que hay capacidades chiquitas a medias, un poquito más grande, y excelente. Que no todo el mundo piensa igual, no todo el mundo…mucho no toleran la situación lo que hacen es que desisten de tomar los medicamentos al encontrarse con tanto obstáculo.

**ENTREVISTADOR: Wow, okay. Interesante. Gracias a Dios a ti…**

Participante masculino 3: Por el momento, le doy gracias a Dios que no me ha pasado.

**ENTREVISTADOR: Eso es interesante, saber esas cosas que pasan así. Y cosas así además del sistema de salud y así, situaciones como esas, ¿has escuchado otras? O alguna otra situación similar de que no tengan los servicios disponibles, lo medicamentos, o verdad, referidos...**

Participante masculino 3: Por el momento no he escuchao’ nada.

**ENTREVISTADOR: Y hablando de cosas que están fuera del control de uno, verdad, ¿la situación económica del país, de alguna manera, verdad, tú crees que influye que la persona no pueda tener el medicamento o no tienen acceso al medicamento…?**

Participante masculino 3: Bueno…hay dos caras. Esta la cara de la persona que puede costear sus mismos medicamentos, su mismo tratamiento. Y la otra cara es de las personas como yo, que no podemos costear esos medicamentos, que contamos con los fondos que se nos ofrecen para poder costear los medicamentos. Si realmente la situación económica de Puerto Rico si es de esa magnitud, pue’, yo entiendo que los más afectados de los que vamos a ser vamos a ser las personas que somos indigentes, que no…carecemos de poder costear el tratamiento de los medicamentos. Y yo entiendo que eso va a influir mucho en el tratamiento de los pacientes de HIV en Puerto Rico y en otros estados.

**ENTREVISTADOR: ¿Cómo va a influir esto, por ejemplo…que piensas que pueda pasar?**

Participante masculino 3: Pues que al no haber los fondos, sae’, no van a haber tampoco los medicamentos, no van a haber tratamiento, muchos internistas van a desistir de darnos los tratamientos, de ayudarnos…y etcétera.

**ENTREVISTADOR: Y, verdad, cambiando un poquito el tema. ¿Alguna vez la transportación, verdad, el no tener transportación ha impedido que puedas buscar los medicamentos, que no te los puedas tomar por que no los tienes…?**

Participante masculino 3: A mí no me ha pasao’. Gracias a Dios siempre hay vehículo. Pero se y he escuchado de personas que han estado largo tiempo sin tomar los medicamentos por falta de transportación.

**ENTREVISTADOR: ¿Alguna otra situación que esté pasando en el país que tú crees que afecte de alguna manera así, como directamente, el no poder tomarse los medicamentos?**

Participante masculino 3: Pue’, esas familias que actualmente han sido víctimas de la criminalidad, que haiga pacientes de HIV envueltos en la familia, puede ser que la misma situación ellos también han desistio’ de haberse tomado los medicamentos, porque recuérdese que la enfermedad del VIH también, cuando tenemos la viralidad alta y las defensas un poco bajas, pues esa enfermedad ataca también parte del cerebro. Entonces nos tendemos a desmotivarnos, a no poder pensar bien las cosas, y etcétera, y yo entiendo que eso puede hacer mucho daño.

**ENTREVISTADOR: Y piensas…ahora yo quiero que pienses en las costumbres de nuestro país…las creencias de nuestra sociedad… ¿de qué manera, si alguna, esas costumbres y esas creencias de nuestra sociedad influyen en la decisión de dejar de tomarse los medicamentos?**

Participante masculino 3: El que dirán. El qué dirán…yo creo por las costumbres y los valores que mucha gente en Puerto Rico…digo, como digo…tenían, porque muchos valores se han perdido. Y las costumbres…el que dirán “pero wow este se está tomando medicamentos para la enfermedad del VIH” y lo ven y entonces muchos de ellos desisten de tomárselos para que no los sigan haciendo comentarios de ese…

**ENTREVISTADOR: Sería, como el discrimen, ¿verdad?**

Participante masculino 3: Sí.

**ENTREVISTADOR: ¿Te ha sentido alguna vez discriminado por tener la condición?**

Participante masculino 3: Por el momento no.

**ENTREVISTADOR: …porque no mucha gente sabe…**

Participante masculino 3: Sí.

**ENTREVISTADOR: ¿Y las creencias religiosas, creen que influyen? ¿Pueden influir?**

Participante masculino 3: Para mí no, para mí no.

**ENTREVISTADOR: ¿Y otras personas? ¿Para otras personas, que hayas escuchado?**

Participante masculino 3: No he escuchado tampoco…de…

**ENTREVISTADOR: Y la situación social, verdad, la situación social que vive la persona, ¿crees que influye o han influido?**

Participante masculino 3: Puede influir, porque recuérdese, que hay personas de alta sociedad que esa enfermedad le ha tocado a las puertas, y prácticamente muchos también han decaído y han, pue’, mermado en sus pensamientos y sus ideas. Y le ha afectado también la sociedad alta, la media y la baja.

**ENTREVISTADOR: Y tú crees que alguna de esas sociedad, de esas clases sociales, eso influya en que…dependiendo de qué clase social sea influya en la decisión de tomarse los medicamentos a las personas…**

Participante masculino 3: Como le dije orita, missy. El qué dirán, porque recuérdate que la sociedad alta, usted sabe, que no deja pasar nada de eso. “Uy mira, fulano, fulano…” y aun también la media, por la...la más baja sigue…los pobres puede decir que exista más consejo para que se tomen los medicamentos…para darle una motivación para que uno siga el tratamiento. Y también puede existir en sociedad, pero de que es más cargado…es más cargado.

**ENTREVISTADOR: ¿Tú crees que la sociedad baja…de clase baja…dé más apoyo a las personas que tienen la condición…?**

Participante masculino 3: Yo entiendo que sí, missy. Yo entiendo que sí.

**ENTREVISTADOR: ¿…qué la gente de clase alta?**

Participante masculino 3: Yo entiendo que sí, porque le ha tocao’ más las puertas de sus hogares a la clase baja que a la clase alta. Y entonces, entienden ellos…aceptan…un poquito más sensible, tu sae’, y tienen más consejo y ayudar…porque como le ha tocado eso más de cerca de muchos más familiares de la clase alta, y lo sienten igual que nosotros lo hacemos. Entonces, en las otras sociedades, recuérdese que existen, lamentablemente, el orgullo un poco más y muchos tienden a no ser amables ni sensibles con los demás seres humanos.

**ENTREVISTADOR: Alguna otra, verdad, cosa con relación a costumbres o creencias de nuestra sociedad, como esos ejemplos que me dijiste…son muy buenos esos ejemplos…algún otro ejemplo, verdad, ¿qué tu creas que influyan en que una persona decida no tomarse los medicamentos?**

Participante masculino 3: ¿A unos ejemplos?

**ENTREVISTADOR: Sí, ejemplos así.**

Participante masculino 3: ¿De…de?

**ENTREVISTADOR: De cosas…de costumbres y creencias de nuestra sociedad que puedan hacer que uno tome…no se tome los medicamentos.**

Participante masculino 3: Estar envuelto en mucho de la religión…tu sae’ por que una persona tiene una fe…hay personas que tienen fe bajita, como los que tienen la fe demasiao’ de alta desisten de tomarse los medicamentos. Y muchas otras personas que por costumbre, ellos siempre han pensado que las cosas de la naturaleza le convienen usarlo…tomarlo…para la enfermedad y deciden no tomarse los medicamentos común y corriente.

**ENTREVISTADOR: Como que los sustituyen por la fe o por las cosas naturales…**

Participante masculino 3: Por la fe o las cosas naturales.

**ENTREVISTADOR: Oh, okay. Y has visto gente o has escuchado…has visto o has escuchado…**

Participante masculino 3: He escuchado de personas de que por la fe han desistido y se han sanado. He esuchao’ también que pue’, los productos naturales también lo ha ayudado, ¿entiende?

**ENTREVISTADOR: Y estos tiempos que estamos viviendo, ¿de qué manera, si alguna, estos tiempos que vivimos influyen en la decisión de dejar de tomar los medicamentos?**

Participante masculino 3: Como le digo, missy…como hay diferentes…gente con capacidades diferentes, hay personas que tal vez, por su salud mental o por cualquier situación que esté pasando por su mente, puedan involucrarse en todo lo que está pasando hoy en dia en el mundo. Pue’, pueden…muchas personas desisten de seguir viviendo y una donde desisten tienen la enfermedad…entiende…y también por las cosas que escuchan de que “ay, posiblemente los fondos que vayan adelante son pa’ los pacientes de cáncer, pa’ los paciente de SIDA, etc.” Todas esas cosas lo van influyendo y eso tal vez puede ser que llene la copa y desistan de seguir tomando los medicamentos hasta que Dios mande y yo le aseguro que hay casos que se están pasando así.

**ENTREVISTADOR: Que se frustran…quizás…okay. Cuando en el pasado…cuando tú dejaste de tomarte los medicamentos….verdad, en el pasado, ¿qué te ayudo a volver a tomarte los medicamentos? ¿Qué fue lo que te ayudó a volver otra vez al tratamiento?**

Participante masculino 3: Mire, yo le voy a ser sincero. A mí lo que me ha ayudado pa’ yo volver al tratamiento es el compromiso que yo he hecho con mi vida de yo seguir hacia delante, sae’ no detenerme simplemente porque estoy…paciente de HIV. Porque yo puedo vivir como otra persona, común y corriente. Primero que nada, a lo mejor no entiende eso…primero que nada yo creo mucho en Dios, antes de los medicamentos. Porque los medicamentos me funcionan si Dios lo permite. Porque ahora mismo ustedes están constituidas en este lugar en este momento porque Dios lo ha permitido. Están haciendo esos estudios porque Dios se lo ha permitido. Pero sinceramente, le digo claramente…lo que me ha motivado a mí en…y me ha ayudado a yo seguir los medicamentos es esa fuerza que he recibido para seguir viviendo. De lo contrario…sae’…y estuviera en…mirando atrás…no me estaría tomando.

**ENTREVISTADOR: Y algo más que te haya ayudado…además de esa fuerza de Dios…**

Participante masculino 3: Pue’, las personas que hoy en dia tienen el caso mío. El manejador de caso, el internista, los enfermeros…

**ENTREVISTADOR: ¿Y cómo fue que llegaste otra vez a tomarte los medicamentos?**

Participante masculino 3: Pues llegue a Hogar CREA y ellos rápido…

**ENTREVISTADOR: ¿Tú voluntariamente llegaste…?**

Participante masculino 3: Sí, estoy voluntario aquí. Rápido que llegue a Hogar CREA, el director del título fue que la atendió a ustedes pue’ me motivó y dio órdenes de que se me resolviera todo lo mío lo antes posible pa’ yo volver a recibir mi tratamiento. Pue’ yo entre…y Dios ha puesto en mi camino…

**ENTREVISTADOR: ¿Y cuándo tomaste esa decisión de que ibas a venir aquí, lo que te ayudo fue…lo que te ayudo a tomar esa decisión “yo tengo que salir de esto, yo voy a buscar ayuda”…?**

Participante masculino 3: Sí, missy, porque ya me estaba apretando la preocupación de que la situación del vicio y ya mi salud no me estaba sintiendo igual. Esos son dos cosas…dos problemas. Esta la enfermedad de la adición y la enfermedad física de uno. Ya yo combinaba esas dos enfermedades, ya me está sintiendo desgastado. Y eso que ya como que me estaba preocupando…me estaba preocupando y también por la vida que estaba pasando no es la…ni se la deseo a un peor enemigo mío…a un ser humano. Y se…a salir de eso.

**ENTREVISTADOR: ¿Y qué le dirías a una persona que está pensando en dejar de tomar los medicamentos?**

Participante masculino 3: Que no lo haga.

**ENTREVISTADOR: Muy bien. Directo al grano.**

Participante masculino 3: Sí, missy. Que no lo haga. Que no lo haga porque sinceramente y lamentablemente todo el mundo no corre con la misma suerte. Hay personas hoy en dia con la condición de HIV que han empezado a tomar los medicamentos y las cosas les han ido demasiado de bien, y por sentirse bien lo han dejado y lamentablemente han decaído nuevamente y han fallecido. Por eso le diría que no lo haga…sae’ de dejar de tomarse los medicamentos ni dejar de ir al médico y tomarse el tratamiento.

**ENTREVISTADOR: ¿Has tenido oportunidad de hablar con personas que han dejado de tomar sus medicamentos? ¿Has hablado…?**

Participante masculino 3: No. No he tenido la oportunidad. Pero uno de los propósitos que Dios tiene conmigo es en ese aspecto. Después que yo bregue conmigo y tenga pa’ los demás unas cosas que Dios tiene conmigo respecto a esto.

**ENTREVISTADOR: ¿Y cuando tengas la oportunidad de…?**

Participante masculino 3: Lo voy a hacer con mucho gusto.

**ENTREVISTADOR: ¿Y qué le diría a las personas? ¿Qué le recomendarías?**

Participante masculino 3: Le recomendaría que vayan a un internista, a un nutricionista, que tomen los medicamentos adecuadamente…una buena dieta. Que se cuiden, que hagan ejercicio y sobre todas las cosas que le pidan mucho a Dios que lo levante y que de ánimo.

**ENTREVISTADOR: ¿Algo más que le recomendarías a ellos?**

Participante masculino 3: Pue’ que se cuiden mucho.

**ENTREVISTADOR: Cuando escuchas la frase “adherencia o cumplimiento con las recomendaciones médicas”, ¿con qué lo relacionas?**

Participante masculino 3: ¿Me puede hacer la pregunta otra vez?

**ENTREVISTADOR: Sí. Cuando escuchas la frase “adherencia o cumplimiento con las recomendaciones médicas”, ¿con qué relacionas esa frase? Cumplimiento de las recomendaciones médicas.**

Participante masculino 3: Adherencia…¿que viene siendo?

**ENTREVISTADOR: Que…es cumplimiento. Cumplir, como…adherencia viene siendo tu cumplir con…**

Participante masculino 3: Bueno si estamos hablando de…lo que ordena el medico a uno, ¿verdad?

**ENTREVISTADOR: Aja**

Participante masculino 3: ¿Que yo le diría a…?

**ENTREVISTADOR: ¿Qué tu…cuando tu escuchas eso…qué significa para ti eso?**

Participante masculino 3: Pue’, que siga las instrucciones que un doctor me lo dice. Que lleve todo a cabo, las dietas, tu sae’, que haga ejercicio, que me cuide, etc. Que no vuelva a caer otra vez en el vicio de las drogas, porque un dia puede ser que vuelva y no vuelva a levantarme ma’…y esas cosas.

**ENTREVISTADOR: Y además de tomarte el medicamento, ¿qué cosas te recomienda tu medico? ¿Todas esas que me dijiste?**

Participante masculino 3: Sí. Que tenga una buena dieta y haga ejercicio.

**ENTREVISTADOR: Te recomienda… ¿hay alguna recomendación de esas que te hace el doctor que se te hace difícil seguir?**

Participante masculino 3: De momento no.

**ENTREVISTADOR: Y examen médico, te recomienda el doctor, que te hagas exámenes médicos?**

Participante masculino 3: Cada 6 meses.

**ENTREVISTADOR: ¿Cada 6 meses? ¿Qué exámenes el medico te recomienda?**

Participante masculino 3: Los C4, carga viral, tuberculosis…este…los de pecho. Psicólogo... y esas cosas.

**ENTREVISTADOR: ¿Y lo haces? O hay alguno que no te los has hecho…**

Participante masculino 3: No.

**ENTREVISTADOR: Orita hablamos de uno, verdad, que no te habías…**

Participante masculino 3: No, gracias a Dios, todos están…como estoy empezando otra vez, ya estoy en eso. Ya lo que me falta a mi así es vacunarme. Ya me vacune con la influenza, que me mando el médico. Pero la vacuna de Hepatitis B, me faltan todavía 2. Ahora voy a coger si Dios quiere un tratamiento en la Escuela de Medicina para la condición del hígado. To’as esas cosas me dice el médico.

**ENTREVISTADOR: ¿Ya tienes cita para ese tratamiento?**

Participante masculino 3: Todavía.

**ENTREVISTADOR: ¿Todavía? ¿Y qué…por qué no te han dado cita?**

Participante masculino 3: Porque…tengo que llegar a la Escuela de Medicina.

**ENTREVISTADOR: AH, que tienes que ir hasta allá. ¿Y tienes quien te lleve allá?**

Participante masculino 3: Aquí.

**ENTREVISTADOR: ¿Aquí te llevan? ¿Cuándo te lo dieron, esa recomendación? Ese referido…**

Participante masculino 3: Desde que me diagnosticaron hepatitis C.

**ENTREVISTADOR: Ah, okay. ¿Hace cuánto…ahora…cuándo te lo diagnosticaron?**

Participante masculino 3: Ahora, me lo dieron…noviembre 17, por ahí.

**ENTREVISTADOR: Ah, hace poquito.**

Participante masculino 3: Sí.

**ENTREVISTADOR: Y hay algún impedimento para…porque todavía no tienes la cita, ¿verdad?**

Participante masculino 3: No, no he ido todavía.

**ENTREVISTADOR: Ah, que no has ido. Por alguna razón se te ha hecho difícil llegar a la universidad…llamar o hacer una cita…**

Participante masculino 3: No, lo que pasa es que como estoy enfocado todavía en lo de la condición de HIV, para luego pue’…entonces quisiera ir con laboratorio de las enzimas. Aunque yo sé que allá me lo sacan, por lo menos ir adelantando algo.

**ENTREVISTADOR: Okay, así que estas esperando terminar con algunas cosas primero para entonces ir a la cita. Alguna cita médica, verdad, que se te haya hecho difícil ir y hayas faltado, a citas médicas…ahora o antes en el algún momento.**

Participante masculino 3: No.

**ENTREVISTADOR: Siempre vas a tus citas médicas?**

Participante masculino 3: Sí, desde que estoy consciente…

**ENTREVISTADOR: Estas entonces rehabilitado.**

Participante masculino 3: Sí.

**ENTREVISTADOR: Cuando estabas en el vicio, me imagino que no…**

Participante masculino 3: No fallaba…no…como le digo. No iba a ninguna.

**ENTREVISTADOR: Hay algo más que tú quieras añadir sobre el tema, que no hemos hablado y creas que es importante que nosotros sepamos de esto.**

Participante masculino 3: Yo…el único comentario que yo diría es que personas así como usted deberían existir más, tu sae’, de que se preocupen por hacer estudios de esa magnitud para que…a ver que se puede resolver con esos estudios, para por lo menos echar un poco más hacia delante la población del VIH. De que pue’, personas como usté’, no se…como le digo…no se cohíban en hacer estudios así, porque son…como le digo…son beneficiosos y saludables para la población del VIH. Y mientras más se estudie y más se sepa de la condición del VIH de los medicamentos, más información y menos ignorancia de esa enfermedad va a mejorar el país, missy. Eso es lo que…el único comentario que yo…

**ENTREVISTADOR: Gracias, gracias. Y nosotros, de verdad, con mucho gusto y con mucho deseo de ayudar a las personas que pasan por la condición, tenemos el deseo de ayudarlos. Por eso estamos haciendo estos estudios. Y en la manera que podamos mejorar los servicios aquí vamos a estar, buscando esas maneras. Y en la manera que ustedes nos ayuden a mejorar, también. Bienvenido sea.**

Participante masculino 3: Yo siempre voy a estar dispuesto para lo que ustedes deseen. Haigan fondos o no haigan fondos. Ustedes vienen y buscan a…y yo siempre voy a estar dispuesto.

**ENTREVISTADOR: Gracias. Y si no hay algo más que añadir, pues ya terminado…con esto ya terminamos la entrevista.**

ENTREVISTA # 4

ENTREVISTADOR: ENTREVISTA INDIVIDUAL CUATRO, FASE UNO. OK EH PARTICIPANTE SE LE EXPLICO, SE DISCUSTIO CON EL PARTICIPANTE EL CONSENTIMIENTO INFORMADO EH Y DIO SU CONSENTIMIENTO PARA PARTICIPAR DEL ESTUDIO. YA FIRMO EL CONSENTIMIENTO ASI QUE COMENZAMOS CON LA ENTREVISTA A PROFUNDIDAD.

ENTREVISTADOR: ¿DESDE CUANDO ESTAS TOMANDO LOS MEDICAMENTOS PARA EL VIH?

Paciente: ¿Desde que yo empecé?

ENTREVISTADOR: ¿DESDE CUANDO? ¿DESDE CUANDO TE LO ESTAS TOMANDO?

Paciente: Yo empecé como en el 2007

ENTREVISTADOR: DESDE EL 2007, EH ¿Y EMPEZASTE A TOMAR LOS MEDICAMENTOS CUANDO TE DIERON EL DIAGNOSTICO O PASO A ALGUN TIEMPO?

Paciente: Rápido yo vine y me hicieron pruebas y me dieron los medicamentos

ENTREVISTADOR: Y CUANDO TE HICIERON LAS PRUEBAS, ¿CUANTO TIEMPO PASO DESDE QUE TE HICIERON LAS PRUEBAS Y TE EMPEZASTE A TOMAR LA PRIMERA PASTILLA?

Paciente: como un mes

ENTREVISTADOR: OK ESE MES FUE POR, ¿TARDO ESE MES POR ALGUNAS RAZONES ESPECIFICAS?

Paciente: aja por las pruebas y eso

ENTREVISTADOR: AH OK Y DON.. AQUI ¿TE TRATABAN AQUI?

Paciente: si

ENTREVISTADOR: ¿TENÍAS LOS MEDICAMENTOS DISPONIBLES CUANDO EMPEZASTE?

Paciente: no lo mandaron a buscar

ENTREVISTADOR: LO MANDARON A BUSCAR, TUVISTE QUE ESPERAR MUCHOS POR ELLOS

Paciente: como diez días

ENTREVISTADOR: AH OK Y ¿LEES LA INFORMACION QUE TE EXPLICA LOS EFECTOS SEGUNDARIOS DEL MEDICAMENTO?, CUANDO TE DAN ESA INFORMACION ¿LA LEES?

Paciente: a veces

ENTREVISTADOR: ¿QUE PREFIERES QUE TE LA DIGAN O QUE TE LA LEAN?

Paciente: que la digan

ENTREVISTADOR: QUE TE LA DIGAN OK Y ¿CONOCES ALGUNOS DE ESOS EFECTOS SEGUNDARIOS?

Paciente: no

ENTREVISTADOR: ¿TE ACUERDAS DE ALGUNO QUE TE HAYAN DICHO?

Paciente: no, no me acuerdo

ENTREVISTADOR: ¿QUE PIENSAS SOBRE LOS MEDICAMENTOS PARA TRATAR EL VIH QUE PIENSAS DE ELLOS QUE TE VIENE A LA MENTE?

Paciente: pues que son buenos

ENTREVISTADOR: OK

Paciente: que eso es pa’ pa’ nuestra salud

ENTREVISTADOR: ¿Y QUE EXPECTATIVAS TIENES PARA EL TRATAMIENTO PARA EL VIH? ¿CUALES SON TUS EXPECTATIVAS?

Paciente: durar más de lo que pueda

ENTREVISTADOR: AH OK TIENES ALGUN NUMERO DE AOS QUE QUIERAS LOGRAR?

Paciente: no

ENTREVISTADOR: CUANDO ESCUCHAS COMENTARIOS DE OTRAS PERSONAS QUE DEJAN DE TOMAR SUS MEDICAMENTOS ¿QUE PIENSAS?

Paciente: a veces... porque yo tengo amigo que tienen eso ahora mismo el los dejo a veces yo se lo digo

ENTREVISTADOR: SI, ¿Y QUE PIENSAS DE ESO?

Paciente: eso mal pa’ el

ENTREVISTADOR: ¿Y QUE LE DICES A EL?

Paciente: que eso es pa’ su salud

ENTREVISTADOR: ¿Y QUE MÁS?

Paciente: y el no, no quiere venir a buscar, el los venía a buscar antes, porque no tenía transportación

ENTREVISTADOR: AH OK ¿POR ESO EL NO VIENE A BUSCARLOS?

Paciente: aja

ENTREVISTADOR: ESTE TU ... ¿EN ALGUN MOMENTO HAS PENSANDO EN DEJAR EL TRATAMIENTO PARA LA CONDICION?

Paciente: fíjate no

ENTREVISTADOR: PERO EN EH... ¿DEJASTE DE TOMARTELOS EN ALGUN MOMENTO VERDAD?

Paciente: si ahora me fui y vine otra vez

ENTREVISTADOR: CUANDO ESTUVISTE FUERA ¿PORQUE DEJASTE DE TOMARTELO?

Paciente: pero con to’ y eso allá en Estados Unidos yo entraba a una clínica

ENTREVISTADOR: ENTRABAS A LA CLINICA PERO NO TE TOMABAS LOS MEDICAMENTOS

Paciente: si allá rapidito también me dieron los medicamentos

ENTREVISTADOR: ¿CUANTO TIEMPO ESTUVISTE SIN TOMARTELO?

Paciente: estuve un mes

ENTREVISTADOR: AH UN MES

Paciente: después fui y brinque a lo que ande to’ los pasos

ENTREVISTADOR: AH OK, ¿ESA ERA LA UNICA VEZ? O EN ALGUNA OTRA OCASION

Paciente: si acá cuando llegue porque no tenía transportación para venir pa’ acá, estuve como dos meses

ENTREVISTADOR: ¿CUANTO TIEMPO ESTUVISTE SIN TOMARTELO CUANDO LLEGASTE?

Paciente: estuve como tres meses

ENTREVISTADOR: ESTE Y EM TU HABIAS PEDIDO TRANSPORTACION Y NO TE LA DABAN O ERA QUE NO SABIAS COMO…

Paciente: no, no sabía

ENTREVISTADOR: ¿NO SABIAS QUE PODIAN DARTE TRANSPORTACION? ¿AHORA MISMO TE DAN LA TRANSPORTACION?

Paciente: no, ahora mismo yo compré un carro y bajo con mi carro

ENTREVISTADOR: AH OK ¿ALGUNA OTRA RAZON ADEMAS DE LA TRANSPORTACION POR LO CUAL HAYAS DEJADO DE TOMARTELOS?

Paciente: no

ENTREVISTADOR: Y ¿DOSIS DE LOS MEDICAMENTOS?, SABES QUE A VECES HAY PERSONAS QUE SE PUEDAN OLVIDAR TOMARSE UNA DOSIS EN LA MANANA POR LA TARDE, AL MEDIO DIA

Paciente: no fíjate ahí la mujer siempre ya tenía su hora ya

ENTREVISTADOR: AH OK ¿SE LAS TOMAN JUNTOS?

Paciente: si

ENTREVISTADOR: ESTE A MUCHAS PERSONAS CON LA CONDICION SE LES HACE DIFICIL TOMARSE LAS PASTILLAS TODOS LOS DIAS POR DIFERENTES RAZONES QUE ESTAN DENTRO Y FUERA DEL CONTROL DE LA PERSONA...ESTE... AHORA TE VOY HACER ALGUNAS PREGUNTAS SOBRE LAS POSIBLES RAZONES PARA QUE EN ALGUN MOMENTO TU HAYAS DEJADO DE TOMARTE, VERDAD, EL MEDICAMENTO PARA EL VIH. EH AHORA YO QUIERO QUE TU PIENSES EN TI SIN INCLUIR A OTRAS PERSONAS SIN INCLUIR OTRAS CIRCUSNSTANCIAS NADA SOLAMENTE EN TI. ¿COMO TU TE DESCRIBES COMO PERSONA?

Paciente: soy bien tranquilo

ENTREVISTADOR: OK. ¿ALGO MÁS?

Paciente: no

ENTREVISTADOR: NOSOTROS SOMOS DE MUCHAS MANERAS

Paciente: si yo soy tranquilo. Todos se llevan conmigo fíjate muy bien

ENTREVISTADOR: ESO ES LAS COSAS POSITIVAS TUYAS VERDAD ¿Y QUE OTRAS COSAS POSITIVAS TU TIENES?

Paciente: …..

ENTREVISTADOR: ES DIFICIL VERDAD CUANDO UNO...OK Y TODOS TENEMOS NUESTRO LADO NEGATIVO, TU SABES LAS COSAS ASI NEGATIVAS QUE UNO TIENE, QUE UNO TIENE QUE MEJORAR ¿QUE COSAS?

Paciente: ay es difícil…a veces los revoluces

ENTREVISTADOR: A VECES ¿QUE?

Paciente: los revoluces y to’ eso uno bajo coraje y to’ eso.

ENTREVISTADOR: A VECES NOS DA CORAJE ESOS NOS PASA A TODOS ¿ALGO MAS?

Paciente: no

ENTREVISTADOR: AHORA... ¿CONSIDERAS QUE ALGUNAS DE ESAS CARACTERISTICAS O TUYAS O PENSAMIENTOS O CREENCIAS PERSONALES TUYAS VERDAD ESTE INFLUYO PARA QUE DEJARAS DE TOMAR MEDICAMENTOS?

Paciente: no

ENTREVISTADOR: ESTE O ALGUNAS AVECES UNA DE ESAS CARACTERISTICAS COMO CUANDO ME DIJISTE BAJO CORAJE UNO PIENSE “AY NO ME VOY A TOMAR LOS MEDICAMENTOS” “NO ME VOY A TOMAR NAH ESAS DOSIS”

Paciente: si a veces

ENTREVISTADOR: ¿ESO SI PASA? OK. ESTE CUANDO DEJASTE DE TOMAR LOS MEDICAMENTOS QUE TE FUISTE PARA ALLA FUERA... Y AHORA QUE ESTUVISTE LOS TRES MESES EN ALGUN MOMENTOS TU CREES PUES COMO PUSISTE DE EJEMPLO AVECES BAJO UN CORAJE “AY YO NO TENGO TRANSPORTACION”

Paciente: a veces a uno le da

ENTREVISTADOR: EL CORAJE ESTE EN ALGUN MOMENTO COMO “QUE MAL NO VOY A TOMAR NAH”

Paciente: desanima a uno

ENTREVISTADOR: ¿ALGO MAS ...QUE ...DE COSAS ASI DE COSAS QUE UNO SIENTE QUE TE DESANIMEN A NO TOMARTE LOS MEDICAMENTOS ALGUNA OTRA COSA QUE TE DESANIME A TI?

Paciente: a veces era el viaje pa’ acá

ENTREVISTADOR: AJA, ¿TIENES QUE VIAJAR?

Paciente: si a veces nosotros somos de Guayanilla

ENTREVISTADOR: AH OK ADEMAS DE LA TRANSPORACION ESTAN PASANDO COSAS EN TU VIDA QUE TAMBIEN ESAS COSAS SE JUNTABAN Y TE DESANIMABA A DEJAR DE TOMARTE LOS MEDICAMENTOS

Paciente: ….. la pelea de la mujer si porque sufre de celos crónicos

ENTREVISTADOR: AH OK

Paciente: chacho eso pelea se muda hasta una vecina pal lao’ y pelea todos los días

ENTREVISTADOR: AH OK ¿Y ESO A VECES TE DESANIMA A TOMARTE LOS MEDICAMENTOS? ¿SI?

Paciente: ujum si porque a veces me descontrolo y lo que hago es que cojo calle y me voy pa’casa de los viejos.

ENTREVISTADOR: AH OK Y SI TE VA LAS DOSIS SE TE VAN. OK...Y ESTE ALGUNA OTRA PERSONA TE DESANIMA ALGUIEN DE TU FAMILIA QUE A VECES TENGA... QUE SE YO... UNA DISCUSION, UNA DIFERENCIA CON ALGUIEN DE TU FAMILIA Y QUE ESO TE DESANIME

Paciente: toda mi familia se lleva bien

ENTREVISTADOR: Y POR EJEMPLO AMISTADES

Paciente: a veces viene uno que siempre viene a buscar problema y eso

ENTREVISTADOR: Y CUANDO PASA ESO COMO QUE SE JUNTA CON LO DEL MEDICO Y DICES “AY… NO ME VOY A TOMAR LOS MEDICAMENTOS”

Paciente: no, pero muchacho después la mujer por la mañana y paff ya está con las pastillas.

ENTREVISTADOR: Y POR EJEMPLO QUIZAS PERSONAS QUE A LO MEJOR ELLOS SEAN LOS QUE TE DESANIMEN Q TE DIGAN “NO TOMES NAH ESO”

Paciente: fíjate no

ENTREVISTADOR: CUANDO A VECES COMO TE DIJE… ALGUNA VEZ COMO LOS MEDICOS TE HAN TRATADO... ALGUNA VEZ TE HAN DESANIMADO A QUE TE DEJES DE TOMARTE LOS MEDICAMENTOS?

Paciente: diantre no aquí tratan a uno bien

ENTREVISTADOR: AH OK O PERSONAL, ALGUN PERSONAL DE ENFERMERIA, DE ALGO…

Paciente: no

ENTREVISTADOR: ¿Y EN LA CLINICA QUE ESTUVISTE ALLA EN NUEVA JERSEY?

Paciente: no fíjate son chévere

ENTREVISTADOR: ESTE POR ALGUNA OTRA PERSONA PUES A LO MEJOR ESO TU SABES, QUE HAYA HECHO ALGUN COMENTARIO O ALGO Y ESO COMO QUE TE DESANIMA… COMO HAYA REACCIONADO LA PERSONA

Paciente: no

ENTREVISTADOR: EH AHORA YO QUIERO QUE TU PIENSES EN EN EL AMBIENTE DONDE TU TE DESENVUELVES. SI FUERAS A DESCRIBIRLO ¿COMO TU DESCRIBIRIAS ESE AMBIENETE DONDE TU DESENVUELVES? DONDE TU VIVES, DONDE TU ESTAS… ¿COMO TU LO DESCRIBES?

Paciente: fíjate es tranquilo

ENTREVISTADOR: ESTE LA COMUNIDAD DONDE VIVES

Paciente: si porque yo vivo frente a la orilla a la mar estamos todo el día en la mar así..

ENTREVISTADOR: OK EH ESE VERDAD A VECES HAY PERSONAS QUE EL AMBIENTE PUEDE INFLUIR O DESANIMAR COMO TU DICES A TOMARSE LOS MEDICAMENTOS ALGUNA … HAY ALGUNA... QUE EL AMBIENTE EN QUE TU DESENVUELVES YA SEA DONDE TU VIVE O TRABAJAS?

Paciente: tu sabes en mecánica por acá

ENTREVISTADOR: AH OK PUES TU SABES EN EL LUGAR DONDE TU TRABAJAS, JANGUEAS, POR EJEMPLO EN EL LUGAR DONDE RECIBE SERVICIO. ¿DE QUE MANERA VERDAD ESO TE INLFLUYE EN DESANIMARTE A TOMARTE LOS MEDICAMENTOS?

Paciente: tu sabes a veces estoy trabajando como que me quita el animo

ENTREVISTADOR: OK CUANDO ESTAS TRABAJANDO ¿QUE PASA AHI?

Paciente: como que pierdo fuerza y to’

ENTREVISTADOR: AJA

Paciente: a veces dejo el carro a mitad y cojo y me baño y me acuesto a dormir

ENTREVISTADOR: OK Y ¿TE TOMA LOS MEDICAMENTOS? ¿ALLI EN EL TRABAJO?

Paciente: si porque en el mismo casa que vivimos

ENTREVISTADOR: ¿Y TE LOS TOMAS? OK Y DONDE JANGUEAS POR EJEMPLO, A VECES... VERDAD... SI JANGUEAS PUES... PASA QUE SE TE HA OLVIDADO TOMARTE LA DOSIS

Paciente: a veces cuando voy pa’ la mar

ENTREVISTADOR: AH PORQUE... ¿TAMBIEN ERES PESCADOR? ¿Y DEJAS DE TOMARTE UNA DOSIS AHI?

Paciente: que salgo así a las millas

ENTREVISTADOR: AJA Y TE ¿VAS?

Paciente: aja

ENTREVISTADOR: Y POR EJEMPLO HA PASADO QUE AVECES POR ESTAR JANGUEANDO COMO UNO DICE POR AHÍ, YA SEA EN LA MAR, O TODO ESO ESTE... ¿HAS DEJADO DE TOMARTE LAS DOSIS COMPLETAS DE UN DIA POR EJEMPLO… ¿CUANTAS DOSIS TU TE TIENES QUE TOMAR?

Paciente: tu dices...

ENTREVISTADOR: ¿CUANTAS DOSIS TU TE TIENES QUE TOMAR?

Paciente: tres pastillas

ENTREVISTADOR: TRES PASTILLAS…

Paciente: diarias, si porque son dos con comidas y una sin…

ENTREVISTADOR: PERO ESAS PASTILLAS ¿TE LAS TOMAS UNA A LA VEZ O TE TOMAS UNAS PRIMERO?

Paciente: tú dices…

ENTREVISTADOR: TE LAS TOMAS UNA A UNA HORA Y OTRA A LA OTRA HORA

Paciente: me la tomo las dos con comida por la tarde y la que es sin comida por la mañana

ENTREVISTADOR: AH OK Y TE HA PASADO PORQUE A VECES ESTAS TODO EL DIA SIN TOMARTE LAS PASTILLAS PORQUE ESTAS HACIENDO ALGO TE VAS PA LA MAR …

Paciente: a veces cuando nos vamos pa’ pescar de un día pa’ otro

ENTREVISTADOR: AH OK ESTE ALGUN OTRO LUGAR COMO ESO QUE ESTAMOS HABLANDO CUANDO ESTAS MECANIANDO, CUANDO ESTAS EN LA MAR, EN OTRO LUGAR QUE CUANDO ESTAS AHI TE PASA ESO… QUE NO TE LA TOMASTE ...QUE SE TE OLVIDO QUE NO TE LA PUDISTE LLEVAR

Paciente: A veces así cuando estoy en casa la suegra se me olvida

ENTREVISTADOR: OK TE VAS Y…

Paciente: A veces estoy uff horas por allá y la mama mira y ya mismo

ENTREVISTADOR: OK ESTE EM TE IBA A PREGUNTAR ALGO DEJAME VER… EH ESTE… ¿HAY PERSONAS QUE SABEN DE TU CONDICION O NO LO SABE MUCHA GENTE?

Paciente: tú sabes lo sabe toda mi familia

ENTREVISTADOR: AH OK

Paciente: algunos amistades por allí.

ENTREVISTADOR: Y CUANDO ESTAS EN LUGARES ASI COMO... O SEA... ¿TU TE HAS TOMADO LAS PASTILLAS SOLO EN TU CASA O TE LAS HAS TOMADO CUANDO NO ESTAS EN TU CASA? COMO POR EJEMPLO SI VAS PA CASA DE LA SUEGRA SI VAS A PESCAR O…

Paciente: no en mi casa

ENTREVISTADOR: AH OK

Paciente: a veces mi hermano me pregunta si yo venía acá

ENTREVISTADOR: AH OK ELLOS TE CUIDAN ESO ES BUENO

Paciente: hasta la suegra a veces pelea con la mujer

ENTREVISTADOR: AJA OK. AHORA PIENSA, VERDAD, YO QUIERO QUE PIENSES EN PUERTO RICO ESTE LAS COSAS QUE PASAN AQUI EN PUERTO RICO EN LA ISLA. ¿QUE TU PIENSAS DE LO QUE PASA EN EL GOBIERNO? POR EJEMPLO, COSAS QUE PASAN EN EL GOBIERNO ACTUALMENTE. ¿QUE HAS VISTO QUE HAS ESCUCHADO?

Paciente: yo no se

ENTREVISTADOR: OK ¿Y DE LOS SISTEMAS DE LA SALUD DE AQUI, LA SALUD DE PUERTO RICO LOS SERVICIOS DE SALUD COMO LOS VE?

Paciente: bregan bien

ENTREVISTADOR: OK TU CREES VERDAD QUE DE ALGUNA MANERA ESO QUE TE ESTOY HABLANDO DE PUERTO RICO: EL GOBIERNO, LOS SISTEMAS DE SALUD, LA REFORMA, LOS PLANES MEDICOS, LAS CLINICAS Y TODO ESO... ESTE... LA SITUACION ECONOMICA DEL PAIS DE ALGUNA MANERA ¿TU CREES QUE DE ALGUNA MANERA ESAS COSAS QUE PASAN AQUI EN PUERTO RICO INFLUYEN EN LA DECISION DE ALGUNAS PERSONAS EN DESANIMARSE Y DEJAR DE TOMAR LOS MEDICAMENTOS?

Paciente: a veces

ENTREVISTADOR: EN QUE POR EJEMPLO QUE HAYAS ESCUCHADO QUE TU QUE

Paciente: como la noticia del nene

ENTREVISTADOR: ¿SI, CUAL?

Paciente: del que tiraron en el piso en estos días, es de un amiguito mío

ENTREVISTADOR: ¿DE VERDAD?

Paciente: hoy lo traen a guayanilla ...muchacho lo que nosotros supimos... el pai está grave en el hospital

ENTREVISTADOR: ¿EL ESTA GRAVE? ¿EL AMIGO TUYO?

Paciente: él trabaja con nosotros, él trabaja con mi mujer en los tomates. El me vio y me dijo… “en estos días me llevas a ver el nene”. Lo llevé a la casa cuando la mai le dio la noticia.

ENTREVISTADOR: AH OK Y POR EJEMPLO ESTA PERSONA BUENO… A VECES… ¿COMO TU CREES QUE ESO PUEDE INFLUIR A UNA PERSONA A DEJAR DE TOMARSE LOS MEDICAMENTOS?

Paciente: no sé cómo te digo la misma emoción y eso

ENTREVISTADOR: AJA QUE LE AFECTA EMOCIONALMENTE

Paciente: ujum como el casi esta todos los días en casa al coger esa noticia de cantazo

ENTREVISTADOR: AJA Y ¿TE HA AFECTADO A TI?

Paciente: entonces a veces a uno le da pena y eso. Se le olvida a uno las pastillas y to’

ENTREVISTADOR: OH OK ESTE Y POR EJEMPLO COSAS ASI COMO… ¿HAY OTRA COSA MAS POR EJEMPLO DE ESTO DE LAS COSAS QUE PASAN EN PUERTO RICO LOS SERVICIOS DE SALUD, LOS PLANES MEDICOS, SITUACION ECONOMICA? ALGO COMO QUE ASI COMO EL EJEMPLO QUE TU ME DISTE AHORA QUE INFLUYE DE ALGUNA MANERA DIRECTA O INDIRECTAMENTE PARA QUE LAS PERSONAS DEJEN DE TOMARSE LOS MEDICAMENTOS PARA EL VIH.

Paciente: ahí no me acuerdo

ENTREVISTADOR: NO TE ACUERDAS, ESTA BIEN. ESTE AHORA YO QUIERO QUE PIENSES COMO COSTUMBRES, CREENCIAS DE NUESTRA SOCIEDAD ESTE ¿COMO NOSOTROS LOS PUERTORRIQUENOS... ESTE ¿COMO TU DESCRIBES A NOSOTROS MISMOS COMO PUERTORRIQUENOS?

Paciente: ¿nosotros?

ENTREVISTADOR: ¿COMO SOMOS LOS BORICUAS? ¿COMO NOS VEN A NOSOTROS LOS BORICUAS? QUE COSAS POSITIVAS TENEMOS NOSOTROS LOS PUERTORRIQUENOS? ¿QUE HACEMOS BIEN?

Paciente: los puertorriqueños le gusta trabajar

ENTREVISTADOR: AJA SOMOS TRABAJADORES ¿QUE MAS?

Paciente: no me “arecuerdo”

ENTREVISTADOR: ¿Y QUE COSAS NEGATIVAS HACEMOS LOS PUERTORRIQUENOS? ...NOSOTROS

Paciente: pelea tanta matanza y to’

ENTREVISTADOR: LA CRIMINALIDAD

Paciente: ujum

ENTREVISTADOR: ¿QUE MÁS?

Paciente: no me “arecuerdo” bien

ENTREVISTADOR: Y ESAS SON LAS COSTUMBRES Y CREENCIAS DE NOSOTROS ¿DE QUE MANERA SI ALGUNA ESAS COSAS QUE SE ESTAN MENCIONANDO VERDAD LAS COSTUMBRES Y CREENCIAS INFLUYEN EN LA DECISION DE ALGUIEN DE ALGUNA PERSONA VERDAD EN DESANIMARSE A DEJAR DE TOMARSE LOS MEDICAMENTOS?

Paciente: para mí lo de este chamaquito na’ mas

ENTREVISTADOR: SI LO QUE PASO CON EL NENE

Paciente: aja

ENTREVISTADOR: ¿Y ALGUNA VEZ TE HAS SENTIDO DESCRIMINADO POR TENER LA CONDICION? ¿HAY GENTE QUE TE HAYA RECHAZADO? ¿GENTE QUE TRATO DIFERENTE POR TENER LA CONDICION?

Paciente: no fíjate no

ENTREVISTADOR: EH ¿HAS ESCUCHADO O CONOCES DE ALGUIEN QUE SE HA SENTIDO RECHAZADO POR OTRAS PERSONAS POR TENER LA CONDICION?

Paciente: el amigo mío

ENTREVISTADOR: SI Y ESTE Y EN ESE CASO DE ESE AMIGO TUYO ¿TU CREES QUE ESE DISCRIMEN O ESE RECHAZO HAYA INFLUENCIADO EN QUE SE DESANIMEN DE TOMARSE LOS MEDICAMENTOS O PIENSEN EN DEJAR DE TOMARSELOS?

Paciente: pero que este los vecinos y eso le gritan a él y to’

ENTREVISTADOR: ¿LE GRITAN?

Paciente: ujum

ENTREVISTADOR: Y EL ALGUNA VEZ TE HA DICHO COMO QUE SE SIENTEN TAN CANSADO

Paciente: a veces vete a la clínica a ver “ah es que ya no”

ENTREVISTADOR: ESTE Y POR EJEMPLO LA RELIGION Y LAS CREENCIAS RELIGIOSAS ESTE ¿CREES QUE ALGUNAS CREENCIAS RELIGIOSAS DE ALGUNA MANERA PUEDEN INFLUIR ASI PARA QUE SE DESANIMEN DE TOMAR LOS MEDICAMENTOS?

Paciente: no

ENTREVISTADOR: AH OK. ESTE Y LA SITUACION SOCIAL ESTE ¿TU CREES QUE INFLUYE EN QUE ALGUIEN SE DEJE DE TOMAR LOS MEDICAMENTOS? LO QUE SOCIALMENTE, VERDAD, LA SITUACION SOCIAL

Paciente: no

ENTREVISTADOR: ¿NO?

Paciente: no

ENTREVISTADOR: ¿ALGUNA OTRA COSA QUE INFLUYE?

Paciente: hasta ahora

ENTREVISTADOR: OK PIENSA AHORA YO QUIERO QUE PIENSES EN LA EPOCA QUE ESTAMOS VIVIENDO VERDAD EN ESTOS TIEMPOS QUE ESTAMOS VIVIENDO AHORA QUE SON DIFERENTES A LOS TIEMPOS VERDAD... ¿DE QUE MANERA SI ALGUNA ESTOS TIEMPOS EN QUE VIVIMOS INFLUYE EN LA DECISION DE ALGUNAS PERSONAS DE DEJAR DE TOMARSE LOS MEDICAMENTOS PARA EL VIH?

Paciente: ¿cómo estamos viviendo?

ENTREVISTADOR: LOS TIEMPOS DE AHORA, LA EPOCA QUE ESTAMOS VIVIENDO AHORA

Paciente: tantas matanza en el tiempo de antes no

ENTREVISTADOR: UJUM ¿Y ESO TU CREES QUE INFLUYE?

Paciente: puede ser que influye

ENTREVISTADOR: ¿SI?

Paciente: si

ENTREVISTADOR: ¿DE QUE MANERA ESO PUEDE INFLUIR EN TI?

Paciente: por tantas muerte y to’ eso uno dice “contra como era ante y ahora... ya uno no puede salir ni pa la calle ni na’”

ENTREVISTADOR: AJA ¿Y EN ALGÚN MOMENTO TE HA DESANIMADO UN POQUITO O ALGO A DEJAR DE TOMAR LOS MEDICAMENTOS?

Paciente: si a veces uno....umm

ENTREVISTADOR: ¿Y TE TOMAS LOS MEDICAMENTOS?

Paciente: si

ENTREVISTADOR: AH OK ESTE QUE LE DIRIAS CUANDO EN EL PASADO CUANDO EN ESAS DOS VECES QUE DEJASTE DE TOMARTE LOS MEDICAMENTOS O CUANDO TE HAS DEJADO DE TOMAR LAS DOSIS QUE TE AYUDO A VOLVER A TOMARTELOS

Paciente: pa’ mi fue la familia y la mujer

ENTREVISTADOR: OK ESTE Y ¿ALGO MAS O ALGUIEN MAS QUE TE HAYA AYUDADO O ALGO QUE TE HAYA AYUDADO A TOMARTELO ADEMAS DE LA FAMILIA Y DE TU MUJER?

Paciente: no los mismo amigos míos y eso..

ENTREVISTADOR: ¿LAS AMISTADES? ¿QUE TE DECIAN?

Paciente: que fuera a la clínica y eso

ENTREVISTADOR: AH OK. ¿QUE LE DIRIAS A UNA PERSONA QUE ESTA PENSANDO EN DEJAR DE TOMARSE LOS MEDICAMENTOS?

Paciente: que no dejaría los medicamentos porque eso es pa’ su salud

ENTREVISTADOR: UJUM ¿QUE MAS LE DIRIAS?

Paciente: que lo medicamentos es pa’ su salud

ENTREVISTADOR: OK Y ¿HAS TENIDO LA OPORTUNIDAD DE HABLAR CON PERSONAS QUE LO HAN DEJADO LOS MEDICAMENTOS, ME DIJISTE DE TU AMIGO VERDAD?

Paciente: el amigo mío

ENTREVISTADOR: EL AMIGO TUYO, ¿ALGUIEN MAS ADEMAS DE TU AMIGO?

Paciente: pues lo que pasa que allí no

ENTREVISTADOR: AJA

Paciente: allí son como culto los que los tienen

ENTREVISTADOR: OK ¿EN DONDE?

Paciente: en Guayanilla

ENTREVISTADOR: ¿Y QUE LE HAS RECOMENDADO A TU AMIGO?

Paciente: yo le dicho que si quiere yo lo traigo y todo.

ENTREVISTADOR: PERO NO...

Paciente: no quiere

ENTREVISTADOR: ¿Y PORQUE NO SE LOS QUIERE TOMAR? QUE..

Paciente: lo que pasa eh que como toa’ la gente le grita y to’ eso

ENTREVISTADOR: AH

Paciente: ps ya el como que ya no...

ENTREVISTADOR: ¿NO? ¿SE QUIERE MORIR?

Paciente: será

ENTREVISTADOR: ¿Y EN CUANTO TIEMPO LLEVA EL CON LA CONDICION?

Paciente: fíjate ya él es mayor como el ahora pa’ el lo que hace es tu sabe pa’ que la gente no le grite ni nah se queda en casa de una hermana y de ahí mismo como el tiene animales se queda metío en el monte por allá

ENTREVISTADOR: ¿Y A TI NO TE GRITAN?

Paciente: no

ENTREVISTADOR: ¿Y ESTE A TU ESPOSA?

Paciente: no

ENTREVISTADOR: OK CUANDO TU ESCUCHAS LA FRASE CUMPLIMIENTO DE RECOMENDACIONES MEDICAS ¿CON QUE LO ASOCIAS? CUMPLIMIENTO DE RECOMENDACIONES MEDICAS.... ¿LO HABIAS ESCUCHADO ANTES?

Paciente: no

ENTREVISTADOR: ESTE Y ¿SABES LO QUE SON RECOMENDACIONES MEDICAS?

Paciente: no

ENTREVISTADOR: CUANDO EL MEDICO TE DICE, EL MEDICO VERDAD TE... ESTE... TE DICE QUE TE TOME LOS MEDICAMENTOS VERDAD PERO EN ADICION TE RECOMIENDA QUE HAGAS OTRAS COSAS. ESTE... QUE TE HAGA UNOS EXAMENES QUE ESTE...

Paciente: si si muchas pruebas

ENTREVISTADOR: AJA ESO ADEMAS DE RECOMENDARTE QUE TE TOME LOS MEDICAMENTOS QUE OTRAS RECOMENDACIONES TE HAN DADO LOS MEDICOS

Paciente: ahora mismo me dieron para el dentista me dieron una ahí para a el mismo Guayanilla en estos días

ENTREVISTADOR: AJA ¿PARA DONDE?

Paciente: pa’ sacarme sangre y todas la pruebas

ENTREVISTADOR: Y POR EJEMPLO ¿TE DAN REFERIDO PARA OTRAS COSAS? EN LO PASADO ¿TE HAN DADO REFERIDO PARA HACERTE OTRAS ANALISIS PARA VERTE OTROS MEDICOS O ALGO ASI?

Paciente: no

ENTREVISTADOR: SABES QUE AHORA MISMO EL DENTISTA

Paciente: aja

ENTREVISTADOR: Y ADEMAS DE ESA RECOMENDACIÓN SE TE HACE DIFICIL SEGUIRLAS TU SABES SI EL TE LA DA TU PIENSAS “AY DIOS MIO ESTO...

Paciente: antes antes si pero ya no

ENTREVISTADOR: AH OK Y ANTES ¿QUE RECOMENDACIONES SE TE HACIAN DIFICIL ANTES?

Paciente: el dentista porque yo le tenía miedo a las agujas y to’

ENTREVISTADOR: Y ¿QUE HACIAS? ¿NO IBAS?

Paciente: aja

ENTREVISTADOR: Y ¿CUANTO TIEMPO ESTUVISTE SIN IR AL DENTISTA?

Paciente: uff hasta que caí preso

ENTREVISTADOR: ¿SI? ¿Y CUANTO TIEMPO FUE ESO?

Paciente: eso fue en el 1999

ENTREVISTADOR: AH OK Y AHÍ ENTONCES CUANDO CAISTE PRESO TUVISTE QUE IR AL DENTISTA PORQUE NO TE PODIAS ESCAPAR PARA IR AL DENTISTA ¿VERDAD?

Paciente: aja

ENTREVISTADOR: ESTE ¿ALGUNA OTRA?... EXAMENES MEDICOS QUE TE RECOMIENDAN EL DOCTOR ME DIJISTE ¿QUE TE RECOMENDO AHORA QUE?

Paciente: hacer esas pruebas

ENTREVISTADOR: ¿LAS PRUEBAS DE SANGRE?

Paciente: aja

ENTREVISTADOR: PERO AHÍ HAY AGUJAS ¿TE LAS HACES?

Paciente: me tienes que coger de una sola vez

ENTREVISTADOR: ¿Y HAY VECES QUE NO TE LAS HACES O A VECES TE TARDAS MUCHO EN HACERTELAS?

Paciente: rapidito que “desto” dale

ENTREVISTADOR: AH OK Y ANTES TE TARDABAS

Paciente: jum imagínate la mai’ mía desde pequeño no me llevaba

ENTREVISTADOR: AH

Paciente: hacia show en los hospitales

ENTREVISTADOR: AH OK SI ...TU SABES QUE MIENTRAS MAS ESTE MIEDO LE TENGAS MAS TE DUELE

Paciente: ujum

ENTREVISTADOR: ASI QUE TIENES QUE RELAJARTE CON ESO. ESTE Y LAS CITAS MEDICAS ESTE ¿VAS A LAS CITAS MEDICAS? ¿A TODAS O ALGUNAS QUE SE TE HACE DIFICIL VENIR?

Paciente: tu sabes a veces si no puedo tu sabes si me dan una por la mañana y no puedo llegar lo que hago es que llamo rápido

ENTREVISTADOR: AH OK Y ¿QUE COSAS INFLUYEN POR EJEMPLO PARA QUE AVECES NO PUEDAS VENIR A LAS CITAS MEDICAS O QUE TENGAS QUE FALTAR A UNA CITA MEDICA?

Paciente: A veces que el carro esta dañado o yo estoy afuera del barrio y eso

ENTREVISTADOR: AJA

Paciente: pues rápido llamo y eso

ENTREVISTADOR: ESTAS EN OTRAS COSAS AFUERA..

Paciente: ujum

ENTREVISTADOR: AH OK. ESTE ¿HAY ALGO MAS QUE QUIERAS ANADIR SOBRE EL TEMA QUE HEMOS ESTADO HABLANDO?. QUE A LO MEJOR NO TE HE PREGUNTADO QUE SEA IMPORTANTE.

Paciente: no

ENTREVISTADOR: PUES ENTONCES FINALIZAMOS LA ENTREVISTA NUMERO CUATRO.

Transcripción de entrevista #5

ENTREVISTADOR: YA SE DISCUTIÓ CON EN PARTICIPANTE EL CONCENTIMIENTO INFORMADO. SE ACLARARON LAS DUDAS Y PREGUNTAS. PARTICIPANTE YA FIRMÓ SU CONSENTIMIENTO. ASI QUE COMENZAMOS CON EL PROCESO DE ENTREVISTA A PROFUNDIDAD

ENTREVISTADOR: ¿DESDE CUÁNDO ESTAS TOMANDO LOS MEDICAMENTOS PARA EL VIH?

Participante: Pues a mí me los recetaron en el 2005 (OK), digo, en la cárcel.

ENTREVISTADOR: SI

Participante: Cuando estaba en la cárcel

ENTREVISTADOR: OK

Participante: Y cuando… yo no conozc…. Quien me lo pegó fue mi esposo y yo lo conocí a él cuando tenía 22 años

DRA. CASTOR: OK

Participante: Yo sé qué hace bastante tiempo.

ENTREVISTADOR: OK, EN EL 2005 FUE QUE TE LO DETECTARON…

Participante: Me lo detectaron.

ENTREVISTADOR: Y AHÍ, EN ESE, Y CUANDO TE LO DETECTARON EN EL 2005 EMPEZASTE A TOMARTE LOS MEDICAMENTOS, ESTE, RÁPIDO, O PASÓ ALGÚN TIEMPO.

Participante: No, pasó algún tiempo por que quien me llevó fue él.

ENTREVISTADOR: AH OK

Participante: Porque yo tuve que esperar que él saliera de la cárcel, porque estaba preso.

ENTREVISTADOR: AH OK. Y EN EL 2005 TE LO DETECTARON CUANDO ESTABAS EN LA CÁRCEL.

Participante: Aja.

ENTREVISTADOR: ENTONCES, DESPUÉS QUE TE LO DETECTARON, QUE TE HICIERON LA PRUEBA, VERDAD Y TE LO DETECTARON, TE DIERON LOS MEDICAMENTOS EN LA CÁRCEL O PASÓ ALGÚN TIEMPO.

Participante: No, pasó algún tiempo porque en la cárcel no me lo dieron.

ENTREVISTADOR: AH ¿NO TE LO DIERON?

Participante: No, no me dieron los medicamentos

ENTREVISTADOR: NO. ¿Y, PORQUE NO TE LO DIERON?

Participante: Pues no duré mucho. Lo que duré fue una semana na más, (OK) ahi.

ENTREVISTADOR: AH, BUENO, OK. Y ENTONCES, ESTE, O SEA QUE EN UNA SEMANA SALISTE Y CUANDO SALISTE, QUE PASÓ QUE NO TUVISTE LOS MEDICAMENTOS.

Participante: Porque mi esposo estaba con (no entendí) era el único que sabía llegar aquí. Sabes, aquí.

DRA. CASTRO. AH OK. ¿Y CUÁNTO TIEMPO DESDE QUE TE LO DETECTARON QUE SALISTE DE LA CÁRCEL Y ENTONSES PUDISTE POR FIN TOMARTE LOS MEDICAMENTOS? ¿CUÁNTO TIEMPO PASÓ MÁS O MENOS?

Participante: Bueno, mi esposo salió a los 3 años

ENTREVISTADOR: ¿ESTUVISTE TRES AÑOS SIN TOMARTE LOS MEDICAMENTOS?

Participante: Tres años.

DRA. CASTO: OK. EN ESE PERIODO DE TRES AÑOS, FUISTE, VINISTE A LAS CLÍNICAS, TE DISTE ALGÚN TRATAMIENTO O SIMPLEMENTE NO FUISTE AL MÉDICO NI NADA.

Participante: Si, vinimos aquí a la clínica (AJA) los dos.

ENTREVISTADOR: ¿CUÁNDO ÉL SALIÓ? (aja) ¿PERO, ANTES DE QUE ÉL SALIERA?

Participante: antes de que él saliera, no.

DRA CASTRO: ANTES DE QUE ÉL SALIERA TU ESTUVISTE FUERA (SI)

Participante: Yo no estaba, yo estaba fuera de la cárcel.

DRA CASTRO: AH OK ¿Y NO VENIAS A LA CLÍNICA?

Participante: No porque yo no sabía (OK). El que sabía era él.

ENTREVISTADOR: ¿TU NO SABÍAS QUE TENÍAS LA CONDICIÓN O NO SABÍAS COMO LLEGAR A…?

Participante: Yo sabía que yo tenía mi condición pero yo no sabía venir pa aquí.

DRA CASTRO: AH OK

Participante: El que sabía era él, él fue que trajo la primera mujer (AH OK). Que fue quien se las pegó a él (OK)… (no entendí lo próximo 3:01)

ENTREVISTADOR: O SEA, QUE ESTUVISTE TRES AÑOS SIN EL SIN (exacto)… ESTE, EH. ¿QUÉ PIENSAS SOBRE LOS MEDICAMENTOS PARA TRATAR EL VIH?

Participante: Que son, son buenos, sae am, ayudan. Ayudan un montón, eso sí.

ENTREVISTADOR: EH. ¿QUÉ ESPERAS DEL TRATAMIENTO PARA EL VIH? ¿QUÉ EXPECTATIVAS TIENES, QUE TU ESPERAS?

Participante: A ver si mejoramos to el mundo sobre eso porque (participante se ríe)… No sé. (OK)

ENTREVISTADOR: CUANDO ESCUCHAS COMENTARIOS DE OTRAS PERSONAS QUE HAN DEJADO DE TOMARSE LOS MEDICAMENTOS PARA EL VIH… ¿QUE PIENSAS?.

Participante: Que no debieron de hacerlo. Porque sin eso no van a poder seguir viviendo. Por eso es lo que le va a dar vida a ellos.

ENTREVISTADOR: Y VERDAD, QUE RAZONES DAN ESAS OTRAS PERSONAS PARA DEJAR DE TOMAR MEDICAMENTOS.

Participantes: No sé, maybe depresión o… I dont know, yo de eso (el participante se ríe)

ENTREVISTADOR: QUE MÁS O MENOS, A VER SI ESCUCHAS DE OTRAS PERSONAS QUE DICEN O DE OTRAS PERSONAS, LO QUE ESCUCHAS…

Participante: O se sienten mal tu sabes por lo que les pasó, y ya no quieren vivir, sae que tienen muchas cosas que ellos tienen que (MJM)…

DRA CASTRO: ¿EN ALGÚN MOMENTO HAS PENSADO EN DEJAR DE TOMARTE LOS MEDICAMENTOS? ¿HAS PENSADO, TE HA PASADO POR LA MENTE?

Participante: “Jm”

DRA CASTO: ¿ESO ES, NO?

Participante: No eso no es…(participante se ríe) eso no, eso ,no me da por… (OK)

DRA CASTRO: PERO ME DIJISTE QUE SI EN ALGÚN MOMENTO HABÍAS DEJADO DE TOMARTE EL MEDICAMENTOS CUANDO TE FUISTE, VERDAD…

Participante: Si porque, am, me fui pa ya y (AJA)entonces, allí me iban a ayudar pero como yo dije que tenía casa acá en Puerto Rico (si) pues no me ayudaron pero me iban a ayudar pa (no entendí 5:13), me iban a ayudar pa apartamento, para todo me iban a ayudar (OK). Pero como yo dije que tenía casa acá.

DRA CASTRO: ¿LA CASA ESTÁ A NOMBRE TUYO? (mjm) OK.

DRA CASTRO: ESTE, EH… ¿Y QUÉ PASÓ QUE DEJASTE DE TOMÁRTELAS ALLÍ, QUE FUE LO QUE PASÓ, QUE NO TE LO PUDISTE TOMAR?

Participante: Porque cuando yo me fui de aquí, am, yo no estaba viviendo (no entendí 5:38)… las pastillas pa ya. (AH OK). So, se me quedaron acá (OK) y por eso fue que, am, (QUE DEJASTE DE TOMAR).

DRA CASTRO: ¿Y DEJASTE DE TOMARTELA POR CUÁNTO TIEMPO?

Participante: Tres meses (TRES MESES) eso fue lo que, porque yo estuve allá.

DRA CASTRO: ¿Y ALLÁ BUSCASTE SERVICIOS, ALLÁ? (si yo busque servicios porque me mandaron…) ¿DE, DE, OSEA PARA TU CONDICIÓN?

Participante: Si, para mi condición pero como, am, el medic-aid yo aplique también wallfare allá (AJA). Pero el medic-aid, a lo que me llegó el medic- aid y eso pues ya, perdón (el participante bosteza) ya yo tenía el pasaje pa venir pa acá.

DRA CASTRO: AH OK, OK, SABES QUE NO TENÍAS TAMPOCO PLAN MÉDICO Y ESO, (aja, exacto). AH OK. Y, ESTE, TRATASTE DE QUE TE MANDARAN LAS PASTILLAS DE ACÁ PARA ALLÁ O NO FUE POSIBLE.

Participante: No, no, llamé para que me las enviaran (AH Ok).

DRA CASTRO: ¿ALGUNA RAZÓN, SABES COMO, PARA QUE NO…?

Participante: No, porque, como yo tampoco era (no entendí 6:36), (Ah ok), tu sabes que, pues…

DRA CASTRO: ¿NO TENÍAS MANERA DE COMUNICARTE?

Participante: De, aja, de comunicarme con ellos (OK).

DRA CASTO: ¿Y POR EJEMPLO HAS DEJADO DE TOMARTE DOSIS? SABES QUE A VECES HAY LA DE POR LA MAÑANA, NO TE LA TOMAS O DE POR LA TARDE… ¿ALGUNA VEZ HAS DEJADO DE TOMAR DOSIS DE LAS PASTILLAS?

Participante: Lo que pasa es que yo me doy, tú sabes, (AJA) y a veces pues, am , si voy a beber pues no me voy a ligar las pastillas porque no es bueno, ligar las dos cosas (ah ok).

DRA CASTRO: ¿LO HAS HECHO ALGUNA VEZ, LA HAZ LIGAO? (no) NO

Participante: Cuando yo quiero así, que tengo ganas así de beber pues (no entendí 7:23), pues no me la bebo.

DRA CASTRO: AH OK.

Participante: Pero cuando termino la fiesta pues ya tú sabes que eso es wuf a (no entendí7:29). Me las bebo (ok).

DRA CASTRO: ¿Y NO TE BEBES LA DOSIS O NO TE BEBES LA DEL DÍA PARA QUE NO TE VAYA A AFECTAR?

Participante: No, me bebo todo el tiempo sabes la que me toca por la mañana así

ENTREVISTADOR: ¿LA QUE TE TOCA CERCA DEL MOMENTO EN QUE TE LA VAS A TOMAR, ESA NO TE LA BEBES?

Participante: Aja

ENTREVISTADOR:¿Y ESO TE PASA CON FRECUENCIA? ¿CÓMO CUANTAS VECES A LA SEMANA, MÁS O MENOS?

Participante: No to el tiempo. Es casual.

ENTREVISTADOR: OK, ENTONCES… A MUCHAS PERSONAS CON LA CONDICIÓN, CON VIH SE LES HACE DIFÍCIL TOMARSE LAS PASTILLAS TODOS LOS DÍAS, QUE ESO ES LO QUE ESTAMOS HABLANDO, POR DIVERSAS RAZONES, QUE ESTÁN DENTRO Y FUERA DEL CONTROL DE LA PERSONA. AHORA YO TE VOY A HACER UNAS PREGUNTAS SOBRE LAS POSIBLES RAZONES PARA QUE EN ALGÚN MOMENTO DEJES DE TOMARTE EL MEDICAMENTO O LAS DOSIS. VERDAD, PORQUE A VECES UNO NO SE TOMA EL MEDICAMENTO POR MUCHO TIEMPO PERO A VECES, COMO HABLAMOS AHORA, UNA DOSIS; NO ME TOMO LA DE POR LA MAÑANA, NO ME TOMO LA DE POR LA TARDE, ESO. PUES VAMOS A HABLAR DE LAS DOS COSAS. TEN EN MENTE QUE SI NO TE APLICA PARA TOMÁRTELO EN MUCHO TIEMPO, PIENSA SI EN ALGÚN MOMENTO SERÁ INFLUENCIADO EL NO TOMARTE UNA DOSIS. ESTE.. AHORA YO QUIERO QUE PIENSES EN TI, SIN INCLUIR OTRAS PERSONAS, SIN INCLUIR OTRAS CIRCUNSTANCIAS. ¿CÓMO TÚ TE DESCRIBES?

Participante: No sé. Ay yo no sé.

ENTREVISTADOR: ¿COMO DICEN LOS DEMÁS QUE TÚ ERES?

Participante: Que yo soy feliz. Que me la pasó siempre riéndome, tripeando, que me paso relajando con todo el mundo. Que yo, am… ¿Cómo se dice eso? soy amigable.

ENTREVISTADOR: ¿Y QUE MÁS DICEN DE TI? ¿O TÚ CREES QUE ERES ASÍ?

Participante: Ay porque yo siempre he sido así. Siempre. Desde jovencita, de que estuve afuera, siempre he sido así. (Ah ok)

ENTREVISTADOR: ESTE… Y POR EJEMPLO, LAS COSAS NEGATIVAS, QUE TODOS TENEMOS NUESTRO LADO NEGATIVO. ESAS COSAS NEGATIVAS COMO TAL, QUE PUEDAS, TUYAS, CARACTERÍSTICAS…

Participante: Tenía algunas porque cundo yo tenía problemas con mi marido yo me trate de envenenar.

ENTREVISTADOR: ¿CON EL DE AHORA OH…?

Participante: Con este. Con pastillas. Hasta me traté de ahorcar, cortarme las venas. Yo hice unas cuantas cosas, no te creas. (OK) Y me mandaron pa psicología (OK), yo estuve un tiempo en psicología. Que me querían encerrar y dije que no. Y yo dije, no, porque yo no estaba loca. Eso fue una depresión que me dio (TENÍA DEPRESIÓN) y le dije que no, que no me iban a encerrar. Y entonces la mai mía me quería encerrar. Y yo dije no mami no, si me encierran me empujo (AJA), le decía yo a ella. Pero na, hasta el sol de hoy.

ENTREVISTADOR: ¿YA ESTAS MEJOR?

Participante: Si. Y ya yo dije to eso, pal olvido.

ENTREVISTADOR: Y QUÉ COSAS QUE A VECES TE DICE LA GENTE, TÚ DEBES CAMBIAR ESTO TUYO, O ESTA FORMA DE SER TUYA. ¿O TÚ PIENSAS QUE DEBES CAMBIAR UNA FORMA DE SER TUYA?

Participante: Que yo sepa, por ahora no.

ENTREVISTADOR: OK PUES ENTONCES AHORA, CONSIDERAS QUE ALGUNAS DE ESAS CARACTERÍSTICAS TUYAS QUE ESTÁBAMOS HALANDO, VERDAD COSAS TUYAS, SENTIMIENTOS, LAS EMOCIONES TUYAS O ALGUNOS PENSAMIENOTS QUE HAYAS TENIDO, COSAS QUE TÚ CREES INFLUYEN EN LA DECISIÓN De, COMO , DESMOTIVARTE, DEJAR DE USAR LOS MEDICAMENTOS, O HAYA INFLUIDO EN EL PASADO.

Participante: No, eso no.

ENTREVISTADOR: ¿CUÁNDO TE SENTÍAS ASÍ DEPRIMIDA, EH, EN ALGÚN MOMENTO PENSASTE, VERDAD CUANDO ESTABAS ASÍ, DEPRIMIDA, SI TENÍAS CORAJE PENSASTE EN DEJAR DE TOMARTE LOS MEDICAMENTOS?

Participante: Si, pero no los deje porque tú sabes, pero me los bebía. (PERO , LO PENSASTE) Pensaba dejarlos por tanto problema y tantos desos que tenía. Yo nunca se lo deje dicho a mis hijos, sae la depresión que tenía. Ellos si se llegaron a dar cuenta de lo que yo iba a hacer sae, de lo que estamos hablando (no entendí 12:40)

ENTREVISTADOR: ¿ELLOS SE DIERON CUENTA? ¿Y QUÉ TE DIJERON?

Participante: A ellos no les gustó. Se enfogonaron conmigo (no entendí 12:49). Nadie me lo puede quitar, yo le decía a ello, hasta el sol de hoy.

ENTREVISTADOR: ¿Y ESTAS TOMANDO MEDICAMENTOS PARA LA DEPRESIÓN?

Participante: No, no estoy tomando.

ENTREVISTADOR: ¿TOMASTE EN ALGÚN MOMENTO?

Participante: En algún momento sí.

ENTREVISTADOR: ¿CUANDO?

Participante: Yo nunca lo veía, como te digo, el doctor nunca lo vi, yo iba a las entrevistas, charlas y eso y…

ENTREVISTADOR: ESTE.. Y POR EJEMPLO, LAS DOSIS EN ALGÚN MOMENTO CUANDO TE SENTÍAS ASÍ, QUE TE SENTÍAS DEPRIMIDA EH… ¿SE TE PASABAN DOSIS DEL MEDICAMENTO?

Participante: A veces se me pasaban.

DRA. CASTRO ¿CUÁNDO SE TE PASABAN ERA PORQUE NO QUERÍAS TOMÁRTELAS O PORQUE SE TE OLVIDABA O PORQUE?

Participante: No, es que se me olvidaban, ahí sí, era que se me olvidaban. (no entendí 13:47)

ENTREVISTADOR: ESTE.. POR LO MENOS HA HABIDO DOS VECES QUE HAS DEJADO LOS MEDICAMENTOS, CUANDO TE DIAGNOSTICARON TÚ ESTUVISTE TRES AÑOS Y CUANDO TE FUISTE PARA ALLÁ FUERA. ¿ALGÚN OTRO MOMENTO QUE HAS DEJADO DE TOMARTE LOS MEDICAMENTO POR UN PERIODO LARGO DE TIEMPO?

Participante: (No entendí 14:14-21)

ENTREVISTADOR: O SEA LO QUE ESTABA PASANDO EN ESE MOMENTO CUANDO, A PRINCIPIOS DE CUANDO TE ENCONTRARON LA CONDICIÓN, EN ESOS TRES AÑOS NO RECIBISTE SERVICIOS LO QUE PASABA ERA QUE NO SABÍAS VENIR PARA ACÁ. ¿ALGO MÁS QUE ESTABA PASANDO EN ESOS TRES AÑOS EN TU VIDA?

Participante: Pues nada en ese tiempo no me pasó más nada.

DRA. CASTRO> Y EN ESE TIEMPO, EN ESOS TRES AÑOS, TÚ LE HABÍAS DICHO A ALGUIEN MÁS QUE TU TENÍAS LA CONDICIÓN O NADIE LO SABÍA?

Participante: Mi madre, mis hermanos lo saben.

ENTREVISTADOR: ¿PERO SE LO DIJISTE CUANDO TE DIAGNOSTICARON?

Participante: Cuando me lo diagnosticaron.

ENTREVISTADOR: ENTONCES YA ELLOS LO SABÍAN. (aja) Y EN ESE TIEMPO ERA QUE NO SABÍAS VENIR PARA ACÁ... ¿LE PEDIAS A ALGUIEN QUE TE TRAJERA?

Participante: No, mi esposo me decía por teléfono: “Cuando yo salga W vamos a ir los dos juntos pa llevarte a un médico pa el tratamiento pa los dos”, me dijo él. Porque él nunca se iba a tratar, no sé si te lo dijo él. Nunca se había tratao. Él se vino a tratar conmigo, fue que se vino a tratar porque yo le dije que viniéramos (OK), hasta el sol de hoy.

ENTREVISTADOR: O SEA QUE TÚ ESPERASTE POR ÉL. (yeah) ¿TU QUERÍAS ESPERAR POR ÉL OH…?

Participante: Yo quería esperar por él porque yo quería que él también se tratara (OK).

ENTREVISTADOR: AHORA VAMOS A HABLAR DE LAS PERSONAS CON LAS QUE TÚ TE RELACIONAS, LAS PERSONAS QUE ESTÁN AHÍ CONTIGO. ¿QUIÉNES SON ESAS PERSONAS QUE USUALMENTE ESTÁN AHÍ CONTIGO, CON LAS QUE TÚ TE RELACIONAS?

Participante: Mi esposo, mi madre, mis amistades que siempre están en casa metidos y más na, así…

ENTREVISTADOR: ¿DE QUÉ MANERA, SI ALGUNA, ESAS PERSONAS CON LAS QUE TÚ TE RELACIONAS PODRÍAN INFLUENCIAR EN QUE TÚ DEJARAS DE TOMARTE EL MEDICAMENTO?

Participante: No les hago caso (NO LES HACES CASO), no.

ENTREVISTADOR: POR LO MENOS ME DIJISTE QUE EN ESOS TRES AÑOS EN QUE ESTUVO TU ESPOSO PRESO QUE ENTONCES AHÍ NO ERA QUE, VERDAD, DE UNA MANERA INDIRECTA PUES AHÍ NO TE LOS TOMASTE PORQUE ESTABAS ESPERANDO POR ÉL.

Participante: Si porque yo quería que él se tratara también (OK). Entiendes…

ENTREVISTADOR: TU FAMILIA, VERDAD… ¿ALGUIEN DE TU FAMILIA INFLUYE? TU SABES, QUE SE YO, A VECES A UNO LE PUEDE DAR CORAJE CON ALGUIEN Y ESE CORAJE QUE LE DA CON ALGUIEN (NO ENTENDÍ 19:40), TANTO CORAJE QUE NO ME QUIERO TOMAR LAS PASTILLAS.

Participante: Ahí no, aunque tenga coraje con quien sea yo, así sea con mi esposo yo me las bebo ahí. Yo me las tengo que beber.

ENTREVISTADOR: Y POR EJEMPLO, A VECES HAY PERSONAS QUE CUANDO LE PASAN COSAS CUANDO VIENEN A LAS CLÍNICAS, COSAS, VERDAD, QUE LOS FRUSTRAN, DICEN; “HAY, NO ME DAN NI DESEOS DE TOMARME LOS MEDICAMENTOS, NI DE VENIR A LA CLÍNICA A BUSCARLOS”. ¿ESO TE HA PASADO?

Participante: No. Siempre he venio (AJA).

ENTREVISTADOR: ¿Y EL PERSONAL DE ENFERMERÍA O LOS MÉDICOS QUE TE ATIENDEN DE ALGUNA MANERA ESO, DE ALGUNA MANERA…?

Participante: De maravilla me han atendio, tanto las enfermeras como la doctora. Ellos se llevan mucho conmigo, con migo y mi esposo, son locos eh…(AJA)

ENTREVISTADOR: ¿Y ALGUNA OTRA PERSONA QUE A LO MEJOR EN ALGÚN MOMENTO, TE, VERDAD, POR ALGO QUE HAYA PASADO CON ESA PERSONA TE HA VENIDO A LA MENTE NO TOMÁRTELO?

Participante: No, no a mí no me da por dejarla de beber.

ENTREVISTADOR: AHORA YO QUIERO QUE TÚ PIENSES EN EL AMBIENTE EN QUE TÚ TE DESENVUELVES. DONDE TÚ USUALMENTE ESTAS… ¿CÓMO ES ESE AMBIENTE, DONDE TÚ TE ENVUELVES?

Participante: En mi casa…

ENTREVISTADOR: OK. ¿CÓMO ES ESE AMBIENTE EN TU CASA?

Participante: Yo me pongo a recoger, a limpiar, hay música, a mí me gusta y a atender a mis perros, porque yo tengo cuatro perros.

ENTREVISTADOR: ¿SON TUS HIJOS?

Participante: Si. Lo único que tengo, porque mis hijos están todos en Estados Unidos, están todos por allá. (AH OK) Y me entretengo con los perros y en casa con mi esposo yeso, con las amistades. (OK)

ENTREVISTADOR: Y DE ALGUNA MANERA VERDAD… ¿ESTÁS TRABAJANDO, TRABAJAS?

Participante: Empiezo a trabajar ahora en enero.

ENTREVISTADOR: ¿Y HAS ESTADO TRABAJANDO AHORA…?

Participante: Si, en los tomates. De agricultura, ahí.

ENTREVISTADOR: ¿DE ALGUNA MANERA, SI ALGUNA, ESE AMBIENTE DONDE TÚ TE ENVUELVES (TU TRABAJO, CUANDO ESTAS CON LOS TOMATES, EN TU CASA, CUANDO ESTAS EN CASA DE TU MAMA, CUANDO ESTABAS ALLÁ EN NUEVA YORK) ALGO DE ESE AMBIENTE INFLUYÓ PARA QUE DEJARAS DE TOMARTE LOS MEDICAMENTOS?

Participante: No. Yo me los bebía, estando por allá yo me bebía los medicamentos. Yo me los bebía, todo..

ENTREVISTADOR: ¿ALLÁ ERA QUE NO LOS TENÍAS?

Participante: En New York, sí, nosotros nos los llegamos a beberlos. Acá fue, en Brooklin, cuando estuvo más de tres meses, pues ahí no

ENTREVISTADOR: ¿ANTES DE AHÍ, TÚ TE LOS BEBÍAS?

Participante: Eh íbamos a la clínica también.

ENTREVISTADOR: AH OK. O SEA QUE ALLÁ EN NUEVA YORK ERA QUE NO TENÍAS LA TARJETA.

Participante: Aja.

ENTREVISTADOR: ¿CUÁNDO ESTAS TRABAJANDO EN LO DE LOS TOMATES, RECOGIENDO LOS TOMATES, TE LLEVABAS LOS MEDICAMENTOS PARA EL TRABAJO?

Participante: Si, yo me los llevo (TE LOS LLEVAS, ¿TE LOS TOMAS?). Ahora mismito me dieron una que es de nevera y el muchacho me dijo que si yo me iba a salir o algo, eh, lo tengo que echar en un hielo para que no se dañe. Entonces, dije yo, yo voy a trabajar en los tomates y eso es bien caliente por allá, yo no sé qué yo me haré. La única manera es que yo consiga un pote de pastillas, por lo menos heche una que es la que me tengo que beber (EXACTO) y lo ponga en hielo y por la mañana me la beba (AJA EXACTO). No por la tarde, o sea, por la mañana (AH OK). Pa que no me, no me olvide de esa píldora que me falta (AH OK).

ENTREVISTADOR: SI, SI, SI, ESA ES BUENA IDEA.

Participante: Aja.

ENTREVISTADOR: ASÍ NO SE TE DAÑA…

Participante: Exactamente.

ENTREVISTADOR: ESTE… ¿Y EL AMBIENTE DONDE TÚ “JANGUEAS” O SOCIALIZAS? ME DIJISTE QUE A VECES TE DAS UN TRAGUITO Y ESO…

Participante: Aja. En mi casa, porque yo no salgo de mi casa, eso me lo doy en mi casa.

ENTREVISTADOR: Y CUANDO TE DAS EL TRAGUITO PUES AHÍ…

Participante: Ah, sí, me lo tengo que beber pa no ligar las dos cosas. Dicen que es malo, ligar pastillas con alcohol.

ENTREVISTADOR: AHORA QUIERO QUE PIENSES EN PUERTO RICO, SABES, LOS QUE ESTÁ PASANDO AQUÍ, LAS COSAS QUE ESTÁN PASANDO AQUÍ EN PUERTO RICO. ¿DE QUÉ MANERA SI ALGUNA, LAS COSAS QUE PASAN AQUÍ EN PUERTO RICO PUEDEN INFLUIR EN LA DECISIÓN DE ALGUNAS PERSONAS EN DEJAR DE TOMARSE LOS MEDICAMENTOS PARA EL VIH?

Participante: Pues si yo, como te dije, si las personas dejan de, tu sabes, las pastillas, so a lo mejor por depresión o que tienen algún problema con familiares o con el esposo y por eso dejan de bebérselas, (AH OK)digo, es lo que yo…

ENTREVISTADOR: ¿QUÉ TÚ PIENSAS EN LAS COSAS QUE PASAN EN EL GOBIERNO DE PUERTO RICO?

Participante: Diache. Acho el gobierno a veces ayuda a uno y a veces no, digo, yo siempre lo he dicho. Porque yo he ido a la alcaldía pa que me rellenen el patio de mi casa, si te digo la boleta desde cuando yo la tengo no me vas a creer.

ENTREVISTADOR: ¿DESDE CUÁNDO LA TIENES?

Participante: Yo la tengo desde el noventi-algo pa que me rellenaran mi patio (WAO) y todavía no me han dado el relleno.

ENTREVISTADOR: ESO ES UN MONTÓN DE TIEMPO.

Participante: Demasiao.

ENTREVISTADOR: DIANTRE.

Participante: Imagínate.

ENTREVISTADOR: Y POR EJEMPLO CON LAS AYUDAR PARA TU CONDICIÓN Y COSAS ASÍ. ¿ESO LO HAS TENIDO O TAMBIÉN TE HA PASADO QUE EL GOBIERNO TE PUEDE AYUDAR EN UNAS COSAS Y EN OTRAS NO?

Participante: No, ahí ellos me ayudan. Ahí sí porque eso, ahí me han ayudao.

ENTREVISTADOR: ¿Y QUÉ PIENSAS DE LOS SERVICIOS DE SALUD EN PUERTO RICO, DE SALUD, DE MÁS, LA MANERA COMO DAN LOS SERVICIOS DE SALUD AQUÍ EN PUERTO RICO?

Participante: Están, no sé, ayudan a uno, lo hacen lo más bien.

ENTREVISTADOR: ¿DE ALGINA MANERA? POR EJEMPLO… BUENO…YA ME HABÍAS HABLADO DE LOS PLANES MÉDICOS, QUE EL PLAN CUANDO ESTUVISTE ALLÁ EN BROOKLIN, PUES AHÍ TUVISTE PROBLEMAS PARA QUE TE DIERAN EL PLAN MÉDICO ENTONCES LOS MEDICAMENTOS… ¿DE ALGUNA MANERA ACÁ HAS TENIDO ALGÚN PROBLEMA CON EL PLAN MÉDICO, ALGÚN MEDICAMENTO QUE NO TE APROBARON?

Participante: Sí, algunos no me los aprobaron.

ENTREVISTADOR: OK. ¿Y QUÉ HAS HECHO, QUE HA PASADO AHÍ QUE ESTAS EN ESO?

Participante: Pues buscar chavos donde no tengo. Si no los tengo pues los dejo perder porque no puedo hacer más na y así.

ENTREVISTADOR: ¿Y ESOS MEDICAMENTOS, ALGUNOS TIENEN QUE VER CON LA CONDICIÓN?

Participante: A veces. Porque yo voy a mi doctor primario y le digo lo que tengo, el dolor y eso que me dan y el me receta según mi enfermedad, tu sabes, él me da medicamentos que no me…

ENTREVISTADOR: QUE NO VALLAN A COMPETIR CON LOS MEDICAMENTOS DE VIH… (aja, exactamente) ¿Y POR EJEMPLO PARA LOS MEDICAMENTOS DEL VIH, EN ALGÚN MOMENTO HAS TENIDO ALGUNA DIFICULTAD POR EL PLAN MÉDICO, UNA FIRMA, APROBACIÓN?

Participante: No, porque ellos me los daban, esta gente me los daban (TE LOS DABAN, YA NO), eran unos potes verdes.

ENTREVISTADOR: ¿Y AHORA COMO LOS CONSIGUES?

Participante: Ahora me los están dando por receta. Y yo voy y los busco a la farmacia.

ENTREVISTADOR: ¿Y CUANDO LOS BUSCAS SIEMPRE LOS TIENES?

Participante: Si siempre los tengo.

ENTREVISTADOR: Y POR EJEMPLO, LA SITUACIÓN ECONÓMICA DEL PAÍS QUE ESTAMOS VIVIENDO. ¿TÚ CREES QUE DE ALGUNA MANERA ESO PUEDE INFLUIR EN QUE, YA SEA QUE LA PERSONA NO TENGA LOS MEDICAMENTOS DISPONIBLES O QUE NO SE LOS PUEDA BEBER POR ALGUNA RAZÓN? ¿PIENSAS QUE ESO PUEDE INFLUIR?

Participante: Para mí sí.

ENTREVISTADOR: ¿DE QUÉ MANERA?

Participante: Pues porque si, am, como te digo, si no los tiene pues no podemos bebernos los medicamentos, o sea que no podemos, este….

ENTREVISTADOR: Y LA TRANSPORTACIÓN… ¿HA SUCEDIDO ALGUNA VEZ UNA BARRERA PARA QUE NO PUEDAS VENIR A LA CITA?

Participante: No. Yo vengo con él en el carro.

ENTREVISTADOR: AHORA QUIERO QUE PIENSES EN NOSOTROS COMO SOCIEDAD LOS PUERTORRIQUEÑOS, LAS COSTUMBRES Y LAS CREENCIAS DE LA SOCIEDAD. ¿CÓMO SOMOS NOSOTROS LOS PUERTORRIQUEÑOS? ¿LOS BORICUAS COMO SOMOS?

Participante: Buenos. Yo, pa mí son buenos, yo no sé.

ENTREVISTADOR: ¿CUÁLES SON LAS CARACTERÍSTICAS QUE TENEMOS NOSOTROS LOS BORICUAS?

Participante: Tratan bien a uno, que son… Hay alguna gente que tratan bien a uno y, no sé, y son chéveres como uno dice (OK, OK)

DRA. CASTRO. ¿Y COSAS ASÍ COMO NEGATIVAS DE NOSOTROS?

Participante: En algunos, en otros… Algunos son como si se hubiesen levantado del otro lao y a veces tratan a uno, este, y como yo no tengo tanto carácter tampoco pa aguantar (participante se ríe)… Pues pero algunos son así. (no entendí 8:56) Aja.

ENTREVISTADOR: ¿Y ALGUNA VEZ TE HAS SENTIDO DISCRIMINADA O RECHAZADA POR ESA GENTE QUE A VECES QUE SE LEVANTA POR EL OTRO LAO? ¿TE HAS SENTIDO RECHAZADA POR TENER LA CONDICIÓN?

Participante: No. Yo no me he sentido así.

ENTREVISTADOR: ESTE… ¿CONOCES PERSONAS QUE SE HAN SENTIDO ASÍ, RECHASADAS POR TENER LA CONDICIÓN?

Participante: Que yo sepa no he conocido más nadie.

ENTREVISTADOR: Y POR EJEMPLO LAS CREENCIAS RELIGIOSAS. ¿TÚ CREES QUE ESO DE ALGUNA MANERA INFLUYE DE QUE ALGUNAS PERSONAS, DIGAMOS, DEJEN DE TOMARSE LOS MEDICAMENTOS O ALGO ASÍ?

Participante: Alguno, puede ser que alguno. Porque yo no, puedo, como se dice, salirme del mundo y entrar a la iglesia pero yo no dejo mis medicamentos yo me los bebo. Porque Dios no te está diciendo a ti, “no te bebas los medicamentos” porque él sabe que uno lo necesita, entiendes…

ENTREVISTADOR: Y POR EJEMPOLO, LA SITUACION… DE ALGUNA MANERA LA SITUACIÓN SOCIAL EN QUE UNO VIVE INFLUYE EN QUE UNO PIENSE EN DEJAR DE TOMARSE LOS MEDICAMENTOS O LAS DOSIS DE LOS MEDICAMENTOS…

Participante: Bueno, dependiendo (¿DE QUÉ?) porque yo no dejaría, yo seguiría bebiendo pero hay muchas personas a veces que lo hacen, algunas, tú sabes

ENTREVISTADOR: Y ESTOS TIEMPOS QUE ESTAMOS VIVIENDO AHORA ¿DE QUÉ MANERA ESTOS TIEMPOS EN QUE ESTAMOS VIVIENDO, INFLUYEN EN DEJAR DE TOMARSE LOS MEDICAMENTOS?

Participante: Bueno, yo en realidad no se decirle, contestación.

ENTREVISTADOR: CUÁNDO EN LOS 3 AÑOS QUE ESTUVO SIN TOMARSE EL MEDICAMENTO Y EN LOS TRES MESES QUE ESTUVISTE EN BROOKLIN QUE NO TE LOS TOMASTE, CUANDO DEJASTE DE TOMÁRTELOS EN ESE MOMENTO… ¿QUÉ TE AYUDÓ A SEGUIR TOMÁNDOSELO?

Participante: ¿Cuando llegue aquí?

ENTREVISTADOR: CUÁNDO FINALMENTE TOMASTE LOS MEDICAMENTOS EN ESAS DOS OCASIONES… ¿QUÉ TE AYUDÓ A YO DECIR “AHORA ME LOS VOY A TOMAR”? ¿LA PRIMERA VEZ CUANDO ESTUVISTE TRES AÑOS, QUE FUE LO QUE TE AYUDÓ?

Participante: Pues mi esposo. Porque él me dijo que me iba a llevar a mi tratamiento.

ENTREVISTADOR: ¿Y CUANDO ESTUVISTE LOS TRES MESES EN BROOKLIN, QUE TE AYUDÓ?

Participante: Porque yo le había dicho a él que fuéramos otra vez a la clínica pa el tratamiento y el me trajo.

ENTREVISTADOR: ¿QUÉ LE DIRÍAS A UNA PERSONA QUE ESTÁ PENSANDO EN DEJAR DE TOMARSE LOS MEDICAMENTOS?

Participante: Pues que no lo dejen. Porque eso es una vida que nosotros tenemos, se puede decir. Sabes, que eso es lo que nos da más vida, pa seguir viviendo.

ENTREVISTADOR: Y CON LAS PERSONAS… ¿HAS TENIDO OPORTUNIDAD DE HABLAR CON PERSONAS QUE HAN DEJADO DE TOMARSE LOS MEDICAMENTOS?

Participante: No. No me ha tocao una, no me ha tocao.

ENTREVISTADOR: Y SI POR EJEMPLO TUVIESES LA OPORTUNIDAD DE HABLAR CON ALGUIEN QUE HA DEJADO DE TOMARSE LOS MEDICAMENTOS… ¿QUÉ LE RECOMENDARÍAS A ESA PERSONA, DE ACUERDO A TU EXPERIENCIA?

Participante: Pues que no lo dejen. Yo le diría, pues mira no lo dejes eso te va a dar una vida más adelante y es para vivir se puede decir y no es bueno que lo dejen yo les digo a ellos.

ENTREVISTADOR: OK. CUANDO ESCUCHAS LA FRASE “CUMPLIMIENTO DE LAS ORDENES MÉDICAS” ¿CON QUÉ LO RELACIONAS?

Participante: Ay no sabría decirle.

ENTREVISTADOR: ¿HAS ESCUCHADO LAS RECOMENDACIONES MÉDICAS? ¿LA HAS ESCUCHADO?

Participante: No

DRA. CASTRO. LAS RECOMENDACIONES MÉDICAS ES COMO LAS COSAS QUE TE RECOMIENDA EL MÉDICO QUE HAGAS. POR EJEMPLO ÉL TE RECOMIENDA QUE TE TOMES LOS MEDICAMENTOS PARA EL VIH, TE HACE UNA RECETA PORQUE ESA FUE LA RECOMENDACIÓN QUE TE DIO. ¿ADEMÁS DE TOMARTE LOS MEDICAMENTOS PARA EL VIH, QUÉ OTRA COSA TE RECOMIENDA EL MÉDICO QUE HAGAS?

Participante: Me manda a hacer pruebas y eso. Me manda a hacer pruebas de sangre, todas las pruebas que le hacen a uno.

ENTREVISTADOR: CUALES SON ESAS. DIME LAS QUE TE ACUERDES.

Participante: La tiroides, este, pa la diabetes, placas del pecho, no me acuerdo la otra, yo sé que me mandaron a hacerme unas cuantas.

ENTREVISTADOR: ¿Y DE ECOLÓGICAS?

Participante: Aja, esa. Y del cáncer vaginal. Me mandaron pal dentista y piensan mandarme pa esto, pa la vista y más na eso es lo único que...

ENTREVISTADOR: Y HAY ALGUNOS DE ESOS REFERIDOS O RECOMENDACIONES QUE TE DA EL DOCTOR QUE SE TE HA HECHO DIFÍCIL HACERLO O SEGUIRLO.

Participante: No yo lo hago. Rápido que me mandan yo lo hago. Digo, no nos es mucho que me recomendaron porque (no entendí 3:01) me dijo que como esta gente ahora están en vacaciones es capaz que valla pal dentista y esté en vacaciones. Sabes que tendré que esperar a hacerme las pruebas de (no entendí 2:50) y eso.

ENTREVISTADOR: Y POR EJEMPLO LAS PRUEBAS DEL PAP Y ESO DEL GINECÓLOGO, YA TE LO HICISTE. ¿CUÁNDO FUE LA ÚLTIMA VEZ QUE TE LO HICISTE?

Participante: Acho eso fue en los Estados Unidos.

ENTREVISTADOR: ¿HACE MÁS DE UN AÑO?

Participante: Si

ENTREVISTADOR: ¿CUÁNTO HACE?

Participante: Yo fui a Brooklin y no me lo llegué a hacer.

ENTREVISTADOR: ¿HACE MÁS DE CINCO AÑOS QUE TE LO HICISTE?

Participante: Como pal 2010 (HACE DOS AÑOS), porque yo me lo vine a hacer en New Yersey. Me lo hicieron como tres veces. (AH OK. ¿EL DEL?) Vaginal y to.

ENTREVISTADOR: ¿CUÁNTO HACE QUE NO VAS A UN GINECÓLOGO?

Participante: ay bendito, hacen años. (¿DE VERDAD?) Si hacen años que no voy al ginecólogo pa chequearme (AH OK)

ENTREVISTADOR: Y POR EJEMPLO, LA PRUEBA DE DIABETES CUANDO FUE LA ÚLTIMA VEZ QUE TE LA HICISTE.

Participante: Hace tiempo.

ENTREVISTADOR: ¿HACE MÁS DE UN AÑO?

Participante: Si, hace más de un año.

ENTREVISTADOR: ¿LA DE LA TIROIDES Y ESAS?

Participante: Si esa me mandaron a hacer la tiroide porque a mí me habían hecho hace bastante tiempo y en la primera salí bien, me la hicieron la segunda y salí mal en la segunda, me mandaron a hacer una tercera y salí bien y yo dije entre mi “la tiroide la tengo loca”. O sea, la tengo descontrolá, porque engordo, rebajo, engordo, rebajo y pues la tengo descontrolá.

ENTREVISTADOR: ¿Y TE MANDARON A IR AL MÉDICO, A AUN ESPECIALISTA?

Participante: No

ENTREVISTADOR: ¿TE DAN ALGÚN MEDICAMENTO PARA ESTO?

Participante: Nada, el medico primario mío, nada, no me ha dao nada nada.

ENTREVISTADOR: ¿Y LAS OTRA PRUEBAS QUE ME DIJISTE? ¿LA PLACA DEL PECHO?

Participante: Eso es pa la tuberculina. Porque no me la pueden hacer aquí porque me sale positiva por eso me la tienen que hacer en el pecho.

ENTREVISTADOR: Y LAS CITAS MÉDICAS… ¿SE TE HACE DIFÍCIL FALTAR A UNA CITA MÉDICA?

Participante: No, chacho. Yo trabajo en los tomates y a veces me dan cita de aquí yo lo que hago es que le enseño la cita a mi capataz y a mi jefe y ya ellos saben que yo falto ese día (AJA). So, ellos me lo pagan como quiera porque le estoy llevando evidencia de que tengo una cita en el medico.

ENTREVISTADOR: ¿HAY ALGUNA OTRA COSA QUE QUIERAS AÑADIR DEL TEMA, QUE NO HEMOS HABLADO?

Participante: Esta bien

ENTREVISTADOR: ENTONCES TERMINAMOS CON LA ENTREVISTA, EH, LA ENTREVISTA INDIVIDUAL.

ENTREVISTA #6

Participante femenina Hx de IDU

ENTREVISTADOR:: OK EH, ENTREVISTA NUMERO SEIS DE LA PARTE , FASE #1. A LA PARTICIPANTE SE LE DISCUTIO EL CONSENTIMIENTO. EH, SE LE ACLARARON DUDAS EN ESTE CASO NO HUBO DUDAS EN EL CONSETIMIENTO. ASI QUE LA PERSONA YA FIRMO Y PUES COMENZAMOS CON EL PROCESO DE ENTREVISTA.

ENTREVISTADOR:: ¿DESDE CUANDO ESTAS TOMANDO LOS MEDICAMENTOS PARA EL VIH?

Participante: desde junio 29 del 2005

ENTREVISTADOR:: OK, ¿Y EMPEZASTE A TOMARTE LOS MEDICAMENTOS CUANDO TE DIAGNOSTICARON LA CONDICION?

Participante: si

ENTREVISTADOR:: ¿O PASO ALGUN TIEMPO DESDE QUE TE LO DIAGNOSTICARAN?

Participante: no, cuando me lo diagnosticaron pues me dieron aquí el tratamiento y rápidito lo empecé.

ENTREVISTADOR:: OH OK, ESTE Y TENIAS LOS MEDICAMENTO AQUÍ?, LOS TENIAS DISPONIBLES?

Participante: si, si

DRA CASTRO: EH Y... ¿LEES LA INFORMACION DE LO QUE IMPLICA LOS EFECTOS SEGUNDARIOS DE LO QUE IMPLICAN LOS MEDICAMENTOS?

Participante: no (risas)

ENTREVISTADOR:: OH OK, ¿CONOCES LOS EFECTOS SEGUNDARIOS?

Participante: Si

DRA CASTRO: ¿SABES LOS EFECTOS SEGUNDARIOS?

Participante: si

DRA CASTRO: ¿QUE PIENSAS SOBRE LOS MEDICAMENTOS PARA TRATAR EL VIH?

Participante: Son buenos, porque son buenos y te ayudan.

DRA CASTRO: AJA

Participante: porque yo llegue a un, como te digo un periodo de que salen no detectables

DRA CASTRO: AJA

Participante: pero como no me los bebo ahí como tiene que ser

DRA CASTRO: AJA

Participante: pues eh te va salir que tiene la condición, te va a salir el sida porque ya no va a ser VIH

ENTREVISTADOR:: CLARO, OK. ¿YA PASASTE A LA ETAPA SIDA?

Participante: Si

DRA CASTRO: ESTE OK, ¿QUE TU ESPERAS DEL TRATAMIENTO PARA EL VIH? ¿QUE EXPECTATIVAS TIENES?

Participante: que pueda sacar una cura, que puedan sacar la cura y los que se puedan beneficiar pues que se beneficien.

ENTREVISTADOR:: OK ¿Y CUANDO ESCUCHAS COMENTARIOS DE OTRAS PERSONAS QUE HAN DEJADO DE TOMARSE LOS MEDICAMENTOS PARA EL VIH, QUE PIENSAS?

Participante: Yo he visto personas que se los beben y las he visto de lo más bien. Pero entonces he visto personas que se las beben y están pa’ atrás y pa’ atrás y pa’ atrás. Entonces, este los que se las beben me dicen “mira como yo estoy me siento bien que si tengo el los CDcuatro al dia”, los retrovirales son?

ENTREVISTADOR:: SI

Participante: Tengo todo bien. Que si no pones de tu parte pues no te los vas a beber pues no vas a echar pa’ lante sino que vas a echar pa’ tras pa’tras y pa’tras.

DRA CASTRO: ¿Y HAS VISTO PERSONAS QUE SE LOS BEBEN Y NO ECHAN PA’ LANTE?

Participante: si , yo

DRA CASTRO: DE VERDAD ¿TE LOS BEBES Y NO ECHAS PA’ LANTE?

Participante: nada, mira que flaca yo estoy

DRA CASTRO: OK

Participante: Yo pesaba ciento ochenta y pico’ de libras

DRA CASTRO: WOW

Participante: me pesaron ayer y pesaba ciento veintitrés

ENTREVISTADOR:: WOW ¿Y QUE TE HAN DICHO QUE PUEDA ESTAR PASANDO QUE NO ECHAS PA’ LANTE?

Participante: me cambiaron otra vez lo medicamentos ayer, no hacia ni un mes que me los cambiaron

ENTREVISTADOR:: AJA

Participante: y misi me los bebo, desayuno verdad y me los bebo ahora; ya a la media hora buaaaa los vomito.

ENTREVISTADOR:: LOS VOMITA?

Participante: si y si me bebo un poquito de agua, voto un galón con to’ y pastilla.

ENTREVISTADOR:: WOW

Participante: Entonces por eso yo deje de tomármelos. Porque te voy a ser sincera ayer mismito me cambiaron los medicamentos y hoy yo desayuné y tenía eso en la mente, y yo dije me los voy a beber, me los voy a beber pero entonces también tengo en la mente que me van a caer mal, los vómitos y estas otras cosas

ENTREVISTADOR:: OK

Participante: y pues no me los bebí. Te soy sincera hoy no me los bebí.

ENTREVISTADOR:: ¿NO TE LOS TOMASTES HOY?

Participante: No

ENTREVISTADOR:: OK

Participante: No, quien sabe si horita cuando vaya pues coma algo más fuerte y me los beba.

ENTREVISTADOR:: AH OK

Participante: pero es que esa es la molestia esa de aquí,

DRA CASTRO: SI DE VOMITAR?

Participante: los vómitos, ay si.

DRA CASTRO: ¿Y CUANTAS DOSIS TIENES AL DIA? ¿CUANTAS VECES TE LOS TIENES QUE TOMAR?

Participante: tres

DRA CASTRO: ¿TRES VECES?

Participante: no, no este.. esa este dos veces me dijo el ayer

DRA CASTRO: ¿DOS VECES AL DIA?

Participante: si, dos veces, pero la otra eran primero tenía un tratamiento que eran dos veces al día. Que eran seis pastillas: tres por la mañana y tres por la tarde. Entonces pues yo estuve nah como desde el 2005 hasta un mes bebiéndomelos pero que yo vi que en un momento salió “no detectable”

ENTREVISTADOR:: SI

Participante: pues tú te sientes de lo mejor porque contra “no detectable” no estoy bien pero tampoco estoy mal.

ENTREVISTADOR:: EXACTO

Participante: pero entonces después me los bebí y seguía como que pa’tras en vez de estar “no detectable” salía que estaba, que estaba mal como que no me los estaba bebiendo y el doctor me preguntaba y yo le decía sí , yo me los estoy bebiendo.

DRA CASTRO: AJA

Participante: Pero ¿porque entonces sale aquí así? (imitando al doctor) y yo: no sé porque en verdad, si no me los estuviera bebiendo te decía: no pero sí, me los estoy bebiendo. Pero entonces cuando me volvieron a hacer el laboratorio todo salió negativo, negativo, todo, todo, y yo decía espérate entonces no vale la pena bebérmelos y entonces que los laboratorios vengan a salir mal.

DRA CASTRO: SI, QUE TE SALIERON…ESTE AM Y NO TE LO DIJE HORITA PERO COMO NOTA ALCALCE ESTO ES COMPLETAMENTE CONFIDENCIAL, QUE NI SIQUIERA LO QUE NOSOTROS HABLEMOS AQUI LO DISCUTO CON EL MANEJADOR DE CASO NI NADA. ESTO ES CONFIDENCIAL.

DRA CASTRO: ESTE… Y POR EJEMPLO DOSIS, POR EJEMPLO AVECES ¿HAS DEJADO DE TOMARTE DOSIS, UNA DOSIS, DURANTE ES TIEMPO?

Participante: Si, porque a veces yo me las bebía la de por la mañana y me caían bien, pero había veces que me caían mal y cuando empezaba a vomitar así, yo decía espérate porque si estas me cayeron mal las de por la noche no me las voy a beber porque va a ser peor. Entonces voy a estar durmiendo con esas cosas, pues no, me bebía nada más que las de por la mañana.

DRA CASTRO: POR EJEMPLO UN DIA COMPLETO USTED….

Participante: si, también

DRA CASTRO: ¿TE PASABA?

Participante: si, si, estuve tiempo, claro como seis meses sin beberme nada.

DRA CASTRO: OK Y AHORA FUE QUE VOLVISTE

Participante: hoy fue que se me paso esa, pero horita yo me las bebo horita.

DRA CASTRO: OK, ESTE EM QUE SI HAS PENSADO DEJAR DE TOMARTE LOS MEDICAMENTOS Y ES POR LOS EFECTOS SEGUNDARIOS

Participante: ujum

DRA CASTRO: Y TE LOS HAS DEJADO DE TOMAR. ¿CUANTAS VECES TE LO HAS DEJADO DE TOMAR?

Participante: dos veces

DRA CASTRO: DOS VECES, ¿DENTRO DEL 2005 PARA ACA, DOS VECES?

Participante: si

DRA CASTRO: ¿Y LA PRIMERA VEZ FUE POR CUANTO TIEMPO?

Participante: por seis meses

DRA CASTRO: ¿Y LA SEGUNDA VEZ?

Participante: nah poquito como semana

DRA CASTRO: OK, PERO EN OTRAS OCASIONES SI TE HA PASADO QUE QUIZAS UN DIA NO TE LO TOMASTE

Participante: aja

DRA CASTRO: UNA DOSIS SE TE…

Participante: si, si me ha pasao’

DRA CASTRO: Y MAS DE UN DIA POR EJEMPLO, COMO DOS DIAS QUE HAYAS DEJADO DE TOMARTELOS

Participante: no, no porque entonces después vengo y me pongo a pensar ay que si me las bebo, que si no me las bebo pues está bien estoy bien pero entonces si me las bebo van a volver otra vez las náuseas y todas esas cosas pero entonces después me sacó eso de la mente porque si no es así no me las voy a beber y pues me las bebí después y gracias a Dios hasta ahora pues…

ENTREVISTADOR:: SI, OK ESTE VERDAD MUCHAS PERSONAS CON VIH EH ESPERATE TE HICE OTRA PREGUNTA, AM ANTES DE ESO VERDAD DE PASAR A ESA, ALGUNAS PERSONAS QUE A LO MEJOR TU HAS ESCUCHADO TU HAS HABLADO CON ELLAS O ESCUCHAS ASI VERDAD QUE PERSONAS DE LOS QUE NO SE TOMAN LOS MEDICAMENTOS ESTE QUE RAZONES PUEDEN TENER ESAS PERSONAS O ¿QUE RAZONES HAN DADO ESAS PERSONAS PARA NO TOMARSE LOS MEDICAMENTOS PARA EL VIH? PERSONAS QUE TU CONOZCAS.

Participante: que se quieren morir, que se quieren morir no tienen hijos, no tienen familia, yo por lo menos tengo mis hijos, tengo mi mama, tengo mi papa.

ENTREVISTADOR:: OK

Participante: que por lo menos tengo, mis hijos no saben mi condición. Porque ninguno lo sabe pero los demás familiares míos si.

ENTREVISTADOR:: OK

Participante: Entonces ellos mismo vienen y me apoyan. “mira fulan@ ven acá mírate esto, tu no quieres ver tus nietos? Tu no quieres ver tus hijos crecer? “ Y me apoyan me dan estimulo como dice uno de apoyo

DRA: CASTRO: SI

Participante: y pues me los bebo pero si no tengo ninguno la mente va a seguir negativo negativo y voy a seguir así no me los voy a beber.

DRA CASTRO: OK, Y ESAS OTRAS PERSONAS QUE HAS ESCUCHADO TAMBIEN ES PORQUE NO TIENEN A NADIE TU DICES.

Participante: no tienen a nadie, no.

DRA CASTRO: OK Y ¿QUE OTRAS RAZONES DAN ELLOS? POR EJEMPLO QUE SE QUIEREN MORIR, QUE OTRAS RAZONES QUE ELLOS DEN.

Participante: Por eso que no tienen apoyo

DRA CASTRO: ESTE COMO ESTABAMOS HABLANDO TU SABES QUE AVECES HAY PERSONAS QUE POR LA CONDICION QUE SE LES HACE DIFICIL TOMARSE LAS PASTILLAS COMO BIEN TU ME HAS DICHO TAMBIEN QUE SE TE A HECHO DIFICIL...

Participante: si

DRA CASTRO: TOMARTELAS TODOS LOS DIAS POR DIVERSAS RAZONES QUE ESTAN DENTRO Y FUERA DEL CONTROL DE UNO. AVECES DE UNAS UNO TIENE EL CONTROL DE OTRAS UNO NO TIENE EL CONTROL DE ESO.

Participante: ujum

DRA CASTRO: TE VOY HACER UNAS PREGUNTAS, UNA SERIE DE PREGUNTAS, SOBRE LAS POSIBLES RAZONES, PARA QUE EN ALGUN MOMENTO TU NO HAYAS DEJADO, VERDAD, HAYAS DEJADO DE TOMARTE LOS MEDICAMENTOS. ESTE YO QUIERO QUE TU PIENSES EN TI AHORA, SIN PENSAR EN FAMILIARES, EN OTRAS PERSONAS, SIN PENSAR EN OTRAS CIRCUNSTANCIAS, TU COMO PERSONA EH YO QUIERO QUE TU DESCRIBAS ¿COMO TU TE VES? COMO PERS.. TU SABES ¿COMO TU ERES QUIEN ERES TU? ¿COMO TU ERES COMO PERSONA?

Participante: Yo soy alegre

DRA CASTRO: aja

Participante: Yo soy activa, soy como eléctrica, entonces este, si tú necesitas un favor mío yo nunca te digo que no

DRA CASTRO: OK

Participante: bueno si está en mi disposición yo nunca te digo .. .Porque en verdad yo no sé decir que no.

DRA CASTRO: SI OK

Participante: Y esteeee nah soy alegre como te dije soy comunicativa, me gusta hablar con las personas, estoy estudiando, (DRA CASTRO: UJUM) que me gusta hablar. (ENTREVISTADOR:: SI) tengo que tener comunicación con los demás personas (DRA: CASTRO: AJA) estee pero me...así soy alegre.

DRA CASTRO: AH OK

Participante: Y ahora mismo me siento como deprimida porque

DRA CASTRO: AJA

Participante: este estoy echando pa’ tras pa’tras y pa’ tras

DRA CASTRO: AH OK ¿POR LA, LO DE LA CONDICION?

Participante: si

DRA CASTRO: OK

Participante : pero hasta ahora gracias a Dios, no me a vencer nada.

DRA CASTRO: OK Y POR EJEMPLO COSAS CARACTERISTICAS NEGATIVAS QUE AVECES QUE TU… ¿TODOS TENEMOS NUESTRO LADO NEGATIVO VERDAD?

Participante: aja

DRA CASTRO: COSAS ASI QUE, NEGATIVAS QUE UNO, QUE PUEDAS IDENTIFICAR TUYAS.

Participante: no, no tengo…negativo no

DRA CASTRO: COSAS QUE LA GENTE TE DICE “TU DEBERIAS CAMBIAR ESTO…”

Participante: (se ríe) ay mira….ay misi es que pues yo soy así

DRA CASTRO: AJA

Participante: entonces por las mañanas es que yo me levanto. Ay Dios mío con esa cara (DRA CASTRO : SI?) y con esa este (DRA CASTRO: AJA) ay con ese humor que me esta malo todo, todo me esta malo.

DRA CASTRO: MAL HUMOR

Participante: ay si todo usted, si te conozco o no te conozco tú me preguntas como estas? Ay bien (DRA CASTRO: AJA) ay bien pero que te pasa? Ay nada, ay déjame quieta, Es que no , Es que yo me levanto así, después que se me pasa es que me puedes hablar. Porque no…Ahora mismito estaba sentada ahí en la sala y hay un muchacho que vino conmigo y el me habla bien “Pero que te pasa?” ay nada déjame quieta… no es que no es que no es que soy así.

DRA CASTRO: AH OK

Participante: pero eso es por la mañana

DRA CASTRO: AH OK, PERO QUE BUENO QUE TE ESTOY ENTREVISTANDO Y YA SON LAS DOCE

Participante: no son ya las doce, entonces si no como nada también me pongo así de mal humor.

DRA CASTRO: COMISTE HOY?

Participante: desayune cuando vine pa acá.

DRA CASTRO: DESAYUNASTE, OK

Participante: pero que, yo soy alegre

DRA CASTRO: PERO EN CUANTO A ESO, SI OK. AHORA YO QUIERO QUE PIENSES, PIENSES SI ALGUNAS DE ESAS CARACTERISITICAS TUYAS ALGUNAS DE ESAS VERDAD DE CÓMO TU TE SIENTES DE CÓMO TU PIENSAS DE ESTO QUE TU HABLAS VERDAD DE LOS MALES HUMORES QUE AVECES QUE TE DAN EH COSAS QUE TU PIENSAS QUE TU CREES ¿INFLUYE DE ALGUNA MANERA PARA DESMOTIVARTE DE TOMARTE LOS MEDICAMENTOS?

Participante: mm no

DRA CASTRO: ESTE POR EJEMPLO ME DIJISTE QUE AVECES TE SIENTES ASI COMO DEPRIMIDA

Participante: si

DRA CASTRO: EH EL SENTIRE ASI VERDAD UNO SE SIENTE COMO TRISTE COMO..

Participante: si uno se siente como si la gente no lo quisieran, la gente no…sino pues la familia.

DRA CASTRO: LA FAMILIA

Participante: como que no apoyan a uno y pa mí no es así

DRACASTRO: Y CUANDO TU TE SIENTES ASI, TU SABES EN EL MOMENTO EN QUE TU TE SIENTE ASI QUE ESTAS AHÍ TU SABES ESTE AVECES TU DICES “AY NO VALE NI LA PENA TOMARME LOS MEDICAMENTOS”

Participante: aja

DRA CASTRO: ¿LO HAS PENSANDO?

Participantes: si, pero entonces llegan mis hijos a lado mío “¿mami que te pasa?” mami esto” nada papi nada. Porque yo tengo gemelos.

DRA CASTRO: TIENES GEMELOS?

Participante: si y casi siempre son los nenes

DRA CASTRO: DOS NENES GEMELOS?

Participante: si tengo dos nenes y dos nenas.

DRA CASTRO: DOS GEMELOS, DOS PARES DE GEMELOS?

Participante: no, tengo una de 17 y la nena de 13, pero los gemelos que son de 14, esos son los más apegao’ a mí. Entonces me ven a veces que estoy acostado o que me siento en el sillón o me pongo a bregar con el teléfono o algo, me ven rara.

DRA CASTRO: SI

Participante: y vienen pa acá “mami que te pasa”? nada entonces viene….”mami que yo te quiero mucho que yo te amo” Y yo papi, pero si no llega a ser por esos nenes

ENTREVISTADOR:: SI

Participante: no…en verdad no sé dónde yo estuviera

ENTREVISTADOR:: OH OK

Participante: créeme que no.

DRA CASTRO: ENTONCES CUANDO AVECES TE SIENTES ASI TAMBIEN PIENSAS EN…

Participante: si en los nenes

DRA CASTRO: PIENSAS EN LOS NENES Y POR ESO TE LOS TOMAS ¿PERO HAS PENSADO EN NO...

Participante: Si porque a veces me vienen pensamientos negativos y yo digo “ay no pero pa’ que yo me los voy a beber sino estoy echando pa’ lante”

DRA CASTRO: OK

Participante: pero entonces después vienen los pensamientos de los nenes, contra no voy a dejar los nenes huérfanos que si esto que si se van a quedar sin su mama y su papa. Porque el papa ahora mismo no esta tampoco.

DRA CASTRO: SU PAPA FALLECIO?

Participante: si, entonces ahí yo digo pues, me los voy a beber porque lo único que tienen ellos es a mí y tienen a mami pero no es lo mismo la mama que la abuela.

DRA CASTRO: UJUM EXACTO

Participante: pues eso es lo que me motiva a mí

DRA CASTRO: AH OK

Participante: pues bebérmelos aunque no heche pa’ lante.

DRA CASTRO: OK, ESTE, ESA, CUANDO TE DEJASTE DE TOMARTE LOS MEDICAMENTOS EH LA PRIMERA VEZ ME DIJISTE CUANTO TIEMPO FUE? COMO SEIS MESES

Participante: por seis meses.

DRA CASTRO: CUANDO DEJASTE DE TOMARTELOS POR SEIS MESES ¿QUE ESTABA PASANDO EN TU VIDA EN ESE MOMENTO?

Participante: ay Dios mío un montón de cosas, un montón de cosas yo este me vinieron vómitos. Me venía este esto de no comer porque en si yo como, porque como mucho y no sé porque en verdad estoy tan flaca.

DRA CASTRO: AJA

Participante: entonces este me ponía a comer. Este decía tu sabes yo me tengo que “desto” porque tengo que estar así porque los nenes míos... Aunque ellos no saben porque en verdad ellos no saben pero ellos ven, yo les digo a los nenes: “papi ven acá, mi casa es de alto y bajo, hazme un favor

DRA CASTRO: UJUM

Participante: en la coqueta allí hay unos potes yo le saco la receta, la.. la.. etiqueta

DRA CASTRO: AJA, SI.

Participante: allá hay unos potecitos blancos tráemelos y cuando yo siento que el viene crack crack y yo que tú estabas abriendo?

DRA CASTRO: AJA

Paciente: Y el: nah pa’ ver qué es esto. Y yo: no es que tú no puedes abrir eso. Por lo menos son de los de esos que no se pueden abrir.

DRA CASTRO: QUE TIENEN EL LOCK ESE AJA.

Participante: entonces ellos me dicen mami pero pa’ que? papi no es que me duele la cabeza o me duele el estómago o algo siempre le tengo que meter un embuste pa’ que ellos desto porque no voy a decirle no papi que esto es…así de cantazo.

DRA CASTRO: SI, SI

Participante: no, no todavía no estoy preparada para eso.

DRA CASTRO: OH OK, ESTE Y ENTONCES LO QUE ESTABA PASANDO ERA LOS EFECTOS…DE LOS VOMITOS Y TODO ESO ESTE... ¿ALGO MAS QUE ESTABA PASANDO EN TU VIDA? QUE SI… SITUACIONES, PROBLEMAS, QUE SI TE PONIAN ...QUIZAS TE AFECTABAN A TI EMOCIONALMENTE Y ESO PUES A SU VES PUES NO DEJABA QUE TE DEJARAN TOMAR LOS MEDICAMENTOS.

Participante: no

DRA CASTRO: ESTE AHORA QUIERO QUE ME HABLES, QUE PIENSES EN LAS PERSONAS CON LAS QUE TU TE RELACIONAS VERDAD, DE QUE MANERA SI ALGUNA LAS PERSONAS CON LAS QUE TE RELACIONAS INFLUYEN EN ESA DECISION DE DEJAR DE TOMARTE LOS MEDICAMENTOS.

Participante: no al contrario, por ejemplo mi vecina de al frente, yo no… porque yo le digo mi condición a todo el mundo

DRA CASTRO: SI, OK

Participante: porque a mi yo no tengo vergüenza de decirlo. Yo lo digo si me quiere seguir hablando me hablas sino pues tú por tú lao’ y yo por el mío.

ENTREVISTADOR:: AJA

Participante: porque tampoco voy a estar dejando de que, porque me siento mal de que me vengan no que está bien que ven acá que necesito algo de ti o algo no horita ya me siento ya como rechazada.

DRA CASTRO: OK

Paciente: Digo espérate esto aquí está pasando algo y es por esto, esto y esto. Que si yo no hubiera dicho esto pues no estuviera pasando esto pero que la vecina mía de al frente yo le digo y ella tiene tiroides

ENTREVISTADOR:: OK

Participante: Entonces ella viene y me dice “traite las pastillas” voy ya mismo y ella “dale que me voy a beber las mías” y yo vengo y subo busco las pastillas y yo mira! Y ella allá en el balcón de su casa y yo acá. Yo mira……… me las bebo y ella… ta’ bien. Si porque yo digo que mi mama me apoya

ENTREVISTADOR:: SI AJA

Participante: y me dice mira que los medicamentos esto que si ayudan los medicamentos, por la tarde, come, los medicamentos pero que me siento más apoyada por ella porque ella me dice yo tengo los míos aquí, yo me los voy a beber. Bébete los tuyos.

DRA CASTRO: ¿QUE TE HACE, VERDAD, PORQUE POR ELLA? QUE TU CREES QUE SEA LO QUE PASA LO QUE ELLA HACE O QUE SE YO...

Participante: No sé, porque, porque es que ella me dice, ella es como que no tiene la misma condición pero que yo veo como se está bebiendo también sus medicamentos y ella pues no tiene lo mismo pero tiene otra condición y se está bebiendo las pastillas porque yo no me puedo beber las mías? Así porque yo me siento bien… como le digo a ella “míralas aquí’ así pues me siento bien pero que no tener a nadie que me “desto” pues no me motiva a bebérmela.

DRA CASTRO: ¿NO TE MOTIVA A QUE PERDON?

Participante: a bebérmela

DRA CASTRO: ¿SI NO ES ASI?

Participante: no pero por lo menos tengo a mami que ella está ahí encima de mí.

DRA CASTRO: COMO QUE NECESITAS ALGUIEN QUE… ¿NECESITAS SENTIR EL APOYO DE ALGUIEN? PA BEBERTE...

Participante: si

DRA CASTRO: Y POR EJEMPLO LO EH LOS MEDICOS QUE TE ATIENDEN ACA HAS TENIDO ALGUNA SITUACION O ALGO ASI QUE TE HAYA INFLUIDO PARA QUE NO TE LOS TOMES? QUE SE YO TE TRATARON DE ALGUNA MANERA TU DICES AHHH

Participante: no, ellos son buenísimos aquí.

DRA CASTRO: EL PERSONAL DE AQUÍ

Participante: Si son muy buenos

DRA CASTRO: OK ESTE ALGUNA PERSONA QUE A LO MEJOR EN ALGUN MOMENTO TE HAYA TRATADO DE ALGUNA MANERA…

Participante: me han buscado una excusa como dice uno pa’ no bebérselo… no.

DRA CASTRO: AH OK ¿NO HAN SIDO EXCUSAS DE OTRAS PERSONAS?

Participante: no

ENTREVISTADOR:: AHORA PIENSA EN EL AMBIENTE EN QUE TU TE DESENVUELVES ESE AMBIENTE… ESTE.. ¿COMO ES ESE AMBIENTE? DESCRIBEME EL AMBIENTE EN EL QUE TU DESENVUELVES

Participante: Realmente es alegre, es tranquilo pero es bueno es alegre. Son muy comunicativos los vecinos parecen como como una familia son unida si yo no sé si… antes si yo cocinaba una comida en mi casa déjame llevarle al vecino o a fulana. Así mismo es allí. Es como si fuéramos familia.

DRA CASTRO: COMO SI FUERAN TODOS FAMILIA

Participante: si

DRA CASTRO: ¿Y TRABAJA?

Participante: no, estudio

DRA CASTRO: ESTAS ESTUDIANDO

Participante: si

DRA CASTRO: DE QUE MANERA SI ALGUNA ESE AMBIENTE VERDAD DE ESTUDIAS DONDE VIVES U ALGUN OTRO AMBIENTE DONDE HAYAS ESTADO VERDAD CUANDO NO TE LO TOMASTE VERDAD PORQUE NO NECESARIAMENTE SEA EL MISMO

Participante: ujum

DRA CASTRO: ESTE INFLUYO EN TU DECISION EN DEJARTE DE TOMAR LOS MEDICAMENTOS, QUE INFLUYO

Participante: mira yo estaba confinada, yo hacen seis meses o siete meses por ahí que salí. Entonces pues allá dentro uno en depresión, primera vez en mi vida

DRA CASTRO: ¿CUANTO TIEMPO ESTUVISTE?

Participante: cinco años

DRA CASTRO: WOW ESO ES MUCHO TIEMPO

Participante: yo dije espérate al principio pues uno, pues la primera vez como te dije estar fuera de mis hijos. Tan apegaos que soy yo a ellos y entonces yo dije no…ahí fue que yo caí en depresión.

DRA CASTRO: OK

Participante: y eché pa’tras pa’tras y después como al tiempito fue que volví otra vez; me daban los medicamentos y la doctora, la enfermera supone que me decía bébetelos aquí, como tienen el agua y todo y yo hacía un aguaje

DRA CASTRO: AH OK

Participante: y vine y me los sacaba esperaba que fuera pa’ otro lao’ y me los sacaba y los escondía. No me los bebía.

DRA CASTRO: NO TE LOS BEBIAS

Participante: No me los bebía, los de por la noche si pero los del por el día no.

Participante: Yo tenía a veces en la taquilla mía un montón de sobrecitos con las pastillas ay Dios mio espérate, que después vienen por ahí a hacer un registro o algo y yo cogía y las botaba

DRA CASTRO: AH OK Y NO TE LAS TOMABAS PORQUE TE SENTIAS...

Participante: Porque me sentía mal la primera vez en mi vida fuera de mis hijos,

DRA CASTRO: AH Ok

Participante: pero después ahí hay psicólogo hay psiquiatra y todo eso, entonces me llevaron ahí y me dieron otros hablé con ellos y me daban otros medicamentos para la depresión. Pero entonces ellos me apoyaron y ahí fue que yo empecé a bebérmelos.

ENTREVISTADOR:: Y LOS MEDICAMENTOS PARA LA DEPRESION ¿TE LOS TOMABAS?

Participantes: No, me los tomaba

DRA CASTRO: Y AHORA MISMO TE LO ESTAS TOMANDO PARA LA DEPRESION

Participante: No, Nada

DRA CASTRO: OK, ESTE ALGÚN OTRO AMBIENTE QUE ASI DE ESAS VECES QUE DEJASTE DE TOMARTELOS, COMO ESE EJEMPLO QUE ME DISTE QUE ESTA MUY BUENO

Participante: ujum

DRA CASTRO: ALGO ASI TU SABES PUES QUE EL AMBIENTE AVECES TE HACE SENTIR DE UNA MANERA O INFLUYE DE ALGUNA MANERA...

Participante: si, no entonces la primera vez pero mas ninguna no... así, no

DRA CASTRO: OK ESTE EM Y DONDE ESTAS ESTUDIANDO AHORA MISMO, ¿TE TOMAS LOS MEDICAMENTOS ALLI? SI TE TOCAN AHÍ CUANDO ESTAS ESTUDIANDO, ¿TE LOS LLEVAS Y TE LOS TOMAS?

Participante: si, yo me los llevo en el bulto

DRA CASTRO: Y TE LOS TOMAS O HAY VECES QUE TE LOS TOMAS Y...

Participante: No, me los tomo

DRA CASTRO: SI

Participante: si, ahí si porque este ahí nos dan brake de ... yo estoy de noche; pasa que hoy como es una actividad pues hoy es por el día.

DRA CASTRO: AH OK

Participante: entonces estoy de noche, entonces estoy de cinco a diez y como a las ocho o las nueve por ahí es que siempre me tocan, entonces nos dan el brake a las siete y media u ocho

DRA CASTRO: AJA

Participante: y yo pues aprovecho y me las bebo. Me tomo algo y me las bebo.

DRA CASTRO: AH OK ESTE Y DEL AMBIENTE DONDE TU JANGUEAS O SOCIALIZAS, ¿TU CREES QUE ESO INFLUYE DE ALGUNA MANERA? EN QUE NO TE LOS TOMAS

Participante: No

DRA CASTRO: AQUÍ EN ESTE AMBIENTE AQUÍ DE RECIBIR SERVICIOS... ALGÚN OTRO LUGAR?

Participante: No

DRA CASTRO: AHORA YO QUIERO QUE PIENSES EN PUERTO RICO. LAS COSAS QUE PASAN AQUÍ EN PUERTO RICO ¿DE QUE MANERA SI ALGUNA LAS COSAS QUE PASAN EN PUERTO RICO INFLUYEN EN LA DECISION DE ALGUNAS PERSONAS A DEJAR DE TOMAR LOS MEDICAMENTOS PARA EL VIH; COSAS QUE PASAN AQUÍ EN ESTE PAIS?

Participante: mmm bueno la...bendito como esta el día hoy en día como esos bebes... como matan a esos nenes chiquitos verdad ay no yo no puedo. Yo me pongo a veces a ver a mis nenes.

DRA CASTRO: AJA

Participante: yo digo yo no se como hay padres que cogen y pueden matar a sus propios hijos. Pero yo tranquila.

DRA CASTRO: UJUM ESTE POR EJEMPLO EL GOBIERNO COSAS QUE HACE EL GOBIERNO, ¿CREES QUE DECICIONES O COSAS QUE HACE EL GOBIERNO DE ALGUNA MANERA INFLUYE PARA QUE LA PERSONA NO PUEDA TOMARSE LOS MEDICAMENTOS?

Participante: No... mira ahora mismo yo cuando salí de confinada

DRA CASTRO: SI

Participante: yo fui a buscar ayuda a los cupones

DRA CASTRO: AJA

Participante: me dijeron que no

DRA CASTRO: ¿NO? ¿PORQUE?

Participante: porque estaba confinada

DRA CASTRO: AHH

Participante: yo digo pero que tiene que ver una cosa con la otra

DRA CASTRO: AJA

Participante: yo he visto otras personas que entran y salen y le dan los cupones na’ mas de emergencia y ¿porque tiene que ser yo? ... mire me entró una rabieta que yo vire to’ aquello allí.

DRA CASTRO: AH OK

Participante: pero me dijeron que no, entonces porque me vengan a decir que no ¿yo me voy a refugiar en eso para entonces yo no beberme los medicamentos? no

DRA CASTRO: OK Y ¿TU CREES QUE HAY PERSONAS QUE LO PUEDEN HACER? ¿QUE TENGAN LA CONDICION Y LO HAGAN? ¿HAS ESCUCHADO DE PERSONAS QUE LO HAGAN?

Participante: si

DRA CASTRO: QUE COSAS HAS ESCUCHADO DE ESAS PERSONAS?

Participantes: pues que... un ejemplo ahora con nosotras aquí que vengan y me digan que si tienen una entrevista con la doctora que se yo que que si te van a dar tanto que si.... pues vengan y te digan no que si no te los van a dar

DRA CASTRO: AJA

Participante: ah pues entonces pa’ que yo voy a llenar eso

DRA CASTRO: AJA

Participante: o pa’ que yo me los voy a beber

DRA CASTRO: SI

Participante: ps no ps eso no es excusa

DRA CASTRO: SI, PERO ¿TU CREES QUE HAY GENTE QUE LO COGE DE EXCUSA?

Participante: si hay gente que si, lo cogen de excusa

DRA CASTRO: OK

Participante: “no que si me dijeron que fuera allí que si esto... mira yo no me voy a beber na” porque hay gente así.

DRA CASTRO: AH OK

Participante: yo digo que eso no es excusa, eso es que no se la quieren beber.

DRA CASTRO: UJUM, SI, EXACTO AH OK. Y POR EJEMPLO HAS ESCUCHADO DE GENTE QUE TOME DE EXCUSAS POR EJEMPLO QUE SI LOS SERVICIOS QUE ME DIERON NO ME GUSTARON Y POR ESOS NO ME LAS VOY A TOMAR

Participante: aja

DRA CASTRO: ¿HAS ESCUCHADO ESO?

Participante: si

DRA CASTRO: OK Y ESTE PLANES MEDICOS POR EJEMPLO TU CREES QUE DE ALGUNA MANERA INFLUYE O LO COGEN DE EXCUSA COMO TU DICES.

Participante: si de excusas pero no, no creo que influya

DRA CASTRO: OK AHORA MISMO ¿TU TIENES PLAN MEDICO?

Participante: la reforma

DRA CASTRO: LA REFORMA , ¿TE CUBRE TODOS LOS MEDICAMENTOS?

Participante: si

DRA CASTRO: ¿TIENES TODOS?

Participante: si

DRA CASTRO: EN ALGÚN MOMENTO DURANTE TODO ESTE TIEMPO DE LA CONDICION VERDAD DESDE EL 2005 QUE ME DIJISTE QUE TE DIAGNOSTICARON. ¿HAS TENIDO POR EJEMPLO ALGUNA DIFICULTAD DE QUE A LO MEJOR NO TE APRUEBEN ALGÚN MEDICAMENTO QUE TUVISTE QUE QUEDARTE SIN EL?

Participante: no, hasta ahora gracias a Dios

DRA CASTRO: TODO ESTA AL DIA Y POR EJEMPLO LA SITUACION ECONOMICA DEL PAIS POR EJEMPLO ¿TU CREES QUE ESO PUEDA DE ALGUNA MANERA INFLUIR PARA QUE ALGUNA PERSONA NO PUEDA TOMARSE LOS MEDICAMENTOS?

Participante: no, no creo. No, porque si tienen la ayuda

DRA CASTRO: UJUM

Participante: y tienen la reforma, por ejemplo yo porque entonces va a buscar a otro lao’ si la tienes ahí.

DRA CASTRO: AH OK ESTE Y LA TRANSPORTACION ¿ALGUNA VEZ LA TRANSPORTACION HA SIDO UNA DIFICULTAD PARA TI?

Participante: si, porque no tengo transportación.

DRA CASTRO: OK... Y AL NO TENER TRANSPORTACION ¿SE TE HA HECHO DIFICIL PARA LOS MEDICAMENTOS?

Participante: SI

DRA CASTRO: ¿HAS ESTADO TIEMPO SIN TOMARTELOS POR EL HECHO QUE NO TIENES TRANSPORTACION?

Participante: no yo como quiera vengo, así sea tarde pero vengo en la guagua en la cita esa..

DRA CASTRO: AH OK

Participante: que eso es gratis, pues vengo y me tiro y así sean las doce que cierran la farmacia yo llego a las menos cinco, como quiera estoy aquí y me lo tienen que dar.

DRA CASTRO: AH OK ESTE ELLOS TIENEN TRANSPORTACION AQUÍ TAMBIÉN

Participante: si, ellos tienen un taxi me dijeron ayer

DRA CASTRO: ¿ALGUNA SITUACION EN EL PAIS QUE TU CREES QUE INFLUYA PORQUE A LO MEJOR LAS PERSONAS NO TENGAN QUIZAS DISPONIBLES LOS MEDICAMENTOS O QUE NO TENGAN...

Participante: no

DRA CASTRO: AHORA YO QUIERO QUE PIENSES EN LAS EXPERIENCIAS DE NOSOTROS LOS PUERTORRIQUENOS Y NUESTRA SOCIEDAD, ¿ DE QUE MANERA SI ALGUNA COSTUMBRES Y CREENCIAS DE NUESTRA SOCIEDAD INFLUYE EN LA DECISION DE DEJAR DE TOMAR LOS MEDICAMENTOS PARA EL VIH O ESO PUEDE SERVIR DE EXCUSA, COMO TU DICES, PARA QUE UNA PERSONA NO SE TOME LOS MEDICAMENTOS?

Participante: aja lo de excusas porque si tu quieres seguir hacia delante pues tu tienes que bebértelos tienes que proponértelo. Tienes que poner de tu parte pa’ bebértelos porque como tu vas a salir de un...

DRA CASTRO: SI, OK Y POR EJEMPLO ESTE LA DISCRIMINACIÓN EL RECHAZO. ¿ALGUNA VEZ TE HAS SENTIDO RECHAZADA POR TENER LA CONDICIÓN?

Participante: Si

DRA CASTRO: SI EH ¿Y EL TU SENTIRTE RECHAZADA DE ALGUNA MANERA ESO INFLUYO EN QUE PENSARAS EN NO TOMARTELOS O DEJARAS DE TOMARTE LOS MEDICAMENTOS?

Participante: no , yo digo pues si me rechazan yo tengo que seguir positivo porque si me va a rechazar porque tengo esto yo no me voy a pegar por eso o sea no beber los medicamentos. Si tu no me quieres hablar y me estas rechazando yo tengo que seguir hacia delante.

DRA CASTRO: UJUM OK Y ESTE ¿CONOCES A ALGUIEN O POR EJEMPLO HAS ESCUCHADO PERSONAS QUE SE HAN SENTIDO RECHAZADO Y HAN DEJADO DE TOMARSE LOS MEDICAMENTOS?

Participante: no

DRA CASTRO: Y ESTE CREENCIAS RELIGIOSAS, ¿COMO TU CREES QUE LAS CREENCIAS RELIGIOSAS PUEDEN INFLUIR EN QUE UNA PERSONA DEJE DE TOMARSE LOS MEDICAMENTOS?

Participante: no se, ¿como es? ¿las creencias religiosas?

DRA CASTRO: LAS CREENCIAS RELIGIOSAS

Participante: bueno pues yo soy católica... hay... como te digo...hay este religiosos que no creen en nada de eso porque hay este... como te digo hay...no me sale la palabra pero este es como decir este los de la iglesias hay unos que son los pentecostales que ellos no creen en nada porque ellos no creen en nada. Que si tomate estas pastillitas para el dolor de cabeza...”no pues si eso no te lo va a quitar, tienes que tener fe para que se te quite el dolor de cabeza” que ellos no creen en nada. Pero pa’ mi yo soy católica y mi religión...

DRA CASTRO: OK Y LA SITUACIÓN SOCIAL, TU SITUACIÓN SOCIAL VERDAD ¿TU CREES QUE HA INFLUIDO?

Participante: no

DRA CASTRO: ¿ALGUNA OTRA SITUACION?

Participante: no

DRA CASTRO: AHORA PIENSA EN ESTOS TIEMPOS QUE ESTAMOS VIVIENDO TU SABES LA EPOCA QUE VIVIMOS AHORA MISMO ESTE ¿DE QUE MANERA SI ALGUNA ESTOS TIEMPOS EN QUE VIVIMOS INFLUYEN EN LA DECISION DE DEJAR DE TOMARSE LOS MEDICAMENTOS?

Participante: ay virgen el tiempo que estamos viviendo eso es así

DRA CASTRO: ¿SI? ¿COMO TU CREES QUE PUEDE INFLUIR EN LA DECISION DE ALGUIEN DE TOMARSE LOS MEDICAMENTOS?

Participante: bueno yo, yo me los bebería

DRA CASTRO: AJA SI

Participante: porque como estamos hoy en día y la ciencias están tan avanzadas quien sabe si ahora mismito yo tengo sida, porque no es HIV sino sida, quien sabe si yo siga mi tratamiento y de aquí a un año mas vengan y me digan ven acá que te vamos a coger de conejitos de india, como uno dice, este para hacerte estas pruebas a ver si con esto tiene la cura.

DRA CASTRO: UJUM

Participante: así

DRA CASTRO: OK, OSEA QUE TU CREES QUE PUEDEN INFLUIR EN QUE TE PUEDA, QUE EN LOS TIEMPOS QUE ESTAMOS VIVIENDO AHORA DE AVANCE DE LA CIENCIA A LO MEJOR PUEDA LLEGAR LA CURA.

Participante: No no yo tengo fe que si va a llegar

DRA CASTRO: OK ESTE Y POR EJEMPLO ¿TU CREES VERDAD LOS TIEMPOS QUE ESTAMOS VIVIENDO TU CREES QUE OTRAS PERSONAS QUE POR LAS COSAS QUE SE ESTAN VIVIENDO HOY EN DIA PUEDAN TENER LA EXCUSA PARA NO TOMARSE LOS MEDICAMENTOS... QUE INFLUYAN?

Participante: no, bueno yo no creo

DRA CASTRO: OK ¿Y DE OTRAS PERSONAS? POR EJEMPLO

Participante: no creo

DRA CASTRO: NO, OK ESTE EH CUANDO DEJASTE DE TOMARTE LOS MEDICAMENTOS EN EL PASADO ¿QUE TE AYUDO A VOLVER A TOMARTELOS QUE FUE LO QUE TE AYUDO?

Participante: Pues los psicólogos, los psiquiatras, los tratamientos que me dieron.

DRA CASTRO: AH OK

Participante: el apoyo

DRA CASTRO: ESTE Y CUANDO PENSASTE EN MOMENTOS, SI PENSASTE DE DEJAR DE TOMARTELOS VERDAD QUE AVECES ME DIJISTE QUE PENSASTE EN NO TOMARTELOS ¿QUE TE AYUDO A SEGUIR TOMANDOTELOS?

Participante: pensar en mis hijos y no dejarlos huérfanos de madre. Porque ya que no tienen a su papa tampoco van a tener a su mama entonces se van a sentir en... sin mami sin papi.

DRA CASTRO: SI OK ¿Y ALGO MÁS QUE TE HAYA AYUDADO A SEGUIR TOMANDOTELOS?

Participante: pues mi mama, mi familia

DRA CASTRO: ¿QUE LE DIRIAS A UNA PERSONA QUE ESTA PENSANDO EN DEJAR DE TOMARSE LOS MEDICAMENTOS?

Participante: no que no los deje que tarde o temprano va a llegar la cura.

DRA CASTRO: OK

Participante: que tenga fe y que se ponga en su mente que con los medicamentos vamos a muy lejos. Sin los medicamentos no vamos a llegar a nada.

DRA CASTRO: Y EH ¿HAS TENIDO LA OPORTUNIDAD DE HABLAR CON PERSONAS QUE HAN DEJADO DE TOMARSE LOS MEDICAMENTOS?

Participante: um no

DRA CASTRO: OK ¿Y SI TUVIERA LA OPORTUNIDAD DE HABLAR CON ALGUIEN, QUE LE RECOMENDARIAS? A ALGUIEN QUE SE LOS HA DEJADO DE TOMAR.

Participante: que se los bebiera otra vez que empezara otra vez el tratamiento porque como te dije vamos a tener la cura

DRA CASTRO: UJUM

Participante: tarde o temprano es pero la vamos a tener.

DRA CASTRO: CUANDO ESCUCHAS LA FRASE RECOMENDACIONES MEDICAS, ¿CON QUE LA RELACIONAS?

Participante: ¿Recomendaciones?

DRA CASTRO: AJA

Participantes: ay Dios mio recomendaciones pues que este... que te están aconsejando para otros medicamentos.

DRA CASTRO: OK ¿Y CUANDO DECIMOS CUMPLIMIENTO DE LAS RECOMENACIONES?

Participante: ahí estas cumpliendo con tu deber con tus medicamentos

DRA CASTRO: ESTE ADEMAS DE TOMAR LOS MEDICAMENTOS PARA EL VIH VERDAD DE ESAS RECOMENDACIONES QUE TE DA TU MEDICO, ¿QUE OTRAS RECOMENDACIONES EL O ELLA TE DA?

Participante: no ninguna que me los beba, que siga el tratamiento como hasta hoy, que estoy bien, no estoy mal, si estoy mal pues que me los siga bebiendo para mejorar eso.

DRA CASTRO: AH OK

Participante: si estoy bien pues que siga bebiéndomelos que estoy bien que estoy ahí.

DRA CASTRO: ELLA TE RECOMIENDA A HACERTE ALGUNA DE ESTAS... OK ¿ALGUNAS RECOMENDACIONES QUE ELLA TE DA SE TE HACE DIFICIL SEGUIRLAS?

Participante: no, a veces lo de los medicamentos.

DRA CASTRO: LO DE LOS MEDICAMENTOS

Participante: si, pero ya no

DRA CASTRO: ¿TE RECOMIENDA HACERTE EXAMEN FISICO, OTROS EXAMENES MEDICOS?

Participante: si ... hace frio verdad

DRA CASTRO: SI

Participante: ayer mismo me dio un papelito para hacerme unos laboratorios afuera

DRA CASTRO: AH OK ESTE Y ¿TE HA RECOMENDADO, TE HA DADO REFERIDO PARA OTROS MEDICOS?

Participante: no

DRA CASTRO: NO, OK

Participante: no aquí mismo con la infectologa

DRA CASTRO: AH OK

Participante: porque me salió esto... y aquí... y entonces me dijeron ayer que por los mismos medicamentos.

DRA CASTRO: AH OK

Participante: él me dijo “eso es que no te estas bebiendo los medicamentos.” Y yo ay Dios mio, me quede así como que..

DRA CASTRO: ESO ES QUE, ¿QUE TE DIJO?

Participante: “que no te estas bebiendo los medicamentos”

DRA CASTRO: AH OK

Participante: y yo me quede Ay Dios mio, porque sabrán tanto verdad? (risas)

DRA CASTRO: BUENO QUE ESTEN PARA AYUDARTE (RISAS)

Participante: si, son doctores, yo estaba en pantalones cortos y yo le digo al doctor “mire doctor...” y el eso es que no ... y yo ay Dios mio ... y el verdad? Y yo no me los estoy bebiendo.. y él ¡viste! Eso también... eso se ve clarito.

DRA CASTRO: AH OK

Participante: él me dijo que va a hacer como una biopsia para ver de que era

DRA CASTRO: AH OK

Participante: porque yo tenia esto bien feo, entonces se me curo yo me estoy untando bastobran, se me curo y volvió otra vez y me salió.

DRA CASTRO: OK

Participante: ahora mismito no me pica, pero a veces yo me cojo yo y me esbarato,

DRA CASTRO: AY BENDITO

Participante: cojo el cepillo como no tiene unas y me hago canto y aquí también me lo hago canto.

DRA CASTRO: ¿Y NO TE DUELE DESPUES?

Participante: No, no me duele

DRA CASTRO: ¿SE TE QUITA EL PICOR?

Participante: se me quita el picor

DRA CASTRO: PERO NO HAGAS ESO MUCHACHA

Participante: Mira ayer.. anoche mismo

DRA CASTRO: LA SOLUCION ES...

Participante: (risas) seguir los medicamentos

DRA CASTRO: SEGUIR LOS MEDICAMENTOS

Participante: el tratamiento, anoche mismo aquí yo me estaba rascando. Y estoy acosta en la cama y yo ahí ahí ahí ahí ahí hasta que pues me quede dormía’ y cuando esta mañana voy a chequear la cama veo un puntito de sangre y yo “ay yo no estoy en menstruación” y cuando yo eso fue... el pie.

DRA CASTRO: SI PORQUE TIENES AHÍ MIRA..

Participante: si aquí esta todavía reciente pero entonces esta mañana tenía como aquí sangre pega, pero espérate si yo no estoy en menstruación y yo “ay eso fue el pie anoche”. Y era eso, él me dijo eso me quede así como que en shock cuando me dijo “esos son los medicamentos que no te los estas bebiendo” y yo “ay Dios mio”

DRA CASTRO: PUES YA SABES, Y POR EJEMPLO ¿TE MANDARON PARA QUE OTRO MEDICO TE VIERA EL RASH QUE TE SALIO?

Participante: No, no me vio el ayer pero no me dijo nada de la infectologa pero la otra vez hace tiempito ya antes de yo caer confinada, la doctora me vio, la infectologa y me dio una cremita pa’ esto y me curó.

DRA CASTRO: AH OK

Participante: Pero que ahora pa’ esto no, me estoy untando Bastobran. Yo acá mi mama me lo compro.

DRA CASTRO: MIRA Y EL GINECOLOGO, ¿HAS IDO AL GINECOLOGO?

Participante: si

DRA CASTRO: ¿CUANDO FUE LA ULTIMA VEZ?

Participante: ay hacen como seis meses

DRA CASTRO: TE HICIERON LA PRUEBA DEL PAP Y TODO ESO

Participante todo eso, Salí con un quiste en un ovario pero

DRA CASTRO: UM OK

Participante: pero de lo otro estoy de lo mas bien. Estuve cuatro anos sin ver menstruación.

DRA CASTRO: WOW

Participante: y después me bajo lo mas bien. Esos cuatro años yo no fui al ginecólogo yo dije “ay que dulzura”

DRA CASTRO: ¿PERO ERA CUANDO ESTABAS CONFINADA?

Participante: no estaba fuera

DRA CASTRO: AH OK

Participante: estaba fuera antes de tener la condición

DRA CASTRO: AH OK, ANTES DE TENER LA CONDICION

Participante: Entonces después yo vine y cogi... después me bajo en el baño y yo... entonces después estuve confinada y estuve nueve meses sin ver menstruación.

DRA CASTRO: SIN VER MENSTRUACION, OK

Participante: hasta que salí los últimos nueve meses sin ver menstruación y yo “ay que rico me voy pa la calle sin ver menstruación”

DRA CASTRO: ¿PERO ALLI TE CHEQUEARON?

Participante: si me chequearon me dieron unas pastilla y me la bajaron

DRA CASTRO: Ok

Participante: pero de todo estoy bien gracias a Dios

DRA CASTRO: Y ESTO CUANDO TE DAN ESOS EXAMENES ¿TE LO HACES O A VECES SE TE HACE DIFICIL HACERTELOS?

Participante: no, a veces se me hace difícil

DRA CASTRO: SI

Participante: pero me los hago

DRA CASTRO: ¿PORQUE SE TE HACE DIFICIL?

Participante: por el PAP

DRA CASTRO: AH OK

Participante: porque hay que pagar afuera

DRA CASTRO: ¿AH ESO HAY QUE PAGAR FUERA?

Participante: si por mas que tenga la tarjeta

DRA CASTRO: AHH TIENE DEDUDICIBLE?

Participante: si, no yo tengo cero. Deducible cero

DRA CASTRO: ¿PORQUE TIENES QUE PAGAR?

Participante: porque eso no lo cubre

DRA CASTRO: AH NO TE LO CUBRE

Participante: no, tengo que pagar

DRA CASTRO: ¿Y EN CUANTO SALE? ¿SALE CARO?

Participante: cincuenta pesos

DRA CASTRO: mmm

Participante: eso, ese papelito cincuenta pesos me salio esas pastillas, esos laboratorios

DRA CASTRO: ¿O SEA QUE TIENES QUE ESPERAR A CONSEGUIRLOS?

Participante: si, tengo hasta el mes que viene para entregarla

DRA CASTRO: Y ESTO LABORATORIOS... SIEMPRE TE PASA CON LOS LABORATORIOS QUE NO TE LO CUBRE EL PLAN Y TIENES ..

Participante: con los laboratorios de calle

DRA CASTRO: mmm

Participante: porque casi siempre me los hacen aquí

DRA CASTRO: AH OK

Participante: pero son con eso

DRA CASTRO: ¿DONDE TE LOS HACEN, LOS LABORATORIOS?

Participante: en el laboratorio Clausel

DRA CASTRO: LLAMAN A LA ESCUELA DE MEDICINA PORQUE ME HAN DICHO QUE AHÍ SALEN MAS BARATOS

Participante: ¿Si?

DRA CASTRO: NO SE, ES ALGO QUE ME DIJERON

Participante: y ese es el numero?

DRA CASTRO: NO OK, PUEDES LLAMAR... OK LLAMA A ESTE Y PIDES QUE TE DEN LA EXTENSION NO A ESTE PORQUE LA EXTENCION SON DE MI OFICINA.

Participante: ah ok

DRA CASTRO: PERO ESTE QUE ESTA AQUÍ ES EL NUMERO DE LA ESCUELA. A VER CUANTO TE SALE PORQUE ME HAN DICHO, YO NO SE, PERO ME HAN DICHO QUE ALLA APARENTEMENTE SON MAS BARATOS

Participante: ujum

DRA CASTRO: PERO AVERIGUATE, PORQUE NO ESTOY SEGURA. ESTE ¿ALGUN OTRO LABORATORIO O COSA, REFERIDO QUE TE DA LA DOCTORA QUE SE TE HACE DIFICIL? O SEA COSAS QUE ESTEN FUERA DE TU CONTROL TAMBIÉN. POR EJEMPLO ESO DE LOS DEDUCIBLES SON COSAS QUE ESTAN FUERA DE TU CONTROL. COSAS ASI QUE SE TE HACE DIFICIL ESTE SEGUIR

Participante: no, eso es lo único

DRA CASTRO: ¿ALGÚN OTRO MEDICO? HASTA EL MOMENTO TODOS AQUÍ TE ATIENDEN, LOS MEDICOS QUE ESTAN AQUÍ, ¿NO TE HAN DADO MEDICOS FUERA?

Participante: no, aquí

DRA CASTRO: ESTE Y LAS CITAS ¿VIENES A LAS CITAS O SE TE HA HECHO DIFICIL VENIR A LAS CITAS?

Participante: no, yo vengo a las citas

DRA CASTRO: ¿HAS FALTADO A ALGUNA?

Participante: una o dos

DRA CASTRO: OK

Participante: pero es por la transportación.

DRA CASTRO: AH POR LA TRANSPORTACION.

Participante: pero mientras yo pueda venir yo..

DRA CASTRO: OK ¿CADA CUANTO TE DAN LAS CITAS?

Participante: mensual

DRA CASTRO: ESTE EH ¿HAY ALGO MAS VERDAD DE LO QUE HEMOS HABLADO QUE TU QUIERAS ANADIR QUE A LO MEJOR NO TE HE PREGUNTADO QUE TU ENTIENDAS QUE ES IMPORTANTE QUE YO SEPA O QUE SEPAMOS DE LA EXPERIENCIA TUYA?

Participante: no

DRA CASTRO: OK, ENTONCES DAMO POR TERMINADA LA ENTREVISTA

Participante: gracias

DRA CASTRO: A TI GRACIAS, MUCHAS GRACIAS.

Entrevista #8

Entrevista numero 8

Participante femenina

**Entrevistador: Entrevista número ocho. Ya se le explico el consentimiento informado y la participante accedió a…firmó y está de acuerdo a participar del estudio. Okay, este…me dijiste…te diagnosticaron la condición hace 21 años.**

Participante femenino 8: En el ’92.

**Entrevistador: ‘92, okay.**

Participante femenino 8: Viene siendo ’91 porque yo estaba en cinta de mi nena y vine a coger tratamiento en el ’92.

**Entrevistador: O sea que desde el ’92 estas tomando los medicamentos para el VIH.**

Participante femenino 8: Ujum.

**Entrevistador: Okay. ¿Empezaste a tomarte los medicamentos cuando te diagnosticaron o paso algún tiempo…?**

Participante femenino 8: Estuve 7 meses y después a beberme los medicamentos. Yo estaba en cinta. En aquel tiempo no había medicamento para embarazadas.

**Entrevistador: Okay. O sea que tu nena…**

Participante femenino 8: Mi nena nació con la condición.

**Entrevistador: Okay. Y en el lugar donde te trataban en ese momento, ¿tenían los medicamentos disponibles o había veces que no los tenían?**

Participante femenino 8: Sí, porque yo me atendía la pipa…el embarazo en otro lado y me refirieron para acá.

**Entrevistador: ¿Y siempre tenían los medicamentos? ¿Y lees la información que explican los efectos secundarios del medicamentos, si? ¿Los entiendes o tienes alguna duda?**

Participante femenino 8: Si, entiendo…todo el mundo tiene efectos secundarios diferentes. A todo el mundo no le da los mismos síntomas. Otros le dan empezando mucho tiempo bebiendo con ellos y después al final es que le dan los efectos secundarios.

**Entrevistador: ¿Y qué tú piensas de los medicamentos para el VIH?**

Participante femenino 8: Bueno ahora mismo, el que empiece ahora está hecho.

**Entrevistador: ¿Sí?**

Participante femenino 8: Cuando nosotros empezamos en el 90 pico había que beber 38 o 24 pastillas.

**Entrevistador: Wow.**

Participante femenino 8: Cuando tú ibas por la 16 ya el galillo no aguantaba ya. Sae’, ahora no. Ahora tú te bebes dos veces al día. Antes no, antes era 3 veces al día. Por la mañana, por la tarde y por la noche. Que era el Corsept.

**Entrevistador: Exacto, lo que llamaban el Corsept.**

Participante femenino 8: ‘Chacha, ahora…ahora son…ahora es un guiso.

**Entrevistador: Okay. Wow. ¿Y qué tu esperas del tratamiento? ¿Qué expectativas tienes del tratamiento para el VIH?**

Participante femenino 8: Yo…yo te digo que el tratamiento y la mente puede más que otra cosa. Yo…mis pastillas tienen tres nombres.

**Entrevistador: ¿Cuáles son?**

Participante femenino 8: Pa’ mí son mi vida, porque si yo no me cuido no me va a cuidar nadie. Si yo no me quiero no me quiere nadie. Sabe, una es mi amor, la otra mi vida y te quiero. Porque muchos de nosotros los pacientes, estamos solos. Digo, yo no, yo tengo el apoyo de mi familia, de mis compañeros. Pero aquí vienen muchos pacientes que no tienen apoyo de nadie. Y los medicamentos pues no te hacen el mismo efecto. Y ahí viene la negación de que tuno quieres aceptar la condición. Y para tu entrar en tratamiento tú tienes que aceptar primero que tu eres paciente de HIV, para que los medicamentos te hagan efectos.

**Entrevistador: ¿Y has visto personas así? ¿Te has encontrado con personas que han hecho…?**

Participante femenino 8: Sí, ‘cacho. Hemos visto desde personas jóvenes hasta personas mayores de 70 y 80 años. Y tú le dices que tú tienes la condición, y ellos “¡que va a hacer! Yo no soy paciente, yo tengo 21 años.” Ellos esperan ver otros pacientes que sea…que este llagoso o muriéndose. Porque casi todo son usuarios de drogas, pero también habemos personas que no somos usuarios de drogas y tenemos la condición. Que nos hemos contagiado por…por sexualmente. Y por eso es que tú ves que muchos pacientes, pue’ no aceptan su condición. No se abren a hablar con las personas, con la familia y decirle a la familia “yo tengo esta condición” para tener más apoyo.

**Entrevistador: ¿Y esas personas…muchas de esas personas no se toman a veces los medicamentos?**

Participante femenino 8: Aja. No se toman y no quieren aceptar que tienen que beber medicamentos porque no quieren…no quieren llegar a su casa con tanto medicamento y le van a preguntar “¿y de donde son esos medicamentos?” ¿Y que tú le vas a decir?

**Entrevistador: ¿Tú crees que no se la toman porque no aceptan la…?**

Participante femenino 8: La mayoría es que…o la beben y empiezan los efectos secundarios y le cogen miedo. No se la quieren beber más. Sae’ tienes que darle un tiempo a que el medicamento funcione para que su sistema inmunológico se acostumbre a las pastillas.

**Entrevistador: Y como que no le dan break a eso.**

Participante femenino 8: No porque…no quiere…vienen una vez y después no vienen más. También hay muchas personas que no saben leer que no saben seguir la rutina de las pastillas.

**Entrevistador: ¿Y van donde ti…hay personas que van donde ti? ¿A pedirte apoyo…a preguntarse?**

Participante femenino 8: Nosotros…pues ya yo y Maritza pue’ la Dra. Sepúlveda y los médicos saben quién son las personas que pueden hablar y nos llaman y nosotros rápido venimos y hablamos con ellos. Porque nosotros somos las…la manera más fácil de llegar a un paciente somos nosotros mismos para que ellos vean…

**Entrevistador: Con su experiencia.**

Participante femenino 8: Nosotros pues hemos sobrevivido y ellos pueden sobrevivir también.

**Entrevistador: Ujum, exacto.**

Participante femenino 8: Los otros días yo vine, que me llamo la Dra. Sepúlveda de una paciente que no se quería beber los medicamentos.

**Entrevistador: Ok. ¿Y por qué no se los quería beber ella?**

Participante femenino 8: Por los efectos secundarios. Y porque estaba en depresión y al estar en depresión pue’ los medicamentos no le funcionaban. Y hoy pregunte por ella y me dicen que ella está de lo más bien ya.

**Entrevistador: Ah, qué bueno.**

Participante femenino 8: Al contrario y que me dejo una caja de chocolate por ahí de agradecimiento. Y yo “no olvídate, yo no estoy pendiente a na’ de eso”. A mi me importa que ella se sienta bien.

**Entrevistador: Sí, sí. ¿Y qué es lo más que tú escuchas, verdad, cuando te llaman para que hables con esos pacientes que no se quieren tomar los medicamentos…además de los efectos secundarios?**

Participante femenino 8: No, que se sienten solos, que se sienten culpables porque tienen una condición y piensan que se van a morir. Y nosotros le decimos a ellos que ya no es como antes, que antes tu decías “no ese tiene el mostro, esa se va a morir”. Ahora no, ahora le han cambiado el nombre que ahora el V1 y V2. Que ya no es ni portador ni detectable ni na’ de eso. Ahora es una palabra.

**Entrevistador: ¿Qué los mismos pacientes se lo cambian?**

Participante femenino 8: No eso viene de…de las pruebas cuando salen pue’ ya ahora…yo soy V1, que no tengo la condición. Soy portadora, pero…

**Entrevistador: Esta no detectable.**

Participante femenino 8: Aja, no estoy detectable.

**Entrevistador: ¿Y V2 es cuando están detectables?**

Participante femenino 8: Cuando tienen la condición…no…que la tienen y su sistema inmunológico es débil.

**Entrevistador: Y se detecta entonces.**

Participante femenino 8: Aja. Ahora decimos que se ponga positivo, que la mente puede más que otra cosa.

**Entrevistador: Eso es así.**

Participante femenino 8: Si tú te levantas deprimido, tú vas a estar todo el tiempo decaído.

**Entrevistador: O sea, que ves mucha gente que está deprimiéndose.**

Participante femenino 8: Sí, porque se sienten solos. Se sienten rechazados. Se sienten sucios porque tienen una condición que no se atreven decir…como yo…digo ahora uno lo dice abierta voz porque uno cogió charlas y uno se…pero al principio cuando tú te enteras es un golpe para uno decirte…yo estaba en cinta. Yo llore los seis meses, porque ya yo sabía que mi nena venía con la condición, que le iban a marcar la cuna, yo no era tanto por la nena…yo era por mi familia, por mi mama…por mi familia que cuando se enteraran iban a…Ahora no porque ya gracias a Dios los bebes ahora son bien pocos los que nacen con la condición, y ya no se ven tanto jóvenes ya. Porque ahora hay tratamientos y la ciencia ha ido avanzando muchísimo también.

**Entrevistador: Eso es así. Algunas otras razones que las personas te dicen…además de que están deprimidas…**

Participante femenino 8: De que no tienen carro, que no pueden como llegar…como no pueden llegar a coger sus medicamentos. Porque hay que andar muchos pasos para conseguir los medicamentos. Digo cuando ya tu estas aquí pue’ te los provee Adapt pero cuando las personas no están en una clínica que tienen que ir a su médico primario, de su médico primario tienen que pasar a otro sitio. Se le hace muy cuesta arriba conseguir sus medicamentos.

**Entrevistador: ¿Y eso está pasando todavía ahora?**

Participante femenino 8: Seguro.

**Entrevistador: ¿Aun con todas las ayudas se les hace cuesta arriba?**

Participante femenino 8: Sí, porque los medicamentos de la condición son bien costosos y la reforma no los cubre.

**Entrevistador: ¿No los cubre todos?**

Participante femenino 8: No los cubre todos. Cubre algunos pero no todos.

**Entrevistador: Y cuando necesitan alguno…**

Participante femenino 8: Ah, porque entonces está el programa Adapt, esta Ryan White Parte A, que entonces ellos te dan…te proveen los medicamentos. Depende de donde estés cogiendo servicios.

**Entrevistador: ¿Y en algún momento tú has dejado de tomarte los medicamentos?**

Participante femenino 8: Sí.

**Entrevistador: ¿Eso fue cuándo? ¿Hace cuánto tiempo de eso?**

Participante femenino 8: Yo estuve dos años sin beber medicamentos por…yo deje de beber medicamentos en el 2006. A mí me dieron unas diarreas que no se me quitaban. Yo estuve 4 años con unas diarreas.

**Entrevistador: Wow. ¿De todos los días?**

Participante femenino 8: De todos los días. Comía y todo me caía mal. Corrí todos los gastro de Ponce. Y me quitaron los medicamentos…deje…yo misma deje de beber los medicamentos.

**Entrevistador: ¿Estuviste dos años sin tomártelos?**

Participante femenino 8: Estuve 6 meses sin…sola. Y después el médico me los quitaron.

**Entrevistador: Ah okay.**

Participante femenino 8: Ah, y entonces lo que me dieron medicamentos nuevos a lo que el cuerpo…y eso son los medicamentos que estoy bebiendo ahora, que son los que le dan a las embarazadas.

**Entrevistador: Ah, okay.**

Participante femenino 8: Y me he quedao’ con ellos.

**Entrevistador: ¿Y esos te han funcionado entonces?**

Participante femenino 8: Bueno, hasta el sol de hoy.

**Entrevistador: ¿Cuánto tiempo llevas con esos medicamentos?**

Participante femenino 8: Llevo…desde el 2006.

**Entrevistador: O sea, que ya llevas más de 6…7 años van…para 7 años.**

Participante femenino 8: Sí.

**Entrevistador: Y entonces, ¿en ese momento, cuando te los dejaste de tomar en el 2006 era por los efectos secundarios? O era una…**

Participante femenino 8: Tengo una bacteria por la condición HIV por los medicamentos y por…me dieron Caletra y al darme Caletra, el Caletra tiene mucho por ciento de alcohol. Y me activó más la bacteria. Y el Norvil tiene mucho por ciento de alcohol, pue’ me los quitaron. Me dieron el Fusion que era inyectable. Pero me dio un anormolia y me lo quitaron.

**Entrevistador: Okay. ¿Y en esos seis meses que estuviste primero, que tú te los dejaste de tomar, tuviste algún efecto…te sentiste…sentiste que empeoro algo…?**

Participante femenino 8: Si, me empeore. Yo estuve seis meses sin las…yo entraba y salía del hospital. Yo salía hoy y a los dos días regresaba para el hospital.

**Entrevistador: ¿Por las diarreas o por otra cosas?**

Participante femenino 8: Por las diarreas y lo que no eran las diarreas. Entonces en lo que estaba en sala de emergencia, a lo que me daban cuarto. Tenía que llamar al procurador del paciente para que me consiguieran cuarto. Porque rápido te mandan pa’ aislamiento y…y te cansas de llamar y no aparece nadie.

**Entrevistador: ¿Cómo te trataron en todo eso?**

Participante femenino 8: Malísimo. Malísimo. Tuve que hacer como tres querellas.

**Entrevistador: ¿De verdad?**

Participante femenino 8: Estuve una semana en sala de emergencia sin bañarme y sin nada.

**Entrevistador: ¿Aquí en Ponce?**

Participante femenino 8: Aquí en el distrito. Me bañé porque llegó una enfermera que el esposo tenía la condición y me ayudó a conseguir cuarto. Y me consiguieron donde bañarme. Pero estuve una semana con la misma ropa.

**Entrevistador: Wow. Y alguna razón por la cual no…**

Participante femenino 8: Por que las enfermeras que estaban no querían bregar con paciente de HIV.

**Entrevistador: Wow. ¿Todavía entonces pasan esas cosas?**

Participante femenino 8: Todavía.

**Entrevistador: Y además de esa vez en el 2006, ¿alguna otra ocasión que habías dejado de tomártelas?**

Participante femenino 8: No.

**Entrevistador: ¿No? Y por ejemplo, a veces uno como que piensa dejarlos, aunque no los deje, uno piensa dejarlos. ¿Te ha pasado eso?**

Participante femenino 8: A veces…a veces por…a veces se le viene a la mente a uno pero por…tiene que seguir bebiéndotelos.

**Entrevistador: Claro que sí.**

Participante femenino 8: De esos días que se levanta uno que no se los quiere beber. Pero uno viene al rato y se los bebe otra vez.

**Entrevistador: Yo te voy a hacer…a perdóname. Entonces además de eso, verdad, por ejemplo, dosis de los medicamentos, alguna dosis que se te haya olvidado.**

Participante femenino 8: Hasta el sol de hoy nunca.

**Entrevistador: ¿Te bebes todos…que se te olvide alguna o qué?**

Participante femenino 8: No, porque yo tengo un potecito…como dice mi nieto con el sol y la luna. Los de dia y los de la noche. Y a veces estoy jugando con ellos, “mamá, ¿te bebiste las pastillas de por la noche?” y yo digo “sí, Papi.”

**Entrevistador: Que ya eso tú lo tienes como una rutina.**

Participante femenino 8: Ujum.

**Entrevistador: ¿Y antes de eso que paso en el 2006, te pasaba, que se te olvidaban alguno?**

Participante femenino 8: A veces se me olvidan, porque como estaba trabajando y con el estrés y los muchachos y las cosas, pue’…

**Entrevistador: Se te olvidaba alguna de las dosis. Ahora yo te voy a hacer unas preguntas con relación a esas vez que se te…que no te estuviste tomando el medicamento, pero también me gustaría saber…me gustaría que pienses también en los momentos en que pensaste en dejar de tomarlos. Aunque te los tomaste, pero pensaste en dejar de tomarlos. La razón es, porque a veces hay personas que le puede pasar como tú, que a veces piensan en dejar de tomarlos, pero yo quiero saber lo hacen pensar en dejar de tomarlo, porque tú te las tomas, pero hay personas empiezan pensándolo y terminan haciéndolo. Entonces es como para…para ver como entonces nosotros podemos ayudar. Te voy a ser, verdad, unas preguntas sobre algunas razones por las cuales tú hayas dejado de tomarte los medicamentos…verdad, esas posibles razones. Antes que nada…¿me gustaría saber cómo tú te describes, como tú eres, que características tú tienes, tu como persona?**

Participante femenino 8: Yo tengo mis momentos tristes y mis momentos alegres.

**Entrevistador: Y en esos momentos tristes, ¿cómo eres?**

Participante femenino 8: Bueno…ya no soy tan explosiva como antes. Antes era más explosiva. Antes reventaba como fosforo. Pero ahora no…ahora me da coraje pero me quedo tranquila. No digo nada. Mejor me aguanto para no explotar como dice uno.

**Entrevistador: Y entonces cuando estas en los momentos alegres, ¿cómo eres?**

Participante femenino 8: Simpática, vacilo, relajo como dice uno. Digo no con todo el mundo, porque tengo mi gente, como dice uno.

**Entrevistador: Ahora yo quiero que pienses en ti, sin incluir a otras personas, otras circunstancias. ¿Consideras que tengas algún sentimiento, o hayas tenido algún sentimiento o pensamiento o creencia personal que influyó en ese momento que dejaste de tomar los medicamentos? Algún pensamiento que tuviste…**

Participante femenino 8: No, porque me sentía mal y el cuerpo de uno sabe cuándo el medicamento está funcionando y cuando el medicamento no está funcionando. Y yo, para mí, el medicamento no estaba funcionando.

**Entrevistador: Y entonces en esos…y ahora, verdad, quiero que hagas el ejercicio un poquito más allá, las veces que tú has pensando en dejar de tomártelos, algo por ejemplo, algún sentimiento, pensamiento que haya surgido en ti…**

Participante femenino 8: Porque estoy cansada de beber tanto medicamento.

**Entrevistador: Ok. Que piensas que…eso es bien común, verdad. Muchas personas le pasa eso, que se…como que se cansan, piensan que ya están tomando tanto.**

Participante femenino 8: Digo, ya no tomo como antes tanto, pero estamos cansados de beber tanto medicamento. Me pregunta a mí, le va a preguntar a todo paciente y le van a decir lo mismo.

**Entrevistador: Lo mismo, es verdad. Me han dicho eso, mucho. O sea, que cuando piensas es que te cansas, ya te cansa en algún momento. Eso yo lo he escuchado. Y cuando en esos momentos cuando piensas, lo piensas, yo sé que no lo haces, pero te pasa por la mente, te sientes de alguna manera, estas en tus momentos tristes o alegres, como tu te sientes?**

Participante femenino 8: Me pasa más cuando estoy triste, porque yo, tú me ves así, pero yo estoy pasando la muerte de mi nene. A mi nene me lo mataron. Recuerda que yo había hablado mucho con Julio Jiménez que ha venido bastante…ya estoy tranquila ya, porque ya…es un dolor que…llevo dos años, pero es un dolor, como dice Julio Jiménez, que nunca se va a acabar. Pero después le digo que me de fuerzas para echar para adelante porque me quedan mi nena y nietas, mis nietos. Jamás pensé que iba a ver a mi nieta de 15 años y estuve con ella en el quinceañero. Pero lo demás.

**Entrevistador: Lo siento mucho.**

Participante femenino 8: Ah, no, está bien. Ya…

**Entrevistador: O sea, que te pasa cuando estas en los momentos tristes.**

Participante femenino 8: Ujum.

**Entrevistador: Y además, verdad, hay otras cosas que tú piensas, que te vienen a la mente, que después terminas diciendo “ay estoy cansa’ de…”**

Participante femenino 8: Por eso, porque uno se siente…uno trabajaba y al uno perder su trabajo por la condición también pue’ uno se siente no capaz para no hacer las…digo no es que no seamos capaz, pero por tener una condición médica hemos perdido nuestro trabajo.

**Entrevistador: ¿Lo perdiste tú?**

Participante femenino 8: Sí, yo perdí mi trabajo.

**Entrevistador: ¿En qué trabajabas?**

Participante femenino 8: Yo trabajaba en una…en Villalba en una…yo era como la mano…la handyman de la casa, yo era la que limpiaba, la que iba a hacer compra, y me sentía por lo menos, en esas seis horitas que trabajaba, me sentía un poquito más…mas…más útil. Pues ahora me entretengo con las nenas ahora, la mai trabaja y pue’ le voy a las citas de las nenas. Todo el mundo piensa que son mías.

**Entrevistador: Sí, sigues siendo útil ahí.**

Participante femenino 8: Por eso, estoy siendo útil pero me gustaría trabajar y…ganar como todo mundo sus chavos y no depender de otras personas.

**Entrevistador: Sí. Y qué… ¿por qué no puedes trabajar en estos momentos?**

Participante femenino 8: Porque yo tengo las diarreas y estoy más en el baño que otro sitio y pue’…esta así no me reporté a trabajar al tiempo que me tocaba y me despidieron.

**Entrevistador: Ah, okay. Wow. ¿Ya de las diarreas estas mejor?**

Participante femenino 8: Ya…me dan, pero sé que puedo comer y que no puedo comer para que no se me activen más rápido cuando estoy en la calle.

**Entrevistador: Si, por que es la bacteria.**

Participante femenino 8: Ujum.

**Entrevistador: Okay. Ahora vamos a hablar de las personas con las que tú te relaciones, verdad, las personas cercanas a ti, familiares, amistades, todo eso. ¿De qué manera, si alguna, las personas con las que tú te relacionas en aquel momento en el 2006 influyeron en dejar de tomar los medicamentos?**

Participante femenino 8: Si yo me dejara llevar por mi mamá nunca me hubiera tomado los medicamentos.

**Entrevistador: ¿No? ¿Por qué?**

Participante femenino 8: Pue’ porque en aquel tiempo, mi mama tiene 70 años, mi mama es una persona mayor que es difícil explicarle a ella que tú tienes una condición médica. En aquel tiempo yo tenía un vaso aparte, todas mis cosas aparte, y ahora ya yo como en el plato que come todo el mundo. Ella me llamaba para acordarme los medicamentos y yo “no me llames para eso que me vas a volver loca”.

**Entrevistador: ¿Y las veces que tú has pensando en, o sea, que piensas en dejar de tomarlos, alguna de esas veces alguna personas cercana a ti, algo que haya pasado con alguna persona, de alguna manera te…?**

Participante femenino 8: No, porque mi…por lo menor yo tengo el apoyo de mi familia. Mucha gente no tiene el apoyo de nadie. Están solos, tienen que beberse los medicamentos a escondidas, tienen que hacer todo a escondido.

**Entrevistador: ¿Alguna amistad, por ejemplo, que a lo mejor de alguna manera…?**

Participante femenino 8: Ah, no porque tú siempre te vas a encontrar gente negativa.

**Entrevistador: ¿Has encontrado gente así, que te dice…?**

Participante femenino 8: Seguro, que tú tienes la condición y ya tu pa’ ellos ya no vales nada.

**Entrevistador: ¿De verdad?**

Participante femenino 8: Sí, mucha gente…pero yo no, ellos han visto la otra cara que uno le ha demostrado, como Marisa y yo decimos. No somos un billete de 100 para caerte bien, para que nos acepte. Tú me tienes que conocer, no te puedes dejar llevar porque me veas seria, “ah esa tipa es una sangrigorda”. Tú me tienes que conocer y darme la oportunidad de yo compartir contigo palabra. Cuando yo empecé con Julio Jiménez, pue’ yo empecé en un sitio donde todos eran profesionales y yo decía “pero como yo voy a hablar aquí si aquí to’ el mundo habla perfecto”. Y yo a veces, creo que fui conociendo par de gente y cuando quería decir una pregunta “mira, como yo puedo hacer esta pregunta que no se sienta que yo estoy siendo abruptamente…explosiva” como dice uno, que a veces tú piensa “pues me salió así del alma y lo dije”. Poco a poco uno, “no, yo quiero que tú seas así, porque así nosotros vamos conociendo la forma de los pacientes, como los pacientes se sienten, como…eso es lo que yo estoy buscando, como ustedes se desenvuelven hablando. No porque ya, nosotros tenemos estudios y ya, pue’, y de vez en cuando yo también hablo como ustedes también”, dice él. Y nos dio esa confianza de sentirnos libremente como hablar con él.

**Entrevistador: Que bueno. Y una pregunta, ¿todas esas personas que tú has ayudado, cuando te llaman, verdad, que no se quieren tomar los medicamentos, ellos te dan alguna…alguna de las razones que te dan tienen que ver con quizás personas con quienes…familiares, amistades, que no…?**

Participante femenino 8: Por eso te digo, la familia, muchos de ellos, pue’ por no aceptarlo pue’ se han muerto.

**Entrevistador: ¿Sí? ¿Por no aceptarlo? Y por ejemplo, ¿que situaciones tú has escuchado de que por algún familiar o por alguna persona no se toman los medicamentos?**

Participante femenino 8: Pue’ porque, la familia le dice que “por tu culpa, por esta jod…por estar haciendo las cosas que hacías en la calle pue’ te los buscaste”.

**Entrevistador: Okay. O sea, que por echarle la culpa, por la familia la persona entonces deja de tomárselos.**

Participante femenino 8: Ujum.

**Entrevistador: Y de amistades, por ejemplo.**

Participante femenino 8: Pue’ también, porque también muchas personas son homosexuales, tienen otras preferencias sexuales, y la familia no los aceptan. Se sienten cohibidos para ser ellos mismos.

**Entrevistador: Y por ejemplo, ¿los médicos que…de los médicos que la atienden, por ejemplo, tanto a ti como a esas otras personas…?**

Participante femenino 8: Bueno, de aquí nosotros tenemos 3. Aquí hay 4 médicos. De los 4 médicos hay dos médicos que tú te puedes sentar con ellos a hablar y tu ser tu pana del médico. Hay otros no, hay otros que tú llegas aquí y ya te tienen la receta ya. No te preguntan cómo tú te sientes, ellos se ponen a nivel tuyo de decirte…te buscan la manera de que no dejes de beberte los medicamentos. Porque lo primordial aquí es que tu cojas tratamiento. Para que tu carga viral siga mejorando poco a poco. Porque de la noche a la mañana no va a mejoral. Tienes que darle tiempo, meses…veas el efecto de las pastillas.

**Entrevistador: Y en el caso tuyo, por ejemplo, ¿en algún momento que algún medico te trate de cierta forma, te…ha hecho pensar como que “ay, ya no me los voy a tomar”?**

Participante femenino 8: Ah, no, porque yo me paro y me voy. Yo se lo digo, yo soy tan fea como tan franca. Si yo…me atiende un medico algo que no me desto, mira…deme mi record, que me atiende otra persona.

**Entrevistador: ¿Si?**

Participante femenino 8: Yo vengo aquí ya, y yo…ya yo tengo dos médicos aquí que me pueden atender. Si yo llego y pregunto “¿esta fulana, esta fulano? ¿Están? A pue’ me quedo. Si no están, pue’, denme otra cita que me voy.” Sae’, yo no me quedo. Otros no, otros se quedan porque, “pue’ ese me da lo que yo quiera y yo pue’…” ¿Me entiende?

**Entrevistador: ¿Y en ese tiempo de cambiar las cita, te has quedado sin algunos medicamentos?**

Participante femenino 8: Ah, no, porque gracias a Dios, pue’ aquí está Parmel, está las enfermeras que son bien…las mismas…sae’ uno ya dicen “mira ya y te consiguen la receta”. Si no llamo a farmacia y digo “mira no tengo medicamento” y a lo que llego a la farmacia me resuelven y nos resuelven. Pero tú tienes que buscar la forma de cómo conseguir los medicamentos, pero otros no, se te acabaron, “ah pue’ yo voy a mi próxima cita que me toca en tres meses”. Y en esos tres meses que pasan…

**Entrevistador: Que no hacen la gestión.**

Participante femenino 8: Ujum.

**Entrevistador: ¿Y tú has escuchado de alguno de ellos de que porque no le guste como un medico lo atienda pue’ entonces dejan de venir…?**

Participante femenino 8: Sí, se van a atenderse a otro sitio.

**Entrevistador: Okay. ¿Y…o dejan de tomarse los medicamentos?**

Participante femenino 8: Por eso, o vienen aquí porque pue’ hay ayudas y cosas y…pero no se beben los medicamentos. Y…tú me preguntas y digo “sí, yo me los estoy bebiendo” y quién sabe si…que tú te los está bebiendo. Porque ya no hay una prueba como antes que te decían que tú no te bebes los medicamentos. Eso viene más pa’l protocolo. Pero así yo te puedo decir “yo vengo aquí” y pue’ las boto. Y no me las bebo

**Entrevistador: ¿Y has escuchado de gente que hace eso?**

Participante femenino 8: Seguro.

**Entrevistador: Y usualmente lo hacen porque no…**

Participante femenino 8: Y otros que están en protocolo porque le pagan y no se beben los medicamentos. El que hizo la trampa hizo la ley.

**Entrevistador: Eso es así. Y por ejemplo, verdad, ahora quiero que pienses en el ambiente donde tú te desenvuelves, donde tu estas, en donde tu estas…tu comunidad, en donde tú te desenvuelves. ¿De alguna manera ese ambiente influyo en la decisión en aquel momento en el 2006…?**

Participante femenino 8: No, porque mira, yo vivo…yo viví, yo vivía en una escalera, éramos 8 apartamentos.

**Entrevistador: ¿Como que en una escalera?**

Participante femenino 8: En un edificio. Éramos 4 y 4, 4 vecinos, pue’ yo los enseñe a ellos que el primero de diciembre todo el mundo se vestía de rojo, yo les fui enseñando las otras cosas de…aunque fueran casados yo le decía “no, te tienes que cuidar, tienes que hacer esto” y yo fui haciendo como una rutina y ellos vieron que yo con mi condición y todo pue’ yo le decía a ellos “cuando ustedes me ven en mi casa deprimida y yo este que yo no salga ustedes van y me tumban la puerta y me sacan de ahí a las millas” y les fui enseñando pero muchas personas, pue’ están solos tu entiendes, que la familia…ellos quisieran tener mucho apoyo de una persona y no la tienen. No se sienten en esa confianza. Aquí pue’ viene mucha gente que va pa’ otro sitio, aquí viene mucha gente de muchos pueblos diferentes. Porque no se quieren atender en su pueblo.

**Entrevistador: VPor miedo a que los vean?**

Participante femenino 8: Sí, porque antes no, antes tu venia aquí en un carro…en carro público de CREA y el tró estaba identificado de CREA. Ahora no, ahora hay un taxi. Ahora tú te montas como si te montaras en un taxi.

**Entrevistador: Por ejemplo, verdad, bueno cuando trabajas, en aquel momento cuando trabajabas, ¿de alguna…en algún momento, por ejemplo que…?**

Participante femenino 8: No, yo me tenía que beber las pastillas a escondidas.

**Entrevistador: ¿Te las tomabas a escondidas? ¿Y en algún momento no te las tomabas por el hecho de que…?**

Participante femenino 8: No me las podía beber porque estaba el personal, mi jefe, mis personas…que me mandaban más que yo. Y tenía que llevármelas a escondidas pa’ bebérmelas. Y a veces se pasaban las horas y me las bebía después.

**Entrevistador: Y en el ambiente donde tu socializas, o como dicen por ahí, que jangeas o algo así, ¿de alguna manera eso influye en que no te las tomas?**

Participante femenino 8: No, porque yo deje la calle, deje todo eso ya, tu entiendes? Yo cuando me enteré de la condición pue’ cambie mi rutina de antes. Y me tenía que adaptar a que yo me tenía que cuidar. Si yo no me cuido no me cuida nadie. Y fui acostumbrando a mis hijas y a todo el mundo y ellos vieron que yo poco a poco fui cambiando. Y ahora pues…no soy la mama perfecta, verdad, pero pue’.

**Entrevistador: Estas ahí luchando. Y en el lugar donde recibes los servicios médicos, por alguna manera…de alguna manera, verdad, de la manera en cómo te traten o algo del lugar que influya en que no…?**

Participante femenino 8: No, por eso, porque aquí…por lo menos aquí tú te sientes cómodo. Y muchos pacientes que vienen aquí se sienten cómodos. Y aquí muchas de las enfermeras cuando te ven deprimido rápido te preguntan que “qué te pasa” y te buscan alternativas. No te dejan…no te dejan sin buscar alternativas.

**Entrevistador: Algún otro lugar que tu creas que influye en que una persona se deje de tomar los medicamentos.**

Participante femenino 8: Por eso, el ambiente que estén atendiéndose pue’ es muy deprimente en algunos sitios.

**Entrevistador: ¿Cómo que deprimente?**

Participante femenino 8: Bueno, pue’ la fachada, por la gente que se atienda.

**Entrevistador: Y tú crees que eso desanime…**

Participante femenino 8: Seguro, como los deambulantes. Los deambulantes no pueden beber medicamentos porque están deambulando en la calle. La desto droga pue’ prefiere inyectarse droga y dejar de beberse las pastillas. Porque con la droga se sienten que no le duele ni una uña.

**Entrevistador: Y cuando se sienten así, la gente no se los toma.**

Participante femenino 8: Por eso. Porque estas en el ambiente del embollo.

**Entrevistador: Sabes que, ahora que tu mencionas eso, yo escuchado de personas que dicen que cuando…**

Participante femenino 8: Que prefieren usar droga que beber medicamentos.

**Entrevistador: Sí, no, y aparte de eso, como se sienten bien a veces no se quieren tomar los medicamentos.**

Participante femenino 8: Por eso, si salgo no detectable para que me tengo que beber los medicamentos si estoy no detectable.

**Entrevistador: Sí. Pero entonces cuando empiezan a tener los…cuando ya no se los toman, que tienen entonces las complicaciones y todo eso, pues ahí es que lo… ¿Tú has escuchado eso mucho también?**

Participante femenino 8: Seguro. Aquí tu oyes de todo, muchacha.

**Entrevistador: ¿Si?**

Participante femenino 8: Hasta de sueños eróticos, de todo tu oyes aquí.

**Entrevistador: ¿Qué le dan con los medicamentos?**

Participante femenino 8: Seguro.

**Entrevistador: ¿Y por eso no se los toman?**

Participante femenino 8: Por eso, porque hay muchos efectos, hay muchos efectos que a todo el mundo le da diferente. A todo el mundo no le da igual. Todo los medicamentos tiene…todo medicamento para la condición HIV tiene efecto secundarios. Unos más que otros menos, pero todos tienen…

**Entrevistador: Y depende de la persona también, ¿verdad?**

Participante femenino 8: Por eso, si te coge una persona que pue’, te da más síntomas diferentes.

**Entrevistador: Ahora yo quiero que tú pienses en Puerto Rico. Verdad, Puerto Rico, las cosas que están pasando aquí, tu estas familiarizada porque tu estas en el comité, verdad, y tú sabes más o menos lo que está pasando a nivel...de unas ayudas y unos proyectos aquí en Puerto Rico. Así que con tu experiencia, me gustaría saber, verdad, si de alguna manera las cosas que pasan en Puerto Rico, influyen en la decisión de algunas personas en dejar de tomarse los medicamentos.**

Participante femenino 8: Seguro. En el ambiente en que tú te críes, en el ambiente que tu estés sintiéndote, en la forma…si son usuarios de droga, pue’ están en ese ambiente. Si son deambulante, pue’ están en su mundo y dejan de beberse los…nunca han medicamento. Muchos usuarios de droga nunca han bebido, igual que los deambulantes. No tienen papeles, no tienen nada.

**Entrevistador: Que no buscan el servicio.**

Participante femenino 8: Como una persona que no tenga papeles, que no sepa leer donde va a buscar papeles. Si se les hace bien…es a veces uno que está bien y se te pierde la tarjeta de seguro social y tú sabes to’ los pasos que tú tienes que andar para conseguir una tarjeta…un duplicado de la tarjeta de seguro social.

**Entrevistador: Y todo eso te lo piden para…**

Participante femenino 8: Para todo. Porque para tu hacerte una revisión de la Reforma tú tienes que llevar cuchucientos papeles.

**Entrevistador: O sea, que tú crees que muchas veces es que las personas no andan los pasos para tener…**

Participante femenino 8: Que tienes que andar muchos pasos para…

**Entrevistador: ¿Son muchos?**

Participante femenino 8: Para tu conseguir una reforma, tú tienes que llevar unos requisitos de papeles.

**Entrevistador: ¿Y se le hace difícil a la gente? Y para solicitar…**

Participante femenino 8: Mas tienes que llevar un papel de tu condición.

**Entrevistador: ¿Y para solicitar los servicios aquí, por ejemplo?**

Participante femenino 8: Ah, no, porque aquí tu vienes y sacas cita y te dan cita…rápidamente te dan cita, te consiguen todo. Aquí te sacan la sangre, aquí te hacen todo.

**Entrevistador: Y por ejemplo, ¿tu primera vez que vienes, te piden muchos documentos para…antes de darte una cita, te piden documentos, o no?**

Participante femenino 8: ¿No? Tú lo que tienes que traer es una carga viral y un CD4. Si no lo tienes, te la hacen.

**Entrevistador: Okay. Y si no tienes plan médico.**

Participante femenino 8: También te la hacen, también.

**Entrevistador: ¿Te hacen esas gestiones? ¿Te ayudan?**

Participante femenino 8: Ujum.

**Entrevistador: Oye, pero que chévere, que bueno. Me gusta eso. Como tu…los servicios de salud del país, ¿cómo tú crees, verdad…?**

Participante femenino 8: Algunos buenos y otros malos.

**Entrevistador: ¿Y de alguna manera tú crees que piensas que de alguna manera eso puede influir en que las personas no se puedan tomar los medicamentos, no tengan medicamento?**

Participante femenino 8: No, porque la Reforma no te los cubre. Y los planes médicos privados tampoco.

**Entrevistador: Eso te iba preguntar, ¿los planes médicos…?**

Participante femenino 8: Hay muchos que te ponen muchas tramas porque el medicamento es demasiao’ costoso. Estamos hablando de medicamentos de 600 y 800 pesos. Hasta de 3 mil pesos. Todos los meses. Porque tú te los tienes que beber todos los meses.

**Entrevistador: ¿Y has escuchado historias de personas que han tenido problemas con el plan médico, que has escuchado?**

Participante femenino 8: Seguro, que el deducible de los planes es demasiao’ cuesta arriba para pagar.

**Entrevistador: ¿Y cuando el deducible no le cubre, tienen alguna otra ayuda que le pueda…?**

Participante femenino 8: No, porque tú tienes un plan privado, no tienes… ¿dónde más vas a tener? Si no tienes…no cualificas para la Reforma. Porque tienes un plan privado. Y ya gastaste la dona, ya.

**Entrevistador: ¿Y transportación, por ejemplo, has escuchado…?**

Participante femenino 8: También, mucha gente también está a pie, y no tienen carro. Vienen en pon, como dice mucha gente. En bicicleta, en pon. Y después de aquí tienes que ir a la farmacia a buscar los medicamentos.

**Entrevistador: O sea, que esa situación la has escuchado.**

Participante femenino 8: Tuvimos un tiempo sin…muchas farmacias no te dan los medicamentos de la condición también.

**Entrevistador: ¿No los tienen la farmacia?**

Participante femenino 8: No, son muchas…son bien pocas las farmacias que tienen los medicamentos de la condición215.

**Entrevistador: ¿Por qué estará pasando eso?**

Participante femenino 8: Pue’, como los medicamentos son tan caros. Las farmacias tienen que comprarlos para que entonces el plan se los apruebe.

**Entrevistador: Si, exacto. Que tienen que tener su inventario, eso les cuesta dinero a ellos. Y a ustedes le dan una lista de las farmacias que si tienen disponible el plan médico, ustedes…**

Participante femenino 8: No, yo no tengo problema porque yo los cojo aquí. Yo los cojo por Adapt. Pero mucha gente los coge en el hipotecario. Que es en la Rambla. Algunos de ello, no todos, pero algunos. Otros tienen que ir a la farmacia del pueblo que te toque.

**Entrevistador: ¿Alguna situación que haya en el país que tú crees que influye en, verdad, de alguna manera en que la persona no tenga sus medicamentos a tiempo, que no tenga los servicios a tiempo…?**

Participante femenino 8: También, porque los fondos de las propuestas se acaban. Esto es por un cierto tiempo los medicamentos. Cuando Adapt tiene que renovar para el año que viene para poder tener los medicamentos, Ryan White también.

**Entrevistador: Y cuando pasa eso, que no llegan…**

Participante femenino 8: Pues nos quedamos un cierto tiempo sin medicamentos, a lo que otro…digo nos resuelven rápidamente, pero estamos a veces dos o tres días sin beber medicamentos.

**Entrevistador: ¿Si? O sea, que eso ha pasado. ¿Te ha pasado a ti?**

Participante femenino 8: Bueno, a mí me paso una sola vez, pero Marisa me resolvió.

**Entrevistador: Ah, bueno. Okay. Qué bueno tener, verdad, alguien que lo apoye. Y a otras personas, ¿has escuchado que pase?**

Participante femenino 8: Sí. ¿Falta mucho eso?

**Entrevistador: No, no. Tú tienes que irte.**

Participante femenino 8: Tengo que irme a las y 10, que tengo que ir a buscar a mi nena que sale del trabajo.

**Entrevistador: Vamos a terminarlo ahora. Lo que necesito es, bueno…**

Participante femenino 8: Dame el papelito para ir llenándolo.

**Entrevistador: Okay. Me hablaste orita de discriminación, verdad, ¿en algún momento te has sentido discriminada…?**

Participante femenino 8: Seguro.

**Entrevistador: ¿Si? Y eso ha influenciado en que pienses en dejar de tomarte los medicamentos?**

Participante femenino 8: Te he dicho que sí, que a veces de vez en cuando me siento, me siento así.

**Entrevistador: ¿Y eso ha influenciado a que pienses en dejar…?**

Participante femenino 8: Seguro.

**Entrevistador: Y por ejemplo, verdad, ¿algunas creencias religiosas que pueda tener la gente…?**

Participante femenino 8: Bueno yo siempre, uno siempre cree en Dios en su manera diferente, pero…

**Entrevistador: Ujum…Okay. Los tiempos que estamos viviendo ahora mismo, verdad, de qué manera si alguna estos tiempos que estamos viviendo puede influenciar en la decisión de otras personas en dejar de tomarse los medicamentos.**

Participante femenino 8: Como se sientan. Como tengan el apoyo de su familia. Si no tiene n apoyo pues ellos se…aunque tengan apoyo se sienten que no tienen apoyo. Porque no se sienten completos.

**Entrevistador: ¿Qué fue lo que te ayudo cuando tú dejaste de tomarte los medicamentos, que fue lo que te ayudo a volver a tomártelos?**

Participante femenino 8: Porque me sentía mal, me sentía…mi cuerpo ya me estaba pidiendo de que necesitaba beber medicamentos.

**Entrevistador: O sea, cuando empezaste a ver los síntomas del algunas…**

Participante femenino 8: Si, de que mi…mi carga viral empezó a…

**Entrevistador: A bajar, entonces te sentiste debilitada, ¿pues ahí empezaste?**

Participante femenino 8: Ujum.

**Entrevistador: Okay. O sea que fue como tener esos síntomas.**

Participante femenino 8: Sí.

**Entrevistador: ¿Algo más que te haya ayudado?**

Participante femenino 8: La mente.

**Entrevistador: ¿Si? ¿La mente?**

Participante femenino 8: La mente es lo que te sienta como tú sientes.

**Entrevistador: ¿Y qué le dirías a una persona que está pensando en dejar de tomar los medicamentos? Cuando tu vez esas personas, ¿qué tu…?**

Participante femenino 8: Que tienen que quererse ellos mismo para que beban medicamentos. Para que se sientan bien.

**Entrevistador: ¿Algo más que le dices a ellos?**

Participante femenino 8: Que nos vean a nosotros como ejemplo. Que tenemos 21 años con la condición y no tenemos que bajar nuestra cabeza para nada. Que el tener la condición HIV no es sentirse culpable por nada. Esto es como si tú tuvieras cáncer, o tuvieras diabetes o tuvieras cualquier enfermedad. Pero que todo el tiempo positivamente. Si no estás positivo, no vas para ningún lado.

**Entrevistador: Cuando tu escuchas la frase, verdad, “cumplir con las recomendaciones médicas”, ¿con que tú la relacionas?**

Participante femenino 8: Con la confianza que tú tengas con tu médico. Como tú te sientas, como…hablando con tu médico. Tu medico es la mejor que te puede decir “te está funcionando” o “o te está funcionando”.

**Entrevistador: Además de tomarte los medicamentos, ¿qué otras cosas te recomienda el medico?**

Participante femenino 8: Que no me descuide, que en ningún momento me descuide.

**Entrevistador: ¿Te manda a hacer, por ejemplo, pruebas…?**

Participante femenino 8: Sí, me mandan a hacer CD4, carga viral…

**Entrevistador: Alguna…**

Participante femenino 8: Las otras pruebas adicionales que te hagas afuera.

**Entrevistador: Por ejemplo, de…**

Participante femenino 8: Prueba…que siempre tienes que traer tu prueba de cáncer, tu mamografía. Si tienes otras condiciones tienes que traer tus laboratorios de…

**Entrevistador: ¿Tienes otras condiciones?**

Participante femenino 8: Ah, no sí. Yo tengo unas cuantas, muchacha.

**Entrevistador: ¿Si? Y algunas de esas recomendaciones o análisis que te manda a hacer el médico, ¿se te hace difícil seguirlos?**

Participante femenino 8: No, ya yo estoy acostumbrada no porque yo…he hecho una rutina, con mi condición.

**Entrevistador: Por ejemplo, verdad, la mamografía, ¿te las ha hecho?**

Participante femenino 8: Si, ya por requisito, todo paciente de HIV tiene que hacerse su Papa Nicolao y sus mamografías.

**Entrevistador: ¿Y te has hecho el Papa Nicolao?**

Participante femenino 8: Si.

**Entrevistador: ¿Cuándo fue la última vez?**

Participante femenino 8: Yo me lo hice en noviembre.

**Entrevistador: Okay.**

Participante femenino 8: Me toca cada seis meses.

**Entrevistador: Las citas médicas, a veces…**

Participante femenino 8: Cada dos meses tenemos nuestras citas médicas.

**Entrevistador: ¿Se te hace difícil a veces?**

Participante femenino 8: No, porque aquí nos dan una tarjeta y tu…tu sabe, una tarjeta de citas y ya tú la tienes ya.

**Entrevistador: ¿Alguna de ellas has faltado?**

Participante femenino 8: Digo, hasta ahora falte solamente a una cita, pero fue por la muerte de mi hijo. Pero después que me sentí, vine. Después que pase el sepelio y todo pue’ vine.

**Entrevistador: Hay algo más que quieras añadir sobre el tema, que hemos hablado.**

Participante femenino 8: No.

**Entrevistador: Okay. Pues con esto terminamos entonces.**

Entrevista número nueve

ENTREVISTADOR: ENTREVISTA NUMERO NUEVE YA SE LE LEYO EL CONSENTIMIENTO INFORMADO A LA PARTICIPANTE Y SE DISCUTIO TAMBIEN CON ELLA Y ACCEDIO A PARTICIPAR DEL ESTUDIO COMENZAMOS.

ENTREVISTADOR:: ¿ESTAS AHORA MISMO BAJO TRATAMIENTO? ¿VERDAD?­

Paciente: si

ENTREVISTADOR:: ¿TIENES MEDICAMENTOS?

Paciente: ujum

ENTREVISTADOR:: ¿DESDE CUANDO, CUANDO EMPEZASTE A TOMARTE LOS MEDICAMENTOS PARA EL VIH PARA LA CONDICION?

Paciente: desde en enero de 2011

ENTREVISTADOR::¿AHÍ FUE CUANDO TE DIAGNOSTICARON?

Paciente: no yo hacen años que lo tenía lo que pasa es que a principio cuando pues me diagnosticaron si venía a citas y que se yo que, pero entonces deje de venir entonces pase todo ese tiempo y no había venido

ENTREVISTADOR:: AH OK ¿CUANDO TE DIAGNOSTICARON CON LA CONDICION?

Paciente: como en el 1991

ENTREVISTADOR:: AH DESDE EL 1991, Y ESTUVISTE DESDE EL 1991 HASTA EL 2011

Paciente: sin tomar el medicamento

ENTREVISTADOR:: NO TE LOS TOMABAS PORQUE EL MEDICO…

Paciente: no, no en si fíjate cuando yo empecé a venir aquí hace tiempo medicamento nunca me dieron solamente era hacerme exámenes de rutina así sacar sangre y nada mas

ENTREVISTADOR:: OK

Paciente:de tomar medicamentos nunca o sea que ahora es que yo estoy empezando a tomar medicamento

ENTREVISTADOR:: AH OK EN ALGUN DE ESOS… PORQUE FUE EN EL 1991 VEINTE AÑOS, EN ESOS VEINTIUNO (SE CUMPLE VEINTIUNAÑO AHORA ESOS VEINTIUN AÑO) EN ALGUN MOMENTO TE RECETARON LOS MEDICAMENTOS

Paciente: no

ENTREVISTADOR:: Y QUE TE DECIA EL MEDICAMENTO NO…

Paciente: como te digo al principio yo empecé a venir aquí eso fue como esto ha cambiado muchísimo aquí pues… entonces después yo deje de venir. Deje de venir y seguí normal.

ENTREVISTADOR::OK, NO VENIAS AL SEGUIMIENTO

Paciente:no venía aja no, deje de venir

ENTREVISTADOR:: ¿CUANTO TIEMPO ESTUVISTE SIN HACERTE LOS EXAMENES DE CARGA VIRAL Y TODO ESO?

Paciente: todo este tiempo nena

ENTREVISTADOR:: A LOS VEINTE AÑOS WOW

Paciente:y gracias a Dios, tu sabe’, nunca sentí nada fuera de lo normal, tu sabes me sentía bien

ENTREVISTADOR:: OK ¿Y QUE TE HIZO VENIR AHORA?

Paciente: nada pues yo dije pues tengo que chequearme a ver tantos años, verdad, no sabe que de momento me explotara algo, tu me entiendes y decidí ya este tiempo pues venir y volver y chequearme hacerme las pruebas de nuevo.

ENTREVISTADOR:: ¿Y SENTISTE QUE ESTABA EXPLOTANDO ALGO AUNQUE SEA PEQUEÑITO ALGUN SINTOMA?

Paciente: nada

ENTREVISTADOR::FUE QUE DE REPENTE…

Paciente: de repente lo pensé y lo decidí

ENTREVISTADOR:: O LO HABIAS PENSANDO ANTES PERO NO LO HABIAS HECHO…

Paciente: fue de momento que decidí, decidí quería saber en si como yo estaba

ENTREVISTADOR:: SI OK Y ¿COMO ESTABA CUANDO EL MEDICO TE HIZO LOS ANALISIS?

Paciente:fue fíjate mi carga estaba bien, tu sabes que ellos mismo se sorprendieron tantos años y tu carga viral está bien bajito o sea que casi ni ni…

ENTREVISTADOR:: NO TENIAS MUCHAS COPIAS DEL VIRUS PERO ¿AUN ASI TE RECETARON?

Paciente: aun así me recetaron, si

ENTREVISTADOR::¿Y LAS DEFENSAS COMO LAS TENIA?

Paciente: las defensas las tenía no tanto así bajita pero estaban este “deso”

ENTREVISTADOR:: AH OK ¿ESTABAN BAJITAS? ..¿TE ESTABAN EMPEZANDO A BAJAR LAS DEFENSAS?

Paciente aja

ENTREVISTADOR:: Y TODO ESE TIEMPO ¿TE ENFERMABAS A MENUDO?

Paciente: no nada nada ni catarro ni nah te digo que nada

ENTREVISTADOR:: WOW PUES QUE SUERTE

Paciente: porque pues después de la persona que estaba pues yo tuve una nena en ese de eso la persona que me dio esto como digo yo el falleció como al año y yo pues gracias a Dios yo seguí .Seguípara ‘lante seguí trabajando y pues normal

ENTREVISTADOR::¿Y TU NENA?

Paciente: ella si ella nació bien ella si pues yo la seguítrayendo aquí hasta los cinco años

ENTREVISTADOR:: AH OK

Paciente: tú sabes le hacían las pruebas y salía todo negativo hasta que le dieron de alta como dicen

ENTREVISTADOR:: AH OK Y EN ESOS CINCO AÑOS QUE TRAISTE A LA NENA ¿TU TE HACIAS TAMBIEN LAS PRUEBAS?

Paciente: si yo también las hacia

ENTREVISTADOR:: CUANDO LA NENA LE DIERON DE ALTA ¿TU DEJASTE DE VENIR PARA TI TAMBIEN?

Paciente: aja

ENTREVISTADOR:: SABES QUE ESTUVIESTE MAS O MENOS COMO UNOS DIESISEIS AÑOS SIN HACERTE LAS PRUEBAS, OK Y EMPEZASTE A TOMARTE LOS MEDICAMENTOS DESDE EL 2011 ¿CUANDO FUE LA FECHA?

Paciente: enenero

ENTREVISTADOR::YA HACE UN AÑO QUE TE ESTAS TOMANDO LOS MEDICAMENTOS

Paciente si ya hace un año

ENTREVISTADOR:: ¿ Y CUANDO TE DIERON LOS MEDICAMENTOS EN ESE MOMENTO DEL 2011, TENIAS LOS MEDICAMENTOS DISPONIBLES?

Paciente: si

ENTREVISTADOR::¿O TUVISTE QUE ESPERAR?

Paciente: no, no, no los tenía habían veces pues que habidas medicamento que no los había aquí y tenía que buscarlo en la farmacia

ENTREVISTADOR:: AH OK Y CUANDO LOS BUSCABAS EN LA FARMACIA ¿TENIAS QUE PAGAR ALGUN DEDUCIBLE?

Paciente: si

ENTREVISTADOR::¿CUANTO DEDUCIBLE TENIAS QUE PAGAR?

Paciente: Al principio eran este como un peso o algo así después subió

ENTREVISTADOR::¿TIENES EL PLAN… QUE PLAN?

Paciente: el de la reforma

ENTREVISTADOR:: ¿ Y QUE TAL EL PLAN DE LA REFORMA COMO EN TERMINOS DE LOS MEDICAMENTOS DE CUBRIR CUAL HA SIDO TU EXPERIENCIA CON ESO?

Paciente: bueno hasta ahora no he tenido problema tu sabes nunca me ha rechazado de que no me lo ha cubierto ni nada tu sabes

ENTREVISTADOR:: OK

Paciente: hasta ahora no he tenido problema

ENTREVISTADOR::ESTE Y VERDAD LA LO LAS EXPLICACIONES QUE TIENEN LOS MEDICAMENTOS SOBRE LOS EFECTOS SEGUNDARIOS

Paciente: si, si yo los leo

ENTREVISTADOR::¿Y ENTENDIENDES LO QUE DICEN?

Paciente si

ENTREVISTADOR:: ¿CUALES SON ALGUNOS DE LOS EFECTOS SEGUNDARIOS POR EJEMPLO ASI QUE TE ACUERDES?

Paciente: ay virgen, así... así... no me acuerdo bien yo sé que al principio cuando yo empecé a tomar el colesterol mío empezó a subir wuuff un montón entonces pues me lo cambiaron y entonces al cambiármelos me ha bajado bastante el colesterol.

ENTREVISTADOR:: OK

Paciente:tu sabes esos son algunos de los efectos que también dicen que de esos medicamentos

ENTREVISTADOR:: ¿Y CUALES MAS TE DIERON A TI ADEMAS QUE TE SUBIO EL COLESTEROL?

Paciente: fíjate eso fue lo uno que, que me “desto” porque todo lo demás salía bien tu sabes cuando hacían los análisis y eso

ENTREVISTADOR::OK ¿Y QUE PIENSAS DE LOS MEDICAMENTOS PARA TRATAR EL VIH?

Paciente:bueno por lo menos yo me los estoy tomando y gracias a Dios cuando me hago las pruebas salgo no detectable tu sabes qué pues yo pienso que sí que funcionan bien porque sino pues no me saliera

ENTREVISTADOR:: ¿Y LAS DEFENSAS COMO HAN ESTADO?

Paciente: pues fíjate las defensas me han subido bastante

ENTREVISTADOR:: ESTE ¿Y QUE TU ESPERAS DE ESTE TRATAMIENTO?

Paciente: que yo quisiera...que yo espero que esto uno no pudiera tenerlo por lo menos llegara el momento en que uno pues no tuviera que tomar nah tuviera libre verdad.

ENTREVISTADOR:: SI, CUANDO TE LAS TOMAS ¿QUE PIENSAS?

Paciente no fíjate no pienso nah yo me las tomo yo sé que al tomármelas pues me estoy ayudando. Ytu sabes que trato de no pensar negativo. Hay momentos que si uno a veces los problemas y que se yo que pero me tomo los medicamentos y tú sabes trato de no dejar de tomármelos tampoco pero.... tu sabe que en cuestión a cuestión a desto pues si me los sigo tomando y gracias a Dios hasta ahora siempre salgo no detectable

ENTREVISTADOR:: OK Y A VECES CUANDO, CUANDO ,ME DIJISTE HORITA QUE A VECES LOS PROBLEMAS VERDAD TRATAS DE PENSAR NEGATIVO. ¿TE HA PASADO QUE MOMENTOS CUANDO TIENES QUE TIENES ALGUN PROBLEMA COMO QUE TE LOS TOMAS COMO QUE SE MEZCLA TODO ESO?

Paciente: no, no, creo que no

ENTREVISTADOR:: ESTE POR EJEMPLO VERDAD CUANDO ESCUCHAS COMENTARIOS DE LAS PERSONAS QUE HAN DEJADO DE TOMARSE LOS MEDICAMENTOS CUANDO UNO ESTA ESPERANDO EN LA SALA A VECES UNOS ESCUCHA ESOS COMENTARIOS ¿QUE TU OPINAS DE ESO?

Paciente:yo puede que la persona que este dejando de tomárselo no sabe lo que está haciendo porque en si esto es para ayudar a uno; para pues poder vivir hasta que Dios permita que uno verdad quizás algunas personas “no me las voy a tomar pues no me va a pasar nah o voy a seguir bien” o que se yo verdad

ENTREVISTADOR::Y CUANDO ESCUCHAS COMENTARIOS DE LAS PERSONAS ¿QUE COSAS ESCUCHAS? EN CUANTO A PORQUE NO SE TOMAN LOS MEDICAMENTOS

Paciente: fíjate para decirte la verdad yo cuando vengo aquí yo casi siempre vengo cuando es médico casi casi no hay casi nadie sabe que en si comentarios así nunca he oído a nadie

ENTREVISTADOR:: ¿CONOCES A OTRAS PERSONAS QUE TIENEN LA CONDICION? ADEMAS DE TU...

Paciente no, no por lo menos afuera no

ENTREVISTADOR:: ESTE Y DURANTE ESTE A~O VERDAD QUE HAS ESTADO TOMANDOTE LOS MEDICAMENTOS EH VERDAD ALGUNAS PERSONAS LES PASA ESO... ¿HAS PENSANDO NO NECESARIAMENTE DEJAR DE TOMARTE LOS MEDICAMENTOS PERO, HAS PENSANDO EN DEJAR DE TOMARTE LOS MEDICAMENTOS ALGUNA DOSIS? POR EJEMPLO QUE TE PASE ASI POR LA MENTE

Pacient: no, no a veces se me ha olvidado tomármela no es que he dejado, no es que he dicho pues no me las voy a tomar más es que se me ha olvidado entonces cuando me acuerdo pues ya se me ha pasado pues me la tomo al otro dia

ENTREVISTADOR:: ¿TE PASA CON FRECUENCIA O...

Paciente: últimamente como me está pasando con más frecuencia

ENTREVISTADOR::SI, OK ¿TIENES ALGO O ALGUNA SITUACION ESTRÉS?

Paciente: bueno si yo tuve porque mi mama falleció hacen cuatro meses y ella estuvo bien enfermita y eso me puso a mi bajo un estrés... bien pues porque yo tenía que estar cuidándola y después ella como que perdió un poco la…

ENTREVISTADOR:: ¿QUE TENIA? ¿ ALZHEIMER?

Paciente: no, no, no, ella esta normal pero que de momento le dio eso así y pues eso me afectó muchísimo también tu sabe que ella pues como que la cogió conmigo y eso me puso…

ENTREVISTADOR:: ¿COMO LA COGIO CONTIGO?

Paciente: ella peleaba muchísimo conmigo me insultaba y todo eso me afectó mucho

ENTREVISTADOR:: Y ESO DE ALGUNA MANERA PUES EL ESTRÉS ¿SE TE OLVIDABAN LAS COSAS? ¿SE TE OLVIDABAN OTRAS COSAS TAMBIÉN? POR EJEMPLO ADEMAS DE LOS MEDICAMENTOS

Paciente: no

ENTREVISTADOR:: ESTE EH Y EN ALGUN MOMENTO VERDAD ¿HAS DEJADO DE TOMARTE VERDAD CONCIENTE MENTE? DECIR MIRA NO ME LOS VOY A TOMAR POR PAR DE DIAS

Paciente: no, como te digo pues si ha habido veces que se me ha olvidado pero que no es que he dejado o he dicho que no me los voy a tomar más.

ENTREVISTADOR:: OH OK ESTE EH CONSIDERAS VERDAD....... AHORA YO QUIERO QUE TU PIENSES EN TI SIN INCLUIR A OTRAS PERSONAS O CIRCUNSTANCIAS, EH, ¿CONSIDERAS QUE EN ALGUN SENTIMIENTO TUYO QUE TENGAS VERDAD ALGUNA EMOCION EMOCIONALMENTE COMO TE SIENTAS ESTE O ALGO VERDAD QUE TU CREAS, CREENCIAS QUE TU TENGAS O COSAS TUYAS PERSONALES TUYAS PUEDAN INFLUIR EN QUE EN ALGUN MOMENTO VERDAD PIENSES O DEJES DE TOMARTE LOS MEDICAMENTOS?… TU SABES COMO TU TE SIENTAS TU ESTADO DE ANIMO ESTE O COSAS QUE TU CREES EN TU MENTE ¿TU PIENSAS QUE ESO EN ALGUN MOMENTO INFLUYA EN QUE DEJES DE TOMARTE EL MEDICAMENTO O QUE PIENSES EN ALGUN MOMENTO EN DEJAR? … COMO TU DIJISTE HORITA LA MENTE NEGATIVA

Paciente: no

ENTREVISTADOR:: TODOS TENEMOS ESE LADO

Paciente: pues fíjate no vuelve y te digo así ha habido veces que se me han olvidado pero en cuestión a de que decida no tomármelas mas no, nunca se me ha venido eso a la mente

ENTREVISTADOR:: OK Y POR EJEMPLO EN ESOS VEINTIUN A~O QUE ESTUVISTE SIN VENIR A LA CLINICA ALGO DIGAMOS ¿ENTIENEDES QUE ALGO TUYO ... UN SENTIMIENTO O PENSANMIENTO QUE TE DECIA COMO QUE NO TE PERMITIA VENIR ACA A LA CLINICA? ¿QUE PASABA QUE TU NO VENIAS?

Paciente: no, era que pues como me sentía bien y para ese entonces yo estuve todo este tiempo sola después de eso no tuve más pareja ni nada tu sabes que yo he estado sola y yo dije pues como me sentía bien y no tenía pareja ni nada yo dije pues …. Tú sabes pues no lo veía que era necesario que siguiera viniendo.

ENTREVISTADOR:: ¿Y AHORAS TIENES PAREJA?

Paciente: no,no he tenido desde que “deso” me quede sola, me quede sola

ENTREVISTADOR:: AH OK

Paciente:tu sabes, que no tengo a nadie

ENTREVISTADOR:: Y ESA DECISION NO LO VISTE NECESARIO Y OBVIAMENTE AHORA LO ESTAS VIENDO NECESARIO, VERDAD, PORQUE ESTAS VINIENDO. ESE PROCESO DE TU DE VERLO QUE NO ERA NECESARIO HASTA VERLO QUE SI ES NECESARIO. CUENTAME UN POQUITO DE ESE PROCESO DE LLEGAR A UN PUNTO A OTRO. TENIAS UNA POSTURA DE QUE DECIAS QUE NO ERA NECESARIO Y AHORA DICES QUE SI O SEA ¿COMO FUE QUE PASO ESO?

Paciente: ok, pues vuelvo y te digo pasaron pues pasaron todos estos años y como no me daba ningún síntoma de nada.

ENTREVISTADOR:: SI

Paciente: nada de nada, de enfermarme no me daba catarro y no me daba nada. Yo sabiendo que era positiva algo tiene que haber verdad que yo no … a mí lo primero que se venía en la mente era que yo no tenía nada

ENTREVISTADOR:: OK PENSASTE QUE QUIZAS FUE UN ERROR O ALGO ASI

Paciente: exacto

ENTREVISTADOR:: OK ¿Y POR CUANTO TIEMPO ESTUVISTE PENSANDO ESO?

Paciente: todos estos años entonces como no vi… tu sabes… tampoco quería que me diera nada yo dije pues déjame ir y volver hacerme las pruebas que en verdad

ENTREVISTADOR:: ¿Y ESE MOMENTO FUE ESPONTANTEO QUE LLEGO O YA TU VENIAS PENSANDOLO?

Paciente: no, fue espontaneo

ENTREVISTADOR::Y TE ACUERDAS CUANDO FUE QUE TE LLEGO VERDAD TOMASTE ESA DECISION

Paciente: eso fue como para eso fue par de meses antes de enero algo así por ahí

ENTREVISTADOR:: ¿Y QUE ESTABA PASANDO EN ESE MOMENTO EN TU VIDA?

Paciente: conocí a una persona tu sabes yo conocí a esa persona entonces, que se yo; yo no encontraba la forma de cómo decirle a esa persona mi situación. Entonces, yo en ese momento pues hice un viaje a Estados Unidos que fui a ver a mi hermana y en ese tiempo que yo estuve allá pues este me ponía a pensar como yo encuentro la forma de decirle a esta persona pues lo que yo estoy que tengo hasta que pues me dio el ánimo y se lo dije

ENTREVISTADOR:: OK

Paciente: ve, entonces la pues la persona en ese momento lo acepto y que se yo y me dijo “¿tú te has chequeado?” y yo sí, o sea no volví mas a chequearme ni nada entonces ahí fue en ese momento que yo dije pues déjame ir a a chequearme a ver

ENTREVISTADOR:: ¿ Y LA PERSONA ESTE CONTINUO CONTIGO?

Paciente: estuvimos como un añoasí pero no, la relación no funciono

ENTREVISTADOR:: OK, O SEA QUE HACE POCO QUE

Paciente: hacen como dos años

ENTREVISTADOR::¿HACE DOS MESES, DOS A~OS, QUE ESTUVIERON JUNTOS?

Paciente: no, no como un año pero hacen dos años que ya la relación se terminó

ENTREVISTADOR:: ESTE EH TU FAMILIA ME DICE QUE TU MAMA MURIO ¿HACE POQUITO, VIVIA CONTIGO TU MAMA?

Paciente: o sean vivían a lado

ENTREVISTADOR:: EH TU MAMA SABIA QUE…

Paciente: ella sabía, siempre me lo machacaba como dice uno siempre me decía “ah que si tú no hubieses hecho esto que si esto”

ENTREVISTADOR:: ¿ TE ECHABA LA CULPA?

Paciente: aja

ENTREVISTADOR:: ESTE Y EM DE ALGUNA MANERA TU MAMA VERDAD ¿TU SIENTES QUE EL ECHARTE LA CULPA VERDAD O TENER ESA LA ACTITUD QUE TENIA EN CUANTO DE ALGUNA MANERA T E HACIA, ESOS VEINTIUNA~O, SENTIR COMO QUE NO QUE NO QUERIAS VENIR A HACERTE LOS EXAMENES Y A DARTE SEGUIMIENTO?

Paciente: o sea ella no como te digo este eso fue en estos últimos momentos que ella enfermo y eso que ella pues antes de eso....Si hubo par de veces que si me lo decía pero no años atrás como te digo o sea que fue ya cuando empezó a enfermar y eso

ENTREVISTADOR:: PARECE QUE ERA PARTE DE LA ENFERMEDAD

Paciente: puede ser, puede ser

ENTREVISTADOR:: Y EN ESE TIEMPO VERDAD ¿ALGUNAS AMISTADES QUE TU CREES QUE HAYAN INFLUIDO EN QUE EN AQUEL MOMENTO HAYAS DEJADO DE VENIR A LA CITA?

Paciente: no, nadie lo sabia

ENTREVISTADOR:: SOLAMENTE TU MAMA

Paciente:mi mama mi hermanas sabían de ahí en fuera ahora que mi mama le haya dicho a otras personas pues ahí no se

ENTREVISTADOR:: EH ¿Y AHORA QUIENES SABEN?

Paciente: mi hermana es la única que sabe y un hijo que yo tengo que él sabe pero la nena mía pues no nunca he… y nunca se lo diré

ENTREVISTADOR:: OK, ESTE ¿TU NENA NO NACIO CON LA CONDICION?

Paciente: ah no

ENTREVISTADOR:: OK, ESTE Y DE ALGUNA MANERA VERDAD SI ALGUNA EN AQUEL MOMENTO TUVISTE ALGUN TRATO CON EL PERSONAL QUE A LO MEJOR SE TE HIZO … NO TE GUSTO Y DECIDISTE NO VENIR PORQUE NO QUERIAS VER AL PERSONAL MEDICO O DE ENFERMERIA O ALGO ASI TU SABE…

Paciente: no, no nunca tuve cuando empecé pues no vine mucho así tampoco tu me entiendes cuando la nena mía “deso” a veces mi hermana era la que me la traía a las citas pero que yo nunca tuve problemas así con nadie

ENTREVISTADOR:: AH OK Y POR EJEMPLO ¿ALGUNA OTRA PERSONA QUE HAYA INFLUENCIADO EN QUE TU TE SINTIERAS ASI DE QUE NO...?

Paciente: no

ENTREVISTADOR:: ESTE YO QUIERO AHORA QUE TU PIENSES EN EL AMBIENTE DONDE TU DESENVUELVES, TE DESENVOLVISTESTE DESDE EL 91 HASTA AHORA, VERDAD, ESTE ¿DE QUE MANERA SI ALGUNA ESE AMBIENTE INFLUYO EN TU DECISION DE DEJAR DE VENIR A LAS CITAS O DEJAR DE DARTE SEGUIMIENTO?

Paciente: no, porque donde yo vivo es un sitio bueno y eso, tranquilo y cada cual está en su casa y nadie esta yo soy una persona que tampoco estoy en ..metías en casa de las personas tampoco yo pues siempre en mi casa o sea era del trabajo a mi casa y si acaso iba salía e iba en casa de mi tía que vive en Peñuelas

ENTREVISTADOR:: AJA

Paciente: pero en cuestión del ambiente y eso pues como te digo, es un sitio bueno tranquilo cada cual en su casa y nadie esta aunque yo siempre he sido una persona de dedede la casa como digo yo pero pues da la mala pata como dice uno que me toco esto a mí.

ENTREVISTADOR:: OK, OK ¿ESTE TU TRABAJAS AHORA?

Paciente: no estoy trabajando

ENTREVISTADOR:: ¿HASTA CUANDO TRABAJASTE?

Paciente: trabajé desde… hasta el 2009

ENTREVISTADOR:: OK, QUE TODAVIA NO TE ESTABAS TOMANDO LOS MEDICAMENTOS

Paciente: no

ENTREVISTADOR:: ESTUDIAS AHORA … ¿VAS A ALGUN CURSO?

Paciente: no, en mi casa era atendiendo un hermano que está impedido, que pues mi mama falleció pues

ENTREVISTADOR:: SI, ¿ LO ESTAS CUIDANDO TU ENTONCES?

Paciente si, entre mi hermana y yo lo cuidamos

ENTREVISTADOR:: ¿ VIVES CON TU HERMANA TAMBIEN EN LA CASA?

Paciente: no, no, ella, yo vivo arriba y mi hermana abajo, mi mama vivía al lao

ENTREVISTADOR:: AH QUE ESTAN CERQUITAS

Paciente: si

ENTREVISTADOR::¿ESA ES TU UNICA HERMANA?

Paciente: tengo otra hermana pero esa está enferma también ella tiene una condición la misma condición que tiene mi hermano que está impedido la tiene ella que no se si habrás, quizás hayas oído hablar de ella se llama Hungtiton

ENTREVISTADOR:: SI

Paciente: pues ellos tienen eso entonces mi hermana tenía otra hermana y murió de esa condición el año pasado

ENTREVISTADOR:: AJA ¿CORRE EN LA FAMILIA?

Paciente: si

ENTREVISTADOR:: ESTE EH POR EJEMPLO HAY PERSONAS QUE A VECES CUANDO ME HAN DICHO NO QUE CUANDO VAN AL LUGAR DE DONDE TIENEN SUS SERVICIOS MEDICOS LAS CONDICIONES EN QUE LO VEN COMO QUE LOS DESANIMAN A VENIR O INCLUSIVE A BUSCAR SUS MEDICAMENTOS A HACER SUS SEGUIMIENTOS SUS ANALISIS DE SEGUIMIENTO. ¿TE HA PASADO ESO?

Paciente: no

ENTREVISTADOR:: ESTE¿ALGUN LUGAR VERDAD QUE TE DE ALGUNA MANERA VERDAD TE HAYA DESANIMADO EN TERMINO DEL AMBIENTE EN DONDE TU TE DESENVUELVES O SEA QUE DE ALGUNA MANERA INDIRECTA TE HAYA DESANIMADO A NO VENIR A LAS CITAS MEDICAS?

Paciente: yo siempre las vengo, siempre vengo a las citas médicas

ENTREVISTADOR:: O SEA LO QUE QUIERO DECIR PERDONAME , ES DURANTE ESE TIEMPO QUE ESTUVISTE SIN DARTE EL SEGUIMIENTO LOS VEINTIUNANNNO QUE ESTUVISTE SIN DARTE EL SEGUIMIENTO

Paciente: que si hubo..

ENTREVISTADOR:: ¿ALGUN LUGAR? POR EJEMPLO ESTE HAY PERSONAS QUE DICEN PUES EL AMBIENTE DONDE YO SOCIALIZO A VECES COMO QUE NO ME QUIERO QUEDAR ALLI Y NO QUIERO ESO ME HACE NO IR A DARME SEGUIMIENTO

Paciente: no, porque como te digo yo este yo siempre he sido una persona de mi casa y eso

ENTREVISTADOR:: ESTE VERDAD... AHORA YO QUIERO QUE TU PIENSES EN PUERTO RICO EN COMO ESTAN LAS COSAS AHORA EN PUERTO RICO ESTE EH ¿DE QUE MANERA SI ALGUNA LAS COSAS QUE ESTAN PASANDO EN PUERTO RICO INFLUYEN EN LA DECISIÓN DE ALGUNAS PERSONAS PUEDE SER TU DECISION O DE ALGUNAS OTRAS PERSONAS DE TOMARSE LOS MEDICAMENTOS PARA EL VIH?

Paciente: por recursos me imagino que no porque por ejemplo yo lo digo por mi yo no he tenido problema en cuestión a obtener los medicamentos. Tu sabe’ que quizás a lo mejor la persona que pues decida no no seguir tomándoselas o algo pue’ a lo mejor se quiere morir o tiene muchísimos problemas que verdad

ENTREVISTADOR:: AJA

Paciente: pues eso pues también influye en los problemas este …familiares y todo eso

ENTREVISTADOR:: SI POR EJEMPLO ¿COMO PUEDE INFLUIR LOS PROBLEMAS FAMILIARES?

Paciente: por ejemplo si tu no tienes apoyo de tus familiares o tu… o te discriminan cosas así

ENTREVISTADOR::OK

Paciente: por lo cual lo hay todavía a pesar de … a principio cuando esta enfermedad este salió pues había mucho discrimen todavía pues a lo mejor dicen ay esa persona tiene eso pues no ..”Déjame echarme pa’ aca’ o no hablar con esa persona… muchas personas piensan así

ENTREVISTADOR::¿TE HA PASADO QUE HAS ESCUCHADO A LA GENTE?

Paciente: por lo menos yo no como te digo afuera no hay nadie más que sepa

ENTREVISTADOR:: POR ESO QUE EN TU CASO NO SE DA

Paciente: en mi caso no

ENTREVISTADOR:: Y PARTE DE TU DECISION DE NO DECIRLO,¿ES POR ESO? PORQUE TIENES MIEDO A QUE TE PUEDAN DISCRIMINAR

Paciente: si

ENTREVISTADOR:: OK, ¿ HAS VISTO QUE HAN DISCRIMINADO O ESCUCHAR A LAS PERSONAS HABLAR MAL DE LAS PERSONAS QUE TIENEN LA CONDICION DE VIH?

Paciente: no por lo menos aquí no

ENTREVISTADOR:: ESTE EH ¿TU CREES QUE DE ALGUNA MANERA PUDIERA INFLUIR EL GOBIERNO LAS DECISIONES QUE TOMA EL GOBIERNO EN CUANTO A LAS PERSONAS TIENEN EL ACCESO AL MEDICAMENTO LA DECISION DE TOMARSE EL MEDICAMENTO?

Paciente: el medicamento lo hay y como te digo el que no quiere tomárselo pues no no creo que sea por parte del gobierno

ENTREVISTADOR:: OK

Paciente: porque tu eres la que tiene la decisión de tomártelas o no tomártelas

ENTREVISTADOR:: AJA, ESTE Y LOS SERVICIOS DE SALUD, ¿QUE TU CREES DE LOS SERVICIOS DE SALUD? ¿TU CREES QUE DE ALGUNA MANERA EH EN LOS SERVICIOS DE SALUD HAY BARRERAS PARA QUE LA PERSONA DEJE TOMARSE LOS MEDICAMENTOS O IMPLICAN ALGUNA BARRERA?

Paciente: no creo, como te digo en el poco tiempo que llevo viniendo aquí si me han tratado muy bien y nunca he visto tu sabe hay algo que he visto o he escuchado algo negativo en contra mío tu sabe

ENTREVISTADOR:: SI OK

Paciente: que por esa parte tu sabe no

ENTREVISTADOR:: ESTE TRANSPORTACION ¿EN ALGUN MOMENTO HAS TENIDO PROBLEMA DE TRANSPORTACION?

Paciente: no, hasta ahora no

ENTREVISTADOR::TIENES TU...

Paciente: si yo tengo un carrito

ENTREVISTADOR:: EH ALGUNA SITUACION EN EL PAIS VERDAD QUE SEA SITUACION ECONOMICA O POR EJEMPLO LOS PLANES MEDICOS QUE ¿TU CREES QUE INFLUYAN O SEAN ALGUNA BARRERA PARA QUE LAS PERSONAS DEJEN DE TENER MEDICAMENTOS O DE TOMARSE LOS MEDICAMENTOS.?

Paciente: por lo menos este eh yo no como vuelvo y te digo yo este en el poquito tiempo que llevo pues este tratando esta “desto” no he tenido problema en cuestión a medicamentos no he tenido como se llama el cómo decirte este ay como decirte que no he tenido problemas en cuestión a eso.

ENTREVISTADOR:: AH OK. ESTE AHORA YO QUIERO QUE PIENSES EN LAS COSTUMBRES EN LAS CREENCIAS DE NUESTRA SOCIEDAD ¿DE QUE MANERA SI ALGUNA COSTUMBRES Y CREENCIAS DE NUESTRA SOCIEDAD INFLUYEN EN LA DECISION DE ALGUIEN DE DEJAR TOMAR LOS MEDICAMENTOS?

Paciente: como vuelvo y te digo tu eres la que decides si te las quiere tomar si quieres seguir el tratamiento o no los quieres seguir yo no creo que nadie influya sobre nadie lo digo por mi parte

ENTREVISTADOR:: POR TU EXPERIENCIA HORITA ME HABLASTE QUE A VECES LAS PERSONAS CUANDO SE SIENTEN DISCRIMINADAS VERDAD ESTE QUE A VECES CUANDO SIENTEN QUE LAS DISCRIMINAN PIENSAS ¿QUE ENTONCES ESO PODRIAS SER UNA RAZON COMO ME DIJO ALGUIEN UNA VEZ UNA EXCUSA PARA QUE ALGUIEN PIENSE O DEJE DE TOMARSE EL MEDICAMENTO?

Paciente: no, no creo, yo este al contrario pues ellos pues que piense lo que quieran pero yo voy a seguir mi tratamiento. Voy a seguir tomándome mis medicamentos.

ENTREVISTADOR:: OK, ESTE EH ¿Y EN LOS TIEMPOS QUE ESTAMOS VIVIENDO TU CREES QUE EN ESTOS TIEMPOS QUE ESTAMOS VIVIENDO, CON TODAS LAS COSAS QUE ESTAN PASANDO AHORA, TU CREES... DE QUE MANERA SI ALGUNA VERDAD ESTOS TIEMPOS INFLUYEN EN LA DECISION DE ALGUIEN, PORQUE UNO TOMA LA DECISION COMO TU DICE, PERO HAY COSAS QUE PUEDEN INFLUIR... ESTOS TIEMPOS DE ALGUNA MANERA PIENSAS QUE INFLUYEN EN LA DECISION DE ALGUIEN TOMARSE MEDICAMENTOS POR LAS COSAS QUE ESTAN PASANDO.

Paciente: ay es que no se como contestarte esa pregunta porque vuelvo y te digo este yo... a mi nadie va influir sobre mi... yo como te digo vuelvo... yo tomo mi decisión si me las tomo, si vengo al tratamiento o no. Lo digo por mi cada cual pues tiene su forma de pensar.

ENTREVISTADOR: : SI, OK. ENTONCES Y VOLVIENDO A LA EXPERIENCIA EN QUE TU TOMASTE LA DECISION DE NO VENIR AL SEGUIMIENTO. ALGO QUE ME ESTA BIEN CURIOSO PORQUE ME HA PASADO CON OTRAS PERSONAS CON QUIEN HE HABLADO ESTE QUE CUANDO SI EN EL MOMENTO NO SIENTE QUE TIENEN LOS SINTOMAS PUES A LO MEJOR OTRAS PERSONAS NO SE TOMAN EL MEDICAMENTO O NO VIENEN EH.... ESTE.... DE ALGUNA MANERA EH VERDAD EL, EL.... Y ES QUE ME DA CURIOSIDAD SABER SI, SI, PASABA TIEMPO QUE A LO MEJOR TU NI SIQUIERA PENSABA QUE LA TENIAS O SI TODOS LOS DIAS LO PENSABAS PERO COMO NO TENIAS LOS SINTOMAS ... ME DA CURIOSIDAD TU EXPERIENCIA

Paciente: no, no lo pensaba

ENTREVISTADOR:: ERA COMPLETAMENTE COMO QUE SE...

Paciente: exacto

ENTREVISTADOR:: AH OK QUE INTERESANTE

Paciente: si

ENTREVISTADOR:: Y POR TODOS LOS VEINTIUN A~OS O EN OCASIONES ASI LO PENSABAS

Paciente: si lo pensaba yo decía... “ ¿porque a mí?”-decía yo. Pero que no tu sabe’ era de momento y seguía normal.

ENTREVISTADOR:: Y EN ESOS MOMENTOS EN QUE TE DABA ESO ¿COMO TE SENTIAS? CUANDO LO PENSABAS O TE VENIA A LA MENTE.

Paciente: me sentía mal, me sentía mal yo decia“ ¿Por qué? ¿Por qué a mi, verdad?”

ENTREVISTADOR: : ¿AHORA QUE TE ESTAS TOMANDO LOS MEDICAMENTOS PIENSAS EN ESO MAS FRECUENTEMENTE QUE ANTES?

Paciente : Porque ya como te digo lo he aceptado, hace tiempo pues no pensaba en eso ni tampoco quería aceptarlo

ENTREVISTADOR:: AHORA SI, ESTAS EN ESE PROCESO AHORA, QUE ES UN PROCESO IMPORTANTE, ¿TU ESTAS RECIBIENDO SERVICIOS PSICOLOGICOS ACA?

Paciente: SI yo a veces hablo con ....

ENTREVISTADOR:: Si aja, que ella es una de las manejadoras

Paciente: si

ENTREVISTADOR:: BUENO ME DIJISTE QUE EN EL MOMENTO CUANDO TU DECIDISTE VOLVER A LA CLINICA ERA QUE ESTABAS COMENZANDO UNA RELACION, VERDAD Y ESO TE MOTIVO ¿CREES QUE SI NO HUBIESE ESTADO ESA SITUACION VERDAD DE QUE ESTUVIERAS COMENZANDO LA SITUACION LA RELACION TODAVIA ESTUVIERAS ASI?

Paciente: si

ENTREVISTADOR:: OK ADEMAS DE ESO VERDAD CONCRETAMENTE QUE ESTABAS COMENZANDO LA RELACION ¿ALGO MAS QUE SE A~ADIO A ESO QUE TE HIZO TOMAR LAS FUERZAS PARA VENIR?

Paciente: no, eso fue lo que me motivo a venir

ENTREVISTADOR:: ENTONCES CAMBIANDO CUANDO SE TE OLVIDA TOMARTE ALGUNA DOSIS DEL MEDICAMENTO QUE TE AYUDA A QUE ESO NO PASE... ¿USAS ALGUNA ESTRATEGIA? PARA QUE VERDAD

Paciente: lo que hago pues es que porque yo normalmente me las tomo por las mañanas, desayuno y me las tomo pero que hay de momento que me las tomo más tarde y más tarde es que se me olvida completamente se me olvida.

ENTREVISTADOR:: ¿CUANDO FUE LA ULTIMA VEZ QUE SE TE OLVIDO?

Paciente: antier

ENTREVISTADOR:: UNA VEZ AL DIA, ¿ESTAS BIEN?

Paciente: si estoy bn

ENTREVISTADOR:: ESTE Y ENTONCES ¿HAS PENSADO EN ALGO VERDAD ALGUNA ESTRATEGIA PARA AYUDARTE A QUE NO SE TE OLVIDE

Paciente: sí que en cuanto desayune me las tome, por la mañana para que no se me olvido

ENTREVISTADOR:: Y POR EJEMPLO SI TU AHORA MISMO TE ENCONTRARAS UNA PERSONA QUE ESTE PENSANDO EN DEJAR DE TOMARTE LOS MEDICAMENTOS ¿Qué LE DIRIAS?

Paciente: que no lo haga que esto es para tu salud, esto es para ayudarte. Para que tu puedas este vivir muchos años mas.

ENTREVISTADOR: : ¿ALGO MAS QUE LE RECOMENDARIAS A UNA PERSONA PARA QUE NO DEJE DE TOMAR..

Paciente: que pues le diría también que, que trate de no pensar en cosas negativas porque a veces tu te pones a que se yo... te vienen cosas negativas a la mente tu sabe’ “pues no me voy a tomar nah”

ENTREVISTADOR:: SI

Paciente: que trate pues de no pensar nada negativo

ENTREVISTADOR:: OK ¿DE QUE MANERA, QUE COSAS PODIA HACER PARA NO PENSAR EN ALGO NEGATIVO Y QUE NO LE VENGA LAS COSAS NEGATIVAS? ALGO QUE QUIZAS TE HAYA AYUDADO A TI O A OTRA PERSONA

Paciente: Por lo menos a mi pues yo como me han venido así cosas negativas a la mente, pues yo trato de que no me afecten y digo “no, no voy a dejar que esto me afecte” yo lo digo yo, trato de que no me afecte o sea trato de vencerlo como digo yo. Es como la persona adicta verdad si la persona dice... es como cuando tu fuma. Yo fumaba antes, yo fume muchísimos años y llego el momento en que yo dije aquí ya no voy a fumar más y tome esa decisión y no volví y no fume más. Y llevo todos estos años sin fumar.

ENTREVISTADOR:: FUISTE VALIENTE

Paciente: Si yo dije no, y me daba el deseo de fumar y yo decía no

ENTREVISTADOR:: Y ESO DA FUERTE CUANDO UNO ESTA DEJANDO

Paciente: que sí que nena yo digo no tengo que hacerlo y tengo que hacerlo; y lo logre.

ENTREVISTADOR:: QUE BUENO

Paciente: o sea es fuerza de voluntad que tú tienes que tener es como el adicto si tú no tienes esa fuerza de voluntad de tu salir de ese vicio nadie lo va a hacer.

ENTREVISTADOR:: Y DESDE LA MISMA MANERA DEBE TENER FUERZA DE VOLUNTAD PARA MANTENERSE ENTONCES EN EL... TOMANDOSE EL MEDICAMENTO, SIGUIENDO EL TRATAMIENTO.

Paciente: exacto

ENTREVISTADOR:: CUANDO TU ESCUCHAS LA FRASE ADHERENCIA O CUMPLIMIENTOS DE LAS RECOMENDACIONES MEDICAS ¿CON QUE LA RELACINAS?

Paciente : cumplimiento, adherencia..no entiendo esa palabra

ENTREVISTADOR:: CUMPLIMIENTO

Paciente: ah cumplimiento, pues si te da algunas instrucciones pues tu seguir las instrucciones que el medico te diga. Si te dice pues tomate este medicamento tres veces al día pues se supone que te las tomes tres veces al día.

ENTREVISTADOR:: OK Y ADEMAS DE TOMARTE EL MEDICAMENTO PARA EL VIH ¿QUE OTRAS COSAS TE RECOMIENDAN TU MEDICO O TUS MEDICOS? QUE TE VEN ACA

Paciente: este nada ellos no me dicen nada a mi aquí, na’ que no deje de tomarme los medicamentos.

ENTREVISTADOR: : OK ¿TE MANDAN A HACER EXAMEN MEDICO?

Paciente: si

ENTREVISTADOR:: VERDAD, ¿MAMOGRAFIAS POR EJEMPLO? ELLOS TE MANDAN, TE DAN ESOS REFERIDOS

Paciente: no, aquí no han dao’ mamografía, aquí nunca han dao’ ellos este gine... este aquí te hacen la prueba de la... de ...de ... el PAP

ENTREVISTADOR:: ¿TE LAS HACES AQUÍ, O TIENES QUE IR AL GINECOLOGO?

Paciente: aqui

ENTREVISTADOR:: ¿CUANDO FUE LA ULTIMA VEZ QUE TE LA HICISTE?

Paciente: antes de navidades

ENTREVISTADOR:: ANTES DE NAVIDADES ¿O SEA EL A~O PASADO?

Paciente: Si

ENTREVISTADOR:: Y ESTE ¿MAMOGRAFIAS, TE HAS HECHO ALGUNA?

Paciente: si pero ya hacen como dos años atrás, como dos

ENTREVISTADOR:: OK ESO TE LA HIZO TU GINECOLOGO

Paciente: aja

ENTREVISTADOR:: ¿TODAVIA VAS AL GINECOLOGO?

Paciente : no porque la ginecóloga que yo tenía pues como era privada y ella no coge la reforma pues... y como aquí me hicieron la prueba del ...

ENTREVISTADOR:: PAP OK ESTE LA MAMOGRAFIA NO TE HAN DADO REFERIDO PARA ESO TU NECESITAS REFERIDO ¿VERDAD?

Paciente: Si porque aquí no la hacen

ENTREVISTADOR:: CUANDO LAS CITAS MEDICAS POR EJEMPLO ¿SE TE HA HECHO DIFICIL VENIR ALGUNA O HAS TENIDO QUE FALTAR EN ESTE A~O?

Paciente: ¿para venir aquí?

ENTREVISTADOR:: SI

Paciente: no

ENTREVISTADOR:: Y POR EJEMPLO LOS EXAMENES DE SANGRE...

Paciente: también siempre vengo

ENTREVISTADOR:: O SEA QUE EN ESTE A~O PUES HAS CUMPLIDO CON LAS CITAS LOS EXAMENES LO UNICO QUE TE FALTA SERIA LA MAMOGRAFIA... ¿ALGUNA OTRA CONDICION MEDICA QUE TU TIENES?

Paciente: este tengo diabetis

ENTREVISTADOR:: OK ¿Y COMO ESTAS ALLI?

Paciente: pues yo no se

ENTREVISTADOR:: ¿TE LA ESTAS TRATANDO?

Paciente: porque al principio si tomaba pastillas y eso pero que las pastillas me daban efecto... me daba rápido para ir al baño ...

ENTREVISTADOR:: AH OK

Paciente: y deje de tomármelas y pues y no así así no he vuelto a tratarme na’ más que esto aquí nada más.

ENTREVISTADOR:: SI, OK

Paciente: pero me he chequeao’ con una maquinita que hay en casa y me ha salido bastante bien pa’ como yo la tenía que subía a casi trescientas y pico.

ENTREVISTADOR:: ¿Cuándo FUE LA ULTIMA VEZ QUE TE CHEQUEASTE LA AZUCAR CON TU MAQUINITA?

Paciente: hace como... yo creo que hace como una semana.

ENTREVISTADOR:: SI

Paciente: La tenía en 135

ENTREVISTADOR:: ¿TIENES DIABETES TIPO DOS? ¿Cuánto HACE QUE TE DIAGNOSTICARON CON DIABETES?

Paciente: Cuando yo salí embarazada de mi nena, que salí con diabetes estacionaria. Entonces después no me trate, entonces después me hacia las pruebas y eso y salí que este

ENTREVISTADOR:: MM OK Y POR EJEMPLO EH PRUEBAS PARA EL CARDIALOGO, PARA VER COMO ESTA EL CORAZON, ¿TE HAN DADO REFERIDO PARA ESO?.

Paciente: no

ENTREVISTADOR:: ¿HAY ALGO MAS EN TERMINOS DE LAS CONDICIONES VERDAD DE DIABETES, RECOMENDACIONES QUE A LO MEJOR NO TE HAYA PREGUNTADO QUE QUIERAS A~ADIR DE ESO?

Paciente: no

ENTREVISTADOR:: ¿Y ALGO MAS QUE QUIERAS A~ADIR DE TODO LO QUE HEMOS ESTADO HABLANDO VERDAD QUE NO TE HE PREGUNTADO QUE QUISIERAS A~ADIR?

Paciente: no, no tengo así nada más que añadir.

ENTREVISTADOR:: ENTONCES CULMINAMOS EL PROCESO.

Entrevista número nueve

ENTREVISTADOR: ENTREVISTA NUMERO NUEVE YA SE LE LEYO EL CONSENTIMIENTO INFORMADO A LA PARTICIPANTE Y SE DISCUTIO TAMBIEN CON ELLA Y ACCEDIO A PARTICIPAR DEL ESTUDIO COMENZAMOS.

ENTREVISTADOR:: ¿ESTAS AHORA MISMO BAJO TRATAMIENTO? ¿VERDAD?­

Paciente: si

ENTREVISTADOR:: ¿TIENES MEDICAMENTOS?

Paciente: ujum

ENTREVISTADOR:: ¿DESDE CUANDO, CUANDO EMPEZASTE A TOMARTE LOS MEDICAMENTOS PARA EL VIH PARA LA CONDICION?

Paciente: desde en enero de 2011

ENTREVISTADOR::¿AHÍ FUE CUANDO TE DIAGNOSTICARON?

Paciente: no yo hacen años que lo tenía lo que pasa es que a principio cuando pues me diagnosticaron si venía a citas y que se yo que, pero entonces deje de venir entonces pase todo ese tiempo y no había venido

ENTREVISTADOR:: AH OK ¿CUANDO TE DIAGNOSTICARON CON LA CONDICION?

Paciente: como en el 1991

ENTREVISTADOR:: AH DESDE EL 1991, Y ESTUVISTE DESDE EL 1991 HASTA EL 2011

Paciente: sin tomar el medicamento

ENTREVISTADOR:: NO TE LOS TOMABAS PORQUE EL MEDICO…

Paciente: no, no en si fíjate cuando yo empecé a venir aquí hace tiempo medicamento nunca me dieron solamente era hacerme exámenes de rutina así sacar sangre y nada mas

ENTREVISTADOR:: OK

Paciente:de tomar medicamentos nunca o sea que ahora es que yo estoy empezando a tomar medicamento

ENTREVISTADOR:: AH OK EN ALGUN DE ESOS… PORQUE FUE EN EL 1991 VEINTE AÑOS, EN ESOS VEINTIUNO (SE CUMPLE VEINTIUNAÑO AHORA ESOS VEINTIUN AÑO) EN ALGUN MOMENTO TE RECETARON LOS MEDICAMENTOS

Paciente: no

ENTREVISTADOR:: Y QUE TE DECIA EL MEDICAMENTO NO…

Paciente: como te digo al principio yo empecé a venir aquí eso fue como esto ha cambiado muchísimo aquí pues… entonces después yo deje de venir. Deje de venir y seguí normal.

ENTREVISTADOR::OK, NO VENIAS AL SEGUIMIENTO

Paciente:no venía aja no, deje de venir

ENTREVISTADOR:: ¿CUANTO TIEMPO ESTUVISTE SIN HACERTE LOS EXAMENES DE CARGA VIRAL Y TODO ESO?

Paciente: todo este tiempo nena

ENTREVISTADOR:: A LOS VEINTE AÑOS WOW

Paciente:y gracias a Dios, tu sabe’, nunca sentí nada fuera de lo normal, tu sabes me sentía bien

ENTREVISTADOR:: OK ¿Y QUE TE HIZO VENIR AHORA?

Paciente: nada pues yo dije pues tengo que chequearme a ver tantos años, verdad, no sabe que de momento me explotara algo, tu me entiendes y decidí ya este tiempo pues venir y volver y chequearme hacerme las pruebas de nuevo.

ENTREVISTADOR:: ¿Y SENTISTE QUE ESTABA EXPLOTANDO ALGO AUNQUE SEA PEQUEÑITO ALGUN SINTOMA?

Paciente: nada

ENTREVISTADOR::FUE QUE DE REPENTE…

Paciente: de repente lo pensé y lo decidí

ENTREVISTADOR:: O LO HABIAS PENSANDO ANTES PERO NO LO HABIAS HECHO…

Paciente: fue de momento que decidí, decidí quería saber en si como yo estaba

ENTREVISTADOR:: SI OK Y ¿COMO ESTABA CUANDO EL MEDICO TE HIZO LOS ANALISIS?

Paciente:fue fíjate mi carga estaba bien, tu sabes que ellos mismo se sorprendieron tantos años y tu carga viral está bien bajito o sea que casi ni ni…

ENTREVISTADOR:: NO TENIAS MUCHAS COPIAS DEL VIRUS PERO ¿AUN ASI TE RECETARON?

Paciente: aun así me recetaron, si

ENTREVISTADOR::¿Y LAS DEFENSAS COMO LAS TENIA?

Paciente: las defensas las tenía no tanto así bajita pero estaban este “deso”

ENTREVISTADOR:: AH OK ¿ESTABAN BAJITAS? ..¿TE ESTABAN EMPEZANDO A BAJAR LAS DEFENSAS?

Paciente aja

ENTREVISTADOR:: Y TODO ESE TIEMPO ¿TE ENFERMABAS A MENUDO?

Paciente: no nada nada ni catarro ni nah te digo que nada

ENTREVISTADOR:: WOW PUES QUE SUERTE

Paciente: porque pues después de la persona que estaba pues yo tuve una nena en ese de eso la persona que me dio esto como digo yo el falleció como al año y yo pues gracias a Dios yo seguí .Seguípara ‘lante seguí trabajando y pues normal

ENTREVISTADOR::¿Y TU NENA?

Paciente: ella si ella nació bien ella si pues yo la seguítrayendo aquí hasta los cinco años

ENTREVISTADOR:: AH OK

Paciente: tú sabes le hacían las pruebas y salía todo negativo hasta que le dieron de alta como dicen

ENTREVISTADOR:: AH OK Y EN ESOS CINCO AÑOS QUE TRAISTE A LA NENA ¿TU TE HACIAS TAMBIEN LAS PRUEBAS?

Paciente: si yo también las hacia

ENTREVISTADOR:: CUANDO LA NENA LE DIERON DE ALTA ¿TU DEJASTE DE VENIR PARA TI TAMBIEN?

Paciente: aja

ENTREVISTADOR:: SABES QUE ESTUVIESTE MAS O MENOS COMO UNOS DIESISEIS AÑOS SIN HACERTE LAS PRUEBAS, OK Y EMPEZASTE A TOMARTE LOS MEDICAMENTOS DESDE EL 2011 ¿CUANDO FUE LA FECHA?

Paciente: enenero

ENTREVISTADOR::YA HACE UN AÑO QUE TE ESTAS TOMANDO LOS MEDICAMENTOS

Paciente si ya hace un año

ENTREVISTADOR:: ¿ Y CUANDO TE DIERON LOS MEDICAMENTOS EN ESE MOMENTO DEL 2011, TENIAS LOS MEDICAMENTOS DISPONIBLES?

Paciente: si

ENTREVISTADOR::¿O TUVISTE QUE ESPERAR?

Paciente: no, no, no los tenía habían veces pues que habidas medicamento que no los había aquí y tenía que buscarlo en la farmacia

ENTREVISTADOR:: AH OK Y CUANDO LOS BUSCABAS EN LA FARMACIA ¿TENIAS QUE PAGAR ALGUN DEDUCIBLE?

Paciente: si

ENTREVISTADOR::¿CUANTO DEDUCIBLE TENIAS QUE PAGAR?

Paciente: Al principio eran este como un peso o algo así después subió

ENTREVISTADOR::¿TIENES EL PLAN… QUE PLAN?

Paciente: el de la reforma

ENTREVISTADOR:: ¿ Y QUE TAL EL PLAN DE LA REFORMA COMO EN TERMINOS DE LOS MEDICAMENTOS DE CUBRIR CUAL HA SIDO TU EXPERIENCIA CON ESO?

Paciente: bueno hasta ahora no he tenido problema tu sabes nunca me ha rechazado de que no me lo ha cubierto ni nada tu sabes

ENTREVISTADOR:: OK

Paciente: hasta ahora no he tenido problema

ENTREVISTADOR::ESTE Y VERDAD LA LO LAS EXPLICACIONES QUE TIENEN LOS MEDICAMENTOS SOBRE LOS EFECTOS SEGUNDARIOS

Paciente: si, si yo los leo

ENTREVISTADOR::¿Y ENTENDIENDES LO QUE DICEN?

Paciente si

ENTREVISTADOR:: ¿CUALES SON ALGUNOS DE LOS EFECTOS SEGUNDARIOS POR EJEMPLO ASI QUE TE ACUERDES?

Paciente: ay virgen, así... así... no me acuerdo bien yo sé que al principio cuando yo empecé a tomar el colesterol mío empezó a subir wuuff un montón entonces pues me lo cambiaron y entonces al cambiármelos me ha bajado bastante el colesterol.

ENTREVISTADOR:: OK

Paciente:tu sabes esos son algunos de los efectos que también dicen que de esos medicamentos

ENTREVISTADOR:: ¿Y CUALES MAS TE DIERON A TI ADEMAS QUE TE SUBIO EL COLESTEROL?

Paciente: fíjate eso fue lo uno que, que me “desto” porque todo lo demás salía bien tu sabes cuando hacían los análisis y eso

ENTREVISTADOR::OK ¿Y QUE PIENSAS DE LOS MEDICAMENTOS PARA TRATAR EL VIH?

Paciente:bueno por lo menos yo me los estoy tomando y gracias a Dios cuando me hago las pruebas salgo no detectable tu sabes qué pues yo pienso que sí que funcionan bien porque sino pues no me saliera

ENTREVISTADOR:: ¿Y LAS DEFENSAS COMO HAN ESTADO?

Paciente: pues fíjate las defensas me han subido bastante

ENTREVISTADOR:: ESTE ¿Y QUE TU ESPERAS DE ESTE TRATAMIENTO?

Paciente: que yo quisiera...que yo espero que esto uno no pudiera tenerlo por lo menos llegara el momento en que uno pues no tuviera que tomar nah tuviera libre verdad.

ENTREVISTADOR:: SI, CUANDO TE LAS TOMAS ¿QUE PIENSAS?

Paciente no fíjate no pienso nah yo me las tomo yo sé que al tomármelas pues me estoy ayudando. Ytu sabes que trato de no pensar negativo. Hay momentos que si uno a veces los problemas y que se yo que pero me tomo los medicamentos y tú sabes trato de no dejar de tomármelos tampoco pero.... tu sabe que en cuestión a cuestión a desto pues si me los sigo tomando y gracias a Dios hasta ahora siempre salgo no detectable

ENTREVISTADOR:: OK Y A VECES CUANDO, CUANDO ,ME DIJISTE HORITA QUE A VECES LOS PROBLEMAS VERDAD TRATAS DE PENSAR NEGATIVO. ¿TE HA PASADO QUE MOMENTOS CUANDO TIENES QUE TIENES ALGUN PROBLEMA COMO QUE TE LOS TOMAS COMO QUE SE MEZCLA TODO ESO?

Paciente: no, no, creo que no

ENTREVISTADOR:: ESTE POR EJEMPLO VERDAD CUANDO ESCUCHAS COMENTARIOS DE LAS PERSONAS QUE HAN DEJADO DE TOMARSE LOS MEDICAMENTOS CUANDO UNO ESTA ESPERANDO EN LA SALA A VECES UNOS ESCUCHA ESOS COMENTARIOS ¿QUE TU OPINAS DE ESO?

Paciente:yo puede que la persona que este dejando de tomárselo no sabe lo que está haciendo porque en si esto es para ayudar a uno; para pues poder vivir hasta que Dios permita que uno verdad quizás algunas personas “no me las voy a tomar pues no me va a pasar nah o voy a seguir bien” o que se yo verdad

ENTREVISTADOR::Y CUANDO ESCUCHAS COMENTARIOS DE LAS PERSONAS ¿QUE COSAS ESCUCHAS? EN CUANTO A PORQUE NO SE TOMAN LOS MEDICAMENTOS

Paciente: fíjate para decirte la verdad yo cuando vengo aquí yo casi siempre vengo cuando es médico casi casi no hay casi nadie sabe que en si comentarios así nunca he oído a nadie

ENTREVISTADOR:: ¿CONOCES A OTRAS PERSONAS QUE TIENEN LA CONDICION? ADEMAS DE TU...

Paciente no, no por lo menos afuera no

ENTREVISTADOR:: ESTE Y DURANTE ESTE A~O VERDAD QUE HAS ESTADO TOMANDOTE LOS MEDICAMENTOS EH VERDAD ALGUNAS PERSONAS LES PASA ESO... ¿HAS PENSANDO NO NECESARIAMENTE DEJAR DE TOMARTE LOS MEDICAMENTOS PERO, HAS PENSANDO EN DEJAR DE TOMARTE LOS MEDICAMENTOS ALGUNA DOSIS? POR EJEMPLO QUE TE PASE ASI POR LA MENTE

Pacient: no, no a veces se me ha olvidado tomármela no es que he dejado, no es que he dicho pues no me las voy a tomar más es que se me ha olvidado entonces cuando me acuerdo pues ya se me ha pasado pues me la tomo al otro dia

ENTREVISTADOR:: ¿TE PASA CON FRECUENCIA O...

Paciente: últimamente como me está pasando con más frecuencia

ENTREVISTADOR::SI, OK ¿TIENES ALGO O ALGUNA SITUACION ESTRÉS?

Paciente: bueno si yo tuve porque mi mama falleció hacen cuatro meses y ella estuvo bien enfermita y eso me puso a mi bajo un estrés... bien pues porque yo tenía que estar cuidándola y después ella como que perdió un poco la…

ENTREVISTADOR:: ¿QUE TENIA? ¿ ALZHEIMER?

Paciente: no, no, no, ella esta normal pero que de momento le dio eso así y pues eso me afectó muchísimo también tu sabe que ella pues como que la cogió conmigo y eso me puso…

ENTREVISTADOR:: ¿COMO LA COGIO CONTIGO?

Paciente: ella peleaba muchísimo conmigo me insultaba y todo eso me afectó mucho

ENTREVISTADOR:: Y ESO DE ALGUNA MANERA PUES EL ESTRÉS ¿SE TE OLVIDABAN LAS COSAS? ¿SE TE OLVIDABAN OTRAS COSAS TAMBIÉN? POR EJEMPLO ADEMAS DE LOS MEDICAMENTOS

Paciente: no

ENTREVISTADOR:: ESTE EH Y EN ALGUN MOMENTO VERDAD ¿HAS DEJADO DE TOMARTE VERDAD CONCIENTE MENTE? DECIR MIRA NO ME LOS VOY A TOMAR POR PAR DE DIAS

Paciente: no, como te digo pues si ha habido veces que se me ha olvidado pero que no es que he dejado o he dicho que no me los voy a tomar más.

ENTREVISTADOR:: OH OK ESTE EH CONSIDERAS VERDAD....... AHORA YO QUIERO QUE TU PIENSES EN TI SIN INCLUIR A OTRAS PERSONAS O CIRCUNSTANCIAS, EH, ¿CONSIDERAS QUE EN ALGUN SENTIMIENTO TUYO QUE TENGAS VERDAD ALGUNA EMOCION EMOCIONALMENTE COMO TE SIENTAS ESTE O ALGO VERDAD QUE TU CREAS, CREENCIAS QUE TU TENGAS O COSAS TUYAS PERSONALES TUYAS PUEDAN INFLUIR EN QUE EN ALGUN MOMENTO VERDAD PIENSES O DEJES DE TOMARTE LOS MEDICAMENTOS?… TU SABES COMO TU TE SIENTAS TU ESTADO DE ANIMO ESTE O COSAS QUE TU CREES EN TU MENTE ¿TU PIENSAS QUE ESO EN ALGUN MOMENTO INFLUYA EN QUE DEJES DE TOMARTE EL MEDICAMENTO O QUE PIENSES EN ALGUN MOMENTO EN DEJAR? … COMO TU DIJISTE HORITA LA MENTE NEGATIVA

Paciente: no

ENTREVISTADOR:: TODOS TENEMOS ESE LADO

Paciente: pues fíjate no vuelve y te digo así ha habido veces que se me han olvidado pero en cuestión a de que decida no tomármelas mas no, nunca se me ha venido eso a la mente

ENTREVISTADOR:: OK Y POR EJEMPLO EN ESOS VEINTIUN A~O QUE ESTUVISTE SIN VENIR A LA CLINICA ALGO DIGAMOS ¿ENTIENEDES QUE ALGO TUYO ... UN SENTIMIENTO O PENSANMIENTO QUE TE DECIA COMO QUE NO TE PERMITIA VENIR ACA A LA CLINICA? ¿QUE PASABA QUE TU NO VENIAS?

Paciente: no, era que pues como me sentía bien y para ese entonces yo estuve todo este tiempo sola después de eso no tuve más pareja ni nada tu sabes que yo he estado sola y yo dije pues como me sentía bien y no tenía pareja ni nada yo dije pues …. Tú sabes pues no lo veía que era necesario que siguiera viniendo.

ENTREVISTADOR:: ¿Y AHORAS TIENES PAREJA?

Paciente: no,no he tenido desde que “deso” me quede sola, me quede sola

ENTREVISTADOR:: AH OK

Paciente:tu sabes, que no tengo a nadie

ENTREVISTADOR:: Y ESA DECISION NO LO VISTE NECESARIO Y OBVIAMENTE AHORA LO ESTAS VIENDO NECESARIO, VERDAD, PORQUE ESTAS VINIENDO. ESE PROCESO DE TU DE VERLO QUE NO ERA NECESARIO HASTA VERLO QUE SI ES NECESARIO. CUENTAME UN POQUITO DE ESE PROCESO DE LLEGAR A UN PUNTO A OTRO. TENIAS UNA POSTURA DE QUE DECIAS QUE NO ERA NECESARIO Y AHORA DICES QUE SI O SEA ¿COMO FUE QUE PASO ESO?

Paciente: ok, pues vuelvo y te digo pasaron pues pasaron todos estos años y como no me daba ningún síntoma de nada.

ENTREVISTADOR:: SI

Paciente: nada de nada, de enfermarme no me daba catarro y no me daba nada. Yo sabiendo que era positiva algo tiene que haber verdad que yo no … a mí lo primero que se venía en la mente era que yo no tenía nada

ENTREVISTADOR:: OK PENSASTE QUE QUIZAS FUE UN ERROR O ALGO ASI

Paciente: exacto

ENTREVISTADOR:: OK ¿Y POR CUANTO TIEMPO ESTUVISTE PENSANDO ESO?

Paciente: todos estos años entonces como no vi… tu sabes… tampoco quería que me diera nada yo dije pues déjame ir y volver hacerme las pruebas que en verdad

ENTREVISTADOR:: ¿Y ESE MOMENTO FUE ESPONTANTEO QUE LLEGO O YA TU VENIAS PENSANDOLO?

Paciente: no, fue espontaneo

ENTREVISTADOR::Y TE ACUERDAS CUANDO FUE QUE TE LLEGO VERDAD TOMASTE ESA DECISION

Paciente: eso fue como para eso fue par de meses antes de enero algo así por ahí

ENTREVISTADOR:: ¿Y QUE ESTABA PASANDO EN ESE MOMENTO EN TU VIDA?

Paciente: conocí a una persona tu sabes yo conocí a esa persona entonces, que se yo; yo no encontraba la forma de cómo decirle a esa persona mi situación. Entonces, yo en ese momento pues hice un viaje a Estados Unidos que fui a ver a mi hermana y en ese tiempo que yo estuve allá pues este me ponía a pensar como yo encuentro la forma de decirle a esta persona pues lo que yo estoy que tengo hasta que pues me dio el ánimo y se lo dije

ENTREVISTADOR:: OK

Paciente: ve, entonces la pues la persona en ese momento lo acepto y que se yo y me dijo “¿tú te has chequeado?” y yo sí, o sea no volví mas a chequearme ni nada entonces ahí fue en ese momento que yo dije pues déjame ir a a chequearme a ver

ENTREVISTADOR:: ¿ Y LA PERSONA ESTE CONTINUO CONTIGO?

Paciente: estuvimos como un añoasí pero no, la relación no funciono

ENTREVISTADOR:: OK, O SEA QUE HACE POCO QUE

Paciente: hacen como dos años

ENTREVISTADOR::¿HACE DOS MESES, DOS A~OS, QUE ESTUVIERON JUNTOS?

Paciente: no, no como un año pero hacen dos años que ya la relación se terminó

ENTREVISTADOR:: ESTE EH TU FAMILIA ME DICE QUE TU MAMA MURIO ¿HACE POQUITO, VIVIA CONTIGO TU MAMA?

Paciente: o sean vivían a lado

ENTREVISTADOR:: EH TU MAMA SABIA QUE…

Paciente: ella sabía, siempre me lo machacaba como dice uno siempre me decía “ah que si tú no hubieses hecho esto que si esto”

ENTREVISTADOR:: ¿ TE ECHABA LA CULPA?

Paciente: aja

ENTREVISTADOR:: ESTE Y EM DE ALGUNA MANERA TU MAMA VERDAD ¿TU SIENTES QUE EL ECHARTE LA CULPA VERDAD O TENER ESA LA ACTITUD QUE TENIA EN CUANTO DE ALGUNA MANERA T E HACIA, ESOS VEINTIUNA~O, SENTIR COMO QUE NO QUE NO QUERIAS VENIR A HACERTE LOS EXAMENES Y A DARTE SEGUIMIENTO?

Paciente: o sea ella no como te digo este eso fue en estos últimos momentos que ella enfermo y eso que ella pues antes de eso....Si hubo par de veces que si me lo decía pero no años atrás como te digo o sea que fue ya cuando empezó a enfermar y eso

ENTREVISTADOR:: PARECE QUE ERA PARTE DE LA ENFERMEDAD

Paciente: puede ser, puede ser

ENTREVISTADOR:: Y EN ESE TIEMPO VERDAD ¿ALGUNAS AMISTADES QUE TU CREES QUE HAYAN INFLUIDO EN QUE EN AQUEL MOMENTO HAYAS DEJADO DE VENIR A LA CITA?

Paciente: no, nadie lo sabia

ENTREVISTADOR:: SOLAMENTE TU MAMA

Paciente:mi mama mi hermanas sabían de ahí en fuera ahora que mi mama le haya dicho a otras personas pues ahí no se

ENTREVISTADOR:: EH ¿Y AHORA QUIENES SABEN?

Paciente: mi hermana es la única que sabe y un hijo que yo tengo que él sabe pero la nena mía pues no nunca he… y nunca se lo diré

ENTREVISTADOR:: OK, ESTE ¿TU NENA NO NACIO CON LA CONDICION?

Paciente: ah no

ENTREVISTADOR:: OK, ESTE Y DE ALGUNA MANERA VERDAD SI ALGUNA EN AQUEL MOMENTO TUVISTE ALGUN TRATO CON EL PERSONAL QUE A LO MEJOR SE TE HIZO … NO TE GUSTO Y DECIDISTE NO VENIR PORQUE NO QUERIAS VER AL PERSONAL MEDICO O DE ENFERMERIA O ALGO ASI TU SABE…

Paciente: no, no nunca tuve cuando empecé pues no vine mucho así tampoco tu me entiendes cuando la nena mía “deso” a veces mi hermana era la que me la traía a las citas pero que yo nunca tuve problemas así con nadie

ENTREVISTADOR:: AH OK Y POR EJEMPLO ¿ALGUNA OTRA PERSONA QUE HAYA INFLUENCIADO EN QUE TU TE SINTIERAS ASI DE QUE NO...?

Paciente: no

ENTREVISTADOR:: ESTE YO QUIERO AHORA QUE TU PIENSES EN EL AMBIENTE DONDE TU DESENVUELVES, TE DESENVOLVISTESTE DESDE EL 91 HASTA AHORA, VERDAD, ESTE ¿DE QUE MANERA SI ALGUNA ESE AMBIENTE INFLUYO EN TU DECISION DE DEJAR DE VENIR A LAS CITAS O DEJAR DE DARTE SEGUIMIENTO?

Paciente: no, porque donde yo vivo es un sitio bueno y eso, tranquilo y cada cual está en su casa y nadie esta yo soy una persona que tampoco estoy en ..metías en casa de las personas tampoco yo pues siempre en mi casa o sea era del trabajo a mi casa y si acaso iba salía e iba en casa de mi tía que vive en Peñuelas

ENTREVISTADOR:: AJA

Paciente: pero en cuestión del ambiente y eso pues como te digo, es un sitio bueno tranquilo cada cual en su casa y nadie esta aunque yo siempre he sido una persona de dedede la casa como digo yo pero pues da la mala pata como dice uno que me toco esto a mí.

ENTREVISTADOR:: OK, OK ¿ESTE TU TRABAJAS AHORA?

Paciente: no estoy trabajando

ENTREVISTADOR:: ¿HASTA CUANDO TRABAJASTE?

Paciente: trabajé desde… hasta el 2009

ENTREVISTADOR:: OK, QUE TODAVIA NO TE ESTABAS TOMANDO LOS MEDICAMENTOS

Paciente: no

ENTREVISTADOR:: ESTUDIAS AHORA … ¿VAS A ALGUN CURSO?

Paciente: no, en mi casa era atendiendo un hermano que está impedido, que pues mi mama falleció pues

ENTREVISTADOR:: SI, ¿ LO ESTAS CUIDANDO TU ENTONCES?

Paciente si, entre mi hermana y yo lo cuidamos

ENTREVISTADOR:: ¿ VIVES CON TU HERMANA TAMBIEN EN LA CASA?

Paciente: no, no, ella, yo vivo arriba y mi hermana abajo, mi mama vivía al lao

ENTREVISTADOR:: AH QUE ESTAN CERQUITAS

Paciente: si

ENTREVISTADOR::¿ESA ES TU UNICA HERMANA?

Paciente: tengo otra hermana pero esa está enferma también ella tiene una condición la misma condición que tiene mi hermano que está impedido la tiene ella que no se si habrás, quizás hayas oído hablar de ella se llama Hungtiton

ENTREVISTADOR:: SI

Paciente: pues ellos tienen eso entonces mi hermana tenía otra hermana y murió de esa condición el año pasado

ENTREVISTADOR:: AJA ¿CORRE EN LA FAMILIA?

Paciente: si

ENTREVISTADOR:: ESTE EH POR EJEMPLO HAY PERSONAS QUE A VECES CUANDO ME HAN DICHO NO QUE CUANDO VAN AL LUGAR DE DONDE TIENEN SUS SERVICIOS MEDICOS LAS CONDICIONES EN QUE LO VEN COMO QUE LOS DESANIMAN A VENIR O INCLUSIVE A BUSCAR SUS MEDICAMENTOS A HACER SUS SEGUIMIENTOS SUS ANALISIS DE SEGUIMIENTO. ¿TE HA PASADO ESO?

Paciente: no

ENTREVISTADOR:: ESTE¿ALGUN LUGAR VERDAD QUE TE DE ALGUNA MANERA VERDAD TE HAYA DESANIMADO EN TERMINO DEL AMBIENTE EN DONDE TU TE DESENVUELVES O SEA QUE DE ALGUNA MANERA INDIRECTA TE HAYA DESANIMADO A NO VENIR A LAS CITAS MEDICAS?

Paciente: yo siempre las vengo, siempre vengo a las citas médicas

ENTREVISTADOR:: O SEA LO QUE QUIERO DECIR PERDONAME , ES DURANTE ESE TIEMPO QUE ESTUVISTE SIN DARTE EL SEGUIMIENTO LOS VEINTIUNANNNO QUE ESTUVISTE SIN DARTE EL SEGUIMIENTO

Paciente: que si hubo..

ENTREVISTADOR:: ¿ALGUN LUGAR? POR EJEMPLO ESTE HAY PERSONAS QUE DICEN PUES EL AMBIENTE DONDE YO SOCIALIZO A VECES COMO QUE NO ME QUIERO QUEDAR ALLI Y NO QUIERO ESO ME HACE NO IR A DARME SEGUIMIENTO

Paciente: no, porque como te digo yo este yo siempre he sido una persona de mi casa y eso

ENTREVISTADOR:: ESTE VERDAD... AHORA YO QUIERO QUE TU PIENSES EN PUERTO RICO EN COMO ESTAN LAS COSAS AHORA EN PUERTO RICO ESTE EH ¿DE QUE MANERA SI ALGUNA LAS COSAS QUE ESTAN PASANDO EN PUERTO RICO INFLUYEN EN LA DECISIÓN DE ALGUNAS PERSONAS PUEDE SER TU DECISION O DE ALGUNAS OTRAS PERSONAS DE TOMARSE LOS MEDICAMENTOS PARA EL VIH?

Paciente: por recursos me imagino que no porque por ejemplo yo lo digo por mi yo no he tenido problema en cuestión a obtener los medicamentos. Tu sabe’ que quizás a lo mejor la persona que pues decida no no seguir tomándoselas o algo pue’ a lo mejor se quiere morir o tiene muchísimos problemas que verdad

ENTREVISTADOR:: AJA

Paciente: pues eso pues también influye en los problemas este …familiares y todo eso

ENTREVISTADOR:: SI POR EJEMPLO ¿COMO PUEDE INFLUIR LOS PROBLEMAS FAMILIARES?

Paciente: por ejemplo si tu no tienes apoyo de tus familiares o tu… o te discriminan cosas así

ENTREVISTADOR::OK

Paciente: por lo cual lo hay todavía a pesar de … a principio cuando esta enfermedad este salió pues había mucho discrimen todavía pues a lo mejor dicen ay esa persona tiene eso pues no ..”Déjame echarme pa’ aca’ o no hablar con esa persona… muchas personas piensan así

ENTREVISTADOR::¿TE HA PASADO QUE HAS ESCUCHADO A LA GENTE?

Paciente: por lo menos yo no como te digo afuera no hay nadie más que sepa

ENTREVISTADOR:: POR ESO QUE EN TU CASO NO SE DA

Paciente: en mi caso no

ENTREVISTADOR:: Y PARTE DE TU DECISION DE NO DECIRLO,¿ES POR ESO? PORQUE TIENES MIEDO A QUE TE PUEDAN DISCRIMINAR

Paciente: si

ENTREVISTADOR:: OK, ¿ HAS VISTO QUE HAN DISCRIMINADO O ESCUCHAR A LAS PERSONAS HABLAR MAL DE LAS PERSONAS QUE TIENEN LA CONDICION DE VIH?

Paciente: no por lo menos aquí no

ENTREVISTADOR:: ESTE EH ¿TU CREES QUE DE ALGUNA MANERA PUDIERA INFLUIR EL GOBIERNO LAS DECISIONES QUE TOMA EL GOBIERNO EN CUANTO A LAS PERSONAS TIENEN EL ACCESO AL MEDICAMENTO LA DECISION DE TOMARSE EL MEDICAMENTO?

Paciente: el medicamento lo hay y como te digo el que no quiere tomárselo pues no no creo que sea por parte del gobierno

ENTREVISTADOR:: OK

Paciente: porque tu eres la que tiene la decisión de tomártelas o no tomártelas

ENTREVISTADOR:: AJA, ESTE Y LOS SERVICIOS DE SALUD, ¿QUE TU CREES DE LOS SERVICIOS DE SALUD? ¿TU CREES QUE DE ALGUNA MANERA EH EN LOS SERVICIOS DE SALUD HAY BARRERAS PARA QUE LA PERSONA DEJE TOMARSE LOS MEDICAMENTOS O IMPLICAN ALGUNA BARRERA?

Paciente: no creo, como te digo en el poco tiempo que llevo viniendo aquí si me han tratado muy bien y nunca he visto tu sabe hay algo que he visto o he escuchado algo negativo en contra mío tu sabe

ENTREVISTADOR:: SI OK

Paciente: que por esa parte tu sabe no

ENTREVISTADOR:: ESTE TRANSPORTACION ¿EN ALGUN MOMENTO HAS TENIDO PROBLEMA DE TRANSPORTACION?

Paciente: no, hasta ahora no

ENTREVISTADOR::TIENES TU...

Paciente: si yo tengo un carrito

ENTREVISTADOR:: EH ALGUNA SITUACION EN EL PAIS VERDAD QUE SEA SITUACION ECONOMICA O POR EJEMPLO LOS PLANES MEDICOS QUE ¿TU CREES QUE INFLUYAN O SEAN ALGUNA BARRERA PARA QUE LAS PERSONAS DEJEN DE TENER MEDICAMENTOS O DE TOMARSE LOS MEDICAMENTOS.?

Paciente: por lo menos este eh yo no como vuelvo y te digo yo este en el poquito tiempo que llevo pues este tratando esta “desto” no he tenido problema en cuestión a medicamentos no he tenido como se llama el cómo decirte este ay como decirte que no he tenido problemas en cuestión a eso.

ENTREVISTADOR:: AH OK. ESTE AHORA YO QUIERO QUE PIENSES EN LAS COSTUMBRES EN LAS CREENCIAS DE NUESTRA SOCIEDAD ¿DE QUE MANERA SI ALGUNA COSTUMBRES Y CREENCIAS DE NUESTRA SOCIEDAD INFLUYEN EN LA DECISION DE ALGUIEN DE DEJAR TOMAR LOS MEDICAMENTOS?

Paciente: como vuelvo y te digo tu eres la que decides si te las quiere tomar si quieres seguir el tratamiento o no los quieres seguir yo no creo que nadie influya sobre nadie lo digo por mi parte

ENTREVISTADOR:: POR TU EXPERIENCIA HORITA ME HABLASTE QUE A VECES LAS PERSONAS CUANDO SE SIENTEN DISCRIMINADAS VERDAD ESTE QUE A VECES CUANDO SIENTEN QUE LAS DISCRIMINAN PIENSAS ¿QUE ENTONCES ESO PODRIAS SER UNA RAZON COMO ME DIJO ALGUIEN UNA VEZ UNA EXCUSA PARA QUE ALGUIEN PIENSE O DEJE DE TOMARSE EL MEDICAMENTO?

Paciente: no, no creo, yo este al contrario pues ellos pues que piense lo que quieran pero yo voy a seguir mi tratamiento. Voy a seguir tomándome mis medicamentos.

ENTREVISTADOR:: OK, ESTE EH ¿Y EN LOS TIEMPOS QUE ESTAMOS VIVIENDO TU CREES QUE EN ESTOS TIEMPOS QUE ESTAMOS VIVIENDO, CON TODAS LAS COSAS QUE ESTAN PASANDO AHORA, TU CREES... DE QUE MANERA SI ALGUNA VERDAD ESTOS TIEMPOS INFLUYEN EN LA DECISION DE ALGUIEN, PORQUE UNO TOMA LA DECISION COMO TU DICE, PERO HAY COSAS QUE PUEDEN INFLUIR... ESTOS TIEMPOS DE ALGUNA MANERA PIENSAS QUE INFLUYEN EN LA DECISION DE ALGUIEN TOMARSE MEDICAMENTOS POR LAS COSAS QUE ESTAN PASANDO.

Paciente: ay es que no se como contestarte esa pregunta porque vuelvo y te digo este yo... a mi nadie va influir sobre mi... yo como te digo vuelvo... yo tomo mi decisión si me las tomo, si vengo al tratamiento o no. Lo digo por mi cada cual pues tiene su forma de pensar.

ENTREVISTADOR: : SI, OK. ENTONCES Y VOLVIENDO A LA EXPERIENCIA EN QUE TU TOMASTE LA DECISION DE NO VENIR AL SEGUIMIENTO. ALGO QUE ME ESTA BIEN CURIOSO PORQUE ME HA PASADO CON OTRAS PERSONAS CON QUIEN HE HABLADO ESTE QUE CUANDO SI EN EL MOMENTO NO SIENTE QUE TIENEN LOS SINTOMAS PUES A LO MEJOR OTRAS PERSONAS NO SE TOMAN EL MEDICAMENTO O NO VIENEN EH.... ESTE.... DE ALGUNA MANERA EH VERDAD EL, EL.... Y ES QUE ME DA CURIOSIDAD SABER SI, SI, PASABA TIEMPO QUE A LO MEJOR TU NI SIQUIERA PENSABA QUE LA TENIAS O SI TODOS LOS DIAS LO PENSABAS PERO COMO NO TENIAS LOS SINTOMAS ... ME DA CURIOSIDAD TU EXPERIENCIA

Paciente: no, no lo pensaba

ENTREVISTADOR:: ERA COMPLETAMENTE COMO QUE SE...

Paciente: exacto

ENTREVISTADOR:: AH OK QUE INTERESANTE

Paciente: si

ENTREVISTADOR:: Y POR TODOS LOS VEINTIUN A~OS O EN OCASIONES ASI LO PENSABAS

Paciente: si lo pensaba yo decía... “ ¿porque a mí?”-decía yo. Pero que no tu sabe’ era de momento y seguía normal.

ENTREVISTADOR:: Y EN ESOS MOMENTOS EN QUE TE DABA ESO ¿COMO TE SENTIAS? CUANDO LO PENSABAS O TE VENIA A LA MENTE.

Paciente: me sentía mal, me sentía mal yo decia“ ¿Por qué? ¿Por qué a mi, verdad?”

ENTREVISTADOR: : ¿AHORA QUE TE ESTAS TOMANDO LOS MEDICAMENTOS PIENSAS EN ESO MAS FRECUENTEMENTE QUE ANTES?

Paciente : Porque ya como te digo lo he aceptado, hace tiempo pues no pensaba en eso ni tampoco quería aceptarlo

ENTREVISTADOR:: AHORA SI, ESTAS EN ESE PROCESO AHORA, QUE ES UN PROCESO IMPORTANTE, ¿TU ESTAS RECIBIENDO SERVICIOS PSICOLOGICOS ACA?

Paciente: SI yo a veces hablo con ....

ENTREVISTADOR:: Si aja, que ella es una de las manejadoras

Paciente: si

ENTREVISTADOR:: BUENO ME DIJISTE QUE EN EL MOMENTO CUANDO TU DECIDISTE VOLVER A LA CLINICA ERA QUE ESTABAS COMENZANDO UNA RELACION, VERDAD Y ESO TE MOTIVO ¿CREES QUE SI NO HUBIESE ESTADO ESA SITUACION VERDAD DE QUE ESTUVIERAS COMENZANDO LA SITUACION LA RELACION TODAVIA ESTUVIERAS ASI?

Paciente: si

ENTREVISTADOR:: OK ADEMAS DE ESO VERDAD CONCRETAMENTE QUE ESTABAS COMENZANDO LA RELACION ¿ALGO MAS QUE SE A~ADIO A ESO QUE TE HIZO TOMAR LAS FUERZAS PARA VENIR?

Paciente: no, eso fue lo que me motivo a venir

ENTREVISTADOR:: ENTONCES CAMBIANDO CUANDO SE TE OLVIDA TOMARTE ALGUNA DOSIS DEL MEDICAMENTO QUE TE AYUDA A QUE ESO NO PASE... ¿USAS ALGUNA ESTRATEGIA? PARA QUE VERDAD

Paciente: lo que hago pues es que porque yo normalmente me las tomo por las mañanas, desayuno y me las tomo pero que hay de momento que me las tomo más tarde y más tarde es que se me olvida completamente se me olvida.

ENTREVISTADOR:: ¿CUANDO FUE LA ULTIMA VEZ QUE SE TE OLVIDO?

Paciente: antier

ENTREVISTADOR:: UNA VEZ AL DIA, ¿ESTAS BIEN?

Paciente: si estoy bn

ENTREVISTADOR:: ESTE Y ENTONCES ¿HAS PENSADO EN ALGO VERDAD ALGUNA ESTRATEGIA PARA AYUDARTE A QUE NO SE TE OLVIDE

Paciente: sí que en cuanto desayune me las tome, por la mañana para que no se me olvido

ENTREVISTADOR:: Y POR EJEMPLO SI TU AHORA MISMO TE ENCONTRARAS UNA PERSONA QUE ESTE PENSANDO EN DEJAR DE TOMARTE LOS MEDICAMENTOS ¿Qué LE DIRIAS?

Paciente: que no lo haga que esto es para tu salud, esto es para ayudarte. Para que tu puedas este vivir muchos años mas.

ENTREVISTADOR: : ¿ALGO MAS QUE LE RECOMENDARIAS A UNA PERSONA PARA QUE NO DEJE DE TOMAR..

Paciente: que pues le diría también que, que trate de no pensar en cosas negativas porque a veces tu te pones a que se yo... te vienen cosas negativas a la mente tu sabe’ “pues no me voy a tomar nah”

ENTREVISTADOR:: SI

Paciente: que trate pues de no pensar nada negativo

ENTREVISTADOR:: OK ¿DE QUE MANERA, QUE COSAS PODIA HACER PARA NO PENSAR EN ALGO NEGATIVO Y QUE NO LE VENGA LAS COSAS NEGATIVAS? ALGO QUE QUIZAS TE HAYA AYUDADO A TI O A OTRA PERSONA

Paciente: Por lo menos a mi pues yo como me han venido así cosas negativas a la mente, pues yo trato de que no me afecten y digo “no, no voy a dejar que esto me afecte” yo lo digo yo, trato de que no me afecte o sea trato de vencerlo como digo yo. Es como la persona adicta verdad si la persona dice... es como cuando tu fuma. Yo fumaba antes, yo fume muchísimos años y llego el momento en que yo dije aquí ya no voy a fumar más y tome esa decisión y no volví y no fume más. Y llevo todos estos años sin fumar.

ENTREVISTADOR:: FUISTE VALIENTE

Paciente: Si yo dije no, y me daba el deseo de fumar y yo decía no

ENTREVISTADOR:: Y ESO DA FUERTE CUANDO UNO ESTA DEJANDO

Paciente: que sí que nena yo digo no tengo que hacerlo y tengo que hacerlo; y lo logre.

ENTREVISTADOR:: QUE BUENO

Paciente: o sea es fuerza de voluntad que tú tienes que tener es como el adicto si tú no tienes esa fuerza de voluntad de tu salir de ese vicio nadie lo va a hacer.

ENTREVISTADOR:: Y DESDE LA MISMA MANERA DEBE TENER FUERZA DE VOLUNTAD PARA MANTENERSE ENTONCES EN EL... TOMANDOSE EL MEDICAMENTO, SIGUIENDO EL TRATAMIENTO.

Paciente: exacto

ENTREVISTADOR:: CUANDO TU ESCUCHAS LA FRASE ADHERENCIA O CUMPLIMIENTOS DE LAS RECOMENDACIONES MEDICAS ¿CON QUE LA RELACINAS?

Paciente : cumplimiento, adherencia..no entiendo esa palabra

ENTREVISTADOR:: CUMPLIMIENTO

Paciente: ah cumplimiento, pues si te da algunas instrucciones pues tu seguir las instrucciones que el medico te diga. Si te dice pues tomate este medicamento tres veces al día pues se supone que te las tomes tres veces al día.

ENTREVISTADOR:: OK Y ADEMAS DE TOMARTE EL MEDICAMENTO PARA EL VIH ¿QUE OTRAS COSAS TE RECOMIENDAN TU MEDICO O TUS MEDICOS? QUE TE VEN ACA

Paciente: este nada ellos no me dicen nada a mi aquí, na’ que no deje de tomarme los medicamentos.

ENTREVISTADOR: : OK ¿TE MANDAN A HACER EXAMEN MEDICO?

Paciente: si

ENTREVISTADOR:: VERDAD, ¿MAMOGRAFIAS POR EJEMPLO? ELLOS TE MANDAN, TE DAN ESOS REFERIDOS

Paciente: no, aquí no han dao’ mamografía, aquí nunca han dao’ ellos este gine... este aquí te hacen la prueba de la... de ...de ... el PAP

ENTREVISTADOR:: ¿TE LAS HACES AQUÍ, O TIENES QUE IR AL GINECOLOGO?

Paciente: aqui

ENTREVISTADOR:: ¿CUANDO FUE LA ULTIMA VEZ QUE TE LA HICISTE?

Paciente: antes de navidades

ENTREVISTADOR:: ANTES DE NAVIDADES ¿O SEA EL A~O PASADO?

Paciente: Si

ENTREVISTADOR:: Y ESTE ¿MAMOGRAFIAS, TE HAS HECHO ALGUNA?

Paciente: si pero ya hacen como dos años atrás, como dos

ENTREVISTADOR:: OK ESO TE LA HIZO TU GINECOLOGO

Paciente: aja

ENTREVISTADOR:: ¿TODAVIA VAS AL GINECOLOGO?

Paciente : no porque la ginecóloga que yo tenía pues como era privada y ella no coge la reforma pues... y como aquí me hicieron la prueba del ...

ENTREVISTADOR:: PAP OK ESTE LA MAMOGRAFIA NO TE HAN DADO REFERIDO PARA ESO TU NECESITAS REFERIDO ¿VERDAD?

Paciente: Si porque aquí no la hacen

ENTREVISTADOR:: CUANDO LAS CITAS MEDICAS POR EJEMPLO ¿SE TE HA HECHO DIFICIL VENIR ALGUNA O HAS TENIDO QUE FALTAR EN ESTE A~O?

Paciente: ¿para venir aquí?

ENTREVISTADOR:: SI

Paciente: no

ENTREVISTADOR:: Y POR EJEMPLO LOS EXAMENES DE SANGRE...

Paciente: también siempre vengo

ENTREVISTADOR:: O SEA QUE EN ESTE A~O PUES HAS CUMPLIDO CON LAS CITAS LOS EXAMENES LO UNICO QUE TE FALTA SERIA LA MAMOGRAFIA... ¿ALGUNA OTRA CONDICION MEDICA QUE TU TIENES?

Paciente: este tengo diabetis

ENTREVISTADOR:: OK ¿Y COMO ESTAS ALLI?

Paciente: pues yo no se

ENTREVISTADOR:: ¿TE LA ESTAS TRATANDO?

Paciente: porque al principio si tomaba pastillas y eso pero que las pastillas me daban efecto... me daba rápido para ir al baño ...

ENTREVISTADOR:: AH OK

Paciente: y deje de tomármelas y pues y no así así no he vuelto a tratarme na’ más que esto aquí nada más.

ENTREVISTADOR:: SI, OK

Paciente: pero me he chequeao’ con una maquinita que hay en casa y me ha salido bastante bien pa’ como yo la tenía que subía a casi trescientas y pico.

ENTREVISTADOR:: ¿Cuándo FUE LA ULTIMA VEZ QUE TE CHEQUEASTE LA AZUCAR CON TU MAQUINITA?

Paciente: hace como... yo creo que hace como una semana.

ENTREVISTADOR:: SI

Paciente: La tenía en 135

ENTREVISTADOR:: ¿TIENES DIABETES TIPO DOS? ¿Cuánto HACE QUE TE DIAGNOSTICARON CON DIABETES?

Paciente: Cuando yo salí embarazada de mi nena, que salí con diabetes estacionaria. Entonces después no me trate, entonces después me hacia las pruebas y eso y salí que este

ENTREVISTADOR:: MM OK Y POR EJEMPLO EH PRUEBAS PARA EL CARDIALOGO, PARA VER COMO ESTA EL CORAZON, ¿TE HAN DADO REFERIDO PARA ESO?.

Paciente: no

ENTREVISTADOR:: ¿HAY ALGO MAS EN TERMINOS DE LAS CONDICIONES VERDAD DE DIABETES, RECOMENDACIONES QUE A LO MEJOR NO TE HAYA PREGUNTADO QUE QUIERAS A~ADIR DE ESO?

Paciente: no

ENTREVISTADOR:: ¿Y ALGO MAS QUE QUIERAS A~ADIR DE TODO LO QUE HEMOS ESTADO HABLANDO VERDAD QUE NO TE HE PREGUNTADO QUE QUISIERAS A~ADIR?

Paciente: no, no tengo así nada más que añadir.

ENTREVISTADOR:: ENTONCES CULMINAMOS EL PROCESO.

Entrevista número DIEZ

ENTREVISTADOR: ENTREVISTA NUMERO DIEZ AL PACIENTE YA SE LE EXPLICO EL CONSENTIMIENTO INFORMADO. SE LE LEYO Y SE LE EXPLICO Y ACCEDIO A PARTICIPAR EN EL ESTUDIO.

ENTREVISTADOR: ME DIJISTE QUE LLEVABAS NUEVE A~OS CON LA CONDICION ¿VERDAD?

Paciente: si

ENTREVISTADOR: ESTE EH ¿Y EMPEZASTE A TOMARTE LOS MEDICAMENTOS CUANDO TE DIERON EL DIAGNOSTICO O PASO ALGÚN TIEMPO?

Paciente: paso algún tiempo, porque no me los quería beber

ENTREVISTADOR: ¿Cuánto TIEMPO PASO?

Paciente: como dos años

ENTREVISTADOR: ¿DOS A~OS SIN TOMARTE? O SEA YA LOS TENIAS

Paciente: si pero no, no quería ir a las clínicas ni nada de eso

ENTREVISTADOR: OK ESTE ¿Cuántas VECES CUANDO TE DIAGNOSTICARON LA CONDICION EH CUANTAS VECES VINISTE A LA CLINICA A BUSCAR MEDICAMENTOS PARA LA CONDICION?

Paciente: dos veces

ENTREVISTADOR: ¿ Y ESAS DOS VECES QUE VINISTE A BUSCAR LOS MEDICAMENTOS TE LOS TOMASTE O SOLAMENTE LOS BUSCABAS Y NO...

Paciente: los buscaba

ENTREVISTADOR: PERO NO TE LOS TOMABAS

Paciente: no me los tomaba

ENTREVISTADOR: AH OK. ESTE ¿EN EL LUGAR DONDE TE ESTABAN TRATANDO TENIAS LOS MEDICAMENTOS DISPONIBLES?

Paciente: si

ENTREVISTADOR: Y ¿TENIAS QUE ESPERAR, TUVISTE QUE ESPERAR EN ALGÚN MOMENTO PARA TENERLOS O LOS TENIAS ALLI?

Paciente: no, siempre los tenían disponibles

ENTREVISTADOR: OK. ESTE Y LA INFORMACION QUE TE EXPLICA LOS EFECTOS SEGUNDARIOS DEL MEDICAMENTO ¿LOS LEES?

Paciente: no

ENTREVISTADOR: ¿SABES LOS EFECTOS SEGUNDARIOS QUE TIENE?

Paciente: si

ENTREVISTADOR: MAS O MENOS DE CUALES TE ACUERDAS

Paciente: este dan pesadillas, nauseas, diarreas, vomito, dolor de estomago

ENTREVISTADOR: OK

Paciente: así más o menos

ENTREVISTADOR: ¿TE DIO ALGUNO DE ESOS A TI?

Paciente: si

ENTREVISTADOR: ¿TODOS O ALGUNOS?

Paciente: todos

ENTREVISTADOR: TODOS

Paciente: nauseas

ENTREVISTADOR: AH OK Y CUANDO TE DIERON ESOS EFECTOS SEGUNDARIOS ¿COMO BREGASTE CON ELLOS?

Paciente: lo que hacía era que no me los bebía al otro día.

ENTREVISTADOR: AH OK.

Paciente: Pa’ que se fuera acostumbrando mas o menos el cuerpo.

ENTREVISTADOR: ¿ESO FUE UNA INDICACION QUE TE DIO EL MEDICO O TU...?

Paciente: no; yo

ENTREVISTADOR: ¿TU LO DECIDISTE?

Paciente: si

ENTREVISTADOR: ¿ Y QUE PIENSAS SOBRE LOS MEDICAMENTOS PARA TRATAR EL VIH?

Paciente: pues nos ayudan, porque pues te ayudan a bajar tus cargas virales y todo eso, pero no se...

ENTREVISTADOR: ¿PERO QUE? ¿Cuál ES LA OTRA CARA DE LA MONEDA, DEL MEDICAMENTO?

Paciente: Ay es que son muchas, cuando empecé eran muchas pastillas

ENTREVISTADOR: AJA

Paciente: eran seis pastillas diarias, por la mañana y por la noche y pues empecé porque pues estaba embarazada de mi nena, pero no me gustaba

ENTREVISTADOR: OK SI

Paciente: no me gusta

ENTREVISTADOR: OK EH TU NENA, ¿TE DIERON TRATAMIENTO CUANDO... CUANDO ESTABAS EMBARAZADA ES QUE TE DIAGNOSTICARON LA CONDICION?

Paciente: no antes de quedar embarazada

ENTREVISTADOR: AH OK

Paciente: me los bebí obligatoriamente cuando quede embarazada de la nena

ENTREVISTADOR: OK EH ¿QUE TU ESPERAS DEL MEDICAMENTO, DEL TRATAMIENTO PARA EL VIH? ¿Qué EXPECTATIVAS TIENES? ¿QUE TU ESPERAS DE ESO?

Paciente: que me ayude a controlar la enfermedad, si es posible que me la quiten obvio... este que me ayude a tener una vida normal como cualquier otra persona.

ENTREVISTADOR: ¿Y AHORA SIENTES QUE TIENES ESA VIDA NORMAL?

Paciente: si

ENTREVISTADOR: SI

Paciente: si

ENTREVISTADOR: OK. ESTE CUANDO ESCUCHAS COMENTARIOS DE OTRAS PERSONAS QUE HAN DEJADO DE TOMARSE LOS MEDICAMENTOS ¿Qué TU PIENSAS?

Paciente: pues, este antes yo no me los bebía, ahora si voy fiel pero pues le daría consejos. Que se los beban porque es por el bien de ellos. Si tienen hijos pues tienen que velar por sus hijos pero si.

ENTREVISTADOR: OK. Y POR EJEMPLO ESAS PERSONAS A QUIENES TU HAS CONSEJADO ¿Qué RAZONES HAN DADO ESAS PERSONAS PARA DEJARSE DE TOMAR LOS MEDICAMENTOS?

Paciente: prácticamente lo mismo, los efectos segundarios de las pastillas. Que son muchas pastillas, que a veces se les olvido una dosis. Ya se les quita el ánimo de bebérselas de nuevo, prácticamente eso los efectos segundarios y las pastillas que son demasiadas.

ENTREVISTADOR: AJA OK. ESTE Y EN ESE MOMENTO CUANDO TU DEJASTE DE TOMARTE OH BUENO NO TE LOS TOMASTE NUNCA PERO CUANDO TU TOMASTE LA DECISIÓN DE TOMARTELOS Y DE NO VENIR A LA CLINICA ¿Cuál FUE LA RAZON?

Paciente: porque yo no aceptaba lo que me estaba pasando yo lo que tenía era...

ENTREVISTADOR: ¿NO LO ESTABAS ACEPTANDO?

Paciente: ay sí, no lo aceptaba y pues la gente; yo decía que la gente me iba a mirar mal porque la gente se pues es egoísta como uno dice y pues no aceptaba. Venía aquí, venía a la clínica los buscaba pero no me los bebía.

ENTREVISTADOR: Y EN ALGÚN MOMENTO, ¿Cuánto TIEMPO LLEVAS TOMANDOTELOS?

Paciente: ahora ya llevo como dos annos ahí corridos fiel

ENTREVISTADOR: AH CORRIDO PORQUE DESPUES TE LOS TOMABAS PERO TOMABA VACACIONES

Paciente: aja tomaba vacaciones porque me cansaba

ENTREVISTADOR: AH OK ¿Cuántas VECES DEJASTE DE TOMARTELOS? EN ESOS EN ESTOS NUEVE A~OS Y ENTONCES SIETE A~OS QUE ENTONCES DECIDISTE VENIR A LA CLINICA... AY LA PREGUNTA ESTA COMPLICADA

Paciente: (rie)

ENTREVISTADOR: ESTUVISTE NUEVE A~OS CON LA CONDICION

Paciente: aja

ENTREVISTADOR: ENTONCES ESTUVISTE DE ESOS NUEVE, ESTUVISTE DOS A~OS QUE NO, QUE NO VENIAS

Paciente: no

ENTREVISTADOR: ENTONCES A LOS DOS A~OS VENISTE, REGRESASTE Y EMPEZASTE A TOMARTELOS. EN ESOS SIETE A~OS VERDAD QUE ESTUVISTE...QUE REGRESASTE Y HASTA HOY. ¿Cuántas VECES HAS TOMADO ESAS VACACIONES O HAS DEJADO DE TOMARTELOS?

Paciente: casi siempre porque no me gustan los medicamentos, los efectos

ENTREVISTADOR: AH OK

Paciente: no cuajo con ellos como uno dice

ENTREVISTADOR: ¿O SEA QUE SON VARIAS, MUCHAS OCASIONES?

Paciente: si

ENTREVISTADOR: ¿Por cuánto TIEMPO ES LO MAS QUE HAS DEJADO DE ...

Paciente: seis meses free

ENTREVISTADOR: AH OK

Paciente: después vuelvo otra vez y me los bebo fiel. Me canso de ellos y vuelvo de nuevo y así me paso

ENTREVISTADOR: Y FIELMENTE ¿Cuánto HA SIDO EL TIEMPO QUE MAS HAS DURADO TOMANDOTELOS?

Paciente: ¿más durado?

ENTREVISTADOR: SI

Paciente: ahora dos años

ENTREVISTADOR: QUE VAN DOS A~OS OK Y EN ESOS MOMENTO QUE NO ERAS FIEL A LOS MEDICAMENTOS ¿Cuánto TIEMPO ERA LO MAS QUE DURABA?

Paciente: un mes, dos meses y más na’

ENTREVISTADOR: AH OK Y AHÍ TE LOS DEJABAS DE TOMAR

Paciente: ahí me los dejaba de tomar

ENTREVISTADOR: ESTE EN ESTOS DOS A~OS QUE LLEVAS FIELMENTE TOMANDOTELOS ¿EN ALGÚN MOMENTO HAS PENSADO EN DEJAR DE TOMARTELOS?, NO QUE LO HAYAS DEJADO PERO COMO QUE TE PASA POR LA MENTE

Paciente: si

ENTREVISTADOR: ¿Cómo CUANTAS VECES TE HA PASADO ESO?

Paciente: me ha pasado par de veces, dos o tres veces ya

ENTREVISTADOR: OK QUE LO HAS PENSADO

Paciente: si

ENTREVISTADOR: ESTE EH Y POR EJEMPLO EN ESTOS DOS A~OS ¿HAS DEJADO DE TOMARTE DOSIS DE MEDICAMENTO?

Paciente: siempre hay un día que otro que a uno se lo olvida pero

ENTREVISTADOR: AJA

Paciente: pero de lo contrario no, porque se me haya olvidao’ no porque salga de mí de que no me lo quiero tomar.

ENTREVISTADOR:OK Y CUANDO... LO QUE HACES ES QUE TE LOS TOMABAS AL OTRO DIA...

Paciente: al otro día si

ENTREVISTADOR: LAS DOSIS... AHORA MISMO LOS MEDICAMENTOS TE TOMAS UNA VEZ AL DIA

Paciente: una vez al día

ENTREVISTADOR: ¿Cuántas ESTAS TOMANDO?

Paciente: una vez al día, por la noche

ENTREVISTADOR: ¿Cuántas PASTILLAS?

Paciente: una tableta

ENTREVISTADOR: ¿UNA SOLAMENTE? AH PUES TIENES SUERTE NO TIENES MUCHAS

Paciente: si porque me las cambiaron

ENTREVISTADOR: ¿TE LAS CAMBIARON? ¿Y QUE FUE QUE LOS OTROS TENIAS NO TE ESTABAN DANDO RESULTADOS?

Paciente: no, me estaban dando resultados pero... este como estaba embarazada pues tenía que ser seis tabletas al diarias

ENTREVISTADOR: OK

Paciente: pero entonces como me opere pues entonces ahora es una tableta por la noche.

ENTREVISTADOR: QUE ES MAS FACIL VERDAD

Paciente: si

ENTREVISTADOR: AHORA YO QUIERO QUE TU PIENSES EN TODAS ESAS VECES QUE TU DEJASTE DE TOMARTE LOS MEDICAMENTOS, VERDAD, ESTE Y EN ESOS DOS A~OS QUE ESTUVISTE SIN VENIR. VAS A PENSAR PRIMERO EN ESAS EXPERIENCIAS Y ENTONCES QUIERO QUE PIENSES AHORA EN TI COMO PERSONA, VERDAD, SI INCLUIR A OTRAS PERSONAS O CIRCUNSTANCIAS ¿Qué CARACTERISTICAS TUYAS SENTIMIENTOS O PENSAMIENTOS EN ESE MOMENTO, O SEA TU... ¿TU CREES QUE INFLUYERON EN QUE DEJARAS DE TOMARTE LOS MEDICAMENTOS O DEJARAS DE VENIR A LAS CITAS?

Paciente: pues a mi siempre me afectaba que yo siempre estaba sola. Mi mama nunca estaba conmigo ni mi papa tampoco...este no se siempre me sentía sola como si yo no le importara a nadie y como si todo el mundo me mirara como si yo tuviera la condición o algo asi.

ENTREVISTADOR: AJA

Paciente: y pues de verdad que me sentía mal

ENTREVISTADOR: AJA

Paciente: no quería y no podía...ahora por lo menos

ENTREVISTADOR: OK ESTE TE... ¿MUCHA GENTE SABIA QUE TENIAS LA CONDICION O NO?

Paciente: no

ENTREVISTADOR: ¿PERO AUN ASI TU SENTIAS QUE OTRAS PERSONAS TE PODIAN..

Paciente: si

ENTREVISTADOR: RECHAZAR O SEA TE PODIAN ECHAR A UN LADO

Paciente: si yo pensaba que pues na’ más por mirarme así porque tenía una mancha o algo asi en el cuerpo pues que las personas podían saber lo que yo tenía. O si iba a la clínica y había alguien, una persona que yo conocía que lo iba a regar por donde yo vivía y pues todas esas cosas me daban como que pánico venir aquí a bebérmela

ENTREVISTADOR: ¿Qué TE IDENTIFICARAN?

Paciente: aja, que me identificaran

ENTREVISTADOR: Y HORITA ME DIJISTE QUE TU NO ACEPTABAS LA CONDICION ¿Y COMO ES ESO DE NO ACEPTARLA?

Paciente: yo tenía dieciséis años pues no pensaba que me iba a enfermar así tan jovencita ni na’ de eso y pues no tuve nadie que me dijera mira tienes estos riesgos o algo así...una persona que estuviera ahí conmigo: “Mira si haces esto sin esto puedes pasar estas enfermedades” pues nunca tuve a nadie así cerca y cuando me contagie pues se me cayó el mundo.

ENTREVISTADOR: UJUM

Paciente: Pensaba no voy a poder tener hijos, mis hijos me van a salir así enfermos...este todo el mundo me va a rechazar y pues me cayó como que me caí del cielo a la tierra.

ENTREVISTADOR: OK

Paciente: pero sin paracaídas como uno dice

ENTREVISTADOR: AH OK

Paciente: y me afecto mucho

ENTREVISTADOR: OK, ESTE ¿COMO TE ENTENTERASTE? ¿O SEA COMO HICISTE.... QUE TE DIO POR EHH POR HACERTE LA PRUEBA?

Paciente: porque yo tenía un novio que era mayor que yo

ENTREVISTADOR: AJA

Paciente: entonces me habían dicho mira cuídate que vete y chequéate porque a mí me esta que tienes sida

ENTREVISTADOR: AH OK

Paciente: y yo pues no lo creía y pues dije “ay esa es la gente, siempre hablando” . Entonces una vez me dio con ir a chequearme y no fui para hacerme la prueba del VIH, fui para pedir anticonceptivos para evitar los hijos y pues en eso me hicieron el recordatorio de que fuera a tomar las pruebas pertinentes...y cuando me hice las pruebas me dijeron que ... que ...me las pruebas del VIH y a las dos semanas me llamaron a mi casa a decirme los resultado.

ENTREVISTADOR: OK

Paciente: Me llamaron a decirme los resultados y pues me encerraron en mi cuarto y me dijeron que tenía VIH y otras enfermedades más que había salido en ese momento.

ENTREVISTADOR: ESTE Y ENTONCES EN ESE MOMENTO CUANDO TU RECIBISTE LA NOTICIA Y TODO ESO QUE COMENZASTE CON EL TRATARMIENTO... ME IMAGINO QUE TE DIO UN SHOCK Y NO ACEPTABAS ENTONCES...

Paciente: no, no quería, no quería pastillas no quería nada

ENTREVISTADOR: UJUM EH COMO TE SENTIAS EN AQUEL MOMENTO ¿TE ACUERDAS POR LO QUE ESTABAS PASANDO CONTIGO EMOCIONALMENTE COMO TU ESTABAS?

Paciente: estaba que no valí ni medio peso, porque no... y después tenía una amiga, mi mejor amiga pues fui se lo conté a ella le dije mira me está pasando esto y esto...este me dijeron que tengo VIH y que se yo que. Y pues era mi mejor amiga y verdad tenía confianza con ella y ella cogió y fue donde yo vivía y se lo dijo a todo el mundo

ENTREVISTADOR: OK

Paciente: y todo el mundo se enteró, y todo el mundo me miraba como que “mira ahí esta tiene sida que si no te ajuntes con ella que si esto” y pues eso me traumatizo más todavía y por eso era la negación de las pastillas y no, y no.

ENTREVISTADOR: OK Y ¿EN AQUEL MOMENTO PENSABAS EN QUE TE QUERIAS MORIR POR EJEMPLO O DE ALGUNA MANERA?

Paciente: si muchas veces, me paso por la cabeza

ENTREVISTADOR: AJA

Paciente: pero nunca llegue a hacer nada en contra mía ni nada de eso pero si eso me paso por la cabeza

ENTREVISTADOR: OK. ESTE ¿Y QUE TU PENSABAS QUE IBA A PASAR O SEA PORQUE NO TE ESTABAS TOMANDO... NO ESTABAS YENDO A LAS CITAS, NO TE ESTABAS DANDO EL TRATAMIENTO... QUE TU PENSABAS QUE IBA A PASAR EVENTUALMENTE?

Paciente: pues ignorante al fin, yo pensaba que me iba a morir, que pues rápido que me enterara que tenía la condición que me iban a salir llagas, que me iba a poner mal, que me no iba a durar mucho, y pues yo me dejaba pues como si me fuera a morir pues yo me dejaba.

ENTREVISTADOR: AJA

Paciente: y no hacía nada por mí

ENTREVISTADOR: AH OK ESTE EH Y ¿ALGO MAS QUE ESTABA PASANDO EN TU VIDA EN ESE MOMENTO? APARTE DE ENTERARTE DE ESTO VERDAD ¿ALGUNA OTRA SITUACION QUE ESTABA PASANDO EN TU VIDA? ¿EN TU CASA, EN LA ESCUELA O ALGO TU SABES?

Paciente: no aparte de eso no, era una nena normal mi vida estaba normal...cuando me entere ahí fue que se me cayó el mundo.

ENTREVISTADOR: OK Y DESPUES QUE SE TE CAYO EL MUNCO ¿Cómo.. ¿LO PUDISTE RECUPERAR?

Paciente: Si

ENTREVISTADOR: ¿Y COMO FUE ESO?

Paciente: después de eso pasaron como dos años; conocí a mi pareja actual y pues quede embarazada de la nena y no sabía nada yo no le dije nada... y pues después de eso pues empecé a beberme los medicamentos de nuevo cuando iba a tener como cinco o seis meses le dije a él lo que tenía pues me estaba acostando con él, el este me acepto así, no me dijo nada, no me puso ningún pero ni nada y hasta el sol de hoy que prácticamente gracias a el y a mis hijos es que yo me he levantado porque sino...

ENTREVISTADOR: ¿SABE QUE CUANDO QUEDASTE EMBARAZADA FUE QUE COMENZASTE OTRA VEZ A ...

Paciente: a beber me el medicamento

ENTREVISTADOR: A BEBERTE EL MEDICAMENTO, SINO HUBIESES QUEDADO EMBARAZADA ¿TU ESTUVIESES... HUBIESES VENIDO A LA CLINICA?

Paciente: no, y ahora me los bebo y me los bebo por mis hijos porque yo los quiero ver crecer, los quiero ver bien...

ENTREVISTADOR: OK Y ESTE... Y EN ESOS VECES QUE TU ... VERDAD, DESPUES QUE QUEDASTE EMBARAZADA ESPERASTE EL TRATAMIENTO Y DURANTE SIETE A~OS HASTA HOY.... BUENO ME DIJISTE LLEVAS DOS A~OS TOMANDOTELO

Paciente: ujum

ENTREVISTADOR: EN ESE TIEMPO QUE DESPUES QUEDASTE EMBARAZADA QUE EMPEZASTE A TOMAR TRATAMIENTO EN ESAS VECES QUE TU HAS DEJADO DE TOMAR LOS MEDICAMENTOS ADEMAS DE LOS EFECTOS SEGUNDARIOS QUE TU ME HAS MENCIONADO ALGO VERDAD, QUIZAS, HAY ALGO EN TU ESTADO DE ANIMO, ALGO QUE TU PENSABAS, TU SENTIAS QUE DE ALGUNA MANERA INFLUIA PARA QUE TU NO TUVIERAS EL DESEO DE TOMARTE LOS MEDICAMENTOS

Paciente: es que a mí de por si nunca me han llamado la atención las pastillas, ni nada de eso... nunca... no se

ENTREVISTADOR: AJA

Paciente: pa’ mí que no había nada así, lo único los efectos segundarios

ENTREVISTADOR: ¿Y CUANTO TIEMPO TE TOMO ACEPTARLO, PORQUE ME DIJISTE HORITA QUE YA LA ACEPTABAS, ¿O TODAVIA TE FALTA UN POQUITO ACEPTARLO?

Paciente: ay veces que a uno le dan los arranques como uno dice, pero ya sí, es normal es algo normal en mí.

ENTREVISTADOR: ¿Cuánto TIEMPO TE TOMO LLEGAR A ESO?

Paciente: me tomo bastante

ENTREVISTADOR: ¿Y COMO FUE ESE PROCESO DE ACEP... HASTA ACEPTARLA O SEA QUE FUE LO QUE PASO QUE FINALMENTE AHORA PIENSAS BUENO “YA LO ACEPTO O YA ESTOY EN...

Paciente: a mí lo que me hizo así a que lo aceptara que después que di a luz y que se yo que la nena mia salió positiva entonces por ella obligatoriamente tenía que traerla a la clínica para que le dieran su tratamiento y eso

ENTREVISTADOR: ¿UNA NENA O UN NENE?

Paciente: una nena, entonces pues después de ella pues como tenía que traerla aquí obligatoriamente pues si yo quiero que ella este bien pues yo tengo que estar bien, y pues cogi y ahí fue que lo acepte y gracias a Dios la nena me salió bien con los medicamento y todo

ENTREVISTADOR: ¿ESTA EN TRATAMIENTO LA NENA?

Paciente: no

ENTREVISTADOR: ¿YA NO?

Paciente: hasta los tres años estuvo en tratamiento pero ya no

ENTREVISTADOR: ¿Y TODAVIA SIEMPRE LLEVAS A LA NENA?

Paciente: no, ya la dieron de alta

ENTREVISTADOR: AH OK. ESTE AHORA YO QUIERO QUE PIENSES, VAMOS A HABLAR CON LAS PERSONAS CON QUE TU TE RELACIONAS, ESTAN CERCA DE TI. ¿DE QUE MANERA SI ALGUNA, VERDAD, EN ESOS MOMENTOS DONDE TU DEJASTE DE TOMARTE LOS MEDICAMENTOS O DEJASTE DE VENIR A LAS CITAS ¿DE QUE MANERA SI ALGUNA, LAS PERSONAS CON QUE TU TE RELACIONAS PUDIERON HABER INFLUIDO EN QUE TU NO TE HAYAS TOMADO LOS MEDICAMENTOS O NO HAYAS VENIDO A LAS CITAS?; DIRECTA O INDIRECTAMENTE

Paciente: pues prácticamente yo creo que em el .. la ignorancia de las personas de pues “ay si me da un peso se me va a pegar o...

ENTREVISTADOR: AJA

Paciente: cosas asi, pues porque la gente siempre está mal informada

ENTREVISTADOR: SI

Paciente: de lo que en realidad es la.. el VIH y pues eso me dolía mucho

ENTREVISTADOR: OK ¿EL RECHAZO TE DESANIMABA PARA NO TOMARTELO?

Paciente: si, así estaba que no quería saber ni nada ni de nadie

ENTREVISTADOR: ¿ESTAS DEPRIMIDA?

Paciente: si, me sentía deprimida, me sentía sola, me sentía que no le importaba a nadie, y que le daba asco a la gente, me sentía bien mal.

ENTREVISTADOR: ¿Y EN ALGÚN MOMENTO TE TRASTASTE PARA LA DEPRESION?

Paciente: no, nunca

ENTREVISTADOR: OK ESTE EH Y TU FAMILIA, ¿TUVISTE ESA EXPERIENCIA DE TU FAMILIA?, QUE TE RECHAZARAN POR EJEMPLO

Paciente: si, yo vivía con mi bisabuela, ella no ella siempre estuvo ahí conmigo, ella era la que me traía a la clínica, la que me llevaba pa’ aquí “mira la cita”, “mira el medicamento” . Pero en la parte de mi papas; mi mama se vino a enterar que yo tenía VIH hace como un año y mi mama se estaba quedando en mi casa (ella vive en Estados Unidos) y yo le dije lo que tenía y al otro día ella cogió motetes y to’ y arranco y se fue de mi casa con los nenes chiquitos y todo.

ENTREVISTADOR: AJA

Paciente: me dijo no yo no voy a estar aquí porque se me puede pegar algo, porque yo me baño en el mismo baño que tú, porque yo uso las cosas que tú tienes ahí y mi mama cogió y mi rechazo porque yo tenía VIH.

ENTREVISTADOR: QUE FUERTE

Paciente: y mi papa pues este, nunca le ha importao’ porque yo no me crie con ninguno de los dos, pues yo se lo dije para ver si había alguien que dijera “mira yo voy a ti que si esto” y se lo dije también como que no le importo mucho ni no me busca a mí, no busca a su nieto o sea nada no le preocupo.

ENTREVISTADOR: ¿ Y CUANDO PASO ESO PASO TE DESANIMASTE EN TOMARTE LOS MEDICAMENTOS?

Paciente: no

ENTREVISTADOR: ¿O PENSASTE EN DEJAR DE TOMARTE?

Paciente: pensé en dejar pero después dije no, ella me lo dice a mí pero yo tengo que velar por dos nenes que yo tengo y pues cogi y eche pa’ lante. Mi esposo me dijo no mira “echa pa’ lante olvídate de eso, tu nunca has contado prácticamente con ella, porque vino ahora pues no te va a hacer nada” y cogi pues y pensé positivo y dije “no, yo voy a seguir luchando porque yo tengo dos hijos y yo no quiero que le pase nada ni nada” y hasta el sol de hoy.

ENTREVISTADOR: Y ESTE ¿SENTISTE ESO EN ALGÚN MOMENTO DE LOS MEDICOS QUE TE ATIENDEN YA SEA DE AQUÍ O DE ALGUNA OTRO LUGAR, UN RECHAZO?

Paciente: no, parte de médico no, al contrario ellos apoyan a uno te insisten “mira eso es por tu bien” y to’ eso

ENTREVISTADOR: OK O POR EJEMPLO EMM.. EL SERVICIO QUE TE HAYAN DADO DE ALGUNA MANERA SIENTES QUE TE DESANIMA EN DEJAR DE TOMARTE LOS MEDICAMENTOS O QUE PENSARAS EN DEJAR DE TOMARLOS

Paciente: no, fíjate siempre a las clínicas que he ido siempre me han tratado bien, con respeto, aunque pues sepan lo tú tienes pero no te rechazan ni te dicen “mira esta...” siempre te tratan bien.

ENTREVISTADOR: Y ESTE PERSONAL DE ENFERMERIA O PERSONAL QUE ATIENDE LA CLINICA, VERDAD, ¿DE ALGUNA MANERA TE HA DESANIMADO?

Paciente: no, nunca me ha pasao’ siempre han estado ahí apoyándome.

ENTREVISTADOR: ESTE AMISTADES POR EJEMPLO, ME CONTASTE HORITA QUE HABIA AMISTADES INCLUSIVE TU MEJOR AMIGA QUE EN AQUEL MOMENTO LO DIJO... EN ESTE MOMENTO ¿TIENES AMIGOS, TIENES AMISTADES O SEA QUE TU DIGAS SON AMIGOS DE VERDAD?

Paciente: si

ENTREVISTADOR: ¿ALGO QUE HAYAN ECHO TUS AMISTADES DE ALGUNA FORMA TE HAN DESANIMADO PARA QUE NO TE TOMES LOS MEDICAMENTOS O PIENSES EN DEJAR DE TOMARTE EL MEDICAMENTO? REACCIONES DE ELLOS..

Paciente: no, fíjate; las amistades que tengo hasta ahora saben lo que tengo y pues han bregado, han estado ahí conmigo, me han apoyado.

ENTREVISTADOR: AHORA YO QUIERO QUE PIENSES EN EL AMBIENTE EN QUE TU DESENVUELVES, DONDE TU VIVES, DONDE TU SOCIALIZAS TODO ESO ¿DE QUE MANERA SI ALGUNA ESE AMBIENTE INFLUYO EN LA DECISION DE DEJAR DE TOMARTE LOS MEDICAMENTOS, TODAS ESES VECES QUE TOMASTE ESA DECISION?

Paciente: en ese entonces cuando yo no me bebía los medicamentos yo estaba viviendo en otro pueblo y en ese pueblo había que ... no había clínica así de inmunología tenías que ir a otro pueblo que quedaba como una hora de distancia. Entonces para ese entonces yo no tenía carro, no tenía modo de transportación para yo poder llegar a las citas y todo eso. Pues prácticamente en ese lapso de tiempo fue por falta de transportación.

ENTREVISTADOR: ¿SABIAS QUE HAY ALGUNAS CLINICAS QUE TIENEN TRANSPORTACION, EN ALGÚN MOMENTO SABIAN QUE LO TENIAN?

Paciente: no aquí no tenían transportación

ENTREVISTADOR: NO TENIAN EL SERVICIO AH OK. Y POR EJEMPLO LA COMUNIDAD DONDE TU VIVES O DONDE VIVIAS EN ESE TIEMPO, ALGO DE LA COMUNIDAD VERDAD QUE QUIZAS TE DESANIMABA Y QUIZAS INDIRECTAMENTE ESE DESANIMO TE HACIA NO TOMARTE EL MEDICAMENTO O NO CUMPLIR?

Paciente: no, la comunidad estaba bien, no me influía nada lo que si me influía era la falta de transportación.

ENTREVISTADOR: OK. ¿TRABAJAS O ESTUDIAS? ¿O TRABAJABAS O ESTUDIABAS?

Paciente: no, estudiaba después que me entere de la condición deje de estudiar

ENTREVISTADOR: ¿TERMINASTE CUARTO A~O?

Paciente: no, llegue hasta grado diez

ENTREVISTADOR: ¿Y EN ESE TIEMPO HAS TRABAJADO?

Paciente: no. He vendido si por catálogo cosas así pero no un trabajo fijo.

ENTREVISTADOR: Y POR EJEMPLO EL AMBIENTE DONDE TU SOCIALIZAS O JANGUEAS COMO UNO DICE POR ALLI ¿DE ALGUNA MANERA TU CREES QUE HA INFLUENCIADO EN QUE TE HAYAS DEJADO DE TOMAR MEDICAMENTO O TE HAYAS DEJADO DE TOMARTE DOSIS DEL MEDICAMENTO?

Paciente: si, siempre influye porque pues ahora mismo yo vivo lejos de aquí yo no tengo transportacion pero aquí tienen transportación pero las amistades siempre como que influyen un poco en las decisiones que uno toma

ENTREVISTADOR: ¿DE QUE MANERA POR EJEMPLO?

Paciente: pues no se gente negativa, que no hacen nada por uno

ENTREVISTADOR: AJA

Paciente: gente negativa es lo más que se ve ahí.

ENTREVISTADOR: Y POR EJEMPLO ALGO DE ESA GENTE QUE TE PONE ASI NEGATIVA, ¿TE HAN DICHO ALGO O QUE DE ALGUNA MANERA TE HAN INSINUADO ALGO QUE TU PUES COMO QUE TE DESANIMAS?

Paciente: no que te desanima directamente sino que siempre sale el tema de las enfermedades y eso. Y tú ves que dicen cosas que no son reales y aunque tú sepas que no es real como quiera siempre te da

ENTREVISTADOR: TE TOCA

Paciente: TE “SHOKA”

ENTREVISTADOR: ESTE AHORA YO QUIERO QUE PIENSES EN PUERTO RICO LAS COSAS QUE ESTAN PASANDO AQUÍ EN PUERTO RICO ¿DE QUE MANERA LAS COSAS QUE PASAN EN PUERTO RICO INFLUYEN EN LA DECISION YA SEA TUYA O DE OTRA PERSONAS VERDAD, EN DEJAR DE TOMAR LOS MEDICAMENTOS?

Paciente: pues yo creo que es la falta de información sobre las enfermedades que hay...este también este transportacion, este nivel de vida

ENTREVISTADOR: NIVEL DE VIDA ¿Cómo? POR EJEMPLO

Paciente: Personas que no tengan dinero

ENTREVISTADOR: RECURSOS

Paciente: recursos exacto

ENTREVISTADOR: OK Y POR EJEMPLO LA ... ESTE ¿TU CREES QUE DE ALGUNA MANERA INFLUYE EL GOBIERNO, LAS COSAS QUE PASAN EN EL GOBIERNO?

Paciente: puede ser también

ENTREVISTADOR: ¿Y LOS SERVICIOS DE SALUD, DEL PAIS TU CREES QUE DE ALGUNA MANERA INFLUYE O TIENE ALGUNA BARRERA?

Paciente: si yo creo que también los servicios de salud influyen porque la tarjeta del gobierno pues como que chocante

ENTREVISTADOR: OK. LOS PLANES MEDICOS POR EJEMPLO ¿HAS TENIDO ALGUNA DIFICULTAD PARA QUE TE APRUEBEN ALGÚN MEDICAMENTO?

Paciente: si

ENTREVISTADOR: HABLAME UN POQUITO DE ESO

Paciente: he tenido problemas con la tarjeta del gobierno porque necesitaba, cierto tiempo necesitaba la carta que te dice que ...la cubierta especial

ENTREVISTADOR: AH OK

Paciente: la necesitaba y sin eso no me daban los medicamentos porque yo los cogía fuera de la clínica y estuve como dos o tres meses fácil esperando la carta para yo poder tener mis medicamentos. Me quede tres o cuatro meses sin medicamento.

ENTREVISTADOR: OK. ESTE EH Y ALGUNA OTRA SITUACION EN EL PAIS QUE TU CREES QUE PUEDA INFLUIR EN ESO

Paciente: en la falta de..

ENTREVISTADOR: DE POR EJEMPLO DE QUE HAYAN BARRERAS PARA QUE LAS PERSONAS DEJEN DE TOMARSE LOS MEDICAMENTOS... QUE SE A UNA BARRERA SITUACIONES QUE ESTEN PASANDO EN EL PAIS. QUE REPRESENTEN UNA BARRERA PARA QUE LA PERSONA SE DEJE DE TOMAR EL MEDICAMENTO YA SEA EN TU EXPERIENCIAS O DE OTRAS PERSONAS QUE HAYAS ESCUCHADO.

Paciente: Pues no así no yo creo que las más esenciales son las que ya te mencione.

ENTREVISTADOR: ¿CUALES?

Paciente: las que mencione antes

ENTREVISTADOR: AH OK

Paciente: no, se para mi pensar, no tengo más ninguna

ENTREVISTADOR: OK ENTONCES HORITA ME ESTABAS HABLANDO VERDAD DEL DISCRIMEN QUE A VECES LAS PERSONAS DISCRIMINAN, RECHAZAN ESTE EM... COSTUMBRES DE NOSOTROS VERDAD O CREENCIAS QUE ESTE DIGAMOS INFLUENCIADAS POR CREENCIAS RELIGIOSAS TU CREES QUE DE ALGUNA MANERA ESO INFLUYEN EN QUE LAS PERSONAS SE DESANIMEN O SE ANIMEN VERDAD A TOMARSE EL MEDICAMENTO

Paciente: Yo creo que siempre la relación con Dios como que influye que uno pues coja ánimo, se levante del piso, suba diga “no yo estoy haciendo las cosas mal tengo que hacerlo por mis hijos por mi familia. Pero yo creo que eso influye mucho a que las personas como que vuelvan en si.

ENTREVISTADOR: OK ESTE EH Y HORITA TAMBIÉN HABLASTE DE LO SOCIAL DE LA SITUACION ECONOMICA, VERDAD, DEL STATUS ECONOMICO ¿ALGUNA OTRA COSA EN TERMINOS DE COSAS QUE PASAN EN NUESTRA SOCIEDAD COSTUMBRES Y CREENCIAS QUE TU CREAS QUE PUEDAN INFLUIR EN QUE LA PEROSNA SE DESANIME EN TOMARSE LOS MEDICAMENTOS.

Paciente: este no, fíjate no creo lo más que yo pienso es el discrimen. Bueno a mí en lo personal lo más que me ataco fue el discrimen que eso es lo más que tengo en mi casa.

ENTREVISTADOR: OK

Paciente: lo más que tengo experiencia es en el discrimen.

ENTREVISTADOR: Y LOS TIEMPOS QUE ESTAMOS VIVIENDOAHORA CON TODAS ESTAS COSAS QUE ESTAN PASANDO, VERDAD, EN ESTE TIEMPO TU CREES QUE ALGUNAS DE ESAS COSAS QUE ESTEN PASANDO INFLUYAN O SE DESANIME LA PERSONA

Paciente: yo creo que si en parte también, porque pues alrededor de tu comunidad están habiendo cosas feas y pues eso desaniman las personas a que pues se tomen sus medicamentos.

ENTREVISTADOR: AJA ME ESTABAS HABLANDO DE LOS TIEMPOS QUE SE DESANIMAN LAS PERSONAS

Paciente: si pues los tiempos que se están viviendo yo creo que influyen mucho a que las personas se desanimen en beberse sus medicamentos porque tantas cosas feas y todas esas cosas pues influye también un poco.

ENTREVISTADOR: OK HORITA ME DIJISTE QUE CUANDO TE DEJASTE DE TOMAR LOS MEDICAMENTOS O PRIMERAMENTE CUANDO DEJASTE DE VENIR A LOS.. A DARTE SEGUIMIENTO; LO QUE TE AYUDO FUE QUEDARTE EMBARAZADA

Paciente: ujum

ENTREVISTADOR: Y TODAS ESTAS VECES , VERDAD, QUE EH... ADEMAS DE ESA VEZ, TODAS ESAS VECES QUE TU HAS DEJADO DE TOMARTELOS POR SEIS MESES POR UN TIEMPO Y REGRESAS ¿QUE O SEA PORQUE TU VOLVISTE? ¿O SEA PORQUE TE TOMASTE LOS MEDICAMENTOS OTRA VEZ?

Paciente: ¿Qué porque me los tome de nuevo?

ENTREVISTADOR: UJUM

Paciente: pues porque tengo dos personas que necesitan de mí, que si yo no estoy ahí con ellos no va a estar nadie. Por mi salud también porque quiero ver a mis hijos crecer y quiero formar parte de su vida con ellos.

ENTREVISTADOR: OK

Paciente: prácticamente eso fue lo más que me motivo así a que me los bebiera.

ENTREVISTADOR: EN ALGÚN MOMENTO DURANTE ESE TIEMPO QUE DEJABAS DE TOMARTELO TE SENTIAS... ¿TENÍAS ALGÚN SINTOMA TU QUE TU DECIAS ESPERATE ESTE...

Paciente: siempre me daba miedo, pues este “sino me los estoy bebiendo pues cualquier momento se que me voy a morir,” decía yo, en cualquier momento podía pasar algo y pues iba a ser muy tarde para bebérmelos de nuevo. Y después pues cogi ánimo y volvía de nuevo y volvía y los dejaba

ENTREVISTADOR: O SEA QUE TE VENIA ESE PENSAMIENTO COMO QUE TE PODIAS MORIR

Paciente: aja

ENTREVISTADOR: ¿TE VISUALIZABAS EN ALGÚN MOMENTO COMO QUE PODIA PASAR ANTES DE MORIRTE?

Paciente: Me visualizaba como que pues que iba a dejar a mis bebes solo y pues

ENTREVISTADOR: AJA

Paciente: yo decía “pues no puedo dejar a mis hijos solos, tengo que seguir hacia delante, tengo que pensar en mí en mi familia” y pues cogía y me los volvía y me los bebía.

ENTREVISTADOR: ESTE ¿Y QUE LE DIRIAS A UNA PERSONA QUE ESTA PENSANDO EN DEJAR DE TOMARSE LSO MEDICAMENTOS?

Paciente: que pues a pesar de pues las circunstancias, porque pues no es fácil pero que siga pa’ lante que se los beba que piense positivamente por que, no piense en lo que digan los demás ni en el discrimen ni nada de eso. Que pienses pues en su salud y que pienses en sus hijos si tienen hijos, en su familia que son los que pues verdaderamente siempre están ahí con uno.

ENTREVISTADOR: OK ¿ALGOMAS QUE LE RECOMENDARIAS A LAS PERSONAS O A LAS PERSONAS CON QUIEN TU HAS HABLADO QUE COSAS LE RECOMIENDAS?

Paciente: nada que eche pa’lante que con eso pueden vivir toda su vida, tener una vida normal y que pues eso es muy importante para su salud.

ENTREVISTADOR: ¿Y CUANDO ESCUCHAS LA FRASE ADHERENCIA O CUMPLIMIENTO DE LAS RECOMENDACIONES MEDICAS, CON QUE LO RELACIONAS?

Paciente: el seguimiento de...

ENTREVISTADOR: ADHERENCIA O CUMPLIMIENTO DE LAS RECOMENDACIONES MEDICAS

Paciente: ay este seguir la orden que te dice el medicamento

ENTREVISTADOR: OK

Paciente: eh pues me imagino que será que se tomen los medicamentos como el te dice eso mas o menos es?

ENTREVISTADOR: OK SI ¿Y ADEMAS DE TOMARTE LOS MEDICAMENTOS PARA EL VIH QUE MAS RECOMIENDAN TU MEDICO?

Paciente: ah me dice “fulana bebete los medicamentos” porque ya sabe

ENTREVISTADOR: YA SABE QUE...

Paciente: Ya me conoce

ENTREVISTADOR: AH OK ESTE EH TE MANDA A HACER ALGUNOS ESTUDIOS O POR EJEMPLO EXAMENES

Paciente: me han mandado a hacer: examen del corazón, me han mandado a sacar placa, laboratorios normales para ver cómo va evolucionando el cuerpo.

ENTREVISTADOR: AJA Y CUANDO TE DAN ESOS REFERIDOS ¿TE LOS HACES O ALGUNOS DE ESOS NO TE LOS HACES?

Paciente: eh casi nunca, eh casi siempre los que me hago son los CBC y esas cosas así que me mandan a hacer pero lo otro.

ENTREVISTADOR: ¿Y ESO TE LOS HACEN AQUÍ O TE LOS MANDAN A HACER..?

Paciente: no, yo voy al laboratorio

ENTREVISTADOR: AH OK ¿Y ALGUNA RAZON POR LA CUAL SE TE ..NO LOS HACES O NO TE LOS...

Paciente: la falta de transportación

ENTREVISTADOR: OH OK ESTE, ¿TIENEN ALGÚN SERVICIO AQUÍ PARA ESO?

Paciente: si, pero es de tu casa aquí y de aquí a tu casa

ENTREVISTADOR: AH OK OK. ¿LOS QUE TE MANDAN A HACER AQUÍ TE LOS HACES?

Paciente: si

ENTREVISTADOR: ¿Y A LAS CITAS MEDICAS VIENES?

Paciente: si

ENTREVISTADOR: ¿O AVECES SE TE HACE DIFICIL?

Paciente: no, se me hace fácil, porque tienen transportación

ENTREVISTADOR: ¿ESTE EL EXAMEN DEL PAPA NICOLAO TE LOS HAS HECHO?

Paciente: no

ENTREVISTADOR: ¿ESTE CUANTO HACE QUE NO TE LO HACES?

Paciente: Ay es que no me gusta

ENTREVISTADOR: LA ULTIMA VEZ QUE TE LO HICISTE... ¿TE LO HAS HECHO ALGUNA VE?

Paciente: si, la última vez que me lo hice hacen como año y medio

ENTREVISTADOR: OK

Paciente: no va tanto

ENTREVISTADOR: AJA

Paciente: como año y medio

ENTREVISTADOR: O SEA QUE TE TOCARIA ESTE A~O?

Paciente: si

ENTREVISTADOR: ADEMAS DE LA TRANSPORTACION ¿HAY ALGUNA OTRA... VERDAD ALGO QUE TE DESANIME EN HACERTE LOS EXAMENES QUE EL MEDICO TE MANDA A HACER?

Paciente: a mí lo que me impide así hacerme los exámenes es la transportación porque pues donde yo vivo no queda nada cerca, no es camino de aquí allí y ya llegue.... hay que caminar bastante para llegar. Entonces hay que llegar al sitio temprano que lo que más influye es en mi parte es la transportación.

ENTREVISTADOR: HAY ALGO MAS QUE QUIERAS A~ADIR SOBRE EL TEMA QUE ESTAMOS HABLANDO QUE NO HEMOS HABLADO Y QUE TU CREAS QUE ES IMPORTANTE A~ADIRLO?

Paciente: no, yo creo que pues mi experiencia lo que yo he vivido hasta ahora pues seguir echar pa’ lante.

ENTREVISTADOR: ¿ALGO MAS? PUES ENTONCES TERMINAMOS YA EL PROCESO DE ENTREVISTA….

Entrevista #11

Fase 1

ENTREVISTADOR: SE DISCUTIÓ EL CONSENTIMIENTO INFORMADO CON EL PARTICIPANTE, FIRMÓ Y ACCEDIÓ A PARTICIPAR DEL ESTUDIO ASÍ QUE COMENZAMOS CON LA ENTREVISTA. EH… ¿DESDE CUÁNDO ESTAS TOMANDO EL MEDICAMENTO PARA EL VIH?

Participante: Hace 2000… Del 2000 pa ca, como trece años, o sea ahora pa… Después que me diagnosticaron el, la condición…

ENTREVISTADOR: ¿Y CUÁNDO TE DIAGNOSTICARON LA CONDICIÓN, EN QUÉ AÑO?

Participante: En el 2000 (EN EL 2000… ESTÁ BIEN)

ENTREVISTADOR: AH EN EL 2000 TAMBIÉN. O SEA COMENZASTE A TOMARTE LOS MEDICAMENTOS CUANDO TE DIAGNOSTICARON O PASÓ ALGÚN TIEMPO…

Participante: No, rapidito me puse en tratamiento.

ENTREVISTADOR: OK… ¿Y TE LOS TOMASTE RÁPIDO?

Participante: Si (OK).

ENTREVISTADOR: Y DONDE TE ESTABAN TRATANDO, EN EL LUGAR DONDE TE ESTABAN TRATANDO, TENÍAN LOS MEDICAMENTOS DISPONIBLES…

Participante: Si

ENTREVISTADOR: Y POR EJEMPLO VERDAD LOS MEDICAMENTOS TIENEN LA INFORMACIÓN DE LOS EFECTOS SECUNDARIOS… ¿LEES ESA INFORMACIÓN?

Participante: Si, siempre los leo.

DRA. CASTRO. ¿LA ENTIENDES?

Participante: Si

ENTREVISTADOR: ¿DE ALGUNOS EFECTOS SECUNDARIOS QUE TE ACUERDES?

Participante: No, los, seme, primero, al principio me dieron unos medicamentos, después me los cambiaron… (OK)Que fue cuando estuve un tiempo que pues, ese año que estuve sin venir y me los cambiaron, pues me he sentido de lo más bien…(OK)

ENTREVISTADOR: ¿Y QUÉ EFECTOS SECUNDARIOS TE DIO?

Participante: No, gracias… No tengo efectos secundarios, por ahora… (OK)

ENTREVISTADOR: ESTE… ¿QUÉ PIENSAS SOBRE LOS MEDICAMENTOS PARA TRATAR EL VIH?

Participante: Bien

ENTREVISTADOR: ¿Y QUÉ ESPERAS DE ELLOS, DEL TRATAMIENTO?

Participante: Espero que me mantenga la condición, ahí, estable (AJA)…Y no, he, como se llama, este, no detectable… Pues tengo fe en Dios a ver, Dios es el único que puede quitarme esto, verda…

ENTREVISTADOR: Y CUANDO ESCUCHAS COMENTARIOS DE PERSONAS QUE HAN DEJADO DE TOMARSE LOS MEDICAMENTOS PARA EL VIH, QUÉ PIENSAS…

Participante: A veces pienso, me siento, me pongo como triste, sabes, sabes, y a la vez me siento yo pues diferente, como que se dejan de seguir el tratamiento y eso como que a mí me afecta un poco, porque yo como que , la depresión también a veces caigo en depresión también y eso…

ENTREVISTADOR: ¿HAS CAÍDO EN DEPRESIÓN?

Participante: Una vez…Que fue pal tiempo que estuve sin venil (AH OK)… Y volví después, pues, venia, volví otra vez y estuve tratándome con el psicólogo y pues gracias a Dios (OK), pues ahora, este…

ENTREVISTADOR: ESTE… ¿O SEA, QUE EN ESE MOMENTO QUE DEJASTE DE TOMARTE EL MEDICAMENTO FUE QUE TE SENTÍAS DEPRIMIDO Y TOMASTE TRATAMIENTO?

Participante: Tuve eso y logré salir y pues ahora otra vez estoy…

ENTREVISTADOR: Y POR EJEMPLO ESAS PERSONAS QUE ESCUCHAS CUANDO DEJAN, QUE ESTAN PENSANDO EN DEJAR DE TOMARSE LOS MEDICAMENTOS O QUE HAN DEJADO DE TOMARSE LOS MEDICAMENTOS, QUÉ RAZONES DAN ELLOS…

Participante: No sé (¿QUÉ HAS ESCUCHADO?), que no tienen ganas de seguir adelante…

ENTREVISTADOR: ¿ALGUNA OTRA RAZÓN?

Participante: Esa es la (3:30 No entendí)

ENTREVISTADOR: ESTE… ADEMÁS DE DEJAR DE TOMÁRTELOS EN AQUEL MOMENTO… ¿CUÁNTO HACE DE ESO?

Participante: Eso fue un año

ENTREVISTADOR: ¿EN QUÉ AÑO FUE ESO?

Participante: Al dos mil, estamos en(No entendí 3:48), como pal 2011 más o menos.

ENTREVISTADOR: O SEA, QUE ES RECIENTE

Participante: Si

ENTREVISTADOR: ESTE… Y DESDE EL 2000 HASTA EL 2011 EN ALGÚN MOMENTO DEJASTE… ¿HUBO OTRO MOMENTO EN QUE DEJASTE DE TOMARTE LOS MEDICAMENTOS?

Participante: No, estuve una semana pero fue que, en, acá que en la farmacia, que estuvo un tiempo que acá no estuvieron dando las pastillas y ente tenían que mandar las recetas a Bayamón y pa poder desto los medicamentos pues esto una semana…. Que fue acá… Pero que hasta ahora pues esto he estao, con los medicamentos (OK).

ENTREVISTADOR: ESTE Y EM… DURANTE ESE TIEMPO HAS PENSADO O, VERDAD, DURANTE TODO ESE TIEMPO QUE TIENES QUE HAS ESTADO TRATÁNDOTE PARA LA CONDICIÓN, TE HA VENIDO A LA MENTE DEJAR DE TOMARTE LOS MEDICAMENTOS…

Participante: En varias ocasiones pero, he tratao de, de, me han venio cosas a la mente pero no, no, no me dejo llevar por las cosas que me vienen a la mente, ves, si…

ENTREVISTADOR: ¿Y QUÉ COSAS TE VIENEN A LA MENTE?

Participante: Este, vienen cosas que no, de no seguir con el tratamiento, de dejar de venir… Entonces pue, lo que hago es que me olvido me pongo a distraerme la mente con otras cosas… Haciendo cosas así y así pues me mantenio… (OK)

ENTREVISTADOR: O SEA, QUE DENTRO DE LOS 12 AÑOS QUE LLEVAS CON LA CONDICIÓN… ¿CUÁNTAS VECES HAS DEJADO DE TOMARTE LAS…?

Participante: Pues solamente una vez… (UNA VEZ Y…)Que fue ese año que estuve…

ENTREVISTADOR: Y LAS DOS SEMANAS QUE FUE PORQUE NO TENÍAN EL MEDICAMENTO… Y DOSIS, POR EJEMPLO… ¿HAS DEJADO DE TOMARTE DOSIS?

Participante: No

ENTREVISTADOR: ¿CADA CUÁNTO DE TOMAS LAS PASTILLAS?

Participante: Este… Son tres que tomo pero, este, una vez al día… Sae, las tres juntas por la mañana… Después de la comida (OK)…

ENTREVISTADOR: ¿Y ALGUNA VEZ SE TE HA OLVIDADO O VERDAD, POR ALGUNA RAZÓN NO TE LA TOMASTE Y TE LA TUVISTE QUE TOMAR AL OTRO DÍA?

Participante: Si… En dos ocasiones, pues se me olvida y me las tomo por la tarde o al otro día, sí que salgo a veces se me quedan en casa que tengo que salir apurao pues a veces se me quedan y después me las tomo… Me acuerdo cuando llegue a casa o al otro día.

ENTREVISTADOR: EH… A VECES VERDAD A MUCHAS PERSONAS QUE TIENEN LA CONDICIÓN COMO ESTÁBAMOS HABLANDO ORITA, SE LES HACE BIEN DIFÍCIL TOMARSE LAS PASTILLAS Y LAS PREGUNTAS QUE TE VOY A HACER EN ESTE MOMENTO PUES BÁSICAMENTE ES SABER CUÁLES SON LAS RAZONES POR LAS CUALES SE LES HACE DIFÍCIL. PUEDE HABER MUCHAS RAZONES, DIVERSAS RAZONES, QUE ESTÉN DENTRO Y FUERA DEL CONTROL DE UNO. ASÍ QUE PUES, BÁSICAMENTE ESO ES LO QUE VOY A ESTAR PREGUNTANDO AHORA Y LO QUE QUISIERA QUE CONVERSÁRAMOS, VERDAD, CUÁLES SON ESAS RAZONES YA SEAN QUE ESTÉN EN TU CONTROL O FUERA DEL CONTROL DE TI, COMO POR EJEMPLO EL EJEMPLO QUE ME DISTE, QUE ESTUVISTE DOS SEMANAS SIN EL MEDICAMENTO PORQUE NO LO HABÍA, VERDAD, ESO NO TE LOS TOMASTE PERO ESO ESTABA FUERA DE TU CONTROL EN ESE MOMENTO, VERDAD… ASÍ QUE ESO ES LO QUE YO QUIERO QUE, VERDAD, QUE NOSOTROS HABLEMOS. YO QUIERO QUE TU PIENSES EN TI, SABES, SOLA MENTE EN TI UN MOMENTITO PIENSA EN TI SIN INCLUIR OTRAS PERSONAS. ¿CONSIDERAS QUE HAYA HABIDO ALGÚN SENTIMIENTO, PENSAMIENTO O CREENCIA PERSONAL QUE EN ESE MOMENTO INFLUYO EN QUE DEJARAS DE TOMARTE EL MEDICAMENTO, ESE AÑO POR EJEMPLO QUE DEJASTE DE TOMARTE EL MEDICAMENTO?

Participante: Eh… Desanimo, (¿CÓMO?) no tenía ánimo, tu sae que (ME HABLASTE QUE ESTABAS DEPIMIDO), deprimido, la misma depresión pues me encerré en mi casa y no, y me olvidé que…

ENTREVISTADOR: ¿CÓMO TE COMENZÓ LA DEPRESIÓN? ¿QUÉ ESTABA PASANDO EN ESE MOMENTO EN TU VIDA?

Participante: Pues el enzorramiento en mi casa, sae, porque yo estaba, yo siempre salgo y pero entonces de momento como que empecé a encerrarme en mi casa. No salía pa ningún lao y ahí fue que empecé (OK). Y eso pues me llevo a tanta, sae que, se me bloqueó la mente (NO QUERÍAS SALIR DE TU CASA), no quería salir pa ningún lao, no hablaba con nadie, ni con mi hermana así, que es la que siempre está en mi casa conmigo, siempre le digo las cosas, le cuento todo. Y pues, hasta que pues, un día pues mi papá fue a casa y él fue el que me hablo y pude salir (OK), del, del.

ENTREVISTADOR: ¿ELLOS SABEN QUE TÚ TIENES LA CONDICIÓN?

Participante: Si, mi familia sabe de la condición.

ENTREVISTADOR: ¿Y PORQUE TE DEPRIMISTE, QUÉ PASÓ?

Participante: Me venían cosas a la mente, cosas negativas que le vienen a la…

ENTREVISTADOR: QUÉ ESTABAN PASANDO EN TU VIDA EN ESE MOMENTO…

Participante: Si… ¿Por qué me había pasao esto, porque? Eh… y eso fue lo que me llevó a la depresión

ENTREVISTADOR: ¿QUIERES COMPARTIR ALGO DE ESO? ¿TE SIENTES CÓMODO COMPARTIÉNDOLO CONMIGO?

Participante: Pues por una relación que me busqué en la calle, pues, me cogí la condición que tengo (AH OK) entonces pues, la persona, ahora mismo no me acuerdo que fue (no entendí 8:53) pero que, la persona parece que ya sabía dónde yo vivía, parece que ella, me mandaron una cita y así fue que yo vine acá y ahí fue que me hicieron las pruebas y rapidito, este, (Y SALIÓ) entonces pues me empezaron a tratar rápido…

ENTREVISTADOR: ESO FUE EN EL 2000

Participante: Si, fue pal 2000, me parece que es….

ENTREVISTADOR: Y CUANDO, POR EJEMPLO, CUANDO TE DEPRIMISTE HACE UN AÑO FUE QUE…

Participante: Si… Y después de tanto tiempo pue ese, ese, estuve tantos años con el tratamiento que fue un tratamiento que ahora otro tratamiento que tenía unos medicamentos que me tumbaban (AJA), eh, hasta ese tiempo sin venir eso fue ese año pues, el médico me cambió los medicamentos. Ese medicamento pues me está trabajando mejor que el que tenía (AH, OK), es que me ha mantenio (TE HA MANTENIDO ENTONCES) la condición bien, estable y eso…

ENTREVISTADOR: ¿Y CÓMO ESTÁN TUS DEFENSAS?

Participante: Están bien.

ENTREVISTADOR: ¿Y LA CARGA VIRAL?

Participante: También…

ENTREVISTADOR: ESTA BAJITA, NO DETECTABLE…

Participante: No detectable…

ENTREVISTADOR: Y ENTONCES, VERDAD, VOLVIENDO UN POQUITO A ESE AÑO QUE ESTUVISTE DEPRIMIDO. EH, PASÓ ALGO, VERDAD, QUE TÚ PUEDAS DECIR, PASO ESTA COSA Y ME DEPRIMÍ ALGO, VERDAD…. ¿CÓMO TE EMPEZÓ LA DEPRESIÓN?... TÚ TE DISTE CUENTA DESPUÉS, PERO QUE ESTABA PASANDO EN TU VIDA Y EN TU MENTE COMO QUE HIZO QUE TE DEPRIMIERAS.

Participante: (No entendí 10:16) Velda que, no se… No tenía ganas de vivir…

ENTREVISTADOR: ¿TE VINO ASÍ DE REPENTE O FUE ALGO O ALGUIEN VERDAD, ALGUNA SITUACIÓN?

Participante: Fue por una pesada (No entendí 10:32), pues tenía esa condición y esa persona pues se quitó la vida por eso (AH)…. Entonces eso, pues, eso se me metió en la mente y eso fue lo que yo tuve to el tiempo en la mente y eso fue lo que me llevó a la…

ENTREVISTADOR: Y ESA PERSONA TÚ CONOCÍAS, ERA UNA PERSONA ALLEGADA A TI…

Participante: No la conocía como tal, la conocía por otra persona…Que era que otra persona que conozco pues la conocían… (OK) Y eso pues cuando me contó esa situación pues eso me chocó a mi (OK)…

ENTREVISTADOR: ¿QUÉ COSAS PENSABAS SOBRE ESO? ¿SOBRE ESA ACCIÓN QUE HIZO ESA PERSONA?

Participante: Era algo que no se podía hacer… Sae que pa mí no sé, quitarse la vida así que no pue seguir hacia delante, entonces pues y eso (TE IMPACTÓ TANTO) me chocó a mí entonces yo (FUE BIEN FUERTE ESO ENTONCES), contra, como que me dolió… Eh una persona que la vi par de veces así pero que como que algo, si no puede seguir hacia adelante porque va a quitarse la vida así si hay mucho tratamiento hoy en día que uno puede dural (AJA)treinta… Como papi, mi papá dice que, uno no puede estar, te estés tratando, tú duras mucho y eso… Y eso pues papi me empezó a hablar y empezó a hablar y así pues poco a poco pues por él fue que me dio pues espérate poquito puedo salir hasta que hablaba con el psicólogo de aquí y gracias a Dios hasta ahora pues…

ENTREVISTADOR: ¿Y ERA LA PRIMERA VEZ QUE TE DEPRIMÍAS O…?

Participante: Si esa fue esa vez… (NUNCA…) Nunca me había deprimio. Nunca había pasao por eso…

ENTREVISTADOR: PUES FUE BIEN IMPACTANTE ENTONCES, ESO QUE PASÓ.

Participante: Eso es algo que no sé, eso me dio, se me metió en la mente… Trataba de salir y no podía no sé…

ENTREVISTADOR: Y EN ESE MOMENTO, QUE COSAS TU PENSABAS EN ESE MOMENTO APARTE DE… CUANDO ESTABAS ASÍ DEPRIMIDO DE LOS PENSAMIENTOS QUE TÚ TE RECUERDAS QUE TENÍAS QUE PASABAN POR TU MENTE. ¿QUÉ COSAS PASABAN POR TU MENTE?

Participante: No sé cómo que, me dio, pensaba lo mismo que ese muchacho hizo…

ENTREVISTADOR: EN QUITARTE LA VIDA…

Participante: Si

ENTREVISTADOR: ¿ALGUNA VEZ LO INTENTASTE?

Participante: No, no… Lo pensé pero nunca lo intenté.

ENTREVISTADOR: ALGO MÁS QUE PENSABAS VERDAD, QUE TE VENÍA A LA MENTE EN ESE MOMENTO CUANDO ESTABAS ASÍ DEPRIMIDO...

Participante: Pensa… En eso eh…

ENTREVISTADOR: ESTE… Y POR EJEMPLO, ESTABAS CONSIENTE DE QUE EN ALGÚN MOMENTO, PODÍAS ENFERMARTE O SEA, TENÍAS ALGUNA, TU CARGA VIRAL PODÍA SUBIR Y BAJAR LAS DEFENSAS A LO MEJOR PODÍAS COGER ALGUNA ENFERMEDAD OPORTUNISTA… ¿PENSASTE EN ESO DURANTE ESE TIEMPO?

Participante: Si, si…

ENTREVISTADOR: Y QUE, Y QUE…

Participante: Pensé en pues, en que se me desarrollara la enfermedad… Eh (13:25 No entendí) si tu dejas el medicamento o tratamiento pues eso, se con, eso te va a pues te baja toas las defensas y eso pues pasa al SIDA y eso pues, eso pues papi me lo explicó también en ese momento que yo estuve en la depresión (AH OK)…

ENTREVISTADOR: ¿TU NO SABÍAS ESO?

Participante: No… Sae el doctor me había explicao pero que como yo estaba en ese momento así pues se me olvidó… Se me fue to de la ment, entonces pues papi fue el que me hizo caer en tiempo…

ENTREVISTADOR: AH OK… CAER EN TIEMPO CUANDO TE DIJO LO QUE PODÍA PASAR…

Participante: Si, que podía pasar y eso (AJA)…

ENTREVISTADOR: ¿QUÉ COSAS TE EXPLICÓ TU PAPÁ? POR EJEMPLO QUE TU TE ACUERDES, QUE TE IMPACTÓ Y TE HIZO CAER EN TIEMPO.

Participante: Pues que me explicó eso; tienes que cuidarte porque esos medicamentos son pa que tú te mantengas bien pero si te lo dejas de tomar eso es, vas a (14:11 no entendí) el, la condición se va a ir, se te va a ir más avanzada. Y pues, todos los días iba a casa y todos los días me hablaba y después poco a poco fue que me convencer, me convenció de volver otra vez a venil aquí y eso.

ENTREVISTADOR: Y POR EJEMPLO, SI EN ESE MOMENTO TU PENSABAS EN, VERDAD, ESTABAS DEPRIMIDO Y PENSABAS EN MORIR, EN QUITARTE LA VIDA, QUE, Y PUES UNA MANERA DE MORIR ES NO CUMPLIR CON EL TRATAMIENTO VERDAD PORQUE SI NO TE CUIDAS PUEDE VENIR UNA ENFERMEDAD, ESTE, COMO DICEN, UNA ENFERMEDAD OPORTUNISTA… QUÉ DE LO QUE TE DESCRIBIÓ TU PAPÁ TE HIZO COMO QUE SALIR DEL DESEO DE LA MUERTE PORQUE EN ESE MOMENTO TÚ ESTABAS PENSANDO EN QUE QUERÍAS, VERDAD, TENÍAS ESOS PENSAMIENTOS… ESTE, COMO TÚ TE VISUALIZABAS O QUE DE ESO QUE TE DIJO TU PAPÁ TE HIZO CAMBIAR ESE PENSAMIENTO.

Participante: Pues eso, por los nenes míos… Porque yo tengo dos nenes pues están bien gracias a Dios, están bien… Entonces pues me empezó a hablar de los nenes pues ahí fue que yo (AHÍ CAÍSTE) pues, piensa en tus nenes que ellos estén bien y ellos lo saben que tengo la condición y por ellos fue que… El me los mencionó y empezó a decir; mira los nenes tuyos, búscalos… Y ahí fue que yo, eso fue lo más que me hizo caer.

ENTREVISTADOR: ESTE, ALGO MÁS, VERDAD, DE ESA EXPERIENCIA DE CUANDO ESTABAS DEPRIMIDO QUE COMO QUE TU ENTIENDES QUE INFLUYÓ O QUE TE AYUDÓ…

Participante: No eso… Más nada.

ENTREVISTADOR: ESTE, AHORA VAMOS A HABLAR, VAMOS A CAMBIAR DE TÍTULO. VAMOS A HABLAR SOBRE LAS PERSONAS QUE TÚ TE RELACIONAS. DE QUÉ MANERA, SI ALGUNA, LAS PERSONAS CON LAS QUE TÚ TE RELACIONAN O QUE EN ESE MOMENTO CUANDO ESTABAS DEPRIMIDO, INTERACTUABAN CONTIGO, DE QUÉ MANERA INFLUYERON PARA QUE TÚ DEJARAS DE TOMARTE EL MEDICAMENTO.

Participante: Saes, lo que escuchaba… Sabes, los comentarios que decían las personas que pues, te topabas con una persona que está así, y pues porque esa persona iba trauma hablaba de las condiciones y pues de los comentarios que yo escuchaba que ellos decían, pues por eso fue que yo…

ENTREVISTADOR: OK. ¿QUÉ TU ESCUCHABAS?

Participante: O sea que no le importaba la condición de ellos, que a ellos no les importaba si tomarse los medicamentos… Venían y dejaban de venil (AJA). Y eso pues eso como que a mi…

ENTREVISTADOR: HABÍAN PERSONAS QUE DEJABAN DE TOMÁRSELOS… O SEA, TE HABLABAN DE QUE TENÍA VACACIONES DE LOS MEDICAMENTOS Y VOLVÍAN… ¿ESCUCHABAS ESOS COMENTARIOS?

Participante: Si, la persona que… Ya hace tiempo que no la veo, no sé si la persona está ya fuera de Puerto Rico y to, hace tiempito que no sé, pero esa persona pues, fue por motivo de que una de las cosas que yo también estuve …

ENTREVISTADOR: AH OK, ALGUIEN QUE TÚ CONOCÍAS QUE TENÍA LA CONDICIÓN. OK. Y TE HABLABA NEGATIVO…

Participante: Hablaba negativo de sí mismo, de su persona, de otras personas y…

ENTREVISTADOR: Y ESO DE ALGUNA MANERA TE…

Participante: Eso, eso yo me lo metí en la mente y eso pues, y (no entendí 17:42) eso fue también más todavía (LO QUE COLMÓ LA COPA) lo cogí muy a pecho.

ENTREVISTADOR: ¿ESA PERSONA ERA UN FAMILIAR O ERA UNA AMISTAD?

Participante: No, una amistad.

ENTREVISTADOR: ESTE… Y DE TU FAMILIA PASABA ALGO SIMILAR DE QUE TE HABLARAN COSAS NEGATIVAS…

Participante: No, no mi familia no. Mi familia, mi papá, mi hermano (SI)y eso son los más, pues ellos saben la condición. Ellos siempre me hablan (AJA), me aconsejan…

ENTREVISTADOR: POR EJEMPLO, DEL PERSONAL QUE TE ATIENDE AQUÍ, LOS MÉDICOS, ENFERMERÍA, PERSONAL QUE ATIENDE, VERDAD… DE ALGUNA MANERA COMO TE TRATAN O ALGO ASÍ, HAN INFLUENCIADO EN QUE TU PIENSES, O QUE HAYAS DEJADO DE TOMARTE LOS MEDICAMENTOS…

Participante: No, aquí no, no… Me tratan bien, en todo, o sea eh, el doctol, la farmacia y todo eso es… Trabajan Bien.

ENTREVISTADOR: ALGUNA OTRA PERSONA, VERDAD, QUE YO NO TE HAYA PREGUNTADO, DE ALGUNA MANERA… PUES COMO LO QUE ME EXPLICASTE ORITA QUE PUES UNA PERSONA COMO MALA INFLUENCIA, POR DECIRLO ASÍ Y PUES TE LLEVE A PENSAR NEGATIVO DEL TRATAMIENTO O A PENSAR EN DEJAR DE TOMÁRTELO.

Participante: No, no, no…

ENTREVISTADOR: OK. AHORA YO QUIERO QUE PIENSES EN EL AMBIENTE DONDE TÚ TE DESENVUELVES. EH, ¿DE QUÉ MANERA, SI ALGUNA, ESE AMBIENTE INFLUYÓ EN LA DECISIÓN DE DEJAR DE TOMAR MEDICAMENTOS…?

Participante: En donde vivía anteriormente…

ENTREVISTADOR: OK… DONDE TÚ VIVÍAS ANTERIORMENTE… ¿COMO INFLUYÓ DONDE TÚ VIVÍAS?

Participante: Pues era un sitio como encerrao, sae, era como un barrio de Yauco pero que era un sitio que no, no… Es como, tu sae que tu sales a hablar con vecinos pa que te desenvuelvas… Era un sitio que a pesar que era como unos apartamentitos pero nunca salía nadie, esa era una de las razones porque también como que yo me encerré y me enzorré y ahora donde estoy viviendo pues ahora es una urbanización donde siempre hay vecinos afuera y yo pues salgo, cuando estoy en casa, pues, siempre estoy hablando y me mantengo (AJA) en comunicación con los vecinos, me entretengo en casa y…

ENTREVISTADOR: O SEA QUE ENCERRARTE Y VIVIR EN UN LUGAR ASÍ QUE NO HABÍA MUCHA GENTE PUES TE AYUDÓ A ENCERRARTE MÁS…

Participante: Si… Como que más todavía. Pero ahora es diferente, ahora no, ahora que estoy acá pues me siento más, más, más tranquilo, más seguro de mí mismo y eso… Y siempre estoy…Pues siempre en mi casa van mis hermanos, mis sobrinos y me mantengo la mente ocupá en eso y con los consejos que siempre me da mi papá que siempre está en casa. Pues cuando a veces me ven u poquito apagao entonces ya ellos saben y rapidito me llaman… Y rapidito me hablan…

ENTREVISTADOR: ¿ESTÁS TRABAJANDO O ESTUDIANDO ACTUALMENTE?

Participante: Hasta ahora estoy trabajando por ahí limpiando patios…

ENTREVISTADOR: Y VERDAD EN EL MOMENTO EN QUE DEJASTE DE TOMARTE LOS MEDICAMENTOS, TRABAJABAS TAMBIÉN…

Participante: SI, estuve, trabajaba en un súper-mercado

ENTREVISTADOR: Y DE ALGUNA MANERA, EL AMBIENTE DE TRABAJO INFLUYÓ EN ESO MISMO EN CÓMO TE SENTÍAS O EN LA DECISIÓN DE DEJAR DE TOMARTE LOS MEDICAMENTOS. ALGO QUE HAYA PASADO EN EL TRABAJO O EL AMBIENTE DEL TRABAJO…

Participante: El trabajo fue, lo dejé por discrimen. Sae que la vez pasada, (sabían, ellos sabían) sabían la condición pues dejé el trabajo. Me juzgaban, (SI) me miraban de diferente manera, me señalaban y yo pues dejé el trabajo.

ENTREVISTADOR: ¿ELLOS SABÍAN QUE TENÍAS LA CONDICIÓN?

Participante: Si.

ENTREVISTADOR: ¿TODO EL MUNDO?

Participante: Dos personas…

ENTREVISTADOR: OK. Y CÓMO… ¿TÚ LE DIJISTE O FUE QUE OTRA PERSONA LE DIJO?¿CÓMO ELLOS SUPIERON QUE TENÍAS LA CONDICIÓN?

Participante: Pues por otras personas que (QUE LE HABÍA DICHO)… Entonces pues cuando yo iba a trabajar así de (no entendí 22:12) me pasaban por el lao y me señalaban; “ah este está enfermo”. Escuchaba los comentarios de esas personas y hablaban y a mí eso… Dejé el trabajo y eso…

ENTREVISTADOR: Y ESO FUE PARA CUANDO ESTABAS, PARA EL TIEMPO QUE ESTABAS DEPRIMIDO…

Participante: Mas o menos pa ese tiempo…

ENTREVISTADOR: Y LOS COMENTARIOS DE ESAS PERSONAS DE ALGUNA MANERA INFLUYERON EN QUE… ¿TÚ CREES QUE DE ALGUNA MANERA INFLUYERON EN LA MANERA QUE TÚ TE SENTÍAS?

Participante: Si.

ENTREVISTADOR: Y CUANDO ESTABAS TRABAJANDO O VERDAD, CUANDO TE VAS A HACER LOS PATIOS Y ESO, TE TOMABAS LOS MEDICAMENTOS EN EL TRABAJO…

Participante: Eh, este, desayunaba en casa, me los tomo en casa y después es que salgo…

ENTREVISTADOR: PORQUE ES UNA VEZ… ¿VERDAD?

Participante: Si, una vez por la mañana

ENTREVISTADOR: Y EN AQUEL TIEMPO CUANDO TODAVÍA NO HABÍAS DEJADO DE TRABAJAR EN EL SÚPER-MERCADO… ¿TAMBIÉN ERA ASÍ? ¿ERA UNA POR LA MAÑANA NADA MÁS?

Participante: Si.

ENTREVISTADOR: QUE AHÍ NO HABÍA (NO ENTENDÍ 23:07)

Participante: Si no había en eso…

ENTREVISTADOR: NO ENTENDÍ (23:11) OK. Y EL AMBIENTE DONDE TÚ “JANGUEABAS” O EN ESE MOMENTO SOCIALIZABAS… ¿CREES QUE DE ALGUNA MANERA INFLUYÓ EN QUE ESTO, COMO TÚ DICES, TENER ESTOS PENSAMIENTOS NEGATIVOS DE DEJAR DE TOMARTE LOS MEDICAMENTOS?

Participante: No, el ambiente… Si, simplemente donde vivía anteriormente y ese ambiente ahí…

ENTREVISTADOR: LA MANERA COMO ESTABA ASÍ, CERRADO…

Participante: Si

ENTREVISTADOR: Y EL LUGAR, AQUÍ DONDE RECIBES SERVICIOS O ALGÚN OTRO LUGAR QUE HAYAN INFLUENCIADO D ALGUNA MANERA…

Participante: No.

ENTREVISTADOR: POR EJEMPLO A VECES HAY PERSONAS QUE YO HE ESCUCHADO QUE DICEN; “AH TU SABES, DONDE YO RECIBO SERVICIOS O EN ALGUNOS LUGARES COMO QUE ESTÁ TAN DEPRIMENTE EL LUGAR, VERDAD QUE A UNO NO LE DAN NI DESEOS DE IR, O…”. ¿ESO NO TE HA PASADO?

Participante: No, por ahora me siento bien. En el tiempo que llevo viniendo aquí me siento bien aquí (OK).

ENTREVISTADOR: AHORA QUIERO QUE PIENSES EN PUERTO RICO, VERDAD, LAS COSAS QUE ESTÁN PASANDO EN PUERTO RICO. ¿DE QUÉ MANERA, SI ALGUNA, LAS COSAS QUE PASAN EN PUERTO RICO INFLUYEN EN LA DECISIÓN YA SEA TUYA O DE ALGUNA OTRA PERSONA EN DEJAR DE TOMAR LOS MEDICAMENTOS PARA EL VIH?

Participante: Como que no entendí un poco la…

ENTREVISTADOR: ¿DE QUÉ MANERA, VERDAD, SI ALGUNA LAS COSAS QUE PASAN EN PUERTO RICO INFLUYEN EN LA DECISIÓN DE DEJAR DE TOMARSE LOS MEDICAMENTOS?

Participante: ¿Las demás personas?

ENTREVISTADOR: AJA. YA SEA, AQUÍ PUEDE SER, TU PUEDE HABLAR DE TU EXPERIENCIA O QUE TÚ HAS ESCUCHADO O QUE TÚ PIENSES QUE OTRAS PERSONAS.

Participante: Bueno, yo pienso que cada cual tiene su manera diferente de… Tal vez porque no, no sé, no se quieren cuidar más o no se quieren a sí mismos o… Eso pienso.

ENTREVISTADOR: Y POR EJEMPLO, TÚ CREES QUE DE ALGUNA MANERA EL GOBIERNO INFLUYE EN QUE LAS PERSONAS NO SE PUEDAN TOMAR LOS MEDICAMENTOS O NO TENGAN LOS MEDICAMENTOS DISPONIBLES…

Participante: No, yo pienso que el gobierno no influye, es la misma persona (LA PERSONA)…

ENTREVISTADOR: Y LOS SERVICIOS DE SALUD… POR EJEMPLO, CÓMO ESTÁN LOS SERVICIOS DE SALUD, LOS PLANES MÉDICOS… DE ALGUNA MANERA TÚ CREES QUE HAYA ALGUNA BARRERA EN ESO PARA QUE LAS PERSONAS DEJEN DE TOMÁRSELOS…

Participante: No, pa mí que no (OK)… No hay ningún impedimento, sae, con los planes que tenga, pues…

(Hubo una interrupción)

ENTREVISTADOR: ¿Y LA SITUACIÓN ECONÓMICA DEL PAÍS, POR EJEMPLO?

Participante: Si, porque, yo la encuentro que está un poquito mala. Sae, que las cosas en Puerto Rico, está la cosa mala…

ENTREVISTADOR: Y DE ALGUNA MANERA TÚ CREES QUE ESO INFLUYE EN QUE LA PERSONA, QUIZÁ ESO PUEDA SER UNA BARRERA PARA LA PERSONA…

Participante: Pues las personas que no tengan planes médicos, pues… Esto es un tratamiento que es caro… Pues la persona que no tenga plan, se le hace difícil costear su tratamiento, eh esa es otra razón por la que se hace difícil venil y tratarse.

ENTREVISTADOR: ¿CONOCES A ALGUIEN QUE LE HAYA PASADO ESO?

Participante: No, no conozco a nadie…

ENTREVISTADOR: ¿HAS ESCUCHADO?

Participante: Pero que he escuchao ese problema, que hay personas que…

ENTREVISTADOR: QUE HAY GENTE QUE NO TENGA, QUE NO PUEDE PAGAR (que no tienen plan) Y NO CUALIFICAN PARA PLAN MÉDICO…

Participante: No cualifican o no tienen seguro, seguro privado o un plan por lo menos que le cubra el tratamiento…

ENTREVISTADOR: Y TRANSPORTACIÓN POR EJEMPLO… ¿TÚ CREES QUE ESO SEA ALGUNA BARRERA EN ALGUNAS PERSONAS? LA FALTA DE TRANSPORTACIÓN…

Participante: Si la persona no tiene vehículo pues… Pues por lo menos aquí ahora mismo ellos tienen transportación o sea que yo le, y ya pues cuando no me trae… Porque mi papá trabaja aquí mismo, al lao, pues cuando mi papá no desto pues yo llamo al consorcio y ellos pues, no tengo problema en eso…

ENTREVISTADOR: ¿ALGUNA SITUACIÓN EN EL PAÍS QUE TÚ CREES QUE INFLUYE?

Participante: Por ahora no… Que yo sepa no…

ENTREVISTADOR: ME DIJISTE QUE TE HABÍAS SENTIDO DISCRIMINADO EN EL TRABAJO (si, en el trabajo) Y DE ALGUNA MANERO ESO INFLUYÓ EN LA DECISIÓN DE, EN AQUEL MOMENTO EN LA DEPRESIÓN EN LA DECISIÓN DE DEJAR DE TOMARTE LOS MEDICAMENTOS (si, eso fue otra…)… POR EJEMPLO, TÚ CREES QUE, CREENCIAS, A VECES COMO EN LA MANERA QUE UNO, CREENCIAS RELIGIOSAS POR EJEMPLO, INFLUYEN EN LA DECISIÓN DE DEJAR DE TOMARTE LOS MEDICAMENTOS O AL CONTRARIO AYUDA…

Participante: Yo en cuestión así a religión y eso, yo voy a la Pentecostal y gracias a Dios pues hasta ahora me han apoyao… (ELLOS SABEN…) Pues yo hablé con la pastora que, de la iglesia ( no entendí 28:31) y pues y me sentio bien por ahora… No me siento como que, diferente, como que me juzgan, al contrario, siempre oran por mí, siempre que voy, los días que voy me ayudan…

ENTREVISTADOR: Y LA SITUACIÓN SOCIAL… ¿CREES QUE HAYA INFLUIDO EN ALGÚN MOMENTO EN DEJAR DE TOMAR LOS MEDICAMENTOS?

Participante: No sé en qué…

ENTREVISTADOR: POR EJEMPLO EL ESTATUS SOCIAL, ECONÓMICO…

Participante: No, eso no…

ENTREVISTADOR: Y POR EJEMPLO, LOS TIEMPOS QUE ESTAMOS VIVIENDO AHORA MISMO, PIENSA EN ESO, EN LOS MOMENTOS QUE ESTAMOS VIVIENDO AHORA… ¿TÚ CREES QUE DE ALGUNA MANERA LOS TIEMPOS QUE ESTAMOS VIVIENDO EH, IMPONGAN UNA BARRERA PARA QUE LA PERSONA DEJE DE TOMARSE LOS MEDICAMENTOS?

Participante: No, no hay…. Pa mí que no hay barrera, porque el que lucha, el que quiere lucha… Eso si yo quiero dejar de tomarlos es porque yo quiero… Sabes… Pero para todo, todo es posible en esta vida.

ENTREVISTADOR: Y POR EJEMPLO, VERDAD, LAS VECES EN QUE TÚ PENSASTE, PORQUE ME DIJISTE QUE HA HABIDO MOMENTOS EN QUE TE LO ESTÁS TOMANDO…

Participante: Como te dije horita, hay momentos que a veces, como que, trato de otra vez como que venir a la depresión pero me mantengo ahí…

ENTREVISTADOR: O SEA, ESOS MOMENTOS ES PORQUE SIENTES QUE SE TE VA A VOLVER LA DEPRESIÓ.

Participante: Si, como que me vuelve a la mente otra vez pero como te dije, en casa papi siempre… De la familia siempre está en casa y pues son el motivo que me mantiene ahí.

ENTREVISTADOR: ¿CADA CUÁNTO TIEMPO TE PASA ESO? QUE TÚ SIENTES QUE TE VA A VOLVER LA DEPRESIÓN OTRA VEZ…?

Participante: A veces, a veces cuando estoy asi en mi cuarto por las noches, antes de acostarme, a veces pues, me pongo a ver televisión, a veces como que me viene eso de momento.

ENTREVISTADOR: Y QUÉ TE AYUDA, EN ESE MOMENTO QUÉ COSAS TE AYUDAN A SALIRTE DE ESOS PENSAMIENTOS…

Participante: Si estoy solo en mi casa pues me pongo a leer la biblia y se me pasa to eso… Me pongo a leer la biblia hasta que me quedo dormido. Y me levanto más bien al otro día

ENTREVISTADOR: ¿Y QUÉ OTRAS COSAS TE HAN AYUDADO?

Participante: Mis papás en casa, los consejos que me dan…

ENTREVISTADOR: ELLOS TE VEN…

Participante: Me ven, rápido se dan de cuenta cuando me veo un poquito (SI) como que aguantao

ENTREVISTADOR: ALGUNA OTRA COSA QUE…

Participante: Los hijos míos, siempre estoy pensando en ellos…

ENTREVISTADOR: ¿TIENES DOS?

Participante: Si, dos varones… Gracias a Dios que están lo mas bien. Gracias a Dios están lo más saludables y son mi razón también de… (DE EXISTIR) si…

ENTREVISTADOR: CUANDO DEJASTE DE TOMARTE LOS MEDICAMENTOS CUANDO ESTABAS DEPRIMIDO ME DIJISTE QUE TE HABÍA AYUDADO EL HECHO DE QUE TU PAPÁ HABLE CONTIGO, VERDAD (SI)… ¿ALGO MÁS QUE SE UNIÓ A ESO? VERDAD EL HECHO DE QUE TU PAPÁ TE HABLÓ… ¿ALGO MÁS QUE TE AYUDÓ EN ESE MOMENTO A PARTE DE TU PAPÁ? ¿OTRA PERSONA?

Participante: No, mi papá fue el único que se mantuvo ahí (si) hasta que…

ENTREVISTADOR: Y ENTONCES ME DIJISTE HORITA QUE EN EL MOMENTO EN QUE PENSASTE DE DEJARTE DE, EN EL MOMENTO QUE DEJASTE DE TOMÁRTELOS, EH, LO QUE TE AYUDÓ A SEGUIRTE TOMÁNDOTELOS FUE VERDAD EL SABER, TUS HIJOS, ESTE, QUE QUERÍAS SENTIRTE MEJOR… Y ALGO MÁS QUE HAYA AYUDADO EN ESE MOMENTO, ALGO MÁS EN LO QUE TÚ TE AGARRASTE PARA TOMAR LA FUERZA DE VOLUNTAD Y SEGUÍRTELOS TOMANDO.

Participante: Pues pensé en Dios…

ENTREVISTADOR: ¿EN QUÉ?

Participante: En Dios.

ENTREVISTADOR: ¿AHÍ FUE QUE EMPEZASTE A IR A LA IGLESIA?

Participante: Si y ahí fue que yo empecé… Que son una de las cosas que mi papá también me... Como él va a la iglesia y es cristiano, siempre me hablaba… Y cuando pues, cuando salí poco a poco de eso (AJA), empecé a ir a la iglesia y pues ahí me he mantenio… Aunque a veces las cosas, sabes que la mente sele vuelca a uno pero que gracias a Dios que me saca de eso.

ENTREVISTADOR: ¿QUÉ LE DIRÍAS A UNA PERSONA QUE ESTÁ PENSANDO DEJAR DE TOMARSE LOS MEDICAMENTOS?

Participante: ¿Qué le diría? Pues que no lo deje de hacer. Que siga hacia delante que no importa lo que pase que busque de Dios que Dios es que… Si uno lo hace de corazón y se lo pides a Dios de corazón Él te va a sanar pero mientras tanto que siga con el tratamiento. Eso no es nada malo hacerlo, al contrario eso te ayuda a vivir más. Ese es mi consejo que yo le podría dar a las personas que yo sepa, que no lo está haciendo.

ENTREVISTADOR: ¿ALGO MÁS QUE LE RECOMENDARÍAS A ESA PERSONA? ¿HAS TENIDO LA OPORTUNIDAD DE HABLAR CON PERSONAS QUE ESTÁN PENSANDO ESO?

Participante: No, no he tenío la oportunidad de hacerlo… En algún momento lo haría, se lo diría así, sin vergüenza.

ENTREVISTADOR: CUANDO ESCUCHAS LA FRASE “CUMPLIMIENTO DE LAS RECOMENDACIONES MÉDICAS”, CON QUÉ LO RELACIONAS.

Participante: Cumplimiento (DE LAS RECOMENDACIONES MÉDICAS)… Pues yo lo relaciono a la condición. A las recomendaciones que te da el médico lo que él, como se llama eso, lo que, lo que me refiero es el tratamiento, todas las recomendaciones, las direcciones que él te tiene que hacer…

ENTREVISTADOR: Y ENTONCES, ADEMÁS DE TOMARTE EL MEDICAMENTO PARA EL VIH, QUE OTRAS COSAS TE DICE TU MÉDICO QUE HAGAS…

Participante: Que coma saludablemente y que lleve una dieta, que coma bien (¿ALGO MÁS?), que no tome alcohol, cosas así.

ENTREVISTADOR: ¿Y ALGUNAS DE ESAS RECOMENDACIONES QUE TE DA EL MEDICO SE TE HACE DIFÍCIL SEGUIRLA? LA DIETA…

Participante: No, por ahora no, siempre me alimento bien.

ENTREVISTADOR: ¿QUE OTROS EXÁMENES MÉDICOS TE MANDA A HACER…?

Participante: Aquí me han hecho un CBC de la hepatitis y eso que eso me la hacen cada cierto tiempo a ver como estoy.

ENTREVISTADOR: ¿TIENES CONDICIÓN TAMBIÉN DE HEPATITIS?

Participante: No gracias a Dios de to eso salí bien. Si, salí negativo en to eso, en hepatitis A, B y eso. Lo único que salgo es los triglicéridos un poquito alto. Es lo único que siempre he tenido un poco alto pero lo demás gracias a Dios estoy bien.

ENTREVISTADOR: ¿Y QUÉ OTRAS COSAS ÉL TE MANDA A HACER? ¿ESTUDIOS FUERA DE AQUÍ? HAY UNOS QUE TE LOS HACEN AQUÍ (SI AQUÍ) Y HAY OTROS QUE TE LOS HACEN…

Participante: No por (TODOS TE LO SHACEN AQUÍ…). Aquí me los hacen.

ENTREVISTADOR: OK. TE ESTÁN MANDANDO A HACE PRUEBAS POR EJEMPLO PARA VER CÓMO ESTÁ TU CORAZÓN, DIABETES, TODAS ESAS COSAS...

Participante: De la diabetes si, en una ocasión el Doctor de acá y el medico mío primario también (OK). Que yo le dije que yo quería saber cómo estaba de la azúcar y eso (SI) y gracias a Dios estoy bien.

ENTREVISTADOR: Y DE LOS TRIGLICÉRIDOS, QUÉ TE DICE ÉL…

Participante: Tengo que tomal, ahora mismo este me dijo que comprara, este, fish oil, unas pastillas que son de aceite de bacalao pa que eso me va a ayudar pa él no tener que recetarme más pastillas (AH OK). Eh (36:41 no entendí) y la virubina mía, la virubina creo que se llama eso, tiene un nombre raro algo así más o menos, que tenía un poquito alto o bajito (VILIRUBINA), si y me dijo que con eso no tenia problema, que estaba un poquito desto pero con eso no hay problema… Lo único los triglicéridos…

ENTREVISTADOR: O SEA QUE ESTAS TOMANDO MEDICACIÓN PARA LOS TRIGLICÉRIDO.

Participante: No, él me dijo que me comprara esas pastillitas pa no recetalme pastillas pa que tome.

ENTREVISTADOR: Y TE COMPRASTE LAS PASTILLAS…

Participante: Eso, voy a comprarlas cuando valla a…

ENTREVISTADOR: AH, ES HOY…

Participante: Si hoy fue que me lo dieron…

ENTREVISTADOR: O SEA QUE NO TE LA HABÍA RECOMENDADO….

Participante: No, (OK…) hasta hoy después de…

ENTREVISTADOR: ¿TIENES ALGUNA OTRA CONDICIÓN ADEMÁS DEL VIH?

Participante: No, la única condición es esa…

ENTREVISTADOR: POR EJEMPLO, LAS CITAS DEL DENTISTA… ¿VAS AL DENTISTA?

Participante: Si, voy al dentista y…

ENTREVISTADOR: ¿CADA CUÁNTO TIEMPO?

Participante: Ahora, mismo la semana que viene voy, cada…

ENTREVISTADOR: ¿QUÉ OTROS MÉDICO ENTONCES, TE REFIEREN?

Participante: Y el de la vista…

ENTREVISTADOR: AH EL DE LA VISTA…

Participante: Que son los que visito regularmente…

ENTREVISTADOR: ¿Y a ese también estas yendo, has ido?

Participante: Si. No hace tanto que fui a chequearme la vista también y…

ENTREVISTADOR: O SEA QUE ESTAS AL DÍA…

Participante: Al día de la vista, lo único que un poco que, sabes que tengo que estar usando unas gotas porque tengo la vista reseca y eso. Unas gotas que salen un poquito caras pero que me…

ENTREVISTADOR: SABES DONDE LAS VI MÁS BARATAS, EN SAMS…

Participante: Si eso me dijeron; vete a Sams allí que te las consigues más baratas. Pero allí en el mismo oculista eso cobran (SI) un montón de dinero…

ENTREVISTADOR: SI PORQUE A MÍ ME LAS RECETARON TAMBIÉN Y LAS VI MÁS BARATAS EN SAMS… Las citas médicas por ejemplo, este… ¿Has tenido que faltar a alguna cita médica que tengas acá?

Participante: No.

ENTREVISTADOR: O SIEMPRE VIENES…

Participante: Siempre vengo (OK).

ENTREVISTADOR: HAY ALGO MÁS QUE TÚ QUIERAS AÑADIR VERDAD, SOBRE EL TEMA QUE ESTAMOS HABLANDO, QUE TÚ CREES QUE SEA IMPORTANTE…

Participante: Por ahora no, no… Eso era... En otra ocasión sí, pues, en caso de que surja algo pues entonces pues yo vengo aquí y decirte mira pasó esto y esto o me siento así o (OK)…

ENTREVISTADOR: PUES NADA, ENTONCES TERMINAMOS CON LA ENTREVISTA…

Entrevista #12 (Participante masculino)

**ENTREVISTADOR: Entrevista número doce. Se discutió el consentimiento con participante y procedemos a comenzar la entrevista. ¿Cuánto tiempo hace que llevas diagnosticado con la condición?**

Participante masculino 12: Exactamente no me acuerdo, pero hace como…tengo como…desde el 2007, por ahí.

**ENTREVISTADOR: ¿2007?**

Participante masculino 12: Sí.

**ENTREVISTADOR: ¿Desde el 2007?**

Participante masculino 12: Sí, 2007.

**ENTREVISTADOR: ¿Y desde cuando estas tomando los medicamentos?**

Participante masculino 12: Desde ese…

**ENTREVISTADOR: ¿Desde el 2007?**

Participante masculino 12: Sí.

**ENTREVISTADOR: Cuando empezaste a tomarte los medicamentos…o sea, ¿empezaste a tomarte los medicamentos cuando te dieron el diagnostico o paso o tiempo?**

Participante masculino 12: No, rápido.

**ENTREVISTADOR: Rapidito. Y donde te estaban tratando en aquel momento…te estaban dando tratamiento, ¿estaban los medicamentos disponibles?**

Participante masculino 12: Sí.

**ENTREVISTADOR: Ok. Y la información que explica los efectos secundarios de los medicamentos, ¿las lees?**

Participante masculino 12: Sí.

**ENTREVISTADOR: Algunos de esos efectos secundarios…tuviste…**

Participante masculino 12: Sí.

**ENTREVISTADOR: ¿Cuáles tuviste?**

Participante masculino 12: Mareos y dolor de estómago.

**ENTREVISTADOR: Ah, okay. Fueron los mareos que te dieron.**

Participante masculino 12: Sí, los más que me dieron.

**ENTREVISTADOR: ¿Qué piensas sobre los medicamentos para tratar el VIH?**

Participante masculino 12: Son buenos. Y te ayuda de verdad.

**ENTREVISTADOR: ¿Qué esperas del tratamiento para el VIH?**

Participante masculino 12: Que haya la cura. Lo que quiere todo el mundo, la cura. Que venga ya una vacuna o algo que…

**ENTREVISTADOR: Cuando escuchas comentarios de otras personas que han dejado de tomarse los medicamentos para el VIH, ¿qué tú piensas de eso?**

Participante masculino 12: Como yo mismo.

**ENTREVISTADOR: ¿Cómo?**

Participante masculino 12: Como yo. Que a veces los dejo de tomar porque me siento como “ay, Dios”, cansao’ y eso. De momento me dan…se me empiezan a…me siento como cansao’ y eso y ahí es que me doy cuenta de que de verdad necesito los medicamentos.

**ENTREVISTADOR: O sea, que si hay veces que te da…**

Participante masculino 12: Sí. Como ansiedad de que no me…de tanto tomar, como que…

**ENTREVISTADOR: Cuántas veces, dentro el periodo…cuánto tiempo, ya vas para seis años con la condición, ¿verdad?**

Participante masculino 12: A veces he dejado de venir como tres meses, así…cuatro.

**ENTREVISTADOR: Ah, okay. Y en esos tres meses no te has tomado los medicamentos?**

Participante masculino 12: No.

**ENTREVISTADOR: Y por ejemplo, esas personas que tú escuchas que han dejado de tomarse los medicamentos, ¿Qué razones dan esas personas?**

Participante masculino 12: Algunos, porque pues, o los venden…

**ENTREVISTADOR: ¿Venden los medicamentos así?**

Participante masculino 12: Sí. O…o pues, no se los quieren tomar. O los botan, o se sienten, que se yo, con depresiones y eso.

**ENTREVISTADOR: Ah, okay. Nunca había escuchado de que los vendían. Los vendían…¿pero hay gente que se los compran?**

Participante masculino 12: Me imagino que sí.

**ENTREVISTADOR: ¿Y para qué, para otras personas que tienen la condición?**

Participante masculino 12: Sí, exacto. Nunca dicen.

**ENTREVISTADOR: ¿Sí? O sea, personas que probablemente no pueden comprarlos.**

Participante masculino 12: Exacto.

**ENTREVISTADOR: Que tienen…quizás el plan médico…**

Participante masculino 12: Que les falla la reforma, o pues, que tienen otro plan que pues el deducible es muy alto.

**ENTREVISTADOR: Ah, y los venden. En cuanto los venden?**

Participante masculino 12: Ay, no sé.

**ENTREVISTADOR: No sabes. Son bien caros, ¿verdad?**

Participante masculino 12: Sí, carísimos.

**ENTREVISTADOR: Me dijiste que si habías dejado de tomar el tratamiento y, en ocasiones, ¿has pensado en no tomártelos aunque te los tomes? ¿También?**

Participante masculino 12: Sí.

**ENTREVISTADOR: Muchas veces. Me dijiste que habías dejado de tomártelos porque te cansaba, ansiedades.**

Participante masculino 12: Sí, exacto.

**ENTREVISTADOR: ¿Algo más, alguna otra razón? Y dosis, por ejemplo, una vez te estas tomando los medicamentos, ¿has dejado de tomarte dosis?**

Participante masculino 12: A veces, una pastilla…la que no me gusta, pero ni modo.

**ENTREVISTADOR: ¿Sí? ¿Te tomas cuantas pastillas?**

Participante masculino 12: Tres.

**ENTREVISTADOR: Tres. ¿Cada cuánto?**

Participante masculino 12: Diarias.

**ENTREVISTADOR: Unas tres diarias.**

Participante masculino 12: Tres diarias nada más.

**ENTREVISTADOR: Si. ¿Te toca en la mañana, en la tarde, o en la noche?**

Participante masculino 12: A la hora que yo quiera, pero tengo que tomármelo siempre a esa misma hora. O por la mañana o por las tardes o por la noche.

**ENTREVISTADOR: Ah, okay. Una vez tu decidas la hora, siempre…**

Participante masculino 12: Sí, exacto. Siempre tiene que ser a esa hora.

**ENTREVISTADOR: Y son tres, entonces.**

Participante masculino 12: Aja.

**ENTREVISTADOR: Y hay una que no te gusta.**

Participante masculino 12: Sí.

**ENTREVISTADOR: ¿Y qué te hace esa que no te gusta?**

Participante masculino 12: Ay, que me cae mal al estómago. En lo que se me adapto…o sea, en lo que el cuerpo se adapta al medicamento, pues…

**ENTREVISTADOR: Y esa que te cae mal al estómago, ¿para qué es?**

Participante masculino 12: Esa es una de las que…de la…las que baja…baja las defensas…te sube las defensas.

**ENTREVISTADOR: Ah, okay. La que te sube las defensas. O sea, la que te baja la carga viral, esas te las tomas…**

Participante masculino 12: Exacto.

**ENTREVISTADOR: …pero las de las defensa no.**

Participante masculino 12: Aja.

**ENTREVISTADOR: Ah, okay. Pues tu sabes, verdad, que hay muchas personas, que se les hace bien difícil tomarse las pastillas, como por ejemplo lo que tú me estás diciendo. Tú por ejemplo me has dicho que se te ha hecho difícil. Y pues eso muchas veces lo hacen por diversas razones, ¿verdad? Hay unas razones que están en el control de la persona y otras razones que no están en su control.**

Participante masculino 12: Sí.

**ENTREVISTADOR: Porque eso también, yo he escuchado de todo. Yo te voy a hacer una serie de preguntas que va a estar dirigiéndose a diferentes razones, posibles razones, por la cual las personas no se toman los medicamentos. De aquí en adelante, entonces vamos a ver esas posibles razones. Ahora, yo quiero que tu pienses en ti, sin incluir a otras personas o circunstancia, sino tú, verdad. Tu individualmente. ¿Hay algún…verdad, alguna característica tuya, pensamiento o creencia personal que en ese momento, cuando dejaste de tomarte los medicamentos, influyó para que tomaras esa decisión?**

Participante masculino 12: No.

**ENTREVISTADOR: Cuantas veces en ese tiempo…me has dicho que te has dejado de tomar…**

Participante masculino 12: Ay, como cinco o seis veces

**ENTREVISTADOR: Como cinco o seis. Está bien. Y por ejemplo, ¿te acuerdas la primera vez que dejaste de tomártelos?**

Participante masculino 12: Sí. La primera vez que deje de tomármelos era porque estaba como que…ahí estaba tomando más medicamentos. Y como que me hastié. Decía “ay son demasiao’” y me sentía mal.

**ENTREVISTADOR: Ah okay. ¿Cuántos medicamentos tomabas en aquel entonces?**

Participante masculino 12: Uy, como 10.

**ENTREVISTADOR: Wow.**

Participante masculino 12: Diez.

**ENTREVISTADOR: Y eso fue al principio cuando te diagnosticaron…**

Participante masculino 12: Al principio.

**ENTREVISTADOR: O sea, que te dieron los medicamentos… ¿Cuánto tiempo corrido estuviste tomándotelos después que te los dieron…te diagnosticaron y te los dieron en el 2007?**

Participante masculino 12: Estuve, par de años…

**ENTREVISTADOR: Ah, o sea, que fue después…**

Participante masculino 12: Sí.

**ENTREVISTADOR: Y ese medicamento…**

Participante masculino 12: Cuando me daban más me las tomaban y ahora…ahora me dan tres y…

**ENTREVISTADOR: Y no…Okay, y entonces esa primera vez, que dejaste de tomártelos, ¿Qué tu sentías?**

Participante masculino 12: Me sentía bien débil después, y bien flaco.

**ENTREVISTADOR: Eso fue después que dejaste de tomar…**

Participante masculino 12: Rebaje un montón.

**ENTREVISTADOR: ¿Después que dejaste de tomar?**

Participante masculino 12: Aja, después.

**ENTREVISTADOR: Pero cuando dejaste…cuanto tomaste la decisión…te los estabas tomando y dijiste “ay, yo no me los quiero tomar esto”, ¿cómo te sentías en ese momento?**

Participante masculino 12: Ay, sí. Mal conmigo mismo.

**ENTREVISTADOR: …emocionalmente…**

Participante masculino 12: Sí, porque pue’, yo…yo tengo un hijo y pensaba en que iba a ser de mí si no me los tomaba. Que, obviamente, ya cuando uno tiene esa condición depende del tratamiento. Y volvía otra vez y me los tomaba.

**ENTREVISTADOR: ¿Y cómo se siente depender del tratamiento?**

Participante masculino 12: Es horrible, déjame decirte. Tu depender de un medicamento para tu…pue’, seguir la vida, como uno dice. Es difícil pero pue’, ni modo, hay que hacerlo.

**ENTREVISTADOR: Claro, claro. Eso es así. Y qué pensabas, por ejemplo, ¿qué cosas te venían a la mente cuando…cuando, verdad, estabas así, en esa dependencia del medicamento?**

Participante masculino 12: Pues, fíjate, gracias a Dios nunca me dio con matarme. Gracias a Dios, no. Pero me…me…los humores, me cambia de…me ponía de mal humor, no quería hablar con nadie. Quería estar…me encerraba en mi cuarto.

**ENTREVISTADOR: Y esa…y las demás veces, verdad, esa fue la primera vez…las otras demás cinco, seis veces, las otras veces, ¿qué había pasado en tu vida en ese momento…qué estabas pensando?**

Participante masculino 12: No, las otras veces era poca vergüenza, que decía “ay”, me cansaba y no me las tomaba. Sí, las otras veces…pero na’….fíjate, así me las había dejado de tomar hace par de meses y…y vine ahora para buscar medicamento y eso, y el…el enfermero me dice “mira, estas gordito”, y yo pensaba que no. Aunque me siento un poquito menos…porque antes estaba bien gordo. Y esa fue una de las cosas también…porque me puse bien gordo. Yo decía “ay Dios, no”.

**ENTREVISTADOR: ¿Los medicamentos, el efecto secundario…?**

Participante masculino 12: Sí. Comía demasiao’ y me puse bien gordo. Diache, llegue a pesar 140 libras.

**ENTREVISTADOR: Wow.**

Participante masculino 12: Uy, no. Yo no nunca había pesao’ eso.

**ENTREVISTADOR: Sí, sí. Y al verte así, ¿qué tu pensabas?**

Participante masculino 12: Ay, no. Yo dije “uy, no, no puede ser” y deje de tomarme el medicamento.

**ENTREVISTADOR: ¿Y qué es lo que cansa de este tratamiento? ¿Qué cosas son las que cansan?**

Participante masculino 12: El tomarte los medicamentos.

**ENTREVISTADOR: El tomártelo. ¿Depender de eso?**

Participante masculino 12: El depender de eso, exacto. El que ya yo digo “ya, denme una pastilla y ya” y yo voy a ser feliz. Pero todavía no, tengo que tomarme las 3, todavía.

**ENTREVISTADOR: Tienes las 3. Este...había alguna situación que estaba ocurriendo en tu vida en alguna de esas veces que estabas…**

Participante masculino 12: No. Soy una persona bien alegre, yo soy bien…

**ENTREVISTADOR: Okay. Ahora vamos a hablar de las personas con las que tú te relacionas. ¿De qué manera, si alguna, esas personas que te relacionas pudieron haber influido en tu decisión de dejar de tomarte los medicamentos todas esas veces en que habías dejado de tomar…?**

Participante masculino 12: No, ninguna.

**ENTREVISTADOR: ¿Tienes apoyo de tu familia?**

Participante masculino 12: Sí. Ellos me cuidan. Ellos están pendientes.

**ENTREVISTADOR: ¿Conocen de tu condición?**

Participante masculino 12: Mi…mi hermana y mi mamá y mi papá. Y la mamá de mi nene. Mi nene no, porque mi nene era chiquito cuando…tiene 13 años ya. Está allá afuera, nosotros nos divorciamos. Pero tenemos buena relación.

**ENTREVISTADOR: ¿Tienen buena relación?**

Participante masculino 12: Sí.

**ENTREVISTADOR: ¿Alguien…algún miembro de tu familia…quizás indirectamente haya influido en que tú dejaras…tomaras la decisión de dejar de tomar los medicamentos?**

Participante masculino 12: No.

**ENTREVISTADOR: ¿Alguna amistad tuya, por ejemplo?**

Participante masculino 12: No.

**ENTREVISTADOR: Yo, por ejemplo, he tenido personas que me han dicho como que “pues mira, como yo escucho a las amistades que también tienen la condición y dicen pues, que se cansan y todo eso”, de alguna manera eso te lleva…**

Participante masculino 12: No, fíjate.

**ENTREVISTADOR: ¿No ha sido tu caso?**

Participante masculino 12: No, no ha sido mi caso.

**ENTREVISTADOR: A veces, a otras personas también le sucede que digamos, van donde al sitio donde reciben los servicios y ven que un médico no trata como lo esperaba y eso nada más le… es suficiente como para decirle a ellos “no voy a tomar más los medicamentos”, “no voy más para allá”. ¿Te ha pasado algo así?**

Participante masculino 12: No. A mí no…mi doctora es la mejor de Dios. La Dra. Pérez. Ella es bien buena. Ella me aguanta todas las pocas vergüenzas.

**ENTREVISTADOR: ¿Te aguanta todo?**

Participante masculino 12: Sí, pero me regaña, me regaña.

**ENTREVISTADOR: Sí, ¿qué te dice ella cuando te regaña?**

Participante masculino 12: “¿Por qué no te has tomado los medicamentos? ¡Tienes que tomártelos! ¡Tienes que tomártelos!”

**ENTREVISTADOR: Y ellos saben, verdad, cuando no te los tomas.**

Participante masculino 12: Sí, porque había dejado de venir. Y vine a hacer cita y eso, y a sacarme sangre. Me la sacaron ya.

**ENTREVISTADOR: Ah, okay.**

Participante masculino 12: Ellos me castigan. Me dicen “como a ti no te gusta que te saquen sangre, te la tienes que sacar”. Y yo “a pues bien”.

**ENTREVISTADOR: ¿Algún personal, verdad, o alguna otra persona que tu entiendas que por alguna razón indirectamente haya influido en la decisión de…?**

Participante masculino 12: No. Yo soy una persona bien fuerte de mente y a veces yo escucho a la gente hablar de la enfermedad, y yo lo que le digo es que peor es un cáncer que un SIDA…HIV, porque tú puedes vivir con la condición toda tu vida. Pero una persona que tiene cáncer muere…rápido.

**ENTREVISTADOR: ¿Y qué cosas escuchas de esas personas que hablan de la enfermedad?**

Participante masculino 12: A veces hablan cosas negativas de las personas, pero como yo soy una persona bien fuerte, tú entiendes, como ellos no saben tampoco que yo tengo la condición, pues, yo soy de esas personas que defiendo a todo el mundo…sabes, y yo digo “no hables así, no puedes hablar así, porque no conoces de esa condición, no puedes hablar”, y que se yo qué. Me molesto y le salgo y le digo y le explico y le digo. Y ellos “¿cómo tu sabes tanto?”…”porque yo leo”.

**ENTREVISTADOR: O sea, que son personas que no tienen la condición y hablan sobre…**

Participante masculino 12: Sí, aja. Le gusta marginal a la gente.

**ENTREVISTADOR: Y eso en ningún momento, verdad, te…**

Participante masculino 12: No, gracias a Dios, no.

**ENTREVISTADOR: Ahora piensa en el ambiente donde tú te desenvuelves, ¿de qué manera si alguna, influyó en la decisión de dejar de tomarte los medicamentos?**

Participante masculino 12: No, en…a veces en sentido de mi trabajo, por lo menos, cuando salía tarde en la noche, a veces como llegaba cansao’, pues…en parte pues influía porque llegaba cansado y me acostaba a dormir y se me olvidaba…no me los tomaba. Por esa parte.

**ENTREVISTADOR: O sea la dosis…te las tomabas en la noche, entonces.**

Participante masculino 12: Ajá. Me la tomaba en la noche para acostarme, como no siento el dolor en el estómago, pues…me acuesto bien tranquilo.

**ENTREVISTADOR: O sea que no te las tomabas en el trabajo.**

Participante masculino 12: A veces.

**ENTREVISTADOR: Cuando llegabas a tu casa.**

Participante masculino 12: Cuando cerraba tienda, o sea que…

**ENTREVISTADOR: ¿Cuando cerrabas tienda te llevabas los medicamentos?**

Participante masculino 12: No.

**ENTREVISTADOR: O esperabas a llegar…**

Participante masculino 12: No, llegaba a casa y me bañaba y eso y se me olvidaban. Me acostaba a dormir.

**ENTREVISTADOR: Por el cansancio.**

Participante masculino 12: Aja.

**ENTREVISTADOR: Y por ejemplo del ambiente donde socializas o jangeas, como dicen por ahí, de alguna manera ha influido en…por ejemplo, como ese ejemplo que me diste…que te ha pasado?**

Participante masculino 12: Mm.

**ENTREVISTADOR: ¿No? Algún otro lugar que haya...que tú digas “pues mira, que se yo, influyó en la decisión por alguna razón, directa o indirectamente.**

Participante masculino 12: No.

**ENTREVISTADOR: Donde recibes los servicios médicos, por ejemplo, aquí o en algún otro servicios. De alguna otra manera, directa o indirectamente ha influido…**

Participante masculino 12: No. Mira, estoy super bien aquí.

**ENTREVISTADOR: Okay, ahora yo quiero que pienses en Puerto Rico. Cosas que están pasando aquí en Puerto Rico. ¿De qué manera, si alguna, las cosas que pasan en Puerto Rico influyen en la decisión de algunas personas, verdad, en dejar de tomarse los medicamentos para el VIH?**

Participante masculino 12: En las personas…

**ENTREVISTADOR: En las personas…puede ser en ti o en las personas…**

Participante masculino 12: No…bueno en las personas pueden ser así que se sienten marginados o se sienten de menos, tu sabes, lo…los tratan de menos, los hechan pa’l lao’. Así…puede ser…

**ENTREVISTADOR: ¿Has escuchado de personas que le han pasado eso?**

Participante masculino 12: No, no he escuchado.

**ENTREVISTADOR: ¿Conoces a otras personas que también tienen la condición?**

Participante masculino 12: De mi familia, mi tío tiene la…tiene la condición.

**ENTREVISTADOR: ¿Y ustedes hablan sobre eso?**

Participante masculino 12: No, no. Él no sabe que yo la tengo ni…yo sí sé que él la tiene, pero él no sabe…

**ENTREVISTADOR: El no sabe. Okay. Por ejemplo, verdad, tu crees que de alguna manera el gobierno influya en la decisión de una persona, o verdad…**

Participante masculino 12: Bueno…en caso…puede ser, por las ayudas. Si no se las dan.

**ENTREVISTADOR: Y eso de las ayudas…ahora mismo tú recibes todos los…recibes ayuda para todos los medicamentos…**

Participante masculino 12: Sí.

**ENTREVISTADOR: …o tienes…has tenido alguna dificultad con eso…que no los hayas tenido…**

Participante masculino 12: Fíjate, no. He tenido…y me ayudan aquí rápido…la Reforma…pero ahora me pidieron eso…tengo que llevar unos papeles de acá. Y me dan la cubierta especial.

**ENTREVISTADOR: Y en algún momento has tenido algún problema que no llegue algún medicamento…**

Participante masculino 12: Sí, he tenido.

**ENTREVISTADOR: ¿Sí? Cuéntame un poquito de eso.**

Participante masculino 12: Cuando no tengo el medicamento….no ha llegado a la farmacia…ellos rápido me resuelven. Me dicen “tal día ven”…

**ENTREVISTADOR: ¿Quiénes resuelven? ¿Acá en la clínica?**

Participante masculino 12: En la…no, en la farmacia.

**ENTREVISTADOR: En la farmacia.**

Participante masculino 12: Sí, ella me dice, “mira llévate estos poquito, y si tienes en tu casa, pues, tal día vienes para que no tengas que dejar de tomártelos”.

**ENTREVISTADOR: ¿Y alguna vez has tenido que…has pasado un día o varios días sin el medicamento porque no esté disponible?**

Participante masculino 12: Sí. El de la nevera, que a veces se me hace difícil conseguirlo.

**ENTREVISTADOR: Ah, ese de la nevera. ¿Y te han dicho por qué es difícil?**

Participante masculino 12: Es el más caro.

**ENTREVISTADOR: Que a veces las farmacias no…**

Participante masculino 12: Aja, porque hay que pedirlos…ahora hay que pedirlo a San Juan.

**ENTREVISTADOR: Ah, okay.**

Participante masculino 12: Y los traen de allá y a veces es difícil.

**ENTREVISTADOR: Y ese de la nevera, ¿para qué es?**

Participante masculino 12: Ese el que…el que me cae mal.

**ENTREVISTADOR: Ah, ese es el de…el que te sube las defensas.**

Participante masculino 12: Ajá. Las defensas…

**ENTREVISTADOR: ¿Cómo se llama ese?**

Participante masculino 12: Este…

**ENTREVISTADOR: Si te acuerdas.**

Participante masculino 12: Caletra, yo creo que es.

**ENTREVISTADOR: Caletra.**

Participante masculino 12: Si, porque…no, está la Caletra y está el otro, este…como cremita. Pero hace el mismo efecto que la Caletra.

**ENTREVISTADOR: Y por ejemplo, verdad, en esta misma línea, los servicios de salud del país, de alguna manera tú crees que puedan tener…significar alguna barrera para que las personas no puedan tener medicamento y por lo tanto no se los puedan…**

Participante masculino 12: Bueno…ahora, con el nuevo gobernado que él dice, que le va pa’ to’ el mundo, todo el mundo debe estar esperando para que se la dé.

**ENTREVISTADOR: Y en tu experiencia antes de todo esto, verdad…**

Participante masculino 12: Fíjate, yo nunca he tenido problema con la Reforma. Siempre me han hecho mis estudios y eso.

**ENTREVISTADOR: Y, por ejemplo, personas que no tengan la Reforma, ¿has escuchado de alguna…?**

Participante masculino 12: No he escuchado.

**ENTREVISTADOR: No conoces hasta el momento…y que tal, por ejemplo, la situación económica, verdad…**

Participante masculino 12: También esta crítica, mija, mis hermanas viven conmigo y yo tengo que…y ellas lo único que cogen son cupones y yo tengo que…

**ENTREVISTADOR: ¿Tú trabajas ahora?**

Participante masculino 12: Yo soy mesero, sí. Es fuerte, pero pues, son mis hermanas, tengo que ayudarlas.

**ENTREVISTADOR: Y cualificas entonces para la Reforma, aunque tengas el trabajo.**

Participante masculino 12: Sí.

**ENTREVISTADOR: ¿Por la condición o…?**

Participante masculino 12: No, ahora mismo por el trabajo no cualifique, pero ya me dijo que llevara lo de los medicamentos y eso y me lo dan…me lo dan rápido.

**ENTREVISTADOR: Y transportación, la transportación, por ejemplo, ¿tú crees que sea alguna barrera…?**

Participante masculino 12: A veces, sí. Porque para venir pa’ las citas tengo que venir en taxi, porque no tengo carro.

**ENTREVISTADOR: Tú vienes el taxi, entonces.**

Participante masculino 12: Aja, hoy vine en taxi.

**ENTREVISTADOR: Okay. ¿Tú sabes que acá tienen transportación? Ellos te dan…**

Participante masculino 12: Sí.

**ENTREVISTADOR: ¿Nunca la has solicitado?**

Participante masculino 12: No, no. Como casi nadie sabe de mi condición, pues no..no.

**ENTREVISTADOR: Sí, porque…nada más la…que te fuera a buscar la guagua, pues ya ahí se identificaría,**

Participante masculino 12: Aja.

**ENTREVISTADOR: ¿Quiénes saben de tu condición?**

Participante masculino 12: Mis hermanas, mi mamá y mi papá.

**ENTREVISTADOR: Tu familia inmediata.**

Participante masculino 12: Aja.

**ENTREVISTADOR: Okay. Ahora…ahora yo quiero que pienses en cosas como las costumbres, creencias de nuestra sociedad, verdad, cosas así. ¿De qué manera, si alguna, esas costumbres o creencias de la sociedad pueden influir en la decisión de otras personas de dejarse de tomar los medicamentos?**

Participante masculino 12: Bueno, en el sentido que no se sienten con ayuda.

**ENTREVISTADOR: Te voy a poner…**

Participante masculino 12: En el sentido que no se sienten con ayuda, o sea que no le brindan ayuda pue’, se sienten como que…

**ENTREVISTADOR: Tú me dijiste que…orita me hablaste, verdad, que a veces hay personas que hablan negativo…**

Participante masculino 12: Sí.

**ENTREVISTADOR: …que discriminan.**

Participante masculino 12: Que discriminan.

**ENTREVISTADOR: Este… ¿te has sentido discriminado?**

Participante masculino 12: No.

**ENTREVISTADOR: Por ejemplo, quizás… ¿Cómo tú crees que pueda influir las creencias religiosas en la decisión de la persona…de tomarse o dejar de tomarse los medicamentos?**

Participante masculino 12: Yo entiendo que no porque los religiosos son personas que ayudan a las personas que tienen impedimentos o alguna condición, ya sea física o mental.

**ENTREVISTADOR: ¿Has tenido ayuda de ese tipo?**

Participante masculino 12: No, porque no…no me he sentido con la necesidad. Sabe, todavía me siento fuerte.

**ENTREVISTADOR: Okay. ¿Alguna situación social que tú crees que influye?**

Participante masculino 12: No.

**ENTREVISTADOR: Okay. Y los tiempos que estamos viviendo, verdad, ¿de qué manera si algunas estos tiempos pudieran digamos representar alguna barrera para que las personas…no puedan tomarse los medicamentos?**

Participante masculino 12: Yo…yo pienso que pues que debe de haber más ayuda pa’ las personas pobres. Yo…yo no me considero tan pobre, pero otras personas menos que yo. Que necesitan ayuda para pagar su agua, su luz. Sae, que haya más fondos para comida, que hay muchas personas que tienen muchos hijos, muchas familias…

**ENTREVISTADOR: Sí, okay. Okay. Ahora yo quiero saber, verdad, cuando tú dejaste de tomarte los medicamentos, todas estas veces, ¿qué fue lo que te ayudó a volver a tomártelos?**

Participante masculino 12: La necesidad de que tenía que tomármelos porque me sentía débil.

**ENTREVISTADOR: ¿Te sentías débil?**

Participante masculino 12: Sí, me sentía débil.

**ENTREVISTADOR: ¿Y te enfermaste en algún momento?**

Participante masculino 12: Fíjate, no. No me enferme, gracias a Dios.

**ENTREVISTADOR: ¿Pero débil de que no te podías ni levantar?**

Participante masculino 12: Ah, no, si me podía levantar, pero que ya tu sientes el cansancio de que necesitas…de la necesidad de que tienes que…

**ENTREVISTADOR: De que tienes que entonces ir a…**

Participante masculino 12: Exacto.

**ENTREVISTADOR: Y esas veces cuando viniste a…nuevamente a tomarte los medicamentos, verdad, a solicitar la receta, que te hicieron los estudios, ¿cómo salieron los estudios?**

Participante masculino 12: Me bajan, me bajan.

**ENTREVISTADOR: ¿Te bajaban las defensas mucho?**

Participante masculino 12: Ujum.

**ENTREVISTADOR: Y ese era el cansancio que sentías entonces.**

Participante masculino 12: Sí, exactamente.

**ENTREVISTADOR: Alguna otra condición, o algo…**

Participante masculino 12: Yo me estoy…a mí me dio un infarto.

**ENTREVISTADOR: Ah, okay. ¿Cuándo te dio el infarto?**

Participante masculino 12: Eso fue en el 2006.

**ENTREVISTADOR: Antes de que se te diagnosticara la condición.**

Participante masculino 12: Después.

**ENTREVISTADOR: Ah, porque… ¿fue en el 2007, la condición?**

Participante masculino 12: No, fue antes.

**ENTREVISTADOR: Ah, fue antes. Okay, está bien. Y el infarto fue a raíz de…**

Participante masculino 12: Del estrés. Tenía mucho estrés.

**ENTREVISTADOR: Y el infarto fue… ¿un infarto cerebral?**

Participante masculino 12: Sí, cerebral.

**ENTREVISTADOR: ¿Estuviste hospitalizado?**

Participante masculino 12: Ujum.

**ENTREVISTADOR: ¿Por cuánto tiempo?**

Participante masculino 12: Estuve como una semana o dos.

**ENTREVISTADOR: ¿Dos?**

Participante masculino 12: Me dio una convulsión.

**ENTREVISTADOR: ¿Estaba ligado de alguna manera a los medicamento o a la condición, o no?**

Participante masculino 12: Sí, tomo también medicamentos para…ahora sí tengo que tomar.

**ENTREVISTADOR: Pero el infarto fue a consecuencia directa o indirecta de la condición del VIH…**

Participante masculino 12: No.

**ENTREVISTADOR: … ¿o fue otra condición aparte?**

Participante masculino 12: Aparte.

**ENTREVISTADOR: Ah, okay. Además de sentir el cansancio, algo más que te ayudo a tomarte…a tomar la decisión…**

Participante masculino 12: El cansancio, el bajo peso, no comía.

**ENTREVISTADOR: No te daba hambre.**

Participante masculino 12: Apetito no. Perdida del pelo.

**ENTREVISTADOR: ¿Sí, también?**

Participante masculino 12: Sí. Este…tenía sudores…sae, sudas fríos mucho, así también.

**ENTREVISTADOR: ¿Y eso por no tomarte…?**

Participante masculino 12: Por no tomarme los medicamentos.

**ENTREVISTADOR: Cuando te bajan las defensas.**

Participante masculino 12: Aja, exacto.

**ENTREVISTADOR: Y ahora mismo, ¿Qué le dirías a una persona que está pensando en dejar de tomarse los medicamentos?**

Participante masculino 12: Ay, que se los tome. De veras que se los tome. Es fuerte, pero hay que tomárselos.

**ENTREVISTADOR: ¿Algo más que le dirías?**

Participante masculino 12: No, que…que siga viviendo, porque imagínate sae’, esto no es una enfermedad, esto es una condición que uno puede vivir toa’ la vida. Digo yo, ve. Ser uno feliz, ser alegre y contento. Yo soy bien contento, a mí no me…si te digo que si tengo momentos de tristeza, son muy pocos. Sae’, tengo más alegrías que tristezas. No tengo a mi hijo aquí conmigo, porque está en Estados Unidos, y no lo he visto hace cinco años, pero no soy persona de que me dejo vencer, que me creo la enfermedad. Que, pues porque no vea a mi hijo me voy a morir, no. Él va a seguir viviendo, yo voy a seguir viviendo también. No voy a ahogarme, a echar pa’ atrás porque no lo veo, porque él no quiere hablar conmigo por teléfono, porque él sea un mal hijo, no. Yo vivo la vida día a día, de verdad. Como si fuera el último día. Me lo gozo igual. Mi trabajo…mi trabajo me encanta, yo soy bien contento, yo donde quiera que voy la gente me conoce, o sea que yo donde quiera que voy siempre…yo deposito un granito de alegría o de…porque yo siempre, sabe soy bien amable con todo el mundo. La gente se lleva conmigo. Sae’, soy una persona bien contenta. No soy un aborrecio’ ni nada, soy bien alegre.

**ENTREVISTADOR: ¿Algo más que le dirías a las personas? Cuanto tú escuchas la frase “cumplimiento de las recomendaciones médicas”, ¿con qué la relacionas?**

Participante masculino 12: Con que *tengo* que venir a las citas, *tengo* que tomarme los medicamentos, *tengo* que hacerme los laboratorios, porque es obligatorio para beneficio mío como tal, porque yo soy el que tengo la condición.

**ENTREVISTADOR: Y además de tomarte los medicamentos para el VIH, ¿qué otras cosas te recomienda el médico o la doctora que te atiende?**

Participante masculino 12: Depende, porque si estoy bajo de peso o algo me dan unos…me dan los Boost. Si estoy bien me dicen no…pero no, no me dan una dieta así como… “come ahí to’ lo que tú quieras”.

**ENTREVISTADOR: Todo lo que tú quieras.**

Participante masculino 12: Sí.

**ENTREVISTADOR: Alguna recomenda…por ejemplo te manda a hacer estudios…**

Participante masculino 12: Sí. Sí, hepatitis todavía…y todo.

**ENTREVISTADOR: Hepatitis, diabetes, por ejemplo…**

Participante masculino 12: Sí, todo, todo. Gracias a Dios, estoy bien.

**ENTREVISTADOR: Corazón…**

Participante masculino 12: Sí, todo. Gracias a Dios, salgo bien de todo.

**ENTREVISTADOR: Y te los haces.**

Participante masculino 12: Sí.

**ENTREVISTADOR: ¿O alguno de ellos se te hace difícil?**

Participante masculino 12: No, me los hago, me los hago.

**ENTREVISTADOR: ¿Dónde te los haces? ¿Aquí mismo te los haces o tienes que…?**

Participante masculino 12: No, ella me hace los referidos y me los hago en diferentes sitios.

**ENTREVISTADOR: Y, por ejemplo, las citas…aparte de las veces cuando no te tomas los medicamentos, que te cansas, ¿hay alguna dificultad que hayas tenido para venir a las citas?**

Participante masculino 12: A veces confligir con mi trabajo y lo cambio. O pido la cita antes y pido el día libre, y vengo.

**ENTREVISTADOR: Okay. Hay algo más que quieras añadir al tema que hayamos hablado.**

Participante masculino 12: No.

**ENTREVISTADOR: ¿No?**

Participante masculino 12: No.

**ENTREVISTADOR: Okay, entonces culminamos acá la entrevista.**

Entrevista #13

Participante masculino

**ENTREVISTADOR: Ultima entrevista. Se…consintió el participante, se explicó el consentimiento informado y participante consintió y da el permiso para participar en la…en el estudio. Así que comenzamos con las primeras preguntas. ¿Desde cuándo estas tomando medicamentos para el VIH?**

Participante masculino 13: Del 2003.

**ENTREVISTADOR: ¿2003? ¿Ahí fue cuando te diagnosticaron la condición?**

Participante masculino 13: Sí

**ENTREVISTADOR: En el 2003. ¿Y empezaste a tomarte los medicamentos cuando te dieron el diagnostico o paso algún tiempo?**

Participante masculino 13: No.

**ENTREVISTADOR: Cuando te dieron el diagnóstico.**

Participante masculino 13: Sí.

**ENTREVISTADOR: ¿Y en el lugar donde te trataban tenían los medicamentos para el VIH? ¿Los tenían disponibles?**

Participante masculino 13: Sí.

**ENTREVISTADOR: ¿Y lees la información que explica, verdad, los efectos secundarios del medicamento?**

Participante masculino 13: Sí.

**ENTREVISTADOR: ¿De qué te acuerdas, más o menos, qué efectos secundarios?**

Participante masculino 13: Bueno, al principio da vómito, diarreas, nauseas, malestar.

**ENTREVISTADOR: ¿Te dio todo eso?**

Participante masculino 13: Sí.

**ENTREVISTADOR: ¿Sí? ¿Siempre te dan o te solamente al principio?**

Participante masculino 13: No, al principio.

**ENTREVISTADOR: ¿Y qué piensas de los medicamentos para el VIH? ¿Qué piensas de ellos?**

Participante masculino 13: Bueno, este, da resultados. Hay que seguirlos tomando.

**ENTREVISTADOR: ¿Algo más que piensas sobre ellos?**

Participante masculino 13: Sin eso uno no…no estuviéramos, no estuviéramos vivos. Estuviéramos 7 pies bajo tierra.

**ENTREVISTADOR: ¿Y qué tu esperas del tratamiento? ¿Cuáles son tus expectativas del tratamiento?**

Participante masculino 13: Pues, que pudiéramos…que se encuentre la cura pa’ curarnos.

**ENTREVISTADOR: Y del tratamiento que estas tomando ahora, ¿qué esperas?**

Participante masculino 13: Me ha venio’ bien.

**ENTREVISTADOR: Cuando escuchas comentarios de otras personas que han…que ya no se toman los medicamentos, que han dejado de tomarse, ¿qué tú piensas? Dejan de tomarse el medicamento para el VIH, ¿qué piensas de esas personas que han dejado de tomárselos?**

Participante masculino 13: Pue’, que quieren acabar con…terminar con sus vidas. Porque no ven…este…resultados.

**ENTREVISTADOR: ¿Algo más que piensas de ellos?**

Participante masculino 13: O los venden.

**ENTREVISTADOR: Venden los medicamentos, ¿sí? Los venden a… ¿tienen clientes ellos para vendérselos?**

Participante masculino 13: Los venden por…por droga o…

**ENTREVISTADOR: ¿Y a quien…que personas, por ejemplo, le pueden comprar los medicamentos a ellos?**

Participante masculino 13: Los de los punto’.

**ENTREVISTADOR: ¿Para vendérselo a otras personas que tienen la condición?¿ Y hay personas que no les cubre el plan médico o algo así? ¿Por eso los compran?**

Participante masculino 13: A mí, en parte, yo tengo que buscar afuera una…una parte del medicamento, porque aquí no me lo…

**ENTREVISTADOR: ¿No te lo cubre? Okay. Y las personas, por ejemplo, que pueden comprar medicamentos afuera son personas que, o sea, ¿por qué los comprarían ellos…para usarlos…?**

Participante masculino 13: Bueno, en parte lo compran pa’ decir que es pa’ uso, pero en parte no.

**ENTREVISTADOR: ¿Y esas son algunas de, verdad, las razones por la que algunas personas no se las toman…Por que las pueden vender?**

Participante masculino 13: Sí. Mejor los venden pa’…mejor prefieren más la droga que los mismos medicamentos.

**ENTREVISTADOR: Ah, okay, okay. Si porque en ese caso están, verdad, necesitados de la droga y eso. Y…porque yo lo había escuchado antes, por eso te pregunto, que yo te pregunto si los compran…quienes los compran, los que tú has escuchado, vedad, de la calle. Si los que los compran son personas que también tienen la condición, que no le cubre el plan médico o se usa para… o que es el medicamento se usa para aliviar otra cosa. ¿Qué has escuchado tú por ahí, en la calle?**

Participante masculino 13: No, los que los…los venden por…los que están en el…en la droga.

**ENTREVISTADOR: En la droga…para conseguir. Okay.**

Participante masculino 13: Muchos los compran y los sacan y lo dan pa’ alante

**ENTREVISTADOR: Ah okay, y no se sabe para qué. Ellos los venden pero no saben para dónde va el medicamento.**

Participante masculino 13: Aja.

**ENTREVISTADOR: Y qué razones, verdad, además de eso, los adictos que venden los medicamentos, que podrían estar vendiéndolos para…por la droga. ¿Qué otras razones dan las personas, verdad, para dejar de tomarse los medicamentos? Que tú hayas escuchado así en la calle.**

Participante masculino 13: Pue’ que no le funciona.

**ENTREVISTADOR: Okay. No le funcionan los medicamentos. ¿Alguna otra razón que tú has escuchado, así en la calle?**

Participante masculino 13: No.

**ENTREVISTADOR: ¿En algún momento tú has dejado, verdad, has pensado en dejar de tomarte los medicamentos, verdad, del tratamiento?**

Participante masculino 13: Ah, a veces cuando estoy deprimio’ no me los tomo.

**ENTREVISTADOR: Y además de cuando estas deprimido, que piensas, verdad, en dejar de tomártelos, ¿hay otra razón por la que piensas en dejar de tomarte los medicamentos?**

Participante masculino 13: No.

**ENTREVISTADOR: ¿No? Okay. ¿Y te has dejado de tomar los medicamentos en algún momento?**

Participante masculino 13: Sí.

**ENTREVISTADOR: ¿Cuántas veces has dejado de tomártelos?**

Participante masculino 13: Bueno depende, cuando se me acaban y no tengo chavo, pue’…tengo que esperar a conseguirlos. A veces pasan seis meses.

**ENTREVISTADOR: ¿En…los medicamentos en llegar?**

Participante masculino 13: No, conseguir los chavos.

**ENTREVISTADOR: Ah, en conseguir los chavos. Pero a ti te cobran… ¿tú tienes que pagar por el medicamento?**

Participante masculino 13: Lo que la farmacia…si no tengo boleta, se me vence pue’ tengo que esperar a venir a buscar acá.

**ENTREVISTADOR: ¿Esos son los del VIH? Para la condición. Okay. ¿Qué plan médico tu tienes?**

Participante masculino 13: Triple S.

**ENTREVISTADOR: ¿De Reforma o privado?**

Participante masculino 13: No, Reforma.

**ENTREVISTADOR: Ah, de Reforma, okay. ¿Y a veces…a veces el plan te…no te cubre algún medicamento o tienes problema con algún…?**

Participante masculino 13: No.

**ENTREVISTADOR: ¿Qué medicamento, por ejemplo, no te ha cubierto?**

Participante masculino 13: Bueno, los de…los de afuera.

**ENTREVISTADOR: Ah, okay, los de afuera. Los que te dan aquí te cubre.**

Participante masculino 13: Aquí me dan una…una boleta, entonces afuera tengo que buscar lo otro.

**ENTREVISTADOR: Cuando…se tarda tanto tiempo así en, en llegarte el medicamento, ¿alguna agencia te ayuda con, verdad, dándote, dándote algún, dándote el medicamento en lo que te llega? Por ejemplo, que te digan “no, este medicamento no lo tengo disponible, pero ve allá, a la clínica de inmunología a ver si lo tienen”, entonces en la clínica, aquí, por ejemplo, te lo dan.**

Participante masculino 13: No, aquí…aquí me suplen la…no me suplen to’. Aquí me suplen parte.

**ENTREVISTADOR: Parte. Y te acuerdas los que no te suplen?**

Participante masculino 13: Los que yo cojo afuera es, este…no me recuerdo.

**ENTREVISTADOR: Pero te acuerdas para que son, aunque no te acuerdes el nombre. Si te acuerdas.**

Participante masculino 13: Si, porque es de la misma condición.

**ENTREVISTADOR: De la misma condición. Okay, está bien. Todos son relacionados a la condición.**

Participante masculino 13: Sí.

**ENTREVISTADOR: O sea, que hay algunos que te los suplen y otros no, y esos que no te los suplen hay veces que estas tiempo sin tomártelos.**

Participante masculino 13: Sí.

**ENTREVISTADOR: O sea, que a veces es porque no tienes…no tienes, no te los suplen, por eso no te los tomas. Otras veces es porque estas deprimido también y no te los has tomado. ¿Alguna otra razón, por la cual, verdad, no te hayas tomado el medicamento, hayas dejado de tomártelo, además de que te deprimes o que no te los suplen?**

Participante masculino 13: Cuando me voy pa’ la, pa’ la escuela. No me da tiempo de tomármelos.

**ENTREVISTADOR: Ah, okay. O sea, que también cuando te vas allá. Y del 2003 para acá, ¿cuántas veces has dejado de tomártelos los medicamentos? Que has estado, verdad, un tiempo sin tomártelos.**

Participante masculino 13: Desde que…nos fuimos pa’…nos embarcamos pa’ fuera, pa’ allá fuera.

**ENTREVISTADOR: Eso fue una vez entonces.**

Participante masculino 13: No, seis, este…como pa’…como pa’ seis meses.

**ENTREVISTADOR: ¿Seis meses estuviste sin tomártelos?**

Participante masculino 13: Sí.

**ENTREVISTADOR: ¿Y ahora llevas cuánto tiempo tomándotelos? ¿O ahora, no te los has tomado hace seis meses atrás?**

Participante masculino 13: No. Ahora empecé cuando volví otra vez pa’ ‘tras. Desde septiembre pa’ acá.

**ENTREVISTADOR: Okay, desde septiembre para acá te los estas tomando. ¿Y seis meses ha sido el tiempo que más…el más…lo más que has estado sin tomarte los medicamentos? ¿Seis meses?**

Participante masculino 13: Aja.

**ENTREVISTADOR: ¿Y lo menos que has estado sin tomártelos, cuántos meses fueron? ¿O cuánto tiempo fue?**

Participante masculino 13: Cuando se me acaban.

**ENTREVISTADOR: Cuando se te acaban.**

Participante masculino 13: Sí, porque las citas me dan cada tres meses.

**ENTREVISTADOR: Y a veces se te acaban y no llegan al tiempo cuando…o sea, cuando se te acaban a veces no tienen los medicamentos.**

Participante masculino 13: No. Tengo que esperar a…a venir a la cita para entonces pedir refill.

**ENTREVISTADOR: Y dosis, por ejemplo, que se te haya olvidado tomarte una pastilla, un día. Por ejemplo... ¿eso te ha pasado, que no te la hayas tomado?**

Participante masculino 13: Cuando vengo bien cansao’ de la escuela, no me las tomo.

**ENTREVISTADOR: A ti te dan cuántas pastillas al día.**

Participante masculino 13: Son…son cinco…cinco frascos.

**ENTREVISTADOR: Cinco frascos, okay. Y te tienes que tomar cinco pastillas al día entonces. ¿Y te las tomas todas de cantazo o tienes una por la mañana, otra por la tarde…cómo son? ¿Cómo te dice el medico que te las tomes?**

Participante masculino 13: No, una es por la mañana, y una a la comida. Entonces, y una por la noche.

**ENTREVISTADOR: Que son como tres veces al día que tienes que tomar alguna de esas pastillas. ¿Y alguna de esas veces se te olvida tomártelas? Me dices cuando llegas de la escuela cansado, ¿verdad? Okay. Te pasa…te pasa casi todo los días, o te pasa de vez en cuando…**

Participante masculino 13: De vez en cuando.

**ENTREVISTADOR: De vez en cuando. Okay. Como estábamos hablando, verdad, a veces hay muchas personas que se le hace difícil tomarse las pastillas todos los días por muchas razones que están dentro, verdad, y fuera del control de la persona. Y ahora te voy a hacer unas preguntas sobre unas posibles razones por la que en algún momento tú hayas dejado de tomarte los medicamentos. Okay. Quiero que pienses en ti, sin incluir a otras personas o circunstancias, solamente, verdad, en ti. ¿Consideras que tengas algún sentimiento o algún pensamiento o creencia personal que haya influido en, verdad, que te haya influido para dejar de tomarte los medicamentos?**

Participante masculino 13: Al principio.

**ENTREVISTADOR: ¿Qué pasó al principio?**

Participante masculino 13: Cuando me dijeron la condición.

**ENTREVISTADOR: ¿Cómo te sentías en ese momento?**

Participante masculino 13: Vacío y sucio.

**ENTREVISTADOR: ¿Y de alguna manera eso, pues, te hacía pensar como que, verdad, o no querer tomarte los medicamentos?**

Participante masculino 13: Sí.

**ENTREVISTADOR: ¿Y no te los tomabas, cuando pensabas eso? ¿No?**

Participante masculino 13: No.

**ENTREVISTADOR: Okay. No te los tomabas. O sea, que pensabas que te sentías vacío, pues, te sentías sucio en ese momento.**

Participante masculino 13: Sí.

**ENTREVISTADOR: Me dijiste orita de depre…que a veces te deprimías, no, cuando te dijeron la condición, ¿te sentiste así, deprimido?**

Participante masculino 13: Sí.

**ENTREVISTADOR: ¿Sí? ¿Recibiste tratamiento en ese momento para la depresión?**

Participante masculino 13: No.

**ENTREVISTADOR: ¿No? Y después que, después que, verdad te dijeron la…sobre la condición, cuantas veces tú te has sentido como que te has deprimido en estos, ¿cuánto llevas? Vas para 13 años, 12 años…bueno 10 años con la condición. Estos 10 años, o 9 años que llevas con la condición, ¿Cuántas veces te has sentido así deprimido como esa vez cuando te diagnosticaron?**

Participante masculino 13: A cada rato.

**ENTREVISTADOR: Y cuando te sientes así, ¿te da por no tomártelos, los medicamentos?**

Participante masculino 13: Es que me voy caminar y se me olvida.

**ENTREVISTADOR: Lo piensas, pero no siempre lo haces. Y además, verdad, de sentirte así esas veces que…que has dejado de tomártelos, verdad, ¿qué estaba pasando en tu vida, en ese momento?**

Participante masculino 13: Me siento infeliz.

**ENTREVISTADOR: Y entonces ahí, pues piensas o dejas de tomártelos. ¿Algo más, que, verdad, de cosas que tú piensas o como te sientas, que haya influido en que dejes de tomarte los medicamentos?**

Participante masculino 13: No.

**ENTREVISTADOR: ¿Tú has estado…te ha…no has ido nunca a un psicólogo, o sí? Para tratarte los sentimientos de tristeza de depresión.**

Participante masculino 13: No.

**ENTREVISTADOR: ¿No? Okay, y ahora…**

Participante masculino 13: Fui…estuve una vez en la Playa, pero no…no me…me ayudó.

**ENTREVISTADOR: ¿No te ayudo estar en un psicólogo? Okay.**

Participante masculino 13: Porque iba a las cita y salía igual.

**ENTREVISTADOR: ¿En algún momento te dieron algún medicamento para tratar la depresión?¿Un psiquiatra?**

Participante masculino 13: No.

**ENTREVISTADOR: Okay. ¿Algo más de eso…del...verdad, de cómo te sentías, de todas…de todo?**

Participante masculino 13: No.

**ENTREVISTADOR: Bueno, pues ahora vamos a hablar, verdad, de las personas con las que tú te relacionas, la que usualmente están al lado tuyo, la que tú te relacionas. ¿De qué manera, si alguna, alguna de estas personas con la que tú te relacionas influyeron en que tu dejaras de tomarte los medicamentos?**

Participante masculino 13: No.

**ENTREVISTADOR: ¿No? Okay. Tu familia… ¿tienes familia acá, en Puerto Rico?**

Participante masculino 13: Mi mamá.

**ENTREVISTADOR: Tu mamá, Okay. ¿Vives con tu mamá?**

Participante masculino 13: Sí.

**ENTREVISTADOR: ¿Ella sabe que tienes el diagnostico? ¿Sí? Okay.**

Participante masculino 13: Ella es la que me acompaña, pero hoy no vino.

**ENTREVISTADOR: Ah, Okay. Okay. ¿Algún familiar, verdad, tú crees que algún familiar de alguna manera ha influido indirectamente, tu sabes, en que tú digas “ay mira, tu sabes, no me lo voy a tomar. Pasó esto con esta personas y lo voy a coger con el tratamiento, que se yo, no me lo tomo”. ¿Te ha pasado eso?**

Participante masculino 13: Eh…mi hermana.

**ENTREVISTADOR: Tu hermana.**

Participante masculino 13: Con la que yo me fui ahora en septiembre.

**ENTREVISTADOR: Ah, Okay, Okay. ¿Tuviste algún problema con ella?**

Participante masculino 13: Una discusión…una pelea que tuvimos entre ella y yo.

**ENTREVISTADOR: Y después que tuvieron esa pelea, ¿pensaste en dejar de tomártelos o dejaste de tomártelos?**

Participante masculino 13: Dejé de tomármelos.

**ENTREVISTADOR: ¿Por qué?**

Participante masculino 13: Porque llamé a la…a la farmacia para que lo descontinuaran.

**ENTREVISTADOR: Algún…algo así…alguna situación así, similar a esa, ya sea con un familiar o con una amistad, que te haya pasada y te haya pasado lo mismo. Como que te…**

Participante masculino 13: No.

**ENTREVISTADOR: ¿No? Okay. Y, por ejemplo, los…los médicos que te atienden, digamos, verdad, que en algún momento te haya atendido algún médico y tú como que no te gustó y decidiste pues no…cancelar lo de los medicamentos…algo así, ¿te ha pasado eso?**

Participante masculino 13: En la Playa.

**ENTREVISTADOR: ¿Sí? Okay. Cuéntame de esa experiencia. ¿Qué paso?**

Participante masculino 13: Que cada vez que iba…me daban los medicamentos incompletos.

**ENTREVISTADOR: Ah, Okay. ¿Y alguna razón por la que te los daban incompletos?**

Participante masculino 13: Que no los tenía o que el suplidor no los había traído.

**ENTREVISTADOR: Y cuando te los daban incompletos, ¿Qué tu hacías?**

Participante masculino 13: Na’, me tomaba lo que tenía y…

**ENTREVISTADOR: ¿Y cuánto tiempo tenías que esperar a lo que…en lo que llegaba?**

Participante masculino 13: Hasta que ellos le saliera…llamaran a buscarlos.

**ENTREVISTADOR: ¿Sí? Y eso era como cuánto tiempo, ¿un mes, dos meses?**

Participante masculino 13: Como un mes, por ahí.

**ENTREVISTADOR: Un mes. Y por ejemplo, alguno que te hayas sentido que te haya tratado mal y que de ahí entonces decidiste no ir y no buscar los medicamentos. ¿Te ha pasado eso?**

Participante masculino 13: No.

**ENTREVISTADOR: O algún, enfermero o personal de la clínica…**

Participante masculino 13: No.

**ENTREVISTADOR: Alguna otra personas que en algún momento por la manera como…tuvieron alguna situación y tú, pues, decidiste “no voy…voy a cancelar lo de los medicamentos”.**

Participante masculino 13: No.

**ENTREVISTADOR: Ahora, piensa en el ambiente donde tú te desenvuelves, donde tu estas, verdad. ¿De qué manera, si alguna el ambiente donde tú te desenvuelves influyó en la decisión de dejar de tomar los medicamentos alguna de esas veces que los dejaste de tomar?**

Participante masculino 13: No.

**ENTREVISTADOR: La…la comunidad donde vives, verdad, ¿sientes que de alguna manera eso, pues, tiene alguna influencia indirecta en de dejar de tomar los medicamentos?**

Participante masculino 13: No.

**ENTREVISTADOR: Me dijiste que estudiabas, verdad.**

Participante masculino 13: Sí.

**ENTREVISTADOR: ¿Trabajas también?**

Participante masculino 13: No.

**ENTREVISTADOR: Solamente estudias. Y en donde…y tu estudias por la mañana, por la tarde o por la noche.**

Participante masculino 13: Por la mañana.

**ENTREVISTADOR: Por la mañana. ¿Te llevas los medicamentos para la universidad?**

Participante masculino 13: No.

**ENTREVISTADOR: No, o sea, te los tomas antes de ir a la universidad. Este…y los que te tocan en la tarde…cuando te tocan, ya no estas estudiando, ¿verdad?**

Participante masculino 13: No.

**ENTREVISTADOR: Okay. Lo que me dijiste orita es que llegabas cansado y a veces se te olvidaba tomártelas. Y cuando llegas cansado y se te olvida tomártelos, te lo…cuando te acuerda, ¿te los tomas o esperas al otro día?**

Participante masculino 13: No, cuando…cuando me recuerdo me los tomo.

**ENTREVISTADOR: Okay. En esta semana, ¿como cuántas veces te ha pasado eso?**

Participante masculino 13: De…

**ENTREVISTADOR: Del lunes pasado hasta hoy.**

Participante masculino 13: De miércoles pa’ acá.

**ENTREVISTADOR: Del miércoles para acá, Okay. ¿Todos los días te ha pasado? Okay. Y, por ejemplo, en el ambiente donde tu socializas, como dicen por ahí, donde tu jangeas, tú crees… ¿de alguna manera ese ambiente, pues, influye directa o indirectamente, ya sea que no te los tomes, o que se te olvide tomarte alguna dosis o que digas “no voy a tomar más el tratamiento”?**

Participante masculino 13: No.

**ENTREVISTADOR: ¿Algún otro lugar que influya?**

Participante masculino 13: No.

**ENTREVISTADOR: Ahora quiero que pienses en Puerto Rico, verdad, todo Puerto Rico. En las cosas que están pasando en Puerto Rico. ¿De qué manera, si alguna, las cosas que pasan en Puerto Rico influyen en la decisión de algunas personas, puede ser tú o puede ser otras personas, de algunas personas…o sea, de qué manera, si alguna, las cosas que pasan en Puerto Rico influyen en la decisión de algunas personas en dejar de tomarse los medicamentos para el VIH?**

Participante masculino 13: No creo.

**ENTREVISTADOR: El gobierno, por ejemplo, ¿crees que tiene alguna influencia el gobierno de Puerto Rico, las cosas que pasan en el gobierno?**

Participante masculino 13: Bueno, en parte tienen la culpa los gobiernos, porque no hay trabajo.

**ENTREVISTADOR: ¿Y el no tener trabajo a veces eso puede influir en las personas que tienen la condición en no tomarse los medicamentos?**

Participante masculino 13: Sí, porque de donde van a suplir los medicamentos.

**ENTREVISTADOR: Y los servicios, por ejemplo, los servicios de salud del país, que… ¿de alguna manera crees que influyen?**

Participante masculino 13: No creo.

**ENTREVISTADOR: ¿Y los planes médicos? Puede ser que deje de tomárselos o a lo menor no tenga el medicamento.**

Participante masculino 13: Muchos no tienen planes.

**ENTREVISTADOR: Y esas personas que no tienen planes, ¿qué hacen?**

Participante masculino 13: No sé.

**ENTREVISTADOR: Y la situación económica del país, ¿de alguna manera influirá?**

Participante masculino 13: Sí.

**ENTREVISTADOR: ¿En qué? ¿Cómo tú crees que influye?**

Participante masculino 13: Pues, no hay trabajo…

**ENTREVISTADOR: Transportación… ¿piensas que hay un problema de transportación de las personas que…para, verdad, tengan transportación, que no tengan transportación y no puedan llegar a tomar…a buscar los medicamentos o a las citas?**

Participante masculino 13: Si viven en sitios lejos, sí.

**ENTREVISTADOR: ¿Te ha pasado eso a ti?**

Participante masculino 13: Cuando no tengo el dinero pa’ transportación.

**ENTREVISTADOR: Para transportación. Y, ¿cómo haces?**

Participante masculino 13: A veces llamo aquí pa’ que me manden el taxi y a veces no…no…cojo chavos prestao’.

**ENTREVISTADOR: ¿Alguna otra situación que puedan influir, que crees que pueda influir?**

Participante masculino 13: No.

**ENTREVISTADOR: Y ahora, quiero que pienses en cosas como las costumbres, las creencias de nuestra sociedad, verdad. ¿De qué manera, si alguna, las costumbres, las creencias de nuestra sociedad influyen en la decisión de dejar de tomarse los medicamentos para el VIH?**

Participante masculino 13: Otros por medio a que la familia no lo sepa que tiene la condición. No acuden a las citas. O qué dirán la sociedad porque están tomando…están bebiendo medicamentos.

**ENTREVISTADOR: Okay. ¿Te has sentido discriminado por tener la condición?**

Participante masculino 13: Sí.

**ENTREVISTADOR: El sentirte, verdad, discriminado, rechazado por tener la condición, ¿eso en algún momento ha influido para que pienses o dejes de tomarte los medicamentos?**

Participante masculino 13: No.

**ENTREVISTADOR: Y, por ejemplo, cosas como las creencias religiosas, ¿tú crees que de alguna manera influyen en, verdad, la decisión de la personas de tomarse los medicamentos?**

Participante masculino 13: Bueno…en parte, en el caso mío, con mi papá…no…no quiso aceptar la…que yo salí con la condición, pue’ empezó a rechazarme.

**ENTREVISTADOR: ¿Tu papá?**

Participante masculino 13: Sí.

**ENTREVISTADOR: Y tu papá, ¿vive aquí o vive en Estados Unidos?**

Participante masculino 13: Estados Unidos.

**ENTREVISTADOR: ¿En algún momento acudiste a alguna iglesia o algo para…cuando…?**

Participante masculino 13: No.

**ENTREVISTADOR: ¿No? Okay. ¿Y alguna razón en específica para no acudir a la iglesia cuando tu…en este tiempo que has estado con la condición?**

Participante masculino 13: No.

**ENTREVISTADOR: Y por ejemplo, el nivel socioeconómico de una persona, ¿tú crees que eso influye en la decisión de tomarse los medicamentos? Situación socioeconómica.**

Participante masculino 13: No creo.

**ENTREVISTADOR: ¿Alguna otra, verdad, costumbre o creencias, de nuestra sociedad o situaciones de nuestra sociedad que influyeran en la decisión de tomar los medicamentos?**

Participante masculino 13: No.

**ENTREVISTADOR: Okay, ahora quiero que pienses en los tiempos que estamos viviendo ahora, estos tiempos. ¿De qué manera, si alguna, estos tiempos en que vivimos influye en la decisión de tomar los medicamentos para el VIH? Estos tiempos de ahora.**

Participante masculino 13: Pa’ buscar la…tener una buena vida…una…y estar…vivir un poquito más.

**ENTREVISTADOR: O sea, que eso influye en la decisión. Algo más, cosas que pasan en estos tiempos…que influyan en esa decisión de tomar los medicamentos o de dejar de tomar los medicamentos.**

Participante masculino 13: No.

**ENTREVISTADOR: Ahora, cuando en el pasado, verdad… ¿Cuántas veces fue que me dijiste que te habías dejado de tomar los medicamentos por mucho tiempo? Hablaste de la vez cuando te diagnosticaron, después cuando estuviste con tu hermana. Además de esas dos veces, ¿Cuántas veces más, en estos 9 años?**

Participante masculino 13: Aquella vez.

**ENTREVISTADOR: ¿Dos veces?**

Participante masculino 13: Sí.

**ENTREVISTADOR: Cuando en el pasado dejaste de tomarte los medicamentos para el VIH, o sea, esas dos veces, ¿Qué te ayudó a volver a tomártelos? ¿Qué fue lo que pasó que dijiste “Okay, ya me los tengo que tomar, voy a empezar a tomármelos”?**

Participante masculino 13: Cuando me enteré que me dio una fiebre de frío.

**ENTREVISTADOR: Ah, Okay, cuando empezaste a sentir entonces…Okay. O sea, que el sentir ya unos síntomas te ayudó.**

Participante masculino 13: Sí.

**ENTREVISTADOR: ¿Y algo más que haya pasado en ese momento, además de eso? Porque hay personas que aún lo experimentan y no se los toman. Pero, ¿qué otras cosas te ayudaron, además de eso?**

Participante masculino 13: No, más na’.

**ENTREVISTADOR: Y esta segunda vez, que te fuiste para allá afuera, para la casa de tu hermana, ¿Qué fue lo que te ayudó a que tu volvieras a tomarte los medicamentos?**

Participante masculino 13: El doctor que me atendió.

**ENTREVISTADOR: Okay. ¿Qué hizo el doctor? ¿Qué fue lo que hizo el doctor que te ayudó?**

Participante masculino 13: Pue’ me dijo que yo…que me faltaba poco de vida.

**ENTREVISTADOR: Ah, Okay. Y ahí entonces tú lo…tú decidiste…Algo más que te haya ayudado, además de eso.**

Participante masculino 13: No.

**ENTREVISTADOR: ¿Qué tú le dirías a una persona que está pensando en dejar de tomarse los medicamentos?**

Participante masculino 13: Que no los deje.

**ENTREVISTADOR: ¿Por qué?**

Participante masculino 13: Porque ese es el…que por lo menos tiene un poquito más de vida.

**ENTREVISTADOR: Okay. ¿Y qué te dicen los médicos ahora, en cuanto a tu expectativa de vida?**

Participante masculino 13: Que tengo el…el virus controlao’.

**ENTREVISTADOR: Lo tienes controlado. Que tienes…que está…**

Participante masculino 13: Estable.

**ENTREVISTADOR: Estas estable. O sea, que esperan que vivas más tiempo entontes. Wow.**

Participante masculino 13: Poniendo a Dios por delante.

**ENTREVISTADOR: Wow. Y, ¿cómo se siente eso? Porque en aquel momento, cuando te dijo eso…sabe, que te estabas muriendo, básicamente. ¿Cómo tú te…qué tú pensaste, que tú dijiste “ay”…?**

Participante masculino 13: Por lo menos tengo un poquito de aliento.

**ENTREVISTADOR: ¿Sí? Y ese momento, que él te dijo eso. Tú estabas con ese médico, y él te dijo… ¿cómo fue que dijo? Tenías…**

Participante masculino 13: Poco tiempo de vida.

**ENTREVISTADOR: Que tenías poco tiempo de vida. ¿Qué tu pensaste en ese momento?**

Participante masculino 13: Que estaba loco.

**ENTREVISTADOR: ¿El doctor?**

Participante masculino 13: (se ríe)

**ENTREVISTADOR: No lo creías. Okay. Pero te empezaste a tomar los medicamentos, entonces.**

Participante masculino 13: Cuando…regresé aquí y empecé a tomármelos…

**ENTREVISTADOR: Aja. Y eso fue en septiembre del año pasado.**

Participante masculino 13: Sí.

**ENTREVISTADOR: ¿Y tenías algún síntoma en ese momento, cuando él te dijo eso, que te quedaba poquito de vida? Como aquella vez te dio fiebre, esta vez te dio algo…**

Participante masculino 13: No.

**ENTREVISTADOR: ¿Y por qué tu volviste a la clínica? ¿Por qué viniste en ese momento?**

Participante masculino 13: Porque cuando yo regrese aquí, estaba como Cristo en la cruz.

**ENTREVISTADOR: ¿Cómo es eso?**

Participante masculino 13: Flaco, se me veían to’as las costillas.

**ENTREVISTADOR: Ah, ¿sí? Ah. Okay.**

Participante masculino 13: Aquí me volvieron a levantar otra vez.

**ENTREVISTADOR: ¿Sí? Wow. Y ahí fue cuando decidiste venir…Flaco porque no estabas comiendo o porque era parte de los síntomas de, verdad, de no tomártelos.**

Participante masculino 13: De…de las tres cosas. Por la noticia que el doctor me dio allá afuera, y porque quería…dejar de tirarme en la cama…

**ENTREVISTADOR: Aja, y dejarte morir. Sin embargo, cuando dijiste al médico, no lo querías creer.**

Participante masculino 13: (se ríe)

**ENTREVISTADOR: Eso es que dentro de ti tienes esa esperanza de vida. Está ahí dentro esa llamita, está ahí. Yo creo que te hice esta pregunta, verdad, ¿Qué le dirías a una persona que está pensando en dejar de tomar los medicamentos?**

Participante masculino 13: Sí.

**ENTREVISTADOR: Ya te la hice. ¿Y has tenido la oportunidad de hablar con personas que han dejado de tomarse los medicamentos?**

Participante masculino 13: No.

**ENTREVISTADOR: Y si tuvieras la oportunidad, ¿qué le recomendarías a esas personas? Si alguien te dice “mira ya yo no me los estoy tomando, no me los quiero tomar, voy a dejar de tomarme eso”.**

Participante masculino 13: Que no los deje. Que siga luchando.

**ENTREVISTADOR: ¿Y por qué? ¿No los deje por qué?**

Participante masculino 13: Porque va a hacer sufrir a su familia, si tiene alguna.

**ENTREVISTADOR: ¿Tú habías escuchado de la palabra “adherencia al tratamiento” antes?**

Participante masculino 13: No.

**ENTREVISTADOR: ¿No? Y por ejemplo, cuando escuchas la frase “cumplimiento de las recomendaciones médicas”, ¿con qué lo relacionas?**

Participante masculino 13: Bueno…directrices que da el doctor.

**ENTREVISTADOR: Okay.**

Participante masculino 13: Los pasos.

**ENTREVISTADOR: Okay. Además de tomarte los medicamentos para el VIH, ¿qué más…qué cosas te dice tu médico que hagas? ¿Qué te recomienda? Además de tomarte los medicamentos,**

Participante masculino 13: Que no puedo mojarme en la lluvia…ni amanecerme ni trasnocharme…de noche.

**ENTREVISTADOR: Ah, Okay. ¿Y qué otras cosas más te dice?**

Participante masculino 13: Que si esta con otro…que si esta con otra pareja, protegerme.

**ENTREVISTADOR: Algunas de esas cosas que te recomienda el médico, ¿se te hace difícil seguirlas?**

Participante masculino 13: No.

**ENTREVISTADOR: ¿No? Okay. Te manda a hacer exámenes médicos?**

Participante masculino 13: Sí.

**ENTREVISTADOR: ¿Qué te manda a hacer, qué te manda a chequear?**

Participante masculino 13: Me hace los CD4 y laboratorios afuera.

**ENTREVISTADOR: Te manda…te manda… (Alguien afuera habla: “Estaba buscando. ¿Falta mucho?”) No estamos ya…como quizás, 10 minutos máximos. Te manda a hacer, por ejemplo, en los laboratorios que te manda a hacer, te manda a hacer pruebas del corazón, para el corazón, o te manda a hacer para la diabetes… ¿cosas así?**

Participante masculino 13: Sí.

**ENTREVISTADOR: ¿Y te los haces, esos, o hay alguno que no te lo has podido hacer por la razón que sea?**

Participante masculino 13: No. Yo me los hago.

**ENTREVISTADOR: Pasa tiempo después que te da la orden o te los hace rapidito?**

Participante masculino 13: No.

**ENTREVISTADOR: No, ¿cuál de las dos?**

Participante masculino 13: Cuando…porque se supone que cuando me llegue la hora de la cita me los tengo que hacer. Un día antes de venir a ver al médico.

**ENTREVISTADOR: Ah, Okay. ¿Y te los haces entonces?**

Participante masculino 13: Sí.

**ENTREVISTADOR: ¿Has tenido que faltar alguna cita, por alguna razón?**

Participante masculino 13: No.

**ENTREVISTADOR: O sea, ¿se te hace difícil venir a las citas?**

Participante masculino 13: Bueno…en el horario de clases.

**ENTREVISTADOR: Oh, Okay. Cuando tienes clases, que es por la mañana, ¿verdad? ¿Hoy tenías clases? O sea, que tuviste que faltar. ¿Hay algo más que se te hace difícil dentro de las recomendaciones médicas?**

Participante masculino 13: No.

**ENTREVISTADOR: O hay algo más que quieras añadir sobre lo que estamos hablando, que yo no te he preguntado y que tu entiendas que es importante decir?**

Participante masculino 13: No.

**ENTREVISTADOR: Pues, entonces culminamos con la entrevista.**

Participante masculino 13: Este…usted está en la escuela de…

**ENTREVISTADOR: De Medicina.**

Participante masculino 13: Que queda después…al frente de Damas.

**ENTREVISTADOR: Sí. Allí mismo. A la orden.**

Participante masculino 13: Sí, porque es que…es que yo parezco de los nervios por mi papá. Porque…estando mi mamá embrazada de mí, él le puso los pies en la barriga pa’ abortar.

**ENTREVISTADOR: Para abortar, Okay.**

Participante masculino 13: Y a través de eso, yo soy nervioso. El maestro mío me dijo que tratara de conseguir una cita con…no sé. A ver si podía meterme en tratamiento pa’…

**ENTREVISTADOR: Sabes lo que podemos hacer. Si quieres yo puedo hablar con El manejador de caso…ahora, para que te haga el referido. Porque aquí hay psicólogos, nosotros tenemos unos psicólogos que vienen de la Escuela de Medicina de Ponce. O sea, que no tendrías que tener que ir hasta allá. Ya ellos están aquí, precisamente para eso, porque a veces hay participantes que no pueden llegar hasta allá, pues pueden llegar hasta aquí. Entonces para que te haga la cita con uno de los psicólogos y si necesitaras, verdad, tratamiento…porque a veces eso pasa, que a veces si uno está muy muy nervioso pues a veces lo que uno tiene el tratamiento con el psicólogo y también psiquiatra. Pues entonces el psicólogo te hace el referido para el psiquiatra. Pero si tú me das permiso, yo hablo con El manejador de caso ahora, entonces para que él te haga la cita con alguno de los psicólogos de aquí. ¿Qué tú crees? Porque lo tienes aquí mismo, lo tienes…**

Participante masculino 13: Sí, porque yo hable con el psicólogo de aquí, pero él me dijo que él tenía que referirme a…a un psicólogo…a un psicólogo de afuera.

**ENTREVISTADOR: Ah, Okay. Quizás al psiquiatra. Está bien. Yo hablo con El manejador de caso, si quieres yo hablo con El manejador de caso a ver que se puede hacer. Porque entonces si es psiquiatra, pues necesitas hacerlo a través de la reforma, verdad, porque tú tienes la reforma, ¿verdad?**

Participante masculino 13: Sí.

**ENTREVISTADOR: Y los servicios psiquiátricos…este, los dan al lado de la Escuela de Medicina de Ponce, sabes…en la Escuela de Medicina, pero entonces tiene que ser a través de un referido. Pero yo puedo hablar con El manejador de caso. Si quieres hablamos…o yo hablo con él, verdad.**

Participante masculino 13: Ujum.

**ENTREVISTADOR: Para que entonces puede hacerte la gestión y puedas hacerte…de hacerte la coordinación con el psiquiatra. Si es así. Porque si es psicólogo, pues hay unas que vienen aquí, que son de la Escuela de Medicina de Ponce. Que te podrían atender. Si es psiquiatra, pues entonces ya…ellas te puedan hacerte la coordinación para hacerte la cita y todo eso…pero ya sería este…tienes que usar la reforma porque pues, el plan médico que ellos cogen es la Reforma y entonces sería una cita aparte con el psiquiatra. Pero yo hablo con él. Yo hablo con él y le explico.**

Participante masculino 13: Sí, porque el maestro me dijo “tienes que ir al…psiquiatra pa’ que te de un tratamiento pa’ los nervios”.

**ENTREVISTADOR: Sí, a veces, cuando son nervios así trabajan los dos, el psicólogo y el psiquiatra, los dos.**

Participante masculino 13: Porque como lo del trabajo mío es de…de recortar y bregar con navajas…ese es el miedo mío de yo coger y…

**ENTREVISTADOR: Sí, sí. Y atendiendo…porque en todo momento estas nervioso. Te tiemblan las manos y todo eso.**

Participante masculino 13: Sí.

**ENTREVISTADOR: Esta bien, pues yo hablo con El manejador de caso ahora, si me das permiso. ¿Me das permiso?**

Participante masculino 13: Sí.

**ENTREVISTADOR: Ok. Pues yo hablo con El manejador de caso a ver como se te puede coordinar el servicio entonces.**

Participante masculino 13: Porque yo hable con la doctora mía de cabecera y me dio unas pastillitas chiquitas de…

**TERMINA LA GRABACION.**
